# Molecular phylogeny of the bivalve superfamily Galeommatoidea (Heterodonta, Veneroida) reveals dynamic evolution of symbiotic lifestyle and interphylum host switching

**DOI:** 10.1186/1471-2148-12-172

**Published:** 2012-09-06

**Authors:** Ryutaro Goto, Atsushi Kawakita, Hiroshi Ishikawa, Yoichi Hamamura, Makoto Kato

**Affiliations:** 1 Graduate School of Human and Environmental Studies, Kyoto University, Yoshida-Nihonmatsu-cho, Sakyo, Kyoto, 606-8501, Japan; 2 Department of Marine Ecosystem Dynamics, Atmosphere and Ocean Research Institute, The University of Tokyo, 5-1-5 Kashiwa-no-ha, Kashiwa, Chiba, 277-8564, Japan; 3 Center for Ecological Research, Kyoto University, 2-509-3 Hirano, Otsu, Shiga, 520-2113, Japan; 4 965-1 Kawachi-ko, Uwajima, Ehime, 798-0075, Japan; 5 14-16 Yakeyama-Hibarigaoka-cho, Kure, Hiroshima, 737-0901, Japan

**Keywords:** Bivalvia, Commensalism, Diversification, Galeommatoidea, Parallel evolution, Symbiosis, Host specialization, Host switching

## Abstract

**Background:**

Galeommatoidea is a superfamily of bivalves that exhibits remarkably diverse lifestyles. Many members of this group live attached to the body surface or inside the burrows of other marine invertebrates, including crustaceans, holothurians, echinoids, cnidarians, sipunculans and echiurans. These symbiotic species exhibit high host specificity, commensal interactions with hosts, and extreme morphological and behavioral adaptations to symbiotic life. Host specialization to various animal groups has likely played an important role in the evolution and diversification of this bivalve group. However, the evolutionary pathway that led to their ecological diversity is not well understood, in part because of their reduced and/or highly modified morphologies that have confounded traditional taxonomy. This study elucidates the taxonomy of the Galeommatoidea and their evolutionary history of symbiotic lifestyle based on a molecular phylogenic analysis of 33 galeommatoidean and five putative galeommatoidean species belonging to 27 genera and three families using two nuclear ribosomal genes (18S and 28S ribosomal DNA) and a nuclear (histone H3) and mitochondrial (cytochrome oxidase subunit I) protein-coding genes.

**Results:**

Molecular phylogeny recovered six well-supported major clades within Galeommatoidea. Symbiotic species were found in all major clades, whereas free-living species were grouped into two major clades. Species symbiotic with crustaceans, holothurians, sipunculans, and echiurans were each found in multiple major clades, suggesting that host specialization to these animal groups occurred repeatedly in Galeommatoidea.

**Conclusions:**

Our results suggest that the evolutionary history of host association in Galeommatoidea has been remarkably dynamic, involving frequent host switches between different animal phyla. Such an unusual pattern of dynamic host switching is considered to have resulted from their commensalistic lifestyle, in which they maintain filter-feeding habits even in symbiotic habitats. The results of the molecular phylogenetic analysis did not correspond with the current taxonomic circumscription. Galeommatidae and Lasaeidae were polyphyletic, and *Basterotia*, which is traditionally assigned to Cyamioidea, formed a monophyletic clade within Galeommatoidea.

## Background

Symbiotic relationships between animals are ubiquitous and diverse in the sea [1-5] and play an important role in shaping the spatial pattern and structure of marine biodiversity [1-5]. At evolutionary timescales, colonization of novel animal hosts may lead to speciation and thus constitutes a primary process driving the diversification of symbiotic animals [6-8]. Although the pattern and process of host switching in the terrestrial ecosystem have been extensively studied, most notably in herbivore insects [9], little is known about how symbiotic marine organisms expand their host ranges and diversify via host switching.

Bivalves are a group of mollusks that are generally filter feeders and exist in various marine habitats [10]. Among the bivalves, the superfamily Galeommatoidea is unique in terms of its symbiotic association with various benthic and burrowing invertebrates [11,12]. They utilize these hosts as a home or shelter and benefit from the water currents created by the host, which are rich in oxygen and food particles [12]. The host taxa of Galeommatoidea are extremely diverse and include the phyla Porifera, Cnidaria, Brachiopoda, Bryozoa, Annelida, Mollusca, Arthropoda, and Echinodermata [11-13]. The ecological modes of host utilization by Galeommatoidea are also diverse and include ectosymbionts, endosymbionts, and burrow associates [11-13]. Furthermore, Galeommatoidea includes many free-living species that often attach to the undersurfaces of rocks in the intertidal zone [14,15], and some species are capable of active movement on the substrates by using their muscular foot [15]. An intriguing question is how these diverse host associations and lifestyles evolved in this superfamily.

The classification of Galeommatoidea is in a state of flux, mostly likely owing to their reduced, and sometimes highly modified, morphologies associated with symbiotic life. Often more than one family is recognized within the superfamily, but these groupings are usually ill-defined when a range of characters are considered [16]. The most recent classification divides Galeommatoidea into two families, Galeommatidae and Lasaeidae, although there is little morphological support for these families [17]. On the basis of this classification, the previously recognized families Kelliidae and Montacutidae are included in Lasaeidae. Furthermore, recent anatomical and ecological studies suggest that the genera *Peregrinamor* and *Basterotia*, which were traditionally classified into Mytiloidea and Cyamioidea, respectively [18], should be included in Galeommatoidea [19,20]. Further investigation is required to resolve these taxonomic issues.

This study aimed to reveal the diversification history of Galeommatoidea and to resolve the taxonomic confusion surrounding this bivalve superfamily. We conducted a molecular phylogenetic analysis using two nuclear ribosomal genes (18S and 28S ribosomal DNA) and one nuclear (histone 3, H3) and one mitochondrial (cytochrome oxidase subunit I, COI) protein-coding genes in galeommatoidean bivalves sampled from a broad range of host animals and habitats in the northwest Pacific Ocean. The resulting phylogenetic tree uncovers an unexpectedly dynamic pattern of host switching and ecological specialization in Galeommatoidea.

## Results and discussion

### Molecular phylogenetic analysis

**Figure 1:**
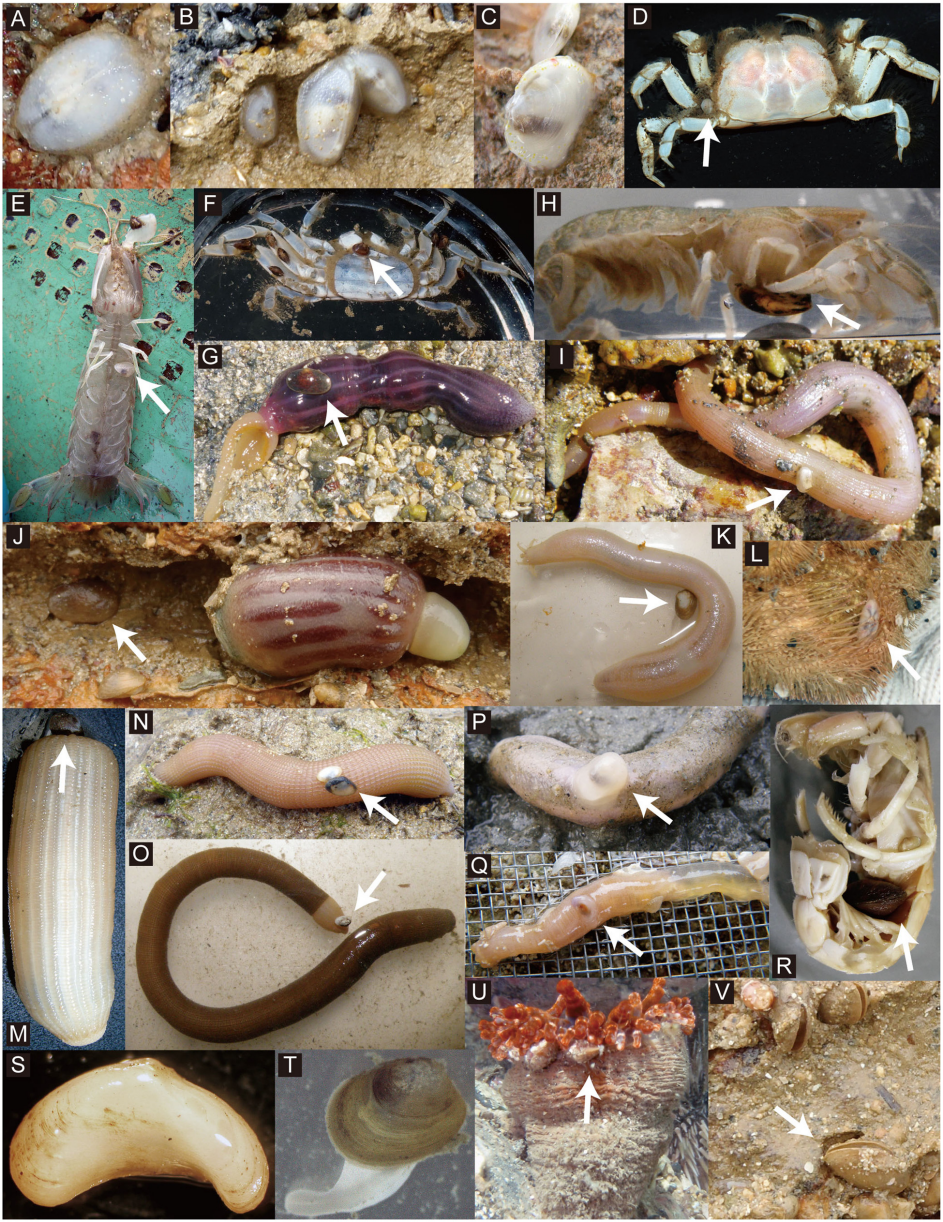
**Various galeommatoidean bivalves, including free-living species (A, C, T) and species symbiotic with their host invertebrates (B, D, E–S, U, V).** (**A**) *Galeomma* sp. attached to the undersurface of a rock; (**B**) *Ephippodonta gigas* living in the burrow of the thalassinidean shrimp *Callianidea typa*; (**C**) *Scintilla* aff. *hydatina* attached to the undersurface of a rock; (**D**) *Arthritica japonica* attached to the intertidal crab *Xenophthalmus pinnotheroides*; (**E**) *Pseudopythina subsinuata* attached to the abdomen of the mantis shrimp *Oratosquilla oratoria*; (**F**) *Pseudopythina macrophthalmensis* attached to the intertidal crab *Macrophthalmus* sp.; (**G**) *Pseudopythina ochetostomae* with its echiuran host *Listriolobus sorbillans*; (**H**) *Peregrinamor ohshimai* attached to the abdomen of the thalassinidean shrimp *Upogebia major*; (**I**) *Pseudopythina* aff. *nodosa* attached to the peanut worm *Siphonosoma cumanense*; (**J**) *Byssobornia yamakawai* living in the burrow of the spoon worm *Ochetostoma erythrogrammon*; (**K**) *Pseudopythina* aff. *ariake* with its holothurian host *Patinapta ooplax*; (**L**) *Scintillona stigmatica* attached to the heart urchin *Brissus latecarinatus*; (**M**) *Salpocola philippinensis* attached to the peanut worm *Sipunculus nudus*; (**N**) *Litigiella pacifica* attached to the peanut worm *S. nudus*; (**O**) *Nipponomysella subtruncata* attached to the peanut worm *S. cumanense*; (**P**) *Devonia semperi* attached to the sea cucumber *Protankyra bidentata*; (**Q**) *Anisodevonia ohshimai* attached to the sea cucumber *P. bidentata*; (**R**) *Neaeromya rugifera* attached to the abdomen of the thalassinidean shrimp *Upogebia pugettensis*; (**S**) *Curvemysella paula* collected from an empty shell carried by the hermit crab *Spiropagurus spiriger*; (**T**) *Mysella* aff. *bidentata* living in sand; (**U**) *Nipponomontacuta actinariophila* attached to the sea anemone *Telmatactis* sp.; (**V**) *Basterotia* sp. living in the burrow of the spoon worm *O. erythrogrammon*.

We collected sequence data for 18S rRNA, 28S rRNA, H3 and COI genes in 33 galeommatoidean species belonging to 25 genera and two families, five putative galeommatoidean species belonging to *Peregrinamor* and *Basterotia*, and eight outgroup species for the molecular phylogenetic analysis (Table 1). To examine whether *Peregrinamor* and *Basterotia* do belong to Galeommatoidea, we selected eight outgroup species representing a range of lineages within Heterodonta, to which Galeommatoidea belongs. We also included three non-heterodont species representing each of three non-heterodont orders to root the entire heterodont tree. All sequences were newly obtained in this study except for the sequences of three non-heterodont species (Additional file 1). Ingroup specimens included 13 free-living species and 25 symbiotic species associated with the crustacean, echinoid, holothurian, cnidarian, sipunculan, and echiuran animal groups (Figure 1; Table 1).

**Table 1:** Sampling information for the specimens used in this study

**Superfamily**	**Family**	**Species**	**Life style**	**Host species**	**Host taxon**	**Host utilization/ habitat**
Galeommatoidea	Galeommatidae	*Divariscintilla toyohiwakensis*	Symbiotic	*Acanthosquilla acanthocarpus*	Mantis shrimp (Arthropoda)	Inside the host burrow
		*Ephippodonta gigas*	Symbiotic	*Callianidea typa*	Thalassinidean shrimp (Arthropoda)	Inside the host burrow
		*Galeomma* sp.	Free-living	*-*	-	On the undersurface of rocks
		*Pseudogaleomma* sp.	Free-living	*-*	-	On the undersurface of rocks
		*Scintilla rosea*	Free-living	*-*	-	On the undersurface of rocks
		*Scintilla* aff. *hydatina*	Free-living	*-*	-	On the undersurface of rocks
		*Scintilla* sp.1	Free-living	*-*	-	On the undersurface of rocks
		*Scintilla* sp.2	Free-living	*-*	-	On the undersurface of rocks
		*Scintillona stigmatica*	Symbiotic	*Brissus latecarinatus*	Heart urchin (Echinodermata)	On the host body
	Lasaeidae	*Anisodevonia ohshimai*	Symbiotic	*Patinapta ooplax*	Sea cucumber (Echinodermata)	On the host body
		*Arthritica japonica*	Symbiotic	*Xenophthalmus pinnotheroides*	Intertidal crab (Arthropoda)	On the host body
		*Byssobornia yamakwai*	Symbiotic	*Ochetostoma erythrogrammon*	Spoon worm (Echiura)	Inside the host burrow
		*Curvemysella paula*	Symbiotic	*Spiropagurus spiriger*	Hermit crab (Arthropoda)	Inside the shell carried by the host
		*Devonia semperi*	Symbiotic	*Protankyra bidentata*	Sea cucumber (Echinodermata)	On the host body
		*Entovalva lessonothuriae*	Symbiotic	*Holothuria* (*Lessonothuria*) *pardalis*	Sea cucumber (Echinodermata)	Inside the host esophagus
		*Kellia porculus*	Free-living	*-*	-	In the crevice of dead corals
		*Lasaea undulata*	Free-living	*-*	-	In the crevice of rocks
		*Litigiella pacifica*	Symbiotic	*Sipunculus nudus*	Peanut worm (Sipuncula)	On the host body
		*Melliteryx puncticulata*	Free-living	*-*	-	On the undersurface of rocks
		*Montacutona* sp*.*	Symbiotic	*Cerianthus filiformis*	Sea anemone (Cnidaria)	On the host body
		*Mysella* aff*. bidentata*	Free-living	*-*	-	In sand sediment
		*Neaeromya rugifera*	Symbiotic	*Upogebia pugettensis*	Thalassinidean shrimp (Arthropoda)	On the host body
		*Nipponomontacuta actinariophila*	Symbiotic	*Telmatactis* sp.	Sea anemone (Cnidaria)	On the host body
		*Nipponomysella oblongata*	Free-living	*-*	-	In sand sediment
		*Nipponomysella subtruncata*	Symbiotic	*Siphonosoma cumanense*	Peanut worm (Sipuncula)	On the host body
		*Paraborniola matsumotoi*	Free-living	*-*	-	On the undersurface of rocks
		*Peregrinamor gastrochaenans*	Symbiotic	*Upogebia carinicauda*	Thalassinidean shrimp (Arthropoda)	On the host body
		*Peregrinamor ohshimai*	Symbiotic	*Upogebia major*	Thalassinidean shrimp (Arthropoda)	On the host body
		*Pseudopythina ochetostomae*	Symbiotic	*Listriolobus sorbillans*	Spoon worm (Echiura)	Inside the host burrow
		*Pseudopythina macrophthalmensis*	Symbiotic	*Macrophthalmus* sp*.*	Intertidal crab (Arthropoda)	On the host body
		*Pseudopythina subsinuata*	Symbiotic	*Oratosquilla oratoria*	Mantis shrimp (Arthropoda)	On the host body
		*Pseudopythina* aff. *ariake*	Symbiotic	*Protankyra bidentata*	Sea cucumber (Echinodermata)	Inside the host burrow
		*Pseudopythina* aff. *nodosa*	Symbiotic	*Siphonosoma cumanense*	Peanut worm (Sipuncula)	On the host body
		*Pythina deshayesiana*	Free-living	*-*	-	On the undersurface of rocks
		*Salpocola philippinensis*	Symbiotic	*Sipunculus nudus*	Peanut worm (Sipuncula)	On the host body
Cyamioidea	Basterotiidae	*Basterotia carinata*	Symbiotic	*Ochetostoma erythrogrammon*	Spoon worm (Echiura)	Inside the host burrow
		*Basterotia gouldi*	Symbiotic	*Ikedosoma gogoshimense*	Spoon worm (Echiura)	Inside the host burrow
		*Basterotia* sp.	Symbiotic	*Ochetostoma erythrogrammon*	Spoon worm (Echiura)	Inside the host burrow
Outgroups	Solecurtidae	*Azorinus minutus*	Free-living	*-*	-	-
	Gastrochaenidae	*Gastrochaena cuneiformis*	Free-living	*-*	-	-
	Veneridae	*Irus mitis*	Free-living	*-*	-	-
	Mactridae	*Meropesta nicobarica*	Free-living	*-*	-	-
	Solenidae	*Solen strictus*	Free-living	*-*	-	-
	Solemyidae	*Solemya velum*	Free-living	*-*	-	-
	Nuculanidae	*Nuculana pella*	Free-living	*-*	-	-
	Neotrigoniidae	*Neotrigonia margaritacea*	Free-living	*-*	-	-

Taxonomic classification follows Bieler *et al*. (2010).

**Figure 2:**
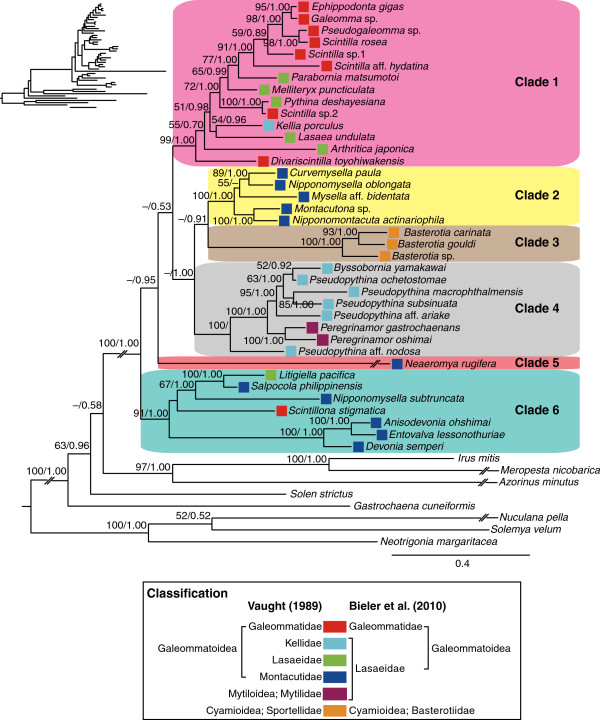
**Maximum-likelihood tree of Galeommatoidea based on the combined dataset of 18S, 28S, H3 and COI genes.** Especially long branches are broken down to fit the page; unmodified phylogeny with correct branch lengths is shown in upper left. Numbers above branches indicate maximum-likelihood bootstrap support values followed by Bayesian posterior probabilities. The color of boxes to the left of species names indicates the family to which the species belongs, as defined by Vaught (1989) and Bieler *et al*. (2010).

Phylogenetic analysis based on the combined data (18S + 28S + COI + H3; Additional file 2) suggests that the currently circumscribed Galeommatoidea is not monophyletic and includes *Peregrinamor* and *Basterotia* (Figure 2). The expanded Galeommatoidea with the above two genera was strongly supported as a monophyletic group and consists of at least six major clades, most of which are supported by high clade support (Figure 2). However, the relationships among these clades received low support and remained obscure (Figure 2). Notably, the branches leading to *Neaeromya* and the three species of *Basterotia* are especially long, which likely further complicates the recovery of branching order among these major lineages. Thus, while we consider that the monophyly of each of the six major clades is supported, the relationships among these clades must be viewed with caution. The seemingly high Bayesian posterior probability values at higher nodes should also be taken with caution because Bayesian posterior probabilities often produce overcredible results when compared with bootstrap analyses [21-23]. 

### Taxonomic classification of the extant Galeommatoidea

Our molecular phylogenetic analysis suggests that Galeommatoidea includes *Basterotia* and *Peregrinamor*, which were previously assigned to Cyamioidea and Mytiloidea, respectively [18]. *Basterotia* bivalves differ from other galeommatoideans in having the inhalant siphon located posteriorly [20], while *Peregrinamor* bivalves were included in Mytilidae on the sole basis of superficial resemblance of their shells to those of true Mytilidae [19]. However, our result firmly places these enigmatic genera within Galeommatoidea (Figure 2). Our analysis further reveals that the expanded Galeommatoidea is composed of six major clades. Below we detail major discrepancies between the current classification of Galeommatoidae and phylogenetic relationships obtained in this study. Detailed accounts of the morphology and ecology of each of the major clades are provided in Additional file 3.

Recent provisional classification suggests that Galeommatoidea comprises two families, Galeommatidae and Lasaeidae [17]. However, our results did not support this taxonomic circumscription (Figure 2) and suggested that Galeommatidae and Lasaeidae are polyphyletic (Figure 2). Except for *Scintillona stigmatica*, the members of the family Galeommatidae were grouped into Clade 1, which also included several lasaeid species (Figure 2), whereas the members of Lasaeidae were divided into six major clades (Figure 2). Lasaeidae was previously divided into several families, namely Lasaeidae, Kellidae, and Montacutidae [18]. However, these previously proposed families were also each non-monophyletic and included genera that were separated into different major clades (Figure 2). These results suggest that the previous family-level classification of Galeommatoidea does not correspond to any of the well-supported clades recovered in our molecular phylogenetic analysis. Furthermore, according to our results, some of the previous genus-level classifications were also not supported. For example, *Scintilla* and *Nipponomysella* are polyphyletic (Figure 2)*.* Yet, the discrepancies between the traditional classification and the results of our molecular phylogenetic analysis are not unexpected, considering that many members of Galeommatoidea have reduced or highly specialized morphologies associated with symbiotic life, which potentially obscures the historical information contained in their morphology.

### Evolutionary pattern of symbiotic lifestyles in Galeommatoidea

**Figure 3:**
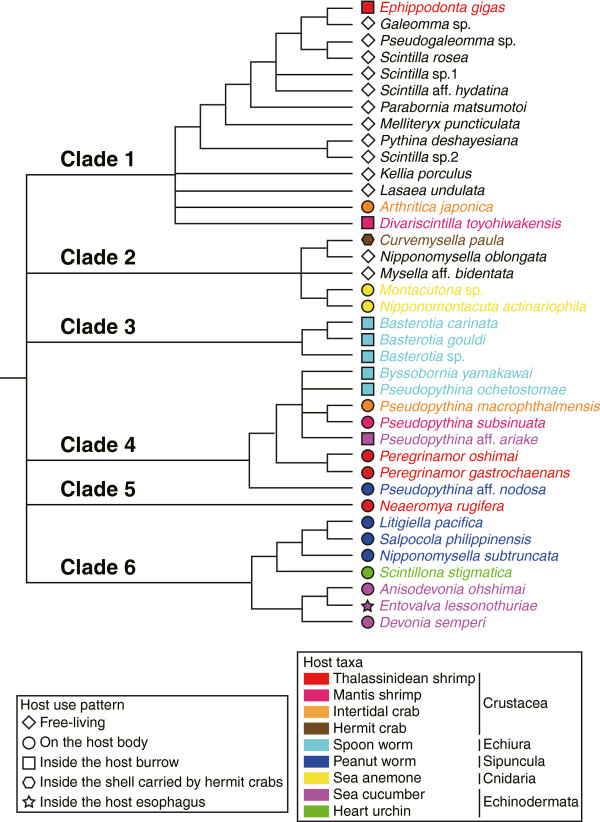
**The modified maximum-likelihood tree of Galeommatoidea based on the combined dataset of 18S, 28S, H3 and COI genes.** Branches supported by ≪65 % maximum-likelihood bootstrap support or ≪90 % Bayesian posterior probability values are collapsed. The color and shape of each symbol indicates the host taxon and host use pattern, respectively.

To investigate the evolutionary pattern of symbiotic lifestyles in Galeommatoidea, we mapped information on the lifestyle (symbiotic or free-living), host taxon, and host association mode of each galeommatoidean species onto the phylogenetic tree (Figure 3). The host organisms we recorded in this study were consistent with those reported in previous studies (Additional file 4), except for *Pseudopythina* aff. *nodosa* and *Nipponomontacuta actinariophila.* The host organism of the latter species was misidentified in the original paper [24]; we obtained *N. actinariophila* from the sea anemone *Telmatactis* sp., which conforms to more recent records. 

Clades 1 and 2 each comprise both free-living and symbiotic species, whereas Clades 3–6 consist of only symbiotic species (Figure 3). The approximately unbiased (AU) test [25] rejected the hypothesis that free-living or symbiotic species are monophyletic in Galeommatoidea (*P* ≪ 0.0001, Additional file 5b), suggesting that transitions between free-living and symbiotic lifestyles occurred multiple times in this bivalve group. Associations with crustaceans, holothurians, sipunculans, and echiurans are each found in multiple major clades (Figure 3). The AU tests also rejected the hypothesis that species with same host taxa are monophyletic (all *P* values ≪ 0.0001, Additional file 5c–f), suggesting that host specialization to these animal groups likely occurred multiple times independently in Galeommatoidea. In addition, species having very similar modes of physical attachment to the hosts were seen in different major clades (Figure 3). For example, the bivalves attached to the abdomen of thalassinidean shrimps were divided into Clades 4 and 5 (Figure 3), and those attached to the body of intertidal crabs were also divided into Clades 1 and 4. These results were also supported by AU tests (all *P* values ≪ 0.0001, Additional file 5g–h). The hypothesis that the species symbiotic with crustaceans in Clade 1 are monophyletic was also rejected (*P* ≪ 0.0001, Additional file 5i), whereas this was not the case in Clade 4 (*P* = 0.065; Additional file 5j). These results suggest that the gain and/or loss of symbiotic association with crustaceans likely occurred multiple times at least in Clade 1. Although the AU test does not account for uncertainty in phylogenetic estimation, all the strongly rejected tests concern species belonging to different major clades, each with high statistical support, indicating that our conclusions are unlikely affected by phylogenetic uncertainty.

Clades 2, 4 and 6 each includes species utilizing hosts from different animal phyla (Figure 3), suggesting that interphylum host switches occurred repeatedly even within these major clades. In contrast, Clades 1, 3 and 5 each includes species symbiotic with a single phylum (Figure 3).

The ecological modes of host associations are diverse in the Galeommatoidea [11-13,20]. We categorized host association modes into four types: ectosymbiotic on the host body, living inside the host burrow, endosymbiotic inside the host esophagus, and living inside the shells carried by host hermit crabs (Table 1). The former two are the predominant host use patterns in Galeommatoidea (Table 1), whereas the latter two are each found in only a single species. According to the phylogenetic tree (Figure 3), the ectosymbiotic species belonged to five major clades, whereas burrow associates belonged to four major clades. The AU tests rejected the hypothesis that ectosymbiotic species or burrow associates are monophyletic (both *P* values ≪ 0.0001, Additional file 5k, l), suggesting that there have been repeated shifts between these two modes of host utilization in this bivalve superfamily.

### Parallel evolution of host associations in Galeommatoidea

Symbiotic associations with crustaceans, holothurians, sipunculans, and echiurans occurred multiple times independently within Galeommatoidea (Figure 3). Below, we compare the groups that independently established symbiotic associations with the same animal groups to better understand the evolution of host associations in Galeommatoidea.

#### Crustacea

Symbiotic associations with crustaceans were found in Clades 1, 2, 4 and 5 (Figure 3). Clade 1 includes *Ephippodonta gigas* that lives in the burrows of thalassinidean shrimps [15] (Figure 1B), *Divariscintilla toyohiwakensis* that lives in the burrows of mantis shrimps [26], and *Arthritica japonica* that attaches directly onto the body surface of intertidal crabs [27] (Figure 1D). Clade 2 includes *Curvemysella paula* that lives inside shells carried by hermit crabs [12,28] (Figure 1S). Clade 4 includes two *Pseudopythina* and two *Peregrinamor* species that attach directly onto the body surface of upogebid shrimps, mantis shrimps, or intertidal crabs [12,19] (Figure 1E, F, H). Clade 5 includes *Neaeromya rugifera* that attaches onto the abdomen of upogebid shrimps [29,30] (Figure 1R).

Interestingly, *Peregrinamor* from Clade 4 and *Neaeromya* from Clade 5 both attach to the abdomen of upogebid shrimps [19,29-31] (Figure 1H, R). The former is distributed in East Asia, and the latter is distributed in North America; thus, similar symbiotic associations with upogebid shrimps likely originated independently in separate geographic locations. *Peregrinamor* bivalves have evolved remarkable behavioral adaptations to maintain the correct location on the individual *Upogebia* host through ecdysis events [32]. However, it is unknown whether *N. rugifera* shows a similar behavioral adaptation.

Additionally, *A*. *japonica* from Clade 1 and *Pseudopythina macrophthalmensis* from Clade 4 both attach onto the body surface of intertidal crabs [12,27] (Figure 1D, F). The former is associated with *Xenophthalmus pinnotheroides* in the Seto Inland Sea, Japan [27], and the latter with various species of *Macrophthalmus* in subtropical intertidal flats of East Asia [12]. Each of these bivalve species has a smaller body than other closely related species within the same clade. Although its adaptive significance is unknown, such a reduction in size may be related to the ectosymbiotic association with host intertidal crabs.

#### Holothuroidea

Symbiotic associations with holothurians were found in Clades 4 and 6. Clade 4 includes *Pseudopythina* aff. *ariake*, which attaches to the burrow walls of the host holothurian [12] (Figure 1K), whereas Clade 6 includes *Devonia semperi* and *Anisodevonia ohshimai*, which attach to the body surface of the host holothurian [12,33] (Figure 1P, Q), and *Entovalva lessonothuriae*, which lives inside the esophagus of the host holothurian [33]. In the Seto Inland Sea, Japan, *Devonia semperi* and *P.* aff. *ariake* are both associated with *Protankyra bidentata* and often co-occur sympatrically in the same *P. bidentata* burrow (Goto, Ishikawa & Hamamura, unpublished data).

#### Echiura

Symbiotic associations with echiurans were found in Clades 3 and 4. Clade 3 includes three species of *Basterotia* (Figure 1V), whereas Clade 4 includes *Byssobornia yamakawai* and *Pseudopythina ochetostomae* (Figure 1G, J). All of these live in the echiuran burrow but in markedly different ways [12,20,34]. The former three species embed their body into the burrow wall, gaping their posterior aperture into the burrow lumen [20,34] (Figure 1V), whereas the latter two live in the narrow space between the host body and burrow wall [12,34] (Figure 1G, J). *Basterotia* sp. (Clade 3) and *Byssobornia yamakawai* (Clade 4) often co-occur in the same *Ochetostoma erythrogrammon* burrow in the Ryukyu Islands, Japan [34].

#### Sipuncula

Symbiotic associations with sipunculans were found in Clades 4 and 6. Clade 4 includes *Pseudopythina* aff. *nodosa* that attaches directly to the body surface of host sipunculans [12] (Figure 1I), whereas Clade 6 includes *Salpocola philippinensis*, *Nipponomysella subtruncata* and *Litigiella pacifica*, all of which also attach to the body surface of host sipunculans [12,35-37] (Figure 1M–O)*. Pseudopythina* aff. *nodosa* (Clade 4) and *N. subtruncata* (Clade 6) both attach to the body surface of the same sipunculan species, *Siphonosoma cumanense* (Figure 1I, O).

### Frequent interphylum host switching in Galeommatoidea

In most parasitic organisms, phylogenetic tests demonstrate that host associations are generally conserved across the phylogeny and that switches between distantly related hosts are relatively infrequent [38-42]. In contrast, we found multiple possible instances of host switching between different phyla by galeommatoidean bivalves (Figure 3), raising the question as to what factors promote such a pattern of host switching in this superfamily.

The predominant mode of host switching in parasitic organisms is that between closely related hosts [38-42]. This is because parasites are under strong pressure to overcome host defenses, and switches to phylogenetically distant hosts likely require entirely new sets of anti-defense adaptations. Parasitic life also involves exploitation of nutrients from host organisms. Thus, parasites undergoing host shift must adapt to drastic changes in the nutrients they derive from the new host. These and other ecological conditions probably constrain the host range of parasitic organisms and make it difficult to switch between distantly related hosts (e.g., across phyla). In contrast, the symbiotic galeommatoideans are predominantly filter feeders and do not depend directly on their hosts for nutrients, although they indirectly benefit from the water currents created by the host [12]. Furthermore, while symbiotic galeommatoideans benefit greatly from being associated with their host organisms, they usually cause little or no harm to their hosts. In fact, there are no known host defense mechanisms that specifically target these symbionts, although the bivalves themselves have evolved adaptations to stay cryptic on the host body or inside the host burrow. Thus, the commensal lifestyle of these bivalves probably allows them to switch hosts without a need to adapt to host physiology or evolve other defense mechanisms. Overall, these ecological attributes of galeommatoidean bivalves probably made them frequent colonizers of various marine invertebrate hosts.

### Remaining issues and directions for future research

Our results suggest that host switching played an important role in the diversification of Galeommatoidea. However, this superfamily also includes a large number of free-living species [15], whose diversification obviously did not occur by host switching. In addition, several well-supported clades of symbiotic bivalves include species that share closely related hosts, suggesting that mechanisms other than host switching have also been important in the evolution of these bivalves. For example, the closely-related *Anisodevonia ohshimai*, *Entovalva lessonothuriae* and *Devonia semperi* in Clade 6 all utilize sea cucumbers as hosts, and thus, cospeciation (i.e., parallel speciation of hosts and parasites) and/or shift in the mode of host utilization may have been the major driver of speciation. Similarly, *Basterotia* species (Calde 3) commonly share the same echiura host species [34], indicating that host switches are unlikely to have occurred in this genus. Clarifying alternative mechanisms of speciation other than host switching is therefore needed to gain a full understanding of the diversification history of Galeommatoidea.

Overall, our molecular phylogenetic analysis has greatly progressed our understanding of the phylogenetic relationships within Galeommatoidea. However, the relationships among the major clades, as well as those within each clade, are still poorly resolved. Inclusion of more species in the analysis and better marker choice are probably needed to obtain higher resolved molecular phylogenies of this bivalve superfamily. Such well-resolved phylogenies can then be used to further test questions such as (1) the direction and frequency of transitions between free-living and symbiotic lifestyles in Galeommatoidea, (2) the relative importance of host switching as compared to other speciation mechanisms, as discussed above, and (3) patterns of morphological, behavioral and physiological specialization/reduction associated with symbiosis with diverse invertebrate hosts.

## Conclusions

The present analysis of nuclear and mitochondrial DNA sequence data is the first comprehensive molecular phylogenetic analysis of the bivalve superfamily Galeommatoidea. The phylogenetic tree suggested the inclusion of *Peregrinamor* and *Basterotia* within Galeommatoidea and recovered six major clades within the expanded Galeommatoidea. Symbiotic associations with crustaceans, echiurans, sipunculans, and holothurians were found in multiple major clades, suggesting that host specialization to these animal groups occurred repeatedly in this superfamily. Furthermore, within the same major clades, the associated hosts were often divergent at the phylum level, suggesting that host switching between different phyla occurred repeatedly in Galeommatoidea. The groupings based on molecular phylogeny did not correspond with the extant familial classification; Galeommatidae and Lasaeidae were found to be polyphyletic. Therefore, taxonomic revision of this bivalve superfamily is needed.

## Methods

### Sampling

We collected 38 specimens from 33 galeommatoidean and five putative galeommatoidean species belonging to 27 genera and three families. For outgroups, we sampled five species belonging to five non-galeommatoidean families within Heterodonta (Table 1). All specimens were collected in southwestern Japan with the exception of *Neaeromya rugifera*, which was collected in Oregon, USA (see Additional file 1). We also included the sequence data of three non-heterodont outgroup species that were available in GenBank (Additional file 1) to root the entire heterodont phylogeny.

### Molecular methods

Total DNA was isolated following a previously described method [43]. Adductor muscle tissue was homogenized in 800 μl lysis buffer and incubated at 55°C overnight, after which 80 μl saturated potassium chloride was added to the lysate. This solution was incubated for 5 min on ice and then centrifuged for 10 min. The supernatant (700 μl) was transferred to a new tube, cleaned once with a phenol/chloroform solution, and precipitated with an equal volume of 2-propanol. The DNA pellet was rinsed with 70% ethanol, vacuum-dried, and dissolved in 100 μl TE buffer.

We sequenced the fragments of the nuclear 18S and 28S ribosomal RNA (rRNA), H3 and the mitochondrial COI genes. Polymerase chain reactions (PCRs) were used to amplify ~1700 bp of 18S rRNA, ~1000 bp of 28S rRNA, ~350 bp of H3 and ~700 bp of COI. Amplifications were performed in 20 μl mixtures consisting of 0.4 μl of forward and reverse primers (primer sequences are provided in Additional file 6), 1.6 μl of dNTP, 2.0 μl of ExTaq buffer, 0.1 μl of ExTaq polymerase (TaKaRa, Otsu, Japan), and 15.1 μl of distilled water. Thermal cycling was performed with an initial denaturation for 3 min at 94°C, followed by 30 cycles of 30 s at 94°C, 30 s at a gene-specific annealing temperature (Additional file 6), and 2 min at 72°C, with a final 3 min extension at 72°C. The sequencing reaction was performed using the PCR primers and internal primers (Additional file 6) and the BigDye Terminator Cycle Sequencing Ready Reaction Kit (Applied Biosystems, Foster City, CA) and electrophoresed on an ABI 3130 sequencer (Applied Biosystems). The obtained sequences have been deposited in the DDBJ/EMBL/GenBank databases with accession numbers AB714745–AB714907 (Additional file 1).

### Phylogenetic analysis

Sequences of the 18S, 28S, H3 and COI genes were aligned using Muscle [44] as implemented in the software Seaview [45,46] under default settings. The alignments of H3 and COI sequences did not require insertion of gaps and was therefore unambiguous. We used the software Gblocks v0.91b [47-49] to delimit ambiguously aligned regions in the 18S and 28S alignments (Additional file 7), and the following phylogenetic analyses were conducted with and without alignment-ambiguous regions. The full 18S and 28S alignments contained 474 and 282 variable sites, respectively, and 453 and 249 variable sites when alignment-ambiguous regions were excluded. The H3 and COI alignments contained 87 and 297 variable sites, respectively, indicating that despite their short sequence lengths, they contain comparable amount of information as the 18S and 28S partitions. Because the initial phylogenetic analyses of individual genes did not produce essentially different results (Additional file 8), we focused our analyses on the combined four-gene data set, which will be described below.

Phylogenetic trees were obtained by the maximum-likelihood (ML) and Bayesian methods. For the ML analysis, model selection and tree search were conducted using the TreeFinder program [50,51]. The robustness of the ML tree was validated by bootstrap analysis with 1000 replications using the same program.

Bayesian analyses were performed using MrBayes 3.1.2 [52] with substitution models chosen using MrModeltest 2.3 [53]. In the combined data set, substitution parameters were estimated separately for each gene using the ‘unlink’ command. Two independent runs of Metropolis-coupled Markov chain Monte Carlo were performed simultaneously, sampling trees every 100 generations and calculating the average standard deviation of split frequencies every 1000 generations. Using the ‘stoprule’ option, analyses were continued until the average standard deviation of split frequencies dropped below 0.01, at which point the two chains were considered to have achieved convergence. Because the average standard deviation of split frequencies was calculated based on the last 75% of the samples, we discarded the initial 25% of the sampled trees as burn-in. We confirmed that analyses reached stationarity well before the burn-in period by plotting the ln-likelihood of the sampled trees against generation time.

### Mapping of host taxon and mode of host utilization

To evaluate the evolutionary history of symbiotic life in Galeommatoidea, we mapped information on the lifestyle (symbiotic or free-living), host taxon, and mode of host utilization of each galeommatoidean species onto the phylogenetic tree. Host information was based on our sampling data. We checked our sampling data against previously available information on host association for each bivalve species (see Additional file 4) to confirm the validity of our observations of their lifestyle and host taxon.

### Approximately unbiased (AU) test

We tested the hypothesis of monophyly of species sharing the same lifestyle (free-living and symbiotic), modes of host utilization, or host taxa using the AU test [25]. The analyses were done using the combined four-gene data set. The alternative trees were obtained by ML heuristic search under the topological constraint (Additional file 5), and the AU test was conducted based on 1,000,000 replications using Treefinder [50,51].

## Competing interests

The authors declared that they have no competing interests.

## Authors’ contributions

RG designed the study, carried out the field survey, performed the molecular sequencing and the phylogenetic analysis, and drafted the manuscript. AK performed the phylogenetic analysis and drafted the manuscript. HI and YH carried out the field survey and collected the specimens. MK participated in the design of the study and drafted the manuscript. All authors read and approved the final manuscript.

## Supplementary Material

Additional file 1 Sampling information of the specimens used in this study. Click here for file

Additional file 2 **Combined molecular data set.** The data provided includes an alignment of the four concatenated molecular data partitions (18S, 28S, H3 and COI). Click here for file

Additional file 3 Morphological and ecological accounts for each clade within Galeommatoidea. Click here for file

Additional file 4 Host information of each galeommatoidean species sampled. Click here for file

Additional file 5 The results of approximately unbiased (AU) tests. Click here for file

Additional file 6 Information on primers and PCR conditions used in this study. Click here for file

Additional file 7 Information on sequence alignment and models of sequence evolution for the maximum likelihood analysis. Click here for file

Additional file 8 **Maximum likelihood tree of Galeommatoidea based on each partition.** Numbers above branches indicate maximum-likelihood bootstrap support values followed by Bayesian posterior probabilities. Especially long branches are broken down to fit the page. Click here for file
