# Supplementary material for: Molecular phylogeny of the bivalve superfamily Galeommatoidea (Heterodonta, Veneroida) reveals dynamic evolution of symbiotic lifestyle and interphylum host switching
Source: BMC Evol Biol. 2012 Sep 6;12:172. doi: 10.1186/1471-2148-12-172 (PMC3532221; doi:10.1186/1471-2148-12-172)
Supplement: Additional file 2 — Combined molecular data set. The data provided includes an alignment of the four concatenated molecular data partitions (18S, 28S, H3 and COI). [file 1471-2148-12-172-S2.pdf]

Additional File 2
Combined molecular data set

DATA PARTITIONS

1-1735 18S
1736-2662 28S
2663-2990 H3
2991-3649 COI

>Divariscintilla_toyohiwakensis
TAGTCATATG CTTGTCTCAA AGATTAAGCC ATGCATGTCT AAGTACACGC CAGAATAATG
GTGAAACTGC GAATGGCTCA TTAAATCAGT TATGGTTCCT TAGATCGTAC AATCCTACTT
GGATAACCGT GGCAATTCTA GAGCTAATAC ATGCGTCAAA GCTCCGACCT TCGGGGAAGA
GCGCTTTTGT TAGCAAAACC AATCCGGTCG GTTGTTGACT CTGAACAACT TTGTGCTGAT
CGCACGGCCT AGCGCCGGCG ATGTATCTTT CGAATGTCTG CCCTATCAAC TGTCGATGGT
ACGTGCTATG CCTACCATGG TTGTAACGGG TAACGGGGAA TCAGGGTTCG ATTCCGGAGA
GGGAGCATGA GAAACGGCTA CCACATCCAA GGAAGGCAGC AGGCGCGCAA ATTACCCAAT
CCCGACACGG GGAGGTAGTG ACGAAAAATA ACAATACGGG ACTCTTTCGA GGCCCCGTAA
TTGGAATGAG TACACTTTAA ATCCTTTAAC GAGGATCCAT TGGAGGGCAA GTCTGGTGCC
AGCAGCCGCG GTAATTCCAG CTCCAATAGC GTATATTAAA GTTGTTGCAG TTAAAAAGCT
CGTAGTTGGA TCTCGGGTGC AGGCTTGCGG TCCGCCTC-C GGCGGCTGCT CGTCCTGGCA
GCCTAGCTGC GGTATCCCTT GGTGCTCTTG ACTGAGTGTC GGTGGCCGGA ACGTTTACTT
TGATGAAATT AGAGTGTTCA AAGCGCGGGC GTTTGCCCGC ATAATGGTGC ATGGAATGAT
AGAATAGGAC CTCGGTTCTA TTTTGTTGGT TTTCGGAGAG GTAATGATTG AGAGGGACAG
ACGGGGGCAT TCGTATTGCG GCGTTAGAGG TGAAATTCTT GGATCGCCGC AAGACGAACT
ACAGCGAAAG CATTTGCCAA GAATGTTTTC CTTAATCAAG AACGAAAGTC AGAGGCTCGA
AGACGATCAG ATACCGTCGT AGTTCTGACC ATAAACCATG CCGACTGGCA ATCCGCCGGA
GTTACTACAA TGACTCGGCG AGCAGCCCCC GGGAAACCAA AGTTTCTGGG TTCCGGGGGG
AGTATGGTTG CAAAGCTGAA ACTTAAAGGA ATTGACGGAA GGGCACCACC AGGAGTGGAG
CCTGTGGCTT AATTTGACTC AACACGGGAA ACCTCACCCG GCCCGGACAC CGTTAGGATT
GACAGATTGA GAGCTCTTTC TTGATTCGGT GGGTGGTGGT GCATGGCCGT TCTTAGTTGG
TGGAGCGATT TGTCTGGTTA ATTCCGATAA CGAACGAGAC TCTAGCCTAC TAAATAGTTC
GAGGATATAT AACCTCGCAA CTTCTTAGAG GGACAGGTGG CGTATAGCCA CACGAGATTG
AGCAATAACA GGTCTGTGAT GCCCTTAGAT GTCCGGGGCC GCACACGCGC TACATTGAAT
GGATCAACGT GCGTCTAGCC TTGCCCGAAA GGGCTGGGAA ACCCGTTGAA ACCCATTCGT
GATAGGGACT GGGGCTTGCA ATTATTTCCC ATGAACGAGG AATTCCTAGT AAGCGCGAGT
CATCAGCTCG CGTTGATTGC GTCCCTGCCC TTTGTACACA CCGCCCGTCG CTACTACCGA
TCGCTCCAGT TAATGAACGC CTCGGATTGG TTAAGCGGGT TTCGGCCTGC TCGCGTGCCG
AGAAGACGTG TAAATTATCT GGAGTAGAGG GAGTAAAAGT CGTAACAAGG TATCCATTAA
TAAGCGGAGG AAAAGAGACT AACTAGGATT CCCCTAGTAA CGGCGAGTGA AGCGGGAAGA
GCCCAGCACC GAATCCCCCG GCATCTGGCT GCCGACGGGA CATGTGGTGT TAGCGGGAGC
TTTTTGTCGG CGCGCTCCGG CGCCAAAGTC CACCTGATCG TGGCAACCCC TGGCGGGTGA
GAGGCCCGTT CCGGTGTCGG ACGCGTCGGA CTCTCTCCAG GAGTCGGGTT GTTTGAGAAT
GCAGCCCAAA GTGGGTGGTA AACTCCATCT AAGGCTAAAT ACGGACACGA GTCCGATAGA
GGACAAGTAC CGTGAGGGAA AGTTGAAAAG AACTTTGAAG AGAGAGTTCA AGAGTACGTG
AAACCGCATA GAGGCAAACG GGTGGATCCG CAAGGTGTCG GACCGGGGAA TTCAGCGGTG
CACTTTCTCC GACGAGAGCC ACGACCGGGT CTGCTGCTCG CGGTGCCCGA AGGTTTCCAG
GCGGCCTGGG AGCTTATAGC GGGCCGCACG CGAGTCTGTA TGTCGCGGGC CCGAGGACGC
GCCGCGCGCC CGGCTCCGGG TCGTCCTCGC GCGTTCAACC TTCT-GGGTA GTGTGCCGTA

ACCGCGCGCG GATCCGG-TG CCAGGGTCGG TGGCGAATCG GTCGGCGCTC CACCCGACCC
GTCTTGAAAC ACGGACCAAG GAGTCTAACA TGTGCGCGAG TCACGGGGAC CCGAATCCCG
AAGGCGCAAT GAAAGTGAAG GCCGCCCACG GCTGGCCGAG GCAGGATCCC GTCG-GGGCG
CACTGCCGGC CCGTCTCGAT AGCGGCGCGG TCGCGGCCTG CGTTCGTCTA CGAGGCGGAG
CAAGAGCGTA CACGTTGGTA CCCGAAAGAT GGTGAACTAT GCCTGAGCAG GACGAAGTCA
GAGGAAACTC TGATGGAGGT CCGCAGCGAT TCTAGAAAAT CCACTGGTGG GAAAGCCCCC
AGGAAACAGC TGGCCACCAA GGCCGCACGT AAGAGTGCGC CAGCCACCGG TGGGGTGAAG
AAACCCCACA GGTACAGGCC TGGCACCGTT GCCCTCCGTG AGATCAGGAG GTACCAGAAG
AGCACCGAGC TTCTCATCAG GAAGCTCCCG TTCCAGCGTC TGGTGCGCGA GATNNCCCAG
GACTTCAAGA CCGACCTGCG CTTCCAGAGC TCCGCCGTCA TGGCCCTGCA GGAGGCCAGC
GAGGCCTACC TGGTCGGGCT GTTTGAGGAC ACCAACTTGT GCGCCATCCA CGCCAAGCGT
TACTTTGTAT TTTTTATTTG GTATTTGGTC AGGGCTGGCT GGAACATCTC TGAGGGTTTT
AATTCGCTTA GAGCTATCTC GGCCAGGGGC CTTTCTAGGT GATGACCATC TTTATAATGT
CATTGTCACA GCTCATGCTT TTGTGATAAT TTTCTTTCTT GTGATGCCAA TAACTGTAGG
AGGTTTTGGT AATTGGTTAG TTCCCCTAAT ACTAACTTCT CCTGATATGG CATTTCCTCG
TATAAACAAT ATGAGGTTTT GGCTTCTCCC TCCTGCTTTG TTTTTACTTT TAAGCTCTGC
TTTCGTTGAA AGAGGTATTG GTACGGGTTG AACAGTTTAT CCTCCTTTAT CTGGAAATGT
AGCCCATAGA GGAGGGTCAG TGGATTATGG AATTTTTTCT CTCCATTTAG CAGGTGTATC
TTCTATTTTA GGTGCTATTA ATTTTTTGGC AAGTTCTATT AACATGCGGC CTGAGATGAT
AGAGCTTAAG CGTGTTACTC TCTTTGTCTG ATCAATTGCT TTGACGGCTT TTTTATTGGT
GGTGGCAATA CCTGTTTTAG CCGGGGCTAT TACTATGCTT TTAACTGATC GGAATTTTAA
TACTTCTTTT TTTGATCCGG CAGGTGGGGG TGATCCTATT CTATTTGTCC ATTTATTTT

>Ephippodonta_gigas
TAGTCATATG CTTGTCTCAA AGATTAAGCC ATGCATGTCT AAGTACACGC CACTGTAACG
GTGAAACTGC GAATGGCTCA TTAAATCAGT TATGGTTCCT TAGATCGTAC AATCCTACTT
GGATAACCGT GGCAATTCTA GAGCTAATAC ATGCGTCAAA GCTCCGACCT TCGGGGAAGA
GCGCTTTTGT TAGCAAGACC AATCCGGTCG GTTGTTGACT CTGAACAACT TTGTGCTGAT
CGCACGGCCT AGCGCCGGCG ATGTATCTTT CGAATGTCTG CCCTATCAAC TGTCGATGGT
ACGTGCTATG CCTACCATGG TTGTAACGGG TAACGGGGAA TCAGGGTTCG ATTCCGGAGA
GGGAGCATGA GAAACGGCTA CCACATCCAA GGAAGGCAGC AGGCGCGCAA ATTACCCAAT
CCCGACACGG GGAGGTAGTG ACGAAAAATA ACAATACGGG ACTCTTTCGA GGCCCCGTAA
TTGGAATGAG TACACTTTAA ATCCTTTAAC GAGGATCAAT TGGAGGGCAA GTCTGGTGCC
AGCAGCCGCG GTAATTCCAG CTCCAATAGC GTATATTAAA GTTGTTGCAG TTAAAAAGCT
CGTAGTTGGA TCTCGGGTGC AGGCTTGCGG TCCGCCTCGC GGCGGCTGCT CGTCCTGGCA
GCCTAGCTGC GGTATCCCTT GGTGCTCTTG ACTGAGTGTC GGTGGCCGGA ACGTTTACTT
TGATGAAATT AGAGTGTTCA AAGCGCGGGC GCTTGCCCGC ATAATGGTGC ATGGAATGAT
AGAATAGGAC CTCGGTTCTA TTTTGTTGGT TTTCGGAGAG GTAATGATTG AGAGGGACAG
ACGGGGGCAT TCGTATTGCG GCGTTAGAGG TGAAATTCTT GGATCGCCGC AAGACGGACT
ACAGCGAAAG CATTTGCCAA GAATGTTTTC CTTAATCAAG AACGAAAGTC AGAGGCTCGA
AGACGATCAG ATACCGTCGT AGTTCTGACC ATAAACCATG CCAACTGGCA ATCCGCCGGA
GTTACTACAA TGACTCGGCG AGCAGCCCCC GGGAAACCAA AGTTTCTGGG TTCCGGGGGG
AGTATGGTTG CAAAGCTGAA ACTTAAAGGA ATTGACGGAA GGGCACCACC AGGAGTGGAG
CCTGTGGCTT AATTTGACTC AACACGGGAA ACCTCACCCG GCCCGGACAC CGTTAGGATT
GACAGATTGA GAGCTCTTTC TTGATTCGGT GGGTGGTGGT GCATGGCCGT TCTTAGTTGG
TGGAGCGATT TGTCTGGTTA ATTCCGATAA CGAACGAGAC TCTAGCCTAC TAAATAGTTC
GAGGATATAT AACCTCGCAA CTTCTTAGAG GGACAGGTGG CGTATAGCCA CACGAGATTG
AGCAATAACA GGTCTGTGAT GCCCTTAGAT GTCCGGGGCC GCACACGCGC TACATTGAAT
GGATCAACGT GCGTCTAGCC TTGCCCGAAA GGGCTGGGAA ACCCGTTGAA ACCCATTCGT
GATAGGGACT GGGGCTTGCA ATTATTTCGC ATGAACGAGG AATTCCTAGT AAGCGCGAGT

CATCAGCTCG CACTGATTGC GTCCCTGCCC TTTGTACACA CCGCCCGTCG CTACTACCGA
TCGCTCCAGT TAATGAACGC CTCGGATTGG TTAAGCGGGT TTCGGCCTGC TCGCGTGCCG
AGAAGACGTG TAAATTATCT GGAGTAGAGG GAGTAAAAGT CGTAACAAGG TATCCATCAA
TAAGCGGAGG AAAAGAGACT AACTAGGATT CCCCTAGTAA CGGCGAGTGA AGCGGGAAGA
GCCCAGCACC GAATCCCCCG GCGTCTGGCC GCCGACGGGA CCTGTGGTGT TAGCGGGAGT
CTTTTGTCGG CGTGCTTCGG CACCAAAGTC CACCTGATCG TGGCAACCCC CGGCGGGTGA
GAGGCCCGTT CCGGTGCCGG ACGCGTCCGA CTCTCTCCAG GAGTCGGGTT GTTTGAGAAT
GCAGCCCAAA GTGGGTGGTA AACTCCACCT AAGGCTAAAT ACCGACACGA GTCCGATAGA
GGACAAGTAC CGTGAGGGAA AGTTGAAAAG AACTTTGAAG AGAGAGTTCA AGAGTACGTG
AAACCGCATA GAGTCAAACG GGTGGATCCG CAAGGTGTTG GACCGGGGAA TTCAGCGGTG
CACTTTCTCC GACGAGAGCC ACGACCGGGT CCGCTGCTCG AGGTGTGCGA AGGTTTCCAG
GCG-CCTGGG AGCTTACAGC GCGCCGTCCT CGAGTCTGTA CGTCGCGGGC CCGAGGACGC
GCCGTGCGCC CGGCACCGGG TCGTCTTCGT GCGTCCGACT GTCT-TGGCA GTGCGCCGCG
ACCGCGCGCG GTCCCGGCCG TGAGGGTCGG TGGCGAATCA GTCGGCACTC CACCCGACCC
GTCTTGAAAC ACGGACCAAG GAGTCTAACA TGTGCGCGAG TCATGGGGAC CCGAATCCCG
AAGGCGCAAT GAAAGTGAAG GCCGCCCTCG GCCGGCCGAG GCAGGATCCC GTCCCGGGCG
CACTGCCGGC CCGTCTCGAC AGCGGCGCGG TCGCGGCCTG CGTTCGTCTG CGAGGCGGAG
CAAGAGCGTA CACGTTGGTA CCCGAAAGAT GGTGAACTAT GCCTGAGCAG GACGAAGTCA
GAGGAAACTC TGATGGAGGT CCGCAGCGAT TCTAGAAAGT CCACTGGTGG GAAAGCCCCC
AGGAAACAGT TGGCCACCAA GGCCGCACGT AAGAGTGCAC CGGCCACCGG TGGTGTCAAG
AAGCCACACA GATACAGGCC CGGAACCGTG GCTCTCCGTG AGATCAGGAG ATACCAGAAG
AGCACCGAGC TTCTCATCCG AAAACTGCCA TTCCAGCGTC TCGTCCGCGA GATTGCCCAG
GACTTCAAGA CCGACCTCCG ATTCCAGAGC TCGGCCGTCA TGGCCCTGCA GGAGGCCAGC
GAGGCTTACC TCGTCGGTCT GTTTGAGGAC ACCAACTTGT GCGCCATCCA CGCCAAGCGT
---------- ---------- ---------- ---GCTAGTT GGCACTTCAT TTAGGGTTTT
AATTCGGTTA GAACTCTCTC GCCCCGGTGC ATTTTTAGGG GATGACCATC TTTATAATGT
TATTGTTACG GCCCACGCGT TTGTAATGAT TTTCTTCTTG GTAATGCCTA TAATAGTTGG
GGGGTTTGGT AATTGGCTTG TGCCTTTGAT GCTAACTTCT CCTGATATGG CGTTTCCTCG
GATGAATAAT ATAAGATTTT GGCTTCTTCC TCCGGCTTTG TCACTCCTCT TAGCGTCTGC
TTTTGTAGAG AGAGGAGTTG GAACTGGGTG AACTGTTTAT CCCCCTCTTT CTGCTAACGT
TGCGCATAGC GGGGGGTCTG TGGATTATGG AATTTTCTCT CTTCACTTAG CAGGTGTTTC
ATCGATTTTA GGGGCTATTA ATTTTTTGGC CAGAACAGTT AACATGCGGC CTGAGATTAT
AGAGTTTAAG CGGGTAACTT TGTTTATCTG GTCTATTGCC ATTACCGCTT TTTTGTTGGT
AGCGGCTATG CCAGTGTTGG CAGGTGCCAT CACAATACTT TTAACGGATC GAAATTTTAA
TACTTCGTTT TTTGACCCTT CGGGGGGAGG GGATCCTATC CTGTTTGTTC ---------

>Galeomma_sp
TAGTCATATG CTTGTCTCAA AGATTAAGCC ATGCATGTCT AAGTACACGC CACTGTAACG
GTGAAACTGC GAATGGCTCA TTAAATCAGT TATGGTTCCT TAGATCGTAC AATCCTACTT
GGATAACCGT GGCAATTCTA GAGCTAATAC ATGCGTCAAA GCTCCGACCT TCGGGGAAGA
GCGCTTTTGT TAGCAAGACC AATCCGGTCG GTTGTTGACT CTGAACAACT TTGTGCTGAT
CGCACGGCCT AGCGCCGGCG ATGTATCTTT CGAATGTCTG CCCTATCAAC TGTCGATGGT
ACGTGCTATG CCTACCATGG TTGTAACGGG TAACGGGGAA TCAGGGTTCG ATTCCGGAGA
GGGAGCATGA GAAACGGCTA CCACATCCAA GGAAGGCAGC AGGCGCGCAA ATTACCCAAT
CCCGACACGG GGAGGTAGTG ACGAAAAATA ACAATACGGG ACTCTTTCGA GGCCCCGTAA
TTGGAATGAG TACACTTTAA ATCCTTTAAC GAGGATCAAT TGGAGGGCAA GTCTGGTGCC
AGCAGCCGCG GTAATTCCAG CTCCAATAGC GTATATTAAA GTTGTTGCAG TTAAAAAGCT
CGTAGTTGGA TCTCGGGTGC AGGCTTGCGG TCCGCCTCGC GGCGGCTGCT CGTCCTGGCA
GCCTAGCTGC GGTATCCCTT GGTGCTCTTG ACTGAGTGTC GGTGGCCGGA ACGTTTACTT
TGATGAAATT AGAGTGTTCA AAGCGCGGGC GCTTGCCCGC ATAATGGTGC ATGGAATGAT

AGAATAGGAC CTCGGTTCTA TTTTGTTGGT TTTCGGAGAG GTAATGATTG AGAGGGACAG
ACGGGGGCAT TCGTATTGCG GCGTTAGAGG TGAAATTCTT GGATCGCCGC AAGACGGACT
ACAGCGAAAG CATTTGCCAA GAATGTTTTC CTTAATCAAG AACGAAAGTC AGAGGCTCGA
AGACGATCAG ATACCGTCGT AGTTCTGACC ATAAACCATG CCAACTGGCA ATCCGCCGGA
GTTACTACAA TGACTCGGCG AGCAGCCCCC GGGAAACCAA AGTTTCTGGG TTCCGGGGGG
AGTATGGTTG CAAAGCTGAA ACTTAAAGGA ATTGACGGAA GGGCACCACC AGGAGTGGAG
CCTGTGGCTT AATTTGACTC AACACGGGAA ACCTCACCCG GCCCGGACAC CGTTAGGATT
GACAGATTGA GAGCTCTTTC TTGATTCGGT GGGTGGTGGT GCATGGCCGT TCTTAGTTGG
TGGAGCGATT TGTCTGGTTA ATTCCGATAA CGAACGAGAC TCTAGCCTAC TAAATAGTTC
GAGGATATAT AACCTCGCAA CTTCTTAGAG GGACAGGTGG CGTATAGCCA CACGAGATTG
AGCAATAACA GGTCTGTGAT GCCCTTAGAT GTCCGGGGCC GCACACGCGC TACATTGAAT
GGATCAACGT GCGTCTAGCC TTGCCCGAAA GGGCTGGGAA ACCCGTTGAA ACCCATTCGT
GATAGGGACT GGGGCTTGCA ATTATTTCGC ATGAACGAGG AATTCCTAGT AAGCGCGAGT
CATCAGCTCG CACTGATTGC GTCCCTGCCC TTTGTACACA CCGCCCGTCG CTACTACCGA
TCGCTCCAGT TAATGAACGC CTCGGATTGG TTAAGCGGGT TTCGGCCTGC TCGCGTGCCG
AGAAGACGTG TAAATTATCT GGAGTAGAGG GAGTAAAAGT CGTAACAAGG TATCCATCAA
TAAGCGGAGG AAAAGAGACT AACTAGGATT CCCCTAGTAA CGGCGAGTGA AGCGGGAAGA
GCCCAGCACC GAATCCCCCG GCGTCTGGCC GCCGACGGGA CCTGTGGTGT TAGCGGGAGT
CTTTTGTCGG CGTGCTTCGG CACCAAAGTC CACCTGATCG TGGCAACCCC CGGCGGGTGA
GAGGCCCGTT CCGGTGCCGG ACGCGTCCGA CTCTCTCCAG GAGTCGGGTT GTTTGAGAAT
GCAGCCCAAA GTGGGTGGTA AACTCCACCT AAGGCTAAAT ACCGACACGA GTCCGATAGA
GGACAAGTAC CGTGAGGGAA AGTTGAAAAG AACTTTGAAG AGAGAGTTCA AGAGTACGTG
AAACCGCATA GAGTCAAACG GGTGGATCCG CAAGGTGTTG GACCGGGGAA TTCAGCGGTG
CACTTTCTCC GACGAGAGCC ACGACCGGGT CCGCTGCTCG AGGTGTGCGA AGGTTTCCAG
GCG-CCTGGG AGCTTACAGC GCGCCGTCCT CGAGTCTGTA CGTCGCGGGC CCGAGGACGC
GCCGCGCGCC CGGCACCGGG TCGTCTTCGT GCGTCCGACT GTCT-TGGCA GTGCGCCGCG
ACCGCGCGCG GTCCCGGCCG TGAGGGTCGG TGGCGAATCA GTCGGCACTC CACCCGACCC
GTCTTGAAAC ACGGACCAAG GAGTCTAACA TGTGCGCGAG TCATGGGGAC CCGAATCCCG
AAGGCGCAAT GAAAGTGAAG GCCGCCCTCG GCCGGCCGAG GCAGGATCCC GTCCCGGGCG
CACTGCCGGC CCGTCTCGAC AGCGGCGCGG TCGCGGCCTG CGTTCGTCTG CGAGGCGGAG
CAAGAGCGTA CACGTTGGTA CCCGAAAGAT GGTGAACTAT GCCTGAGCAG GACGAAGTCA
GAGGAAACTC TGATGGAGGT CCGCAGCGAT TCTAGAAAGT CCACTGGTGG GAAAGCCCCC
AGGAAACAGT TGGCCACCAA GGCCGCACGT AAGAGTGCAC CGGCCACCGG TGGTGTCAAG
AAGCCACACA GATACAGGCC CGGAACCGTG GCTCTCCGTG AGATCAGGAG ATACCAGAAG
AGCACCGAGC TTCTCATCCG AAAACTGCCA TTCCAGCGTC TCGTCCGCGA GATTGCCCAG
GACTTCAAGA CCGACCTCCG ATTCCAGAGC TCGGCCGTCA TGGCCCTGCA GGAGGCCAGC
GAGGCTTACC TCGTCGGTCT GTTTGAGGAC ACCAACTTGT GCGCCATCCA CGCCAAGCGT
AACATTGTAC TTTTTGTTTG GAATTTGGTC GGGGTTAGTA GGTACGTCAT TTAGGGTTTT
AATTCGATTA GAACTTTCTC GTCCTGGTGC GTTTTTGGGG GATGACCATC TTTATAATGT
TATTGTCACA GCCCACGCAT TTGTGATAAT TTTTTTTTTG GTTATACCTA TAATAGTTGG
GGGGTTTGGG AATTGGCTCG TACCTTTGAT GCTGACTTCT CCTGATATGG CGTTCCCCCG
GATGAACAAT ATAAGATTTT GGCTTCTTCC TCCGGCTTTG TCACTTCTTT TGGCATCTGC
TTTTGTGGAG AGAGGAGCTG GAACTGGTTG AACTGTTTAT CCCCCTCTTT CTGCTAATGT
TGCGCATAGC GGGGGATCTG TGGATTATGG AATCTTCTCT CTTCATTTGG CAGGTGTTTC
ATCGATTTTA GGGGCTATTA ATTTTTTGGC TAGGACAGTT AACATGCGGC CTGAGATTAT
AGAATTTAAA CGGGTAACTT TATTTATTTG GTCTATTGCT ATTACTGCTT TTTTATTAGT
AGCGGCTATG CCAGTGTTAG CAGGTGCTAT CACAATACTT NNGACGGATC GAAATTTTAA
TACCTCGTTC T--------- ---------- ---------- ---------- ---------

>Pseudogaleomma_sp

TAGTCATATG CTTGTCTCAA AGATTAAGCC ATGCATGTCT AAGTACACGC CACTGTAACG
GTGAAACTGC GAATGGCTCA TTAAATCAGT TATGGTTCCT TAGATCGTAC AATCCTACTT
GGATAACCGT GGCAATTCTA GAGCTAATAC ATGCGTCAAA GCTCCGACCT TCGGGGAAGA
GCGCTTTTGT TAGCAAGACC AATCCGGTCG GTTGTTGACT CTGAACAACT TTGTGCTGAT
CGCACGGCCT AGCGCCGGCG ATGTATCTTT CGAATGTCTG CCCTATCAAC TGTCGATGGT
ACGTGCTATG CCTACCATGG TTGTAACGGG TAACGGGGAA TCAGGGTTCG ATTCCGGAGA
GGGAGCATGA GAAACGGCTA CCACATCCAA GGAAGGCAGC AGGCGCGCAA ATTACCCAAT
CCCGACACGG GGAGGTAGTG ACGAAAAATA ACAATACGGG ACTCTTTCGA GGCCCCGTAA
TTGGAATGAG TACACTTTAA ATCCTTTAAC GAGGATCAAT TGGAGGGCAA GTCTGGTGCC
AGCAGCCGCG GTAATTCCAG CTCCAATAGC GTATATTAAA GTTGTTGCAG TTAAAAAGCT
CGTAGTTGGA TCTCGGGTGC AGGCTTGCGG TCCGCCTCGC GGCGGCTGCT CGTCCTGGCA
GCCTAGCTGC GGTATCCCTT GGTGCTCTTG ACTGAGTGTC GGTGGCCGGA ACGTTTACTT
TGATGAAATT AGAGTGTTCA AAGCGCGGGC GCTTGCCCGC ATAATGGTGC ATGGAATGAT
AGAATAGGAC CTCGGTTCTA TTTTGTTGGT TTTCGGAGAG GTAATGATTG AGAGGGACAG
ACGGGGGCAT TCGTATTGCG GCGTTAGAGG TGAAATTCTT GGATCGCCGC AAGACGGACT
ACAGCGAAAG CATTTGCCAA GAATGTTTTC CTTAATCAAG AACGAAAGTC AGAGGCTCGA
AGACGATCAG ATACCGTCGT AGTTCTGACC ATAAACCATG CCAACTGGCA ATCCGCCGGA
GTTACTACAA TGACTCGGCG AGCAGCCCCC GGGAAACCAA AGTTTCTGGG TTCCGGGGGG
AGTATGGTTG CAAAGCTGAA ACTTAAAGGA ATTGACGGAA GGGCACCACC AGGAGTGGAG
CCTGTGGCTT AATTTGACTC AACACGGGAA ACCTCACCCG GCCCGGACAC CGTTAGGATT
GACAGATTGA GAGCTCTTTC TTGATTCGGT GGGTGGTGGT GCATGGCCGT TCTTAGTTGG
TGGAGCGATT TGTCTGGTTA ATTCCGATAA CGAACGAGAC TCTAGCCTAC TAAATAGTTC
GAGGATATAT AACCTCGCAA CTTCTTAGAG GGACAGGTGG CGTATAGCCA CACGAGATTG
AGCAATAACA GGTCTGTGAT GCCCTTAGAT GTCCGGGGCC GCACACGCGC TACATTGAAT
GGATCAACGT GCGTCTAGCC TTGCCCGAAA GGGCTGGGAA ACCCGTTGAA ACCCATTCGT
GATAGGGACT GGGGCTTGCA ATTATTTCGC ATGAACGAGG AATTCCTAGT AAGCGCGAGT
CATCAGCTCG CACTGATTGC GTCCCTGCCC TTTGTACACA CCGCCCGTCG CTACTACCGA
TCGCTCCAGT TAATGAACGC CTCGGATTGG TTAAGCGGGT TTCGGCCTGC TCGCGTGCCG
AGAAGACGTG TAAATTATCT GGAGTAGAGG GAGTAAAAGT CGTAACAAGG TATCCATCAA
TAAGCGGAGG AAAAGAGACT AACTAGGATT CCCCTAGTAA CGGCGAGTGA AGCGGGAAGA
GCCCAGCACC GAATCCCCCG GCGTCTGGCC GCCGACGGGA CCTGTGGTGT TAGCGGGAGT
CTTTTGTCGG CGTGCTTCGG CACCAAAGTC CACCTGATCG TGGCAACCCC CGGCGGGTGA
GAGGCCCGTT CCGGTGCCGG ACGCGTCCGA CTCTCTCCAG GAGTCGGGTT GTTTGAGAAT
GCAGCCCAAA GTGGGTGGTA AACTCCACCT AAGGCTAAAT ACCGACACGA GTCCGATAGA
GGACAAGTAC CGTGAGGGAA AGTTGAAAAG AACTTTGAAG AGAGAGTTCA AGAGTACGTG
AAACCGCATA GAGTCAAACG GGTGGATCCG CAAGGTGTTG GACCGGGGAA TTCAGCGGTG
CACTTTCTCC GACGAGAGCC ACGACCGGGT CCGCTGCTCG AGGTGTGCGA AGGTTTCCAG
GCG-CCTGGG AGCTTACAGC GCGCCGTCCT CGAGTCTGTA CGTCGCGGGC CCGAGGACGC
GCCGCGCGCC CGGCACCGGG TCGTCTTCGT GCGTCCGACT GTCT-TGGCA GTGCGCCGCG
ACCGCGCGCG GATCCGGCCG TGAGGGTCGG TGGCGAATCA GTCGGCACTC CACCCGACCC
GTCTTGAAAC ACGGACCAAG GAGTCTAACA TGTGCGCGAG TCATGGGGAC CCGAATCCCG
AAGGCGCAAT GAAAGTGAAG GCCGCCCTCG GCCGGCCGAG GCAGGATCCC GTCCCGGGCG
CACTGCCGGC CCGTCTCGAC AGCGGCGCGG TCGCGGCCCG CGTTCGTCTG CGAGGCGGAG
CAAGAGCGTA CACGTTGGTA CCCGAAAGAT GGTGAACTAT GCCTGAGCAG GACGAAGTCA
GAGGAAACTC TGATGGAGGT CCGCAGCGAT TCTAGAAAGT CCACTGGCGG GAAAGCCCCC
AGGAAACAGT TGGCCACCAA GGCCGCCCGT AAGAGTGCAC CAGCCACTGG TGGTGTCAAG
AAGCCACACA GATACAGGCC TGGAACAGTG GCTCTCCGTG AAATCAGGAG ATACCAGAAG
AGCACCGAGC TTCTCATCCG AAAACTGCCA TTCCAGCGTC TGGTCCGCGA GATTGCCCAG
GACTTCAAGA CCGACCTCCG ATTCCAGAGC TCCGCCGTCA TGGCCCTGCA GGAAGCCAGC
GAGGCTTACC TCGTCGGTCT GTTTGAGGAC ACCAACTTGT GTGCCATCCA CGCCAAGCGT

TACACTATAT TTTTTATTTG GAATTTGATC GGGGCTAGTT GGTACCTCAT TTAGAGTTTT
GATTCGATTG GAGTTATCTC GACCAGGGGC ATTTTTAGGG GATGATCATT TGTATAATGT
TATCGTAACC GCACACGCTT TTGTTATAAT TTTCTTTTTG GTTATACCTA TAATAGTTGG
TGGATTTGGT AATTGGCTTG TGCCTTTAAT ACTAACTTCT CCTGACATAG CATTTCCTCG
AATGAATAAC ATAAGATTTT GGTTGCTTCC CCCTGCTTTG TCATTGTTAT TAGCATCAGC
TTTTGTTGAG AGAGGCGCAG GGACGGGTTG AACTGTTTAT CCTCCTTTGT CGGCTAATGT
TGCTCATAGC GGGGGTTCAG TAGATTATGG AATTTTCTCT CTTCATTTAG CAGGGGTTTC
GTCAATTTTA GGAGCTATTA ATTTTTTAGC TAGAACGGTT AATATGCGGC CAGAAATTAT
GGAATTTAAA CGGGTAACTT TGTTTATTTG GTCTATTGCT ATTACTGCTT TTTTGTTAGT
GGCCGCCATG CCAGTGTTGG CAGGGGCTAT CACAATGCTT TTAACTGATC GAAATTTTAA
TACGTCATTT TTTGACCCTT CAGGGGGAGG AGACCCTATT TTATTTGTCC ACTTGTTTT

>Scintilla_rosea
TAGTCATATG CTTGTCTCAA AGATTAAGCC ATGCATGTCT AAGTACACGC CACTGTAACG
GTGAAACTGC GAATGGCTCA TTAAATCAGT TATGGTTCCT TAGATCGTAC AATCCTACTT
GGATAACCGT GGCAATTCTA GAGCTAATAC ATGCGTCAAA GCTCCGACCT TCGGGGAAGA
GCGCTTTTGT TAGCAAGACC AATCCGGTCG GTTGTTGACT CTGAACAACT TTGTGCTGAT
CGCACGGCCT AGCGCCGGCG ATGTATCTTT CGAATGTCTG CCCTATCAAC TGTCGATGGT
ACGTGCTATG CCTACCATGG TTGTAACGGG TAACGGGGAA TCAGGGTTCG ATTCCGGAGA
GGGAGCATGA GAAACGGCTA CCACATCCAA GGAAGGCAGC AGGCGCGCAA ATTACCCAAT
CCCGACACGG GGAGGTAGTG ACGAAAAATA ACAATACGGG ACTCTTTCGA GGCCCCGTAA
TTGGAATGAG TACACTTTAA ATCCTTTAAC GAGGATCAAT TGGAGGGCAA GTCTGGTGCC
AGCAGCCGCG GTAATTCCAG CTCCAATAGC GTATATTAAA GTTGTTGCAG TTAAAAAGCT
CGTAGTTGGA TCTCGGGTGC AGGCTTGCGG TCCGCCTCGC GGCGGCTGCT CGTCCTGGCA
GCCTAGCTGC GGTATCCCTT GGTGCTCTTG ATTGAGTGTC GGTGGCCGGA ACGTTTACTT
TGATGAAATT AGAGTGTTCA AAGCGCGGGC GCTTGCCCGC ATAATGGTGC ATGGAATGAT
AGAATAGGAC CTCGGTTCTA TTTTGTTGGT TTTCGGAGAG GTAATGATTG AGAGGGACAG
ACGGGGGCAT TCGTATTGCG GCGTTAGAGG TGAAATTCTT GGATCGCCGC AAGACGGACT
ACAGCGAAAG CATTTGCCAA GAATGTTTTC CTTAATCAAG AACGAAAGTC AGAGGCTCGA
AGACGATCAG ATACCGTCGT AGTTCTGACC ATAAACCATG CCAACTGGCA ATCCGCCGGA
GTTACTACAA TGACTCGGCG AGCAGCCCCC GGGAAACCAA AGTTTCTGGG TTCCGGGGGG
AGTATGGTTG CAAAGCTGAA ACTTAAAGGA ATTGACGGAA GGGCACCACC AGGAGTGGAG
CCTGTGGCTT AATTTGACTC AACACGGGAA ACCTCACCCG GCCCGGACAC CGTTAGGATT
GACAGATTGA GAGCTCTTTC TTGATTCGGT GGGTGGTGGT GCATGGCCGT TCTTAGTTGG
TGGAGCGATT TGTCTGGTTA ATTCCGATAA CGAACGAGAC TCTAGCCTAC TAAATAGTTC
GAGGATATAT AACCTCGCAA CTTCTTAGAG GGACAGGTGG CGTATAGCCA CACGAGATTG
AGCAATAACA GGTCTGTGAT GCCCTTAGAT GTCCGGGGCC GCACACGCGC TACATTGAAT
GGATCAACGT GCGTCTAGCC TTGCCCGAAA GGGCTGGGAA ACCCGTTGAA ACCCATTCGT
GATAGGGACT GGGGCTTGCA ATTATTTCGC ATGAACGAGG AATTCCTAGT AAGCGCGAGT
CATCAGCTCG CACTGATTGC GTCCCTGCCC TTTGTACACA CCGCCCGTCG CTACTACCGA
TCGCTCCAGT TAATGAACGC CTCGGATTGG TTAAGCGGGT TTCGGCCTGC TCGCGTGCCG
AGAAGACGTG TAAATTATCT GGAGTAGAGG GAGTAAAAGT CGTAACAAGG TATCCATCAA
TAAGCGGAGG AAAAGAGACT AACTAGGATT CCCCTAGTAA CGGCGAGTGA AGCGGGAAGA
GCCCAGCACC GAATCCCCCG GCGTCTGGCC GCCGACGGGA CCTGTGGTGT TAGCGGGAGT
CTTTTGTCGG CGTGCTTCGG CACCAAAGTC CACCTGATCG TGGCAACCCC CGGCGGGTGA
GAGGCCCGTT CCGGTGCCGG ACGCGTCCGA CTCTCTCCAG GAGTCGGGTT GTTTGAGAAT
GCAGCCCAAA GTGGGTGGTA AACTCCACCT AAGGCTAAAT ACCGACACGA GTCCGATAGA
GGACAAGTAC CGTGAGGGAA AGTTGAAAAG AACTTTGAAG AGAGAGTTCA AGAGTACGTG
AAACCGCATA GAGTCAAACG GGTGGATCCG CAAGGTGTTG GACCGGGGAA TTCAGCGGTG
CACTTTCTCC GACGAGAGCC ACGACCGGGT CCGCTGCTCG AGGTGTGCGA AGGTTTCCAG

GCG-CCTGGG AGCTTACAGC GCGCCGTCCT CGAGTCTGTA CGTCGCGGGC CCGAGGACGC
GCCGCGCGCC CGGCACCGGG TCGTCTTCGT GCGTCCGACT GTCT-TGGCA GTGCGCCGCG
ACCGCGCGCG GATCCGGCCG TGAGGGTCGG TGGCGAATCA GTCGGCACTC CACCCGACCC
GTCTTGAAAC ACGGACCAAG GAGTCTAACA TGTGCGCGAG TCATGGGGAC CCGAATCCCG
AAGGCGCAAT GAAAGTGAAG GCCGCCCTCG GCCGGCCGAG GCAGGATCCC GTCCCGGGCG
CACTGCCGGC CCGTCTCGAC AGCGGCGCGG TCGCGGCCCG CGTTCGTCTG CGAGGCGGAG
CAAGAGCGTA CACGTTGGTA CCCGAAAGAT GGTGAACTAT GCCTGAGCAG GACGAAGTCA
GAGGAAACTC TGATGGAGGT CCGCAGCGAT TC???????? ?????????? ??????????
?????????? ?????????? ?????????? ?????????? ?????????? ??????????
?????????? ?????????? ?????????? ?????????? ?????????? ??????????
?????????? ?????????? ?????????? ?????????? ?????????? ??????????
?????????? ?????????? ?????????? ?????????? ?????????? ??????????
?????????? ?????????? ?????????? ?????????? ?????????? ??????????
TACATTGTAC TTTTTATTTG GAATTTGATC TGGATTAGTG GGCACATCAT TTAGGGTTTT
AATTCGGTTG GAGCTATCTC GGCCCGGGGC GTTTTTGGGG GATGATCATT TGTACAATGT
TATTGTAACC GCGCATGCTT TTGTTATAAT TTTCTTTTTG GTTATACCGA TAATAGTTGG
GGGGTTTGGT AATTGGCTTG TACCCTTAAT GTTAACCTCT CCTGATATAG CATTTCCTCG
AATAAATAAT ATAAGATTTT GGCTGCTTCC CCCTGCCTTG TCTCTATTAT TGGCATCTGC
TTTTGTTGAA AGAGGCGCAG GAACGGGTTG AACTGTTTAC CCCCCTTTAT CAGCTAATGT
TGCTCACAGC GGAGGTTCAG TAGATTACGG AATTTTTTCT CTTCATTTAG CAGGGGTTTC
GTCAATTTTA GGGGCTATTA ATTTTTTGGC TAGGACGGTT AATATGCGGC CAGAAATTAT
GGAGTTTAAA CGGGTTACTC TATTTATCTG ATCTATTGCT ATTACTGCTT TTCTGTTAGT
GGCCGCTATG CCAGTATTGG CGGGGGCTAT TACAATGCTT TTAACGGATC GAAACTTTAA
TACGTCATTC TTTGACCCTT CGGGGGGAGG GGACCCTATT TTATTTGTTC ACTTG----

>Scintilla_aff_hydatina
TAGTCATATG CTTGTCTCAA AGATTAAGCC ATGCATGTCT AAGTACACGC CAGATTAAAG
GTGAAACTGC GAATGGCTCA TTAAATCAGT TATGGTTCCT TAGATCGTAC AATCCTACTT
GGATAACCGT GGCAATTCTA GAGCTAATAC ATGCGTCAAA GCTCCGACCT TCGGGGAAGA
GCGCTTTTGT TAGCAAAACC AATCCGGTCG GTTGTTGACT CTGAACAACT TTGTGCTGAT
CGCACGGCCT AGCGCCGGCG ATGTATCTTT CGAATGTCTG CCCTATCAAC TGTCGATGGT
ACGTGCTATG CCTACCATGG TTGTAACGGG TAACGGGGAA TCAGGGTTCG ATTCCAGAGA
GGGAGCATGA GAAACGGCTA CCACATCCAA GGAAGGCAGC AGGCGCGCAA ATTACCCAAT
CCCGACACGG GGAGGTAGTG ACGAAAAATA ACAATACGGG ACTCTTTCGA GGCCCCGTAA
TTGGAATGAG TACACTTTAA ATCCTTTAAC GAGGATCAAT TGGAGGGCAA GTCTGGTGCC
AGCAGCCGCG GTAATTCCAG CTCCAATAGC GTATATTAAA GTTGTTGCAG TTAAAAAGCT
CGTAGTTGGA TCTCGGGTGC AGGCGTGCGG TCCGCCTCGC GGCGTCTGCA CGTCCTGGCA
GCCTAGCTGC GGTATCCCTT GGTGCTCTTG ACTGAGTGTC GGTGGCCGGA ACGTTTACTT
TGATGAAATT AGAGTGTTCA AAGCGCGGGC GCTTGCCCGC ATAATGGTGC ATGGAATGAT
AGAATAGGAC CTCGGTTCTA TTTTGTTGGT TTTCGGAGAG GTAATGATTG AGAGGGACAG
ACGGGGGCAT TCGTATTGCG GCGTTAGAGG TGAAATTCTT GGATCGCCGC AAGACGGACT
ACAGCGAAAG CATTTGCCAA GAATGTTTTC CTTAATCAAG AACGAAAGTC AGAGGCTCGA
AGACGATCAG ATACCGTCGT AGTTCTGACC ATAAACCATG CCAACTGGCA ATCCGCCGGA
GTTACTACAA TGACTCGGCG AGCAGCCCCC GGGAAACCTA AGTTTCTGGG TTCCGGGGGG
AGTATGGTTG CAAAGCTGAA ACTTAAAGGA ATTGACGGAA GGGCACCACC AGGAGTGGAG
CCTGTGGCTT AATTTGACTC AACACGGGAA ACCTCACCCG GCCCGGACAC CGGTAGGATT
GACAGATTGA GAGCTCTTTC TTGATTCGGT GGGTGGTGGT GCATGGCCGT TCTTAGTTGG
TGGAGCGATT TGTCTGGTTA ATTCCGATAA CGAACGAGAC TCTAGCCTAC TAAATAGTTC
GAGGATATAT AACCTCGCAA CTTCTTAGAG GGACAGGTGG CGTATAGCCA CACGAGATTG
AGCAATAACA GGTCTGTGAT GCCCTTAGAT GTCCGGGGCC GCACACGCGC TACATTGAAT

GGATCAGCGT GCGTCTAGCC TGGCCCGAAA GGGCTGGGAA ACCCGTTGAA ACCCATTCGT
GATAGGGACT GGGGCTTGCA ATTCTTTCGC ATGAACGAGG AATTCCTAGT AAGCGCGAGT
CATCAGCTCG CGCTGATTGC GTCCCTGCCC TTTGTACACA CCGCCCGTCG CTACTACCGA
TCGCTCCAGT TAATGAACGC CTCGGATTGG TTAAGCGGGT TTCGGCCTGC TCGCGTGCCG
AGAAGACGTG TAAATTATCT GGAGTAGAGG GAGTAAAAGT CGTAACAAGG TATCCATAAA
TAAGCGGAGG AAAAGAAACT AACTAGGATT CCCCTAGTAA CGGCGAGTGA AGCGGGAAGA
GCCCAGCACC GAATCCCCCT GCGGCTGGCC GCGGACGGGA CATGTGGTGT TAGCGGGAGT
CCCTTGTCGG CGCACTTCGG CACCCAAGTC CACCTGATCG TGGCTATCCC CGGCGGGTGA
GAGGCCCGTT CCGGTGTCGG ACGCGCACGA CTCTCTCCAG GAGTCGGGTT GTTTGAGAAT
GCAGCCCAAA GTGGGTGGTA AACTCCACCT AAGGCTAAAT ACAGACACGA GTCCGATAGA
GGACAAGTAC CGTGAGGGAA AGTTGAAAAG AACTTTGAAG AGAGAGTTCA AGAGTACGTG
AAACCGCATA GAGTCAAACG GGTGGAACCG CAAGGTGTTG GACCGGGGAA TTCAGCGGTG
CACTTTCTCC GACGAGAGCC ATGACCGGGT CCGCTGCTCG AGGTGTGCGA AGGTATCCCG
GCGCGTT-GG GAGTTATAGC GCGCCGTCCT CGAGTCGGTA CGCCGCGGAC CCGAGGACGC
GCCGCGCGCC TGGCACCGGG TCGTCTTCGT GCGTTCGACT GTTCTCGGCA GTGCGTCGCG
ACCGCGCGCG GTTCCGGTG- TGAGGGTCGG TGGCGAATCA GTCGGCACTC CACCCGACCC
GTCTTGAAAC ACGGACCAAG GAGTCTAACA TGTGCGCGAG TCATAGGGAC GCGCATCCCG
AAGGCGCAAT GAAAGTGAAG GCCGCCCTCG GCTGGCCGAG GCAGGATCCC GTCGTGGGCG
CACTGCCGGC CCGTCTCGAT AGCGGCGCGC TCGCGGGCCG CGTTCGTCTA CGAGGCGGAG
CAAGAGCGTA CACGTTGGTA CCCGAAAGAT GGTGAACTAT GCCTGAGCAG GACGAAGTCA
GAGGAAACTC TGATGGAGGT CCGCAGCGAT TCTAGAAAGT CCACTGGTGG CAAGGCCCCC
AGGAAACAAC TGGCCACAAA GGCCGCACGT AAGAGTGCAC CGGCCACTGG TGGCGTCAAG
AAGCCACACA GATACAGGCC AGGAACCGTC GCTCTCCGTG AGATCAGGAG ATACCAGAAG
AGCACCGAGC TTTTGATCAG AAAGCTGCCC TTCCAGCGTC TGGTCAGAGA GATTGCCCAG
GACTTCAAGA CCGACCTCCG ATTCCAGAGC TCTGCCGTCA TGGCCCTGCA GGAAGCCAGC
GAGGCTTACC TCGTCGGACT GTTTGAGGAC ACCAACTTGT GCGCTATCCA CGCCAAGCGT
---------- ---------- -------ATC TGGGCTTGTT GGTACTTCTT TTAGGGTATT
AATTCGTTTA GAGCTTTNAC NACCCGGGGC TTTTCTTGGA GATGATCATT TGTATAATGT
GATTGTTACG GCGCATGCTT TTGTCATGAT TTTTTTCTTG GTTATGCCGA TGATGGTGGG
TGGATTTGGA AATTGGCTTG TGCCTTTAAT ACTAACAGCT CCTGATATGG CTTTCCCTCG
AATAAACAAT ATAAGGTTTT GGCTTTTGCC GCCTGCTTTA TCTTTATTAC TGGCTTCTGC
TTTTGTTGAA AGGGGGGCGG GGACTGGGTG AACGGTTTAC CCGCCTTTGT CAGCTAATGT
GGCCCATAGA GGGGGTTCTG TGGACTATGC TATTTTTTCT CTTCACTTAN CAGGTGTTTC
TTCAATTTTG GGGGCTATTA ACTTTTTGGC AAGGACTGTA AATATGCGAC CTGAAATTAT
AGAGTTTAAG CGGGTTACTT TATTTATTTG ATCTATTGCT ATTACCGCCT TTTTATTAGT
TGCAGCGATG CCGGTGTTGG CTGGGGCCAT TACAATACTT TTGACGGATC GAAATTTTAA
CACATCTTTT TTTGATCCTT CGGGAGGTGG GGACCCTATT CTGTTTGTCC ATCTCTTTT

>Scintilla_sp1
TAGTCATATG CTTGTCTCAA AGATTAAGCC ATGCATGTCT AAGTACACGC CACTGTAACG
GTGAAACTGC GAATGGCTCA TTAAATCAGT TATGGTTCCT TAGATCGTAC AATCCTACTT
GGATAACCGT GGCAATTCTA GAGCTAATAC ATGCGTCAAA GCTCCGACCT TCGGGGAAGA
GCGCTTTTGT TAGCAAAACC AATCCGGTCG GTTGTTGACT CTGAACAACT TTGTGCTGAT
CGCACGGCCT AGCGCCGGCG ATGTATCTTT CGAATGTCTG CCCTATCAAC TGTCGATGGT
ACGTGCTATG CCTACCATGG TTGTAACGGG TAACGGGGAA TCAGGGTTCG ATTCCGGAGA
GGGAGCATGA GAAACGGCTA CCACATCCAA GGAAGGCAGC AGGCGCGCAA ATTACCCAAT
CCCGACACGG GGAGGTAGTG ACGAAAAATA ACAATACGGG ACTCTTTCGA GGCCCCGTAA
TTGGAATGAG TACACTTTAA ATCCTTTAAC GAGGATCAAT TGGAGGGCAA GTCTGGTGCC
AGCAGCCGCG GTAATTCCAG CTCCAATAGC GTATATTAAA GTTGTTGCAG TTAAAAAGCT
CGTAGTTGGA TCTCGGGTGC AGGCTTGCGG TCCGCCTCGC GGCGGCTGCT CGTCCTGGCA

GCCTAGCTGC GGTATCCCTT GGTGCTCTTG ACTGAGTGTC GGTGGCCGGA ACGTTTACTT
TGATGAAATT AGAGTGTTCA AAGCGCGGGC GCTTGCCCGC ATAATGGTGC ATGGAATGAT
AGAATAGGAC CTCGGTTCTA TTTTGTTGGT TTTCGGAGAG GTAATGATTG AGAGGGACAG
ACGGGGGCAT TCGTATTGCG GCGTTAGAGG TGAAATTCTT GGATCGCCGC AAGACGGACT
ACAGCGAAAG CATTTGCCAA GAATGTTTTC CTTAATCAAG AACGAAAGTC AGAGGCTCGA
AGACGATCAG ATACCGTCGT AGTTCTGACC ATAAACCATG CCAACTGGCA ATCCGCCGGA
GTTACTACAA TGACTCGGCG AGCAGCCCCC GGGAAACCTA AGTTTCTGGG TTCCGGGGGG
AGTATGGTTG CAAAGCTGAA ACTTAAAGGA ATTGACGGAA GGGCACCACC AGGAGTGGAG
CCTGTGGCTT AATTTGACTC AACACGGGAA ACCTCACCCG GCCCGGACAC CGTTAGGATT
GACAGATTGA GAGCTCTTTC TTGATTCGGT GGGTGGTGGT GCATGGCCGT TCTTAGTTGG
TGGAGCGATT TGTCTGGTTA ATTCCGATAA CGAACGAGAC TCTAGCCTAC TAAATAGTTC
GAGGATATAT AACCTCGCAA CTTCTTAGAG GGACAGGTGG CGTATAGCCA CACGAGATTG
AGCAATAACA GGTCTGTGAT GCCCTTAGAT GTCCGGGGCC GCACACGCGC TACATTGAAT
GGATCAACGT GCGTCTAGCC TTGCCCGAAA GGGCTGGGAA ACCCGTTGAA ACCCATTCGT
GATAGGGACT GGGGCTTGCA ATTATTTCGC ATGAACGAGG AATTCCTAGT AAGCGCGAGT
CATCAGCTCG CGTTGATTGC GTCCCTGCCC TTTGTACACA CCGCCCGTCG CTACTACCGA
TCGCTCCAGT TAATGAACGC CTCGGATTGG TTAAGCGGGT TTCGGCCTGC TCGCGTGCCG
AGAAGACGTG TAAATTATCT GGAGTAGAGG GAGTAAAAGT CGTAACAAGG TATCCATTAA
TAAGCGGAGG AAAAGAGACT AACTAGGATT CCCCTAGTAA CGGCGAGTGA AGCGGGAAGA
GCCCAGCACC GAATCCCCCG GCATCTGGCA GCCGACGGGA CATGTGGTGT TAGCGGGAGT
CTTTTGTCGG CGTGCTTCGG CACCAAAGTC CACCTGATCG TGGCAACCCC CGGCGGGTGA
GAGGCCCGTT CCGGTGCCGG ACGCGCACGA CTCTCTCCAG GAGTCGGGTT GTTTGAGAAT
GCAGCCCAAA GTGGGTGGTA AACTCCACCT AAGGCTAAAT ACAGACACGA GTCCGATAGA
GGACAAGTAC CGTGAGGGAA AGTTGAAAAG AACTTTGAAG AGAGAGTTCA AGAGTACGTG
AAACCGCATA GAGTCAAACG GGTGGATCCG CAAGGTGTTG GACCGGGGAA TTCAGCGGTG
CACTTTCTCC GACGAGAGCC ACGACCGGGT CCGCCGCTCG AGGTGTGCGA AGGTTTCCAG
GTGCCCTGGG AGCTTACAGC GCGCCGTCCT CGAGTCGGTA CGTCGCGGGC CCGAGGACGC
GCCGTGCGCC CGGCACCGGG TCGTCTTCGT GCGTTCGACT GTCT-GGGCA GTGCGTCGCG
ACCGCGTGCG GTCTCGGTCG TGAGGGTCGG TGGCGAATCA GTCGGCACTC CACCCGACCC
GTCTTGAAAC ACGGACCAAG GAGTCTAACA TGTGCGCGAG TCATAGGGAC CCGAATCCCG
AAGGCGCAAT GAAAGTGAAG GCCGGGCTCG CCCGGCCGAG GCAGGATCCC GTCCCGGGCG
CACTGCCGGC CCGTCTCGAT AGCGGCGCGG TCGCGGCCTG CGTTCGTCTG CGAGGCGGAG
CAAGAGCGTA CACGTTGGTA CCCGAAAGAT GGTGAACTAT GCCTGAGCAG GACGAAGTCA
GAGGAAACTC TGATGGAGGT CCGCAGCGAT TCTAGAAAGT CCACTGGTGG GAAAGCCCCC
AGGAAACAGT TGGCCACAAA GGCTGCACGT AAGAGTGCAC CGGCCACTGG TGGTGTCAAG
AAGCCACACA GATACAGGCC CGGAACCGTC GCTCTCCGTG AAATCAGGAG ATACCAGAAG
AGCACCGAGC TTCTCATCCG AAAACTGCCC TTCCAGCGTC TAGTCCGTGA GATTGCCCAA
GACTTCAAGA CCGACCTCCG ATTCCAGAGC TCCGCCGTCA TGGCCCTGCA AGAAGCCAGC
GAGGCTTACC TGGTTGGTCT GTTTGAGGAC ACCAACTTGT GCGCTATCCA CGCCAAGCGT
AACATTATAT TTTTTATTTG GAATTTGATC GGGGTTAGTA GGAACGTCGT TCAGAGTGTT
AATTCGGTTG GAGTTGTCAC GTCCGGGAGC TTTTCTAGGG GATGATCATC TTTATAATGT
TATTGTTACG GCGCATGCGT TTGTAATAAT TTTCTTTTTG GTTATGCCAA TAATGGTTGG
GGGTTTTGGA AACTGGCTGG TTCCATTGAT ACTAACTTCT CCTGATATGG CGTTTCCACG
AATGAATAAT ATAAGGTTTT GGCTTCTTCC TCCGGCTTTA TCACTTCTTT TGGCGTCTGC
TTTCGTAGAA AGAGGAGCTG GGACTGGGTG AACTGTTTAC CCTCCTTTGT CAGCAAATGT
TGCTCATAGA GGCGGGTCAG TAGATTATGG AATTTTTTCT CTTCATTTAG CGGGTGTTTC
GTCAATTTTA GGGGCTATTA ACTTTTTGGC TAGAACTGTT AATATGCGGC CTGAGATTAT
GGAGTTTAAG CGAGTAACGT TGTTCATTTG GTCTATTGCT ATTACGGCAT TTTTGTTGGT
GGCGGCGATG CCTGTGTTAG CGGGGGCTAT TACTATACTC TTGACTGATC GGAATTTTAA
TACTTCATTT TTTGACCCTT CAGGAGGAGG TGATCCTATC CTGTTTGTCC ATTTATTTT

>Scintilla_sp2
TAGTCATATG CTTGTCTCAA AGATTAAGCC ATGCATGTCT AAGTACACGC CAGATTAATG
GTGAAACTGC GAATGGCTCA TTAAATCAGT TATGGTTCCT TAGATCGTAC AATCCTACTT
GGATAACCGT GGCAATTCTA GAGCTAATAC ATGCGTCAAA GCTCCGACCT TCGGGGAAGA
GCGCTTTTGT TAGCAAAACC AATCCGGTCG GTTGTTGACT CTGAACAACT TTGTGCTGAT
CGCACGGCCT AGCGCCGGCG ATGTATCTTT CGAATGTCTG CCCTATCAAC TGTCGATGGT
ACGTGCTATG CCTACCATGG TTGTAACGGG TAACGGGGAA TCAGGGTTCG ATTCCGGAGA
GGGAGCATGA GAAACGGCTA CCACATCCAA GGAAGGCAGC AGGCGCGCAA ATTACCCAAT
CCCGACACGG GGAGGTAGTG ACGAAAAATA ACAATACGGG ACTCTTTCGA GGCCCCGTAA
TTGGAATGAG TACACTTTAA ATCCTTTAAC GAGGATCAAT TGGAGGGCAA GTCTGGTGCC
AGCAGCCGCG GTAATTCCAG CTCCAATAGC GTATATTAAA GTTGTTGCAG TTAAAAAGCT
CGTAGTTGGA TCTCGGGTGC AGGCTTGCGG TCCGCCTCGC GGCGGCTGCT CGTCCTGGCA
GCCTAGCTGC GGTATCCCTT GGTGCTCTTG ACTGAGTGTC GGTGGCCGGA ACGTTTACTT
TGATGAAATT AGAGTGTTCA AAGCGTGGGC GCTTGCCCGC ATAATGGTGC ATGGAATGAT
AGAATAGGAC CTCGGTTCTA TTTTGTTGGT TTTCGGAGAG GTAATGATTG AGAGGGACAG
ACGGGGGCAT TCGTATTGCG GCGTTAGAGG TGAAATTCTT GGATCGCCGC AAGACGGACT
ACAGCGAAAG CATTTGCCAA GAATGTTTTC CTTAATCAAG AACGAAAGTC AGAGGCTCGA
AGACGATCAG ATACCGTCGT AGTTCTGACC ATAAACCATG CCGACTGGCA ATCCGCCGGA
GTTACTACAA TGACTCGGCG AGCAGCCCCC GGGAAACCAA AGTTTCTGGG TTCCGGGGGG
AGTATGGTTG CAAAGCTGAA ACTTAAAGGA ATTGACGGAA GGGCACCACC AGGAGTGGAG
CCTGTGGCTT AATTTGACTC AACACGGGAA ACCTCACCCG GCCCGGACAC CGTTAGGATT
GACAGATCGA GAGCTCTTTC TTGATTCGGT GGGTGGTGGT GCATGGCCGT TCTTAGTTGG
TGGAGCGATT TGTCTGGTTA ATTCCGATAA CGAACGAGAC TCTAGCCTAC TAAATAGTTC
GAGGATATAT AACCTCGCAA CTTCTTAGAG GGACAGGTGG CGTATAGCCA CACGAGATTG
AGCAATAACA GGTCTGTGAT GCCCTTAGAT GTCCGGGGCC GCACACGCGC TACATTGAAT
GGATCAACGT GCGTCTAGCC TTGCCCGAAA GGGCTGGGAA ACCCGTTGAA ACCCATTCGT
GATAGGGACT GGGGCTTGCA ATTATTTCCC ATGAACGAGG AATTCCTAGT AAGCGCGAGT
CATCAGCTCG CGTTGATTGC GTCCCTGCCC TTTGTACACA CCGCCCGTCG CTACTACCGA
TCGCTCCAGT TAATGAACGC CTCGGATTGG TTAAGCGGGT TTCGGCCTGC TCGCGTGCCG
AGAAGACGTG TAAATTATCT GGAGTAGAGG GAGTAAAAGT CGTAACAAGG TATCCATCAA
TAAGCGGAGG AAAAGAGACT AACAAGGATT CCCCCAGTAA CGGCGAGTGA AGCGGGAAAA
GCCCAGCACC GAATCCCCCG GCGTCTGGCC GCCGACGGGA CCTGTGGTGT TAGCGGGAGT
CTTTTGTCGG CGTGCTTCGG CACCAAAGTC CACCTGATCG TGGCAACCCC TGGCGGGTGA
GAGGCCCGTT CCGGTGTCGG ACGCGTTCGA CTCTCTCCAG GAGTCGGGTT GTTTGAGAAT
GCAGCCCAAA GTGGGTGGTA AACTCCACCT AAGGCTAAAT ACGGACACGA GTCCGATAGA
GGACAAGTAC CGTGAGGGAA AGTTGAAAAG AACTTTGAAG AGAGAGTTCA AGAGTACGTG
AAACCGCATA GAGGCAAACG GGTGGATCCG CAAGGTGTTG GACCGGGGAA TTCAGCGGTG
CACTTTCTCC GACGAGAGCC ACGACCGGGT CTGCTGCTCG AGGCGTGCGA AGGTTTCCAG
GCGCCTCGGG AGCTTACAGC GCGCCGCCAT CGAGTCTGTA CGTCGCGGGC CCGAGGACGC
GCCGCGCGCC TGGCACCGGG TCGTCTTCGC GCGTTCGACT GTTCTCGGCA GTGCGCTGCG
ACCGCGTGCG GTCCCGGCG- TGAGGGTCGG TGGCGAATCA GTCGGTGCTC CACCCGACCC
GTCTTGAAAC ACGGACCAAG GAGTCTAACA TGTGCGCGAG TCATGGGGAC CCGAATCCCG
AAGGCGCAAT GAAAGTGAAG GCCGCCCACG GCTGGCCGAG GCAGGATCCC GTCGCGGGCG
CACTGCCGGC CCGTCTCGAT AGCGGCGCGG TCGCGGCCTG CGTTCGTCTA CGAGGCGGAG
CAAGAGCGTA CACGTTGGTA CCCGAAAGAT GGTGAACTAT GCCTGAGCAG GACGAAGTCA
GAGGAAACTC TGATGGAGGT CCGCAGCGAT TCAAGAAAAT CCACTGGTGG GAAAGCCCCC
AGGAAACAGC TGGCCACCAA GGCCGCACGT AAGAGTGCAC CAGCCACTGG TGGTGNGAAG
AAACCACACA GGTACAGGCC CGGAACTGTC GCTCTCCGTG AAATCAGGAG ATACCAGAAG
AGCACCGAGC TGCTGATCAG AAAACTGCCT TTCCAGCGTC TGGTCCGTGA AATCGCTCAG

GACTTCAAGA CTGACCTCCG ATTCCAGAGC TCTGCAGTCA TGGCTTTGCA GGAGGCCAGT
GAAGCTTACT TGGTCGGACT TTTCGAAGAC ACCA------ ---------- ----------
TACTCTTTAT TTTCTATTTG GTATCTGGTC TGGGCTAGTT GGAACTTCGT TTAGGGTTTT
AATTCGGTTG GAATTGTCTC GGCCGGGTGC GTTTTTGGGG GATGACCATT TATACAACGT
TATTGTTACG GCGCATGCAT TTGTGATAAT TTTTTTCTTA GTAATACCAA TAATAGTAGG
GGGGTTTGGA AATTGATTGG TTCCATTGAT GTTGACATCC CCCGACATGG CATTCCCTCG
AATGAATAAT ATGAGATTTT GGCTGCTTCC TCCTGCGTTA TTTTTACTAT TAAGGTCGGC
TTTTGTTGAA AGAGGGGTTG GTACTGGTTG GACAGTCTAC CCCCCGTTGT CAGCTAATGT
AGCTCACAGC GGCGGGTCAG TTGATTATGG TATTTTTTCT TTACACTTGG CTGGTGTTTC
ATCAATTTTA GGAGCTATTA ATTTTTTGGC TAGGACTGTT AATATGCGAC CAGAAATTAT
GGAGTTTAAA CGTGTTACCC TATTTGTGTG GTCTATTGCT ATTACGGCGT TTTTATTGGT
GGTAGCTATG CCTGTTTTGG CCGGAGCTAT TACGATATTG TTGACAGATC GTAATTTTAA
CACATCATTT TTCGATCCTT CAGGGGGTGG TGATCCAATT CTGTTTGTTC ATTTATTTT

>Scintillona_stigmatica
TAGTCATATG CTTGTCTCAA AGATTAAGCC ATGCATGTCT AAGTACACGC CAGATACAAG
GTGAAACTGC GAATGGCTCA TTAAATCAGT TATGGTTCCT TAGATCGTAC AATCCTACTT
GGATAACTGT GGCAATTCTA GAGCTAATAC ATGCATCAAA GCTCCGACCT TCGGGGAAGA
GCGCTTTTGT TAGCAAAACC AATGCGGCCG CTTGTTGACT CTGAACAACT TTGTGCTGAT
CGCACGGCCT CGCGCCGGCG ACGAATCTTT TGAATGTCTG CCCTATCAAC TGTCGATGGT
ACGTGCTATG CCTACCATGG TTGTAACGGG TAACGGGGAA TCAGGGTTCG ATTCCGGAGA
GGGAGCATGA GAAACGGCTA CCACATCCAA GGAAGGCAGC AGGCGCGCAA ATTACCCAAT
CCCGACACGG GGAGGTAGTG ACGAAAAATA ACAATACGGG ACTCTTTCGA GGCCCCGTAA
TTGGAATGAG TACACTTTAA ATCCTTTAAC GAGGATCCAT TGGAGGGCAA GTCTGGTGCC
AGCAGCCGCG GTAATTCCAG CTCCAATAGC GTATATTAAA GTTGTTGCAG TTAAAAAGCT
CGTAGTTGGA TCTCGGGTGC AGGCTTGCGG TCCGCCTCGC GGTGGCTGCT CGTCCTGGCA
GCCTAGCTGC GGTG-CCCTT GGTGCTCTTG ACTGAGTGTC GGTGGCCGGA ACGTTTACTT
TGATGAAATT AGAGTGTTCA AAGCGCGGGC GTTTGCCCGC ATAATGGTGC ATGGAATGAT
GGAATAGGAC CTCGGTTCTA TTTTGTTGGT TTTCGGAGAG GTAATGATTG AGAGGGACAG
ACGGGGGCAT TCGTATTGCG GCGTTAGAGG TGAAATTCTT GGATCGCCGC AAGACGGACG
AGAGCGAAAG CATTTGCCAA GAATGTTTTC CTTAATCAAG AACGAAAGTC AGAGGCTCGA
AGACGATCAG ATACCGTCGT AGTTCTGACC ATAAACCATG CCAACTGGCA ATCCGCCGGA
GTTACTACAA TGACTCGGCG AGCTGCCCCC GGGAAACCAA AGTTTTTGGG TTCCGGGGGG
AGTATGGTTG CAAAGCTGAA ACTTAAAGGA ATTGACGGAA GGGCACCACC AGGAGTGGAG
CCTGTGGCTT AATTTGACTC AACACGGGAA ACCTCACCCG GCCCGGACAC TGTAAGGATT
GACAGATTGA GAGCTCTTTC TTGATTCGGT GGGTGGTGGT GCATGGCCGT TCGTAGTTGG
TGGAGCGATT TGTCTGGTTA ATTCCGATAA CGAACGAGAC TCTAGCCTGC TAACTAGTTC
GAGGCTATAT AGCCTCGCAA CTTCTTAGAG GGACAGGTGG CGTATAGCCA CACGAGATTG
AGCAATAACA GGTCTGTGAT GCCCTTAGAT GTTCGGGGCC GCACACGCGC TACATTGAAT
GGATCAACGT GCTTTTAGCC TTGCCCGAAA GGGCTGGGAA ACCCGTTGAA ACCCATTCGT
GCTAGGGATT GGGGCTTGCA ATTATTTCCC ATGAACGAGG AATTCCTAGT AAGCGCGAGT
CATCAGCTCG CGTTGATTGC GTCCCTGCCC TTTGTACACA CCGCCCGTCG CTACTACCGA
TCGCTCCAGT TAATGAACGC TTCGGATTGG TTAAGCGGGT TTCGGCCTGC TCGCGTGCCG
AGAAGATGCG TGAATTATCC GGGGTAGAGG TCGTAAAAGT CGTAACAAGG TATCCATTAA
TAAGCGGAGG AAAAGAAACT AACTAGGATT CCCCTAGTAA CGGCGAGTGA AGCGGGAAGA
GCCCTGCACC GAATCCCCTG GCGTCTGGTC GCCGGCGGGA AATGTGGTGT TAGCGGGAGC
CCTACGTCGG CGTGCTCTGG CACCTAAGTC CACTTGATTG TGGCAACCCA TGGCGGGTGA
GAGGCCCGTG TCGGTGCCGG ACGCGTTCGA CTCTCTCCAG GAGTCGGGTT GTTTGAGAAT
GCAGCCCAAA GTGGGTGGTA AACTCCATCT AAGGCTAAAT ACAGACACGA GTCCGATAGA
GGACAAGTAC CGTGAGGGAA AGTTGAAAAG AACTTTGAAG AGAGAGTTCA AGAGTACGTG

AAACCGCATA GAGGCAAACG GGTGGATCCG CAAGGTGTCG GACCGGGGAA TTCAGCGGCG
CACTTTCTCC GACGAAAGCC ACGACCGGGA CCGCTGCTCG AGGTGCGACA AGGTTGTCCG
GCGCTTCGGT CGCTTACAGG TCGCCGTCCT CGAGCTGGAA AGGCGT-GTC CCGAGGATGC
GCCGCGCGCA CGGCCCCGGA CCGTTCTGGT GCGTTCGACT T--TCTGGCA GTGTGCCGAG
ACCGCGCGCC GGTTCGGGTG CGAGGGTCGG TGGCGAATCA GTCGGTGCTC CACCCGACCC
GTCTTGAAAC ACGGACCAAG GAGTCTAACA TGTGCGCGAG TCGTAGGGAC CCAAATCCCG
AAGGCGCAAT GAAAGTGAAG GTCGTCCTCG GTCGGCCGAG GCAGGATCCC GTCGCGGGCG
CACTGCCGGC CCGTCTCGAC GGCGGGGGCT CTGGGGCCC- --TTCGTCCG CGAGGCGGAG
CAAGAGCGTA CACGTTGGTA CCCGAAAGAT GGTGAACTAT GCCTGAGCAG GACGAAGTCA
GAGGAAACTC TGATGGAGGT CCGCAGCGAT TC???????? ?????????? ??????????
?????????? ?????????? ?????????? ?????????? ?????????? ??????????
?????????? ?????????? ?????????? ?????????? ?????????? ??????????
?????????? ?????????? ?????????? ?????????? ?????????? ??????????
?????????? ?????????? ?????????? ?????????? ?????????? ??????????
?????????? ?????????? ?????????? ?????????? ?????????? ??????????
TACTTTATAT TTTATTTTTG GGGTTTGGTC GGGAATGGTT GGGACATCAC TCAGGGTGTT
AATTCGTTTA GAATTGTCTC GGCCTGGGGC TTTTTTAGGT GATGACCATC TTTATAATGT
TATTGTCACT GCCCATGCAT TTGTGATAAT CTTTTTTTTA GTAATGCCTA TAATAATGGG
CGGGTTTGGG AATTGACTTA TTCCTTTAAT GTTAACTTCT CCTGATATGG CTTTTCCTCG
AATGAACAAT ATGAGGTTTT GACTTTTACC CCCTGCTTTG TTTTTGTTAG TTAGTTCTGC
TTTTGTGGAG AGGGGGGTTG GCTCTGGATG AACTGTTTAT CCGCCGCTTT CTGGTAATGT
GACTCATAGG GGGGGGTCGG TGGATTATGC CATTTTTTCT CTTCACTTGG CTGGGGTTTC
CTCTATTTTA GGGTCTATTA ATTTTTTGGG GAGAACTTTG AATATGCGCC CTGAAATTAT
GGAATTTAAA CGGGTGACCC TGTTTGTTTG GTCTGCTGCT ATTACGGCTT TTTTACTGGT
TTTAGCTATG CCAGTTTTGG CTGGGGCTAT TACTATGTTG TTAACTGATC GTAATTTTAA
TACGTCTTTT TTTGATCCTT CTGGTGGTGG GGATCCAATT TTGTTTGTGC ATTTATTCT

>Anisodevonia_ohshimai
TAGTCATATG CTTGTCTCAA AGATTAAGCC ATGCATGTCT AAGTACACGC CAGATACAAG
GTGAAACTGC GAATGGCTCA TTAAATCAGT TATGGTTCCT TAGATCGTAC AATCCTACTT
GGATAACTGT GGCAATTCTA GAGCTAATAC ATGCATCAAA GCTCCGACCT CCGGGGAAGA
GCGCTTTTGT TAGCAAAACC AATCCGGCCG GTTGTTGACT CTGAACAACT TTGTGCCGAT
CGCACGGCCT TGCGCCGGCG ACGTATCTTT CGAATGTCTG CCCTATCAAC TGTCGATGGT
ACGTGCTATG CCTACCATGG TTGTAACGGG TAACGGGGAA TCAGGGTTCG ATTCCGGAGA
GGGAGCATGA GAAACGGCTA CCACATCCAA GGAAGGCAGC AGGCGCGCAA ATTACCCAAT
CCCGACACGG GGAGGTAGTG ACGAAAAATA ACAATACGGG ACTCTTTCGA GGCCCCGTAA
TTGGAATGAG TACACTTTAA ATCCTTTAAC GAGGATCCAT TGGAGGGCAA GTCTGGTGCC
AGCAGCCGCG GTAATTCCAG CTCCAATAGC GTATATTAAA GTTGCTGCAG TTAAAAAGCT
CGTAGTTGGA TCTCGGGTGC AGGCCTGCGG TCCGCCTCGC GGCGGCCGCT CGTCCTGGCA
GCCTAGCTGC GGTGTCCCTT GGTGCTCTTG GTTGAGTGTC GGCGGCCGGA ACGTTTACTT
TGATGAAATT AGAGTGTTCA AAGCGTGGGC GCTTGCCCGC ATAATGGTGC ATGGAATGAT
GGAATAGGAC CTCGGTTCTA TTTTGTTGGT TTTCGGAGAG GTAATGATTG AGAGGGACAG
ACGGGGGCAT TCGTATTGCG GCGTTAGAGG TGAAATTCTT GGATCGCCGC AAGACGGACG
AGAGCGAAAG CATTTGCCAA GAATGTTTTC CTTAATCAAG AACGAAAGTC AGAGGCTCGA
AGACGATCAG ATACCGTCGT AGTTCTGACC ATAAACCATG CCAACTGGCA ATCCGCCGGA
GTTACTACAA TGACTCGGCA GGCAGCCCCC GGGAAACCAA AGTTTCTGGG TTCCGGGGGG
AGTATGGTTG CAAAGCTGAA ACTTAAAGGA ATTGACGGAA GGGCACCACC AGGAGTGGAG
CCTGTGGCTT AATTTGACTC AACACGGGAA ACCTCACCCG GCCCGGACAC TGTAAGGATT
GACAGATTGA GAGCTCTTTC TTGATTCGGT GGGTGGTGGT GCATGGCCGT TCTTAGTTGG
TGGAGCGATT TGTCTGGTTA ATTCCGATAA CGAACGAGAC TCCGGCCTGC TAAATAGTTC

GGGGCTATAT AGCCCCGCAA CTTCTTAGAG GGACAGGTGG CGTATAGCCA CACGAGATTG
AGCAATAACA GGTCTGTGAT GCCCTTAGAT GTTCGGGGCC GCACACGCGC TACATTGAAT
GGATCAACGT GCGTCTAGCC TTGCCCGAAA GGGCTGGGAA ACCCGTTGAA ACCCATTCGT
GATAGGGATT GGGGCTTGCA ATTGTTTCCC ATGAACGAGG AATTCCTAGT AAGCGCGAGT
CATCAGCTCG CGTTGATTGC GTCCCTGCCC TTTGTACACA CCGCCCGTCG CTACTACCGA
TCGCTCCAGT TAATGAACGC TTCGGATTGG TTAAGCGGGT TTCGGCCCGC TCGCGTGCCG
AGAAGATGCG TGAATTATCC GGGGTAGAGG TCGTAAAAGT CGTAACAAGG TATTCATTAA
TCAGCGGAGG AAAAGAAACT AACTAGGATT CCCCTAGTAA CGGCGAGTGA AGCGGGAAGA
GCCCAGCACC GAATCCCCCA GCGTCTGGCC GCTGACGGGA CATGTGGTGT TAGCGGGAGC
CCATCGTTGT CGCGTTCCGC CGCCAAAGTC CACCTGATCG TGGCATCCCA GAGCGGGTGA
GAGGCCCGTG ACGGCGGCGG GCGCGTTCGA CTCTCTCCAG GAGTCGGGTT GTTTGAGAAT
GCAGCCCAAA GCGGGTGGTA AACTCCATCT AAGGCTAAAT ACAGACACGA GTCCGATAGC
AAACAAGTAC CGTGAGGGAA AGTTGAAAAG AACTTTGAAG AGAGAGTTCA AGAGTACGTG
AAACCGTATA GAGTCAAACG GGTGGACCCG CGAGGTGTCG GCCCGGGGAA TTCAGCGGCG
CACTTTCTCC GACGAGAGCC ACGACCGGGT CGGCCACTCC GCGCGCGTCA AGGTGACCCA
CGCCCCTGGG AGCTTACAGG TCGCTGCGAT GGAGTCGGCA CG---CCGGC CCGAGGACGC
GCCGCGCGTC GGGCTCCGGA CCGTCCCGGT GCGTTCGACT T--TTTCGCA GTGTGCCGAG
ACCGCGCGCC GTTCCGGATG CAGGGGTCGG TGGCGAATCG GTCGGTACTC CACCCGACCC
GTCTTGAAAC ACGGACCAAG GAGTCTAACA TGTGCGCGAG TCACAGGGAC CCGAATCCCG
AAGGCGCAAT GAAAGTGAAG GTCGGCCTCG GCCGTCCGAG GCAGGATCCC GTCCCGGGCG
CACTGCCGGC CCGTCTCGAT AGCG------ ----AGCTCG CGCTCGTCTG CGAGGCGGAG
CAAGAGCGTA CACGTTGGTA CCCGAAAGAT GGTGAACTAT GCCTGAGCAG GACGAAGTCA
GAGGAAACTC TGATGGAGGT CCGCAGCGAT TCACGTAAAT CCACCGGAGG CAAGGCTCCC
CGCAAACAGC TGGCCACCAA GGCTGCTCGC AANAGCGCCC CGGCCACTGG CGGCGTGAAG
AAGCCCCACA GGTACAGGCC CGGAACCGTC GCCCTGAGAG AGATCAGGAG NTACCAGAAG
AGCACCGAGC TGCTCATCCG CAAGTTGCCC TTCCAGCGCC TGGTGAGAGA GATTGCTCAG
GACTTCAAGA CCGACCTGCG CTTCCAGAGC TCTGCCGTCA TGGCTCTGCA GGAGGCCAGC
GAAGCCTACC TGGTCGGCCT GTTCGAGGAC ACCAACCTGT GCGCCATTCA CGCCAAGCGT
AACTTTATAT TTTATTTTTG GAGTATGAGC TGGCTTAGTT GGAAGTTCTC TAAGGGTTTT
AATTCGAGTC GAATTATCTC GTCCGGGGAG ATTTTTGGGT GACGATCATT TATATAATGT
TGTTGTAACT GCTCATGCAT TTGTAATAAT TTTTTTCTTA GTAATGCCTA TAATGGTTGG
TGGATTTGGT AACTGGTTAG TTCCTTTAAT ACTTGTTTGT CCTGATATGG CGTTTCCTCG
GATGAATAAC ATGAGATTTT GGCTTTTGCC TCCTGCGTTA TCATTATTGT TGGGATCTTC
TTTTGTCGAA GGGGGAGTTG GTGCTGGGTG AACGGTTTAT CCACCTTTAG CAGGAAACGT
TAGTCATGGA GGTGGGTCAA TAGATTTTGC TATTTTTTCA CTTCATCTGG CTGGTGTTTC
ATCTATTTTA GGAGCTATTA ATTTTTTGGT TAGAATTGGA AATATGCGGT CAGAAATTAT
GGGATTAAAA CGTGTCACTT TATTTTGTTG ATCAATTGGA ATTACTGCTT TTCTTTTAGT
GGTAGCAATA CCAGTTTTAG CAGGTGCTAT TACTATGCTT TTATTTGATC GAAATTTTAA
TACTTCTTTT TTTGACCCTG CAGGAGGAGG AGATCCAGTT CTTTTTGTTC ATTTATTTT

>Arthritica_japonica
TAGTCATATG CTTGTCTCAA AGATTAAGCC ATGCATGTCT AAGTACACGC CAGATTCATG
GTGAAACTGC GAATGGCTCA TTAAATCAGT TATGGTTCCT TAGATCGTAC AATCCTACTT
GGATAACCGT GGCAATTCTA GAGCTAATAC ATGCGTCAAA GCTCCGACCT TCGGGGAAGA
GCGCTTTTGT TAGCAAAACC AATCCGGTCG GTTGTTGACT CTGAACAACT TTGTGCTGAT
CGCACGGCCT AGCGCCGGCG ATGTATCTTT CGAATGTCTG CCCTATCAAC TGTCGATGGT
ACGTGCTATG CCTACCATGG TTGTAACGGG TAACGGGGAA TCAGGGTTCG ATTCCGGAGA
GGGAGCATGA GAAACGGCTA CCACATCCAA GGAAGGCAGC AGGCGCGCAA ATTACCCAAT
CCCGACACGG GGAGGTAGTG ACGAAAAATA ACAATACGGG ACTCTTTCGA GGCCCCGTAA
TTGGAATGAG TACACTTTAA ATCCTTTAAC GAGGATCCAT TGGAGGGCAA GTCTGGTGCC

AGCAGCCGCG GTAATTCCAG CTCCAATAGC GTATATTAAA GTTGTTGCAG TTAAAAAGCT
CGTAGTTGGA TCTCGGGTGC AGGCTTGCGG TCCGCCTCGC GGCGGCTGCT CGTCCTGGCA
GCCTAGCTGC GGTATCCCTT GGTGCTCTTG ACTGAGTGTC GGTGGCCGGA ACGTTTACTT
TGATGAAATT AGAGTGCTCA AAGCGAGGGC GATTGCCCGT ATAATGGTGC ATGGAATGAT
AGAATAGGAC CTCGGTTCTA TTTTGTTGGT TTTCGGAGAG GTAATGATTG AGAGGGACAG
ACGGGGGCAT TCGTATTGCG GCGTTAGAGG TGAAATTCTT GGATCGCCGC AAGACGGACT
ACAGCGAAAG CATTTGCCAA GAATGTTTTC CTTAATCAAG AACGAAAGTC AGAGGCTCGA
AGACGATCAG ATACCGTCGT AGTTCTGACC ATAAACCATG CCAACTGGCA ATCCGCCGGA
GTTACTACAA TGACTCGGCG AGCAGCCTCC GGGAAACCTA AGTTTCTGGG TTCCGGGGGG
AGTATGGTTG CAAAGCTGAA ACTTAAAGGA ATTGACGGAA GGGCACCACC AGGAGTGGAG
CCTGTGGCTT AATTTGACTC AACACGGGAA ACCTCACCCG GCCCGGACAC CGTAAGGATT
GACAGATTGA GAGCTCTTTC TTGATTCGGT GGGTGGTGGT GCATGGCCGT TCTTAGTTGG
TGGAGCGATT TGTCTGGTTA ATTCCGATAA CGAACGAGAC TCTAGCCTAC TAAATAGTTC
GAGGATATAT ATATTCGTAA CTTCTTAGAG GGACAGGTGG CGTATAGCCA CACGAGATTG
AGCAATAACA GGTCTGTGAT GCCCTTAGAT GTCCGGGGCC GCACACGCGC TACATTGAGT
GGATCAGCGT GCGTCTAGCC TTGCCCGAGA GGGCTGGGAA ACCCGTTGAA ACCCACTCGT
GATAGGGACT GGGGCTTGCA ATTATTTCCC ATGAACGAGG AATTCCTAGT AAGTGCGAGT
CATCAGCTCG CGCTGATTGC GTCCCTGCCC TTTGTACACA CCGCCCGTCG CTACTACCGA
TCGCTCCAGT TAATGAACGC CTCGGATTGG TTAAGCGGGT TTCGGCCTGC TCGCGTGCCG
AGAAGACGTG TAAATTATCT GGAGTAGAGG GAGTAAAAGT CGTAACAAGG TATCCATCAA
TAAGCGGAGG AAAAGAGACT AACAAGGATT CCCTTAGTAA CGGCGAGTGA AGCGGGAAGA
GCCCAGCACC GAATCCCCCA GCGTCTGGCC GCTGACGGGA CATGTGGTGT TAGCGGGAGC
CTTTTGTCGG CGTGCCTCGG CACCAAAGTC CACCTGATCG TGGCTACCCA GGGCGGGTGA
GAGGCCCGTT CCGGTGTCGA GCGCGTCTGA CTCTCTCCAG GAGTCGGGTT GTTTGAGAAT
GCAGCCCAAA GTGGGTGGTA AACTCCATCT AAGGCTAAAT ACTGACACGA GTCCGATAGA
GGACAAGTAC CGTGAGGGAA AGTTGAAAAG AACTTTGAAG AGAGAGTTCA AGAGTACGTG
AAACCGCATA GAGTCAAACG GGTGGATCCG CAAGGTGTTG CCCCGGGGAA TTCAGCGGTG
CACTTTCTCC GGCGAGAGCC ACGACCGGGT TCGCTGCTCG GACTGACCGA AGGTTTCCAG
GCGGCCTGGG AGCTTACAGC GGTCCGTCGT CGAGTGTGCG TGTCGCGAGC CCGAGGACGC
GCCGCGCGTC GGGTTTCGGG TCGTCTTCGG GCGTTCGACT GTCTGCGGCA GTGCGTCGCG
ACCGCGCTCG GTCCCGT-CG CTAGGGTCGG TGGCGAATCA GTCGGTACTC CACCCGACCC
GTCTTGAAAC ACGGACCAAG GAGTCTAACA TGTGCGCGAG TCATAGGGAC GCGAATCCCG
AAGGCGCAAT GAAAGTGAAG GTCGGCTTCG GCCGTCCGAG GCAGGATCCC GTCG-GGGCG
CACTGCCGGC CCGTTTCGAT AGCGTCCCG- --GCG-CCGG TG-ACGTCTG CGAGGCGGAG
CAAGAGCGTA CACGTTGGTA CCCGAAAGAT GGTGAACTAT GCCTGAGCAG GACGAAGTCA
GAGGAAACTC TGATGGAGGT CCGCAGCGAT TCCAGAAAGT CCACTGGTGG GAAGGCCCCC
AGGAAGCAGC TTGCAACCAA GGCTGCAAGG AAATCCGCAC CAGCTACCGG TGGGGTAAAG
AAGCCGCACA GGTACAGGCC CGGAACCGTG GCTCTCCGTG AGATCAGGAG ATACCAGAAG
AGCACCGAGC TTCTCATCAG GAAACTGCCA TTCCAGCGCC TGGTCCGTGA AATCGCCCAG
GACTTCAAGA CCGATCTGCG CTTCCAGAGC TCGGCAGTCA TGGCTCTGCA GGAGGCCAGC
GAGGCTTACT TGGTCGGACT GTTTGAGGAC ACCAACTTGT GCGCCATCCA CGCCAAGCGT
GACGTTGTAC TTTTTGTTTG GTATTTGGTC TGGTTTGGTG GGCACATCTT TTAGAGTTTT
AATTCGATTA GAATTATCCC GTCCGGGTGC ATTTTTAGGA GATGATCACT TGTATAATGT
TATTGTTACG GCTCATGCGT TTGTTATAAT TTTTTTTCTT GTTATACCTA TGATAGTTGG
CGGGTTCGGT AATTGGTTAG TTCCTTTAAT ATTAACTTCT CCTGATATAG CTTTCCCTCG
GATGAATAAT ATAAGATTTT GGCTCTTACC GCCGGCTTTG TTTTTACTTC TTAGGTCTGC
GTTTGTTGAA AGCGGTGTAG GTACGGGTTG AACTGTTTAT CCTCCTCTAT CTGCTAATAT
TGCCCATAGT GGTGGTTCTG TAGATTATGG GATTTTTTCT TTGCATTTAG CTGGTGTTTC
ATCAATTTTA GGTGCTATTA ATTTTTTAGC TAGGACAATT AACATACGTC CGGAGATTAT
AGAATTTAAG CGAGTAACAT TATTTGTATG GTCTATTGCC ATTACAGCTT TTTTATTAGT

AGTAGCTATG CCAGTTTTAG CAGGGGCTAT TACAATATTA TTGACTGATC GAAATTTCAA
TACTTCG--- ---------- ---------- ---------- ---------- ---------

>Byssobornia_yamakawai
TAGTCATATG CTTGTCTCAA AGATTAAGCC ATGCATGTCT AAGTACACGC CAGATACATG
GTGAAACTGC GAATGGCTCA TTAAATCAGT TATGGTTCCT TAGATCGTAC AATCCTACTT
GGATAACTGT GGCAATTCTA GAGCTAATAC ATGCGTCAAA GCTCCGACCT CCGGGGAAGA
GCGCTTTTGT TAGCAAAACC AATCCGGTCG GTTGTTGACT CTGGACAACT T--TGCCGAT
CGCACGGCCT AGCGCCGGCG ACGTATCTTT TGAATGTCTG CCCTATCAAC TGACGATGGT
ACGTGCTATG CCTACCATGG TTGTAACGGG TAACGGGGAA TCAGGGTTCG ATTCCGGAGA
GGGAGCATGA GAAACGGCTA CCACATCTAA GGAAGGCAGC AGGCGCGCAA ATTACCCAAT
CCCGACACGG GGAGGTAGTG ACGAAAAATA ACAATACGGG ACTCTTTCGA GGCCCCGTAA
TTGGAATGAG TACACTTTAA ATCCTTTAAC GAGGATCCAT TGGAGGGCAA GTCTGGTGCC
AGCAGCCGCG GTAATTCCAG CTCCAATAGC GTATATTAAA GTTGCTGTAG TTAAAAAGCT
CGTAGTTGGA TCTCGGGTGC AGGCTTGCGG TCCGCCTCGC GGCGGCTGCT CGTCCTAGCA
GCCTAGCTTC GGTGTCCCTT GGTGCTCTTG ACCGAGTGTC GGTGGCCGGA ACGTTTACTT
TGATGAAATT AGAGTGTTCA GAGCGCGGGC GTTTGCCCGT ATAATGGTGC ATGGAATGAT
AGAATAGGAC CTCGGTTCTA TTTTGTTGGT TTTCGGAGAG GTAATGATCA AGAGGGACAG
ACGGGGGCAT TCGTATTGCA GCGTTAGAGG TGAAATTCTT GGATCGTTGC AAGACGGACG
ACAGCGAAAG CATTTGCCAA GAATGTTTTC CTTAATCAAG AACGAAAGTC AGAGGCTCGA
AGACGATCAG ATACCGTCGT AGTTCTGACC ATAAACCATG CCAACTGGCA ATCCGCCGGA
GTTACTACAA TGACTCGGCG AGCAGCCCCC GGGAAACCAA AGTTTCTGGG TTCCGGGGGG
AGTATGGTTG CAAAGCTGAA ACTTAAAGGA ATTGACGGAA GGGCACCACC AGGAGTGGAG
CCTGTGGCTT AATTTGACTC AACACGGGAA ACCTCACCCG GCCCGGACAC CGTTAGGATT
GACAGATTGA GAGCTCTTTC TTGATTCGGT GGGTGGTGGT GCATGGCCGT TCTTAGTTGG
TGGAGCGATT TGTCTGGTTA ATTCCGATAA CGAACGAGAC TCTAGCCTAC TAAATAGTTC
GAGGATATAT TACCTCGCAA CTTCTTAGAG GGACAGGTGG CGTATAGCCA CACGAGATTG
AGCAATAACA GGTCTGTGAT GCCCTTAGAT GTTCGGGGCC GCACACGCGC TACATTGAAT
GGATCAACGT GCGTCTAGCC TTGCCCGAAA GGGCTGGGAA ACCCGTTGAA CCCCATTCGT
GATAGGGATT GGGGCTTGCA ATTATTTCCC ATGAACGAGG AATTCCTAGT AAGCGCGAGT
CATCAGCTCG CGTTGATTGC GTCCCTGCCC TTTGTACACA CCGCCCGTCG CTACTACCGA
TCGTTCCAGT TAATGAACGC CTCGGATTGG TTAAGCGGG- -GAAACCTGC TCGCGTGCCG
AGAAGAAGTG TAAGTTATCT GGGATAGAGG TCGTAAAAGT CGTAACAAGG TATCCATTAA
TAAGCGGAGG AAAAGAGACT AACTAGGATT CCCCTAGTAA CGGCGAGTGA AGCGGGAAGA
GCCCAGCACC GAATCCCCCG GCGTCTGGCC GCCGACGGGA CATGTGGTGT TAGCGGGAGT
CTTTTGTCGG CGCGTTCTGG CACCAAAGTC CACCGGGTCG TGGCAATCCA GTGCGGGTGA
GAGGCCCGTT CCGGTGCCGG TCGCGTTGGA CTCTCTCCAG GAGTCGGGTT GTTTGAGAAT
GCAGCCCAAA GTGGGTGGTA AACTCCATCT AAGGCTAAAT ACAGACACGA GTCCGATAGA
GGACAAGTAC CGTGAGGGAA AGTTGAAAAG AACTTTGAAG AGAGAGTTCA AGAGTACGTG
AAACCGCATA GAGTCAAACG GGTGGATCCG CAAGGTGTTG GACCGGGGAA TTCAGCGGTG
CACTTTCTCC GACGAGAGCC ACGACCGGGT TCGCTGTCCG CGGTGCGGGA AGGTTTCCCG
GCGCCT-GGG AGT-TACAGC CCGTCGGCCC TGGACATCCA TGTCGCGGAC CCGAGGACGC
GCCGCGCGTC GGGTCTCGGG TCGTCCTCGT GCGTTCGACT TTCGTCGGTA GTTCTTCGAG
ACCGCGCGCT GGCTCGGTGG CCAGGGTCAG TGGCGAATCA GTCGGTACTC CACCCGACCC
GTCTTGAAAC ACGGACCAAG GAGTCTAACA TGTGCGCGAG TCGTAGGGAC GCGAATCCCG
AAGGCGCAAT GAAAGTGAAG GCCGCCTACG GTTGGCCGAG GCAGGATCCC GTCGCGGGCG
CACTGCCGGC CCGTCTCGAT AGCGGCGTAG GCGTGCCCAA CGTTCGTCTA CGAGGCGGAG
CAAGAGCGTA CACGTTGGTA CCCGAAAGAT GGTGAACTAT GCCTGAGCAG GACGAAGTCA
GAGGAAACTC TGATGGAGGT CCGCAGCGAT TCTAGAAAGT CAACTGGAGG GAAAGCCCCC
AGGAAACAGT TGGCCACCAA GGCCGCACGT AAGAGTGCAC CGGCCACCGG TGGTGTGAAG

AAGCCACACA GATACAGGCC CGGAACCGTT GCCCTCCGTG AAATCAGACG ATACCAGAAG
AGCACTGAAC TGCTCATCAG AAAATTGCCC TTCCAGCGTC TCGTCCGTGA GATCGCCCAG
GACTTCAAGA CTGACCTGCG ATTCCAGAGC TCTGCTGTCA TGGCACTGCA GGAGGCCAGC
GAGGCTTACT TGGTCGGACT TTTCGAGGAC ACCAACTTGT GCGCTATCCA CGCCAAGCGT
---------- ---------- ---------- ---------- -----TTCGT TCAGAGTTTT
GATTCGTTTA GAGCTCTCCC GTCCTGGTGC TTTTCTAGGG GATGATCATC TTTATAATGT
GATCGTGACT GCTCATGCTT TCGTAATGAT TTTTTTTTTA GTTATACCCA TAATGGTTGG
GGGGTTTGGT AATTGGTTAG TGCCTTTGAT GCTAACTTCT CCANATATGG CTTTTCCGCG
GATGAACAAT ATGAGTTTTT GATTATTGCC TCCTGCTTTG TTTTTGTTGT TGAGCTCTGC
TTTTGTANAG AGTGGAGTTG GTACCGGGTG AACGGTCTAT CCTCCTTTGT CAGGTAATGT
GACTCATAGA GGGGGTTCAG TTGATTATGG AATTTTTTCT CTTCATTTAG CTGGTGTTTC
CTCTATTTTA GGTGCGATTA ATTTTTTGGC AACAATAGTA AATATGCGTC CTGAAATTAT
AGAATTGAAG CGTGTTACGC TTTTCGTTTG ATCTATTGGT ATCACGGCAT TTTTATTAGT
TGTGGCTATA CCCGTTTTAG CTGGTGCTAT TACNATGTTG TTGACTGATC GAAACTTTAA
TACTTCG--- ---------- ---------- ---------- ---------- ---------

>Curvemysella_paula
TAGTCATATG CTTGTCTCAA AGATTAAGCC ATGCATGTCT AAGTACACGC CAGATTAATG
GTGAAACTGC GAATGGCTCA TTAAATCAGT TATGGTTCCT TAGATCGTAC AATCCTACTT
GGATAACTGT GGCAATTCTA GAGCTAATAC ATGCGTCAAA GCTCCGACCT TCGGGGAAGA
GCGCTTTTGT TAGCAAGACC AATCCGGTCG GTTGTTGACT CTGAACAACT TTGTGCTGAT
CGCACGGCCT AGCGCCGGCG ACGTATCTTT CGAATGTCTG CCCTATCAAC TGACGATGGT
ACGTGCTATG CCTACCATGG TTGTAACGGG TAACGGGGAA TCAGGGTTCG ATTCCGGAGA
GGGAGCATGA GAAACGGCTA CCACATCCAA GGAAGGCAGC AGGCGCGCAA ATTACCCAAT
CCCGACACGG GGAGGTAGTG ACGAAAAATA ACAATACGGG ACTCTTTCGA GGCCCCGTAA
TTGGAATGAG TACACTTTAA ATCCTTTAAC GAGGATCCAT TGGAGGGCAA GTCTGGTGCC
AGCAGCCGCG GTAATTCCAG CTCCAATAGC GTATATTAAA GTTGCTGCAG TTAAAAAGCT
CGTAGTTGGA TCTCGGGTGT AGGCTTGCGG TCCGCCTCGC GGCGGCTGCT CGTCCTGACA
GCCTAGCTTC GGTGTCCCTT GGTGCTCTTG ATTGAGTGTC GGTGGCCGGA ACGTTTACTT
TGATGAAATT AGAGTGTTTA AAGCGTGGGC GTTTGCCCGT ATAATGGTGC ATGGAATGAT
AGAATAGGAC CTCGGTTCTA TTTTGTTGGT TTTCGGAGAG GTAATGATTG AGAGGGACAG
ACGGGGGCAT TCGTATTGCA GCGTTAGAGG TGAAATTCTT GGATCGTTGC AAGACGGCCG
ACAGCGAAAG CATTTGCCAA GAATGTTTTC CTTAATCAAG AACGAAAGTC AGAGGCTCGA
AGACGATCAG ATACCGTCGT AGTTCTGACC ATAAACTATG CCAACTGGCA ATCCGCCGGA
GTTACTACAA TGACTCGGCG AGCAGCCCCC GGGAAACCAA AGTTTCTCGG TTCCGGGGGG
AGTATGGTTG CAAAGCTGAA ACTTAAAGGA ATTGACGGAA GGGCACCACC AGGAGTGGAG
CCTGTGGCTT AATTTGACTC AACACGGGAA ACCTCACCCG GCCCGGACAC CGTTAGGATT
GACAGATTGA GAGCTCTTTC TTGATTCGGT GGGTGGTGGT GCATGGCCGT TCTTAGTTGG
TGGAGCGATT TGTCTGGTTA ATTCCGATAA CGAACGAGAC TCTAGCCTAC TAAATAGTTC
GAGGATATAC AACCTCGCAA CTTCTTAGAG GGACAGGTGG CGTTTAGCCA CACGAGATTG
AGCAATAACA GGTCTGTGAT GCCCTTAGAT GTCCGGGGCC GCACACGCGC TACATTGAAT
GGATCAACGT GCGTCTAGCC TTGCCCGAAA GGGCTGGGAA ACCCGTTGAA CCCCATTCGT
GATAGGGACT GGGGCTTGCA ATTATTTCCC ATGAACGAGG AATTCCTAGT AAGCGCGAGT
CATCAGCTCG CGTTGATTGC GTCCCTGCCC TTTGTACACA CCGCCCGTCG CTACTACCGA
TCGTTCCAGT TAATGAACAC TTCGGATTGG TTAAGCGGGT TTCGGCCTGC TCGCGTGCCG
AGAAGATTTG TGAATTATCT GGGATAGAGG TCGTAAAAGT CGTAACAAGG TATCCATTAA
TAAGCGGAGG AAAAGAGACT AACTAGGATT CCCCTAGTAA CGGCGAGTGA AGCGGGAAGA
GCCCAGCACC GAATCCCCCG GCGTCTGGCT GCCGACGGGA CATGTGGTGT TAGTGGGAGC
TTTGTGTCGG CGTGTTTCGG CACCAAAGTC CACTTGATCG TGGCAATCCA TGGCGGGTGA
GAGACCCGTT CCGGTGTCGA ACGCGTCGGA CTCTCTCCAG GAGTCGGGTT GTTTGAGAAT

GCAGCCCAAA GTGGGTGGTA AACTCCATCT AAGGCTAAAT ACTGACACGA GTCCGATAGA
GGACAAGTAC CGTGAGGGAA AGTTGAAAAG AACTTTGAAG AGAGAGTTCA AGAGTACGTG
AAACCGCATA GAGTCAAACG GGTGGATCCG CAAGGTGTTG GCCCGGGGAA TTCAGCGGTG
CACTTTCTCC GACGAGAGCC ACGACCGGGT TCGCTGCTCG CAGTGCGAGA AGGTTTCCCC
GCGTGCTGGG AGCATACAGC TCGTCGGCCT CGAGTCTATA TGTGGCGGAC CCGAGGACGC
GCCGCGCGTC CGGCTCCGGG CCGTCCGCGT GCGTTCGACC T-TCCTCGTA GAGTGCCGTA
ACCGCGTGCG GGCTCGGGTG CTAGGGTCGG TGGCGAATCA GTCGGTACTC CACCCGACCC
GTCTTGAAAC ACGGACCAAG GAGTCTAACA TGTGCGCGAG TCATAGGGAC CCGAATCCCG
AAGGCGCAAT GAAAGTGAAG GCCGCCTACG GCTGGCCGAG GCAGGATCCC GTCGCGGGCG
CACTGCCGGC CCGTCTCGAT AGCGGTGTAG CA-GTGCCAA CATTCGTCTA CGAGGCGGAG
CAAGAGCGTA CACGTTGGTA CCCGAAAGAT GGTGAACTAT GCCTGAGCAG GACGAAGTCA
GAGGAAACTC TGATGGAGGT CCGCAGCGAT TCTAGAAAAT CCACTGGTGG CAAGGCCCCA
CGAAAACAGC TGGCCACCAA GGCCGCACGT AAGAGTGCGC CCGCCACTGG TGGTGTGAAG
AAACCACACA GATACAGGCC CGGAACCGTC GCTCTCCGTG AGATCAGAAG GTACCAGAAG
AGCACCGAGC TCCTGATCAG AAAATTGCCC TTCCAGCGTC TCGTCCGTGA GATCGCCCAG
GACTTCAAGA CTGATCTCCG ATTCCAGAGC TCCGCCGTCA TGGCTCTGCA GGAGGCCAGC
GAGGCTTACC TCGTCGGTCT TTTCGAGGAC ACCAACTTGT GCGCTATCCA CGCCAAGCGT
TACTTTATAT TTCATTTTTG GAATTTGGTC CGGGATGGTT GGAACTTCTT TTAGGGTATT
AATTCGACTA GAGTTATCAC GTCCCGGAGC ATTTCTTGGA GATGACCATC TTTACAATGT
GATTGTAACA GCCCATGCAT TTGTTATAAT TTTTTTCTTA GTAATACCTA TAATGGTGGG
AGGATTTGGT AATTGATTAG TGCCTTTAAT ACTAACTTCG CCTGATATGG CTTTTCCACG
AATAAATAAT ATAAGATTTT GGCTTTTACC TCCAGCCTTA TTTCTTCTTT TAAGGTCAGC
TTTTGTAGAA AGTGGTGTGG GCACTGGGTG AACTGTCTAT CCCCCATTAT CAAGAAATGT
TACTCATAGA GGAGGGTCGG TTGATTACGG GATTTTTTCT CTTCATTTAG CTGGTGTTTC
TTCTATTTTA GGTGCTATTA ATTTTTTGGC AACTACTGTT AACATACGGC CTGAAATTAT
AGAGCTAAAA CGAGTTACTT TATTTGTTTG ATCTATTGCA ATTACTGCTT TCTTATTAGT
TGTAGCAATA CCTGTTTTGG CTGGTGCTAT TACTATATTA CTAACAGATC GTAATTTTAA
TACATCTTTT TTTGACCCGT CAGGCGGGGG GGATCCAATT TTATTTGTTC ATTTGTTCT

>Devonia_semperi
TAGTCATATG CTTGTCTCAA AGATTAAGCC ATGCATGTCT AAGTACACGC CAGATACAAG
GTGAAACTGC GAATGGCTCA TTAAATCAGT TATGGTTCCT TAGATCGTAC AATCCTACTT
GGATAACTGT GGCAATTCTA GAGCTAATAC ATGCATCAAA GCTCCGACCT CCGGGGAAGA
GCGCTTTTGT TAGCAAAACC AATCCGGCCG GTTGTTGACT CTGAACAACT TTGTGCCGAT
CGCACGGCCT TGCGCCGGCG ACGTATCTTT CGAATGTCTG CCCTATCAAC TGTCGATGGT
ACGTGCTATG CCTACCATGG TTGTAACGGG TAACGGGGAA TCAGGGTTCG ATTCCGGAGA
GGGAGCATGA GAAACGGCTA CCACATCCAA GGAAGGCAGC AGGCGCGCAA ATTACCCAAT
CCCGACACGG GGAGGTAGTG ACGAAAAATA ACAATACGGG ACTCTTTTGA GGCCCCGTAA
TTGGAATGAG TACACTTTAA ATCCTTTAAC GAGGATCCAT TGGAGGGCAA GTCTGGTGCC
AGCAGCCGCG GTAATTCCAG CTCCAATAGC GTATATTAAA GTTGCTGCAG TTAAAAAGCT
CGTAGTTGGA TCTCGGGTGC AGGCCTGCGG TCCGCCTCGC GGCGGCTGCT CGTCCTGGCA
GCCTAGCTGC GGCGTCCCTT GGTGCTCTTG ATTGAGTGTC GGCGGCCGGA ACGTTTACTT
TGATGAAATT AGAGTGTTCA AAGCGTGGGC GCTTGCCCGC ATAATGGTGC ATGGAATGAT
GGAATAGGAC CTCGGTTCTA TTTTGTTGGT TTTCGGAGAG GTAATGATTG AGAGGGACAG
ACGGGGGCAT TCGTATTGCG GCGTTAGAGG TGAAATTCTT GGATCGTCGC AAGACGGACG
AGAGCGAAAG CATTTGCCAA GAATGTTTTC CTTAATCAAG AACGAAAGTC AGAGGCTCGA
AGACGATCAG ATACCGTCGT AGTTCTGACC ATAAACCATG CCAACTGGCA ATCCGCCGGA
GTTACTACAA TGACTCGGCA GGCAGCCCCC GGGAAACCAA AGTTTCTGGG TTCCGGGGGG
AGTATGGTTG CAAAGCTGAA ACTTAAAGGA ATTGACGGAA GGGCACCACC AGGAGTGGAG
CCTGTGGCTT AATTTGACTC AACACGGGAA ACCTCACCCG GCCCGGACAC TGTAAGGATT

GACAGATTGA GAGCTCTTTC TTGATTCGGT GGGTGGTGGT GCATGGCCGT TCTTAGTTGG
TGGAGCGATT TGTCTGGTTA ATTCCGATAA CGAACGAGAC TCTGGCCTGC TAAATAGTTC
GGGGCTATAT AGCCCCGCAA CTTCTTAGAG GGACAGGTGG CGTATAGCCA CACGAGATTG
AGCAATAACA GGTCTGTGAT GCCCTTAGAT GTTCGGGGCC GCACACGCGC TACATTGAAT
GGATCAACGT GCGTCTAGCC TTGCCCGGAA GGGCTGGGAA ACCCGTTGAA ACCCATTCGT
GATAGGGATT GGGGCTTGCA ATTGTTTCCC ATGAACGAGG AATTCCTAGT AAGCGCGAGT
CATCAGCTCG CGTTGATTGC GTCCCTGCCC TTTGTACACA CCGCCCGTCG CTACTACCGA
TCGCTCCAGT TAATGAACGC TTCGGATTGG TTAAGCGGGT TTCGGCCCGC TCGCGTGCCG
AGAAGATGTG TGAATTATCC GGGGTAGAGG TCGTAAAAGT CGTAACAAGG TATTCATTAA
TCAGCGGAGG AAAAGAAACT AACTAGGATT CCCCTAGTAA CGGCGAGTGA AGCGGGAAGA
GCCCAGCACC GAATCCCCCA GCGTCTGGTC GCTGACGGGA AATGTGGTGT TAGCGGGAGC
CCATCGTCGT CGCGTCGCGC CGCCCAAGTC CACCTGATCG TGGCATCCCA GAGCGGGTGA
GAGGCCCGTT ACGGCGGGGC GCGCGTCTGA CTCTCTCCAG GAGTCGGGTT GTTTGAGAAT
GCAGCCCAAA GCGGGTGGTA AACTCCATCT AAGGCTAAAT ACAGACACGA GTCCGATAGC
AAACAAGTAC CGTGAGGGAA AGTTGAAAAG AACTTTGAAG AGAGAGTTCA AGAGTACGTG
AAACCGTATA GAGTCAAACG GGTGGACCCG CGAGGTGTCG GACCGGGGAA TTCAGCGGCG
CACTTTCTCC GACGAGTGCC ACGACCGGGT CCGCTACTCC GCGCGCGTCA AGGTGACCCG
G--CCCTGGG AGCATACAGG TCGCTGCGTC GGAGTCGGCA C---GCGGGC CCGAGGACGC
GCCGCGCGTC CGGCTCCGGA CCGTCCCGGT GCGTTCGACT T--TTTCGCA GTGTGCCGAG
ACTGCGCGTC GTTCCGGATG CAGGGGTCGG TGGCGAATCG GTCGGTACTC CACCCGACCC
GTCTTGAAAC ACGGACCAAG GAGTCTAACA TGTGCGCGAG TCACAGGGAC CCGAATCCCG
AAGGCGCAAT GAAAGTGAAG GTCGGCCTTG GCCGTCCGAG GCAGGATCCC GTCCCGGGCG
CACTGCCGGC CCGTCTCGAT AGCGAGTCT- ---------- --CTCGTCTG CGAGGCGGAG
CAAGAGCGTA CACGTTGGTA CCCGAAAGAT GGTGAACTAT GCCTGAGCAG GACGAAGTCA
GAGGAAACTC TGATGGAGGT CCGCAGCGAT TCCCGTAAAT CCACCGGAGG AAAGGCTCCC
AGGAAACAGC TGGCCANTAA GGCAGCCAGA AAGAGTGCCC CGGCCACCGG CGGCGTGAAG
AAGCCCCACA GGTACAGGCC CGGAACTGTC GCCNTGAGAG AGATCAGGAG ATACCAGAAG
AGCACCGAGC TTCTCATCCG CAAGCTGCCA TTCCAGCGCC TGGTGAGAGA AATTGCCCAG
GACTTCAAGA CCGACCTGCG CTTCCAGAGC TCTGCCGTTA TGGCTCTGCA GGAGGCCAGC
GAAGCTTACC TGGTCGGCCT GTTCGAGGAC ACCAACCTGT GCGCTATCCA CGCCAAGCGT
GACTTTATAT TTTATTTTTG GAGTATGAGC TGGGTTAGTT GGAAGATCTT TAAGAGTTTT
AATTCGGGTG GAATTATCTC GACCTGGAAC TTTTTTAGGT GATGACCATC TTTATAATGT
TGTTGTTACT GCCCATGCTT TTGTAATAAT TTTTTTTTTA GTAATACCTA TAATAGTTGG
TGGTTTCGGA AATTGGTTGG TACCTTTAAT ATTGGTTTGT CCGGATATAG CTTTTCCTCG
GATGAACAAT ATAAGATTTT GACTTCTTCC TCCGGCTTTG TCATTATTAT TGGGTTCTTC
TTTTGTAGAG GGGGGAGTAG GTGCTGGTTG AACTGTTTAT CCTCCTTTGT CTGGAAATAT
TAGTCATGGG GGAGGTTCTA TAGATTTTGC AATTTTTTCT CTTCACTTAG CTGGTGTTTC
TTCAATTTTG GGAGCTATTA ACTTTTTGGT GAGAATTGGT AATATGCGTT CTGATATTAT
GGGATTAAAG CGAGTGAGAT TATTTTGTTG GTCAATTGGA ATTACTGCTT TTCTTTTGGT
TGTAGCCATG CCTGTTTTGG CTGGGGCTAT TACTATGCTT TTGTTTGATC GTAATTTTAA
TACTTCTTTT TTTGATCCTG CGGGTGGGGG TGATCCAATT TTATTTATTC A--------

>Entovalva_lessonothuriae
TAGTCATATG CTTGTCTCAA AGATTAAGCC ATGCATGTCT AAGTACACGC CAGATACAAG
GTGAAACTGC GAATGGCTCA TTAAATCAGT TATGGTTCCT TAGATCGTAC AATCCTACTT
GGATAACTGT GGCAATTCTA GAGCTAATAC ATGCATCAAA GCTCCGACCT CCGGGGAAGA
GCGCTTTTGT TAGCAAAACC AATCCGGCCG GTTGTTGACT CTGAACAACT TTGTGCCGAT
CGCACGGCCT TGCGCCGGCG ACGTATCTTT CGAATGTCTG CCCTATCAAC TGTCGATGGT
ACGTGCTATG CCTACCATGG TTGTAACGGG TAACGGGGAA TCAGGGTTCG ATTCCGGAGA
GGGAGCATGA GAAACGGCTA CCACATCCAA GGAAGGCAGC AGGCGCGCAA ATTACCCAAT

CCCGACACGG GGAGGTAGTG ACGAAAAATA ACAATACGGG ACTCTTTCGA GGCCCCGTAA
TTGGAATGAG TACACTTTAA ATCCTTTAAC GAGGATCCAT TGGAGGGCAA GTCTGGTGCC
AGCAGCCGCG GTAATTCCAG CTCCAATAGC GTATATTAAA GTTGCTGCAG TTAAAAAGCT
CGTAGTTGGA TCTCGGGTGC AGGCCTGCGG TCCGCCTCGC GGCGGCCGCT CGTCCTGGCA
GCCTAGCTGC GGTGTCCCTT GGTGCTCTTG GTTGAGTGTC GGCGGCCGGA ACGTTTACTT
TGATGAAATT AGAGTGTTCA AAGCGTGGGC GCTTGCCCGC ATAATGGTGC ATGGAATGAT
GGAATAGGAC CTCGGTTCTA TTTTGTTGGT TTTCGGAGAG GTAATGATTG AGAGGGACAG
ACGGGGGCAT TCGTATTGCG GCGTTAGAGG TGAAATTCTT GGATCGCCGC AAGACGGACG
AGAGCGAAAG CATTTGCCAA GAATGTTTTC CTTAATCAAG AACGAAAGTC AGAGGCTCGA
AGACGATCAG ATACCGTCGT AGTTCTGACC ATAAACCATG CCAACTGGCA ATCCGCCGGA
GTTACTACAA TGACTCGGCA GGCAGCCCCC GGGAAACCAA AGTTTCTGGG TTCCGGGGGG
AGTATGGTTG CAAAGCTGAA ACTTAAAGGA ATTGACGGAA GGGCACCACC AGGAGTGGAG
CCTGTGGCTT AATTTGACTC AACACGGGAA ACCTCACCCG GCCCGGACAC TGTAAGGATT
GACAGATTGA GAGCTCTTTC TTGATTCGGT GGGTGGTGGT GCATGGCCGT TCTTAGTTGG
TGGAGCGATT TGTCTGGTTA ATTCCGATAA CGAACGAGAC TCTTTCCTGC TAAATAGTTC
GGGGCTATAT AGCCCCGCAA CTTCTTAGAG GGACAGGTGG CGTATAGCCA CACGAGATTG
AGCAATAACA GGTCTGTGAT GCCCTTAGAT GTTCGGGGCC GCACACGCGC TACATTGAAT
GGATCAACGT GCGTCTAGCC TTGCCCGAAA GGGCTGGGAA ACCCGTTGAA ACCCATTCGT
GATAGGGATT GGGGCTTGCA ATTGTTTCCC ATGAACGAGG AATTCCTAGT AAGCGCGAGT
CATCAGCTCG CGTTGATTGC GTCCCTGCCC TTTGTACACA CCGCCCGTCG CTACTACCGA
TCGCTCCAGT TAATGAACGC TTCGGATTGG TTAAGCGGGT TTCGGCCCGC TCGCGTGCCG
AGAAGATGCG TGAATTATCC GGGGTAGAGG TCGTAAAAGT CGTAACAAGG TATTCATTAA
TCAGCGGAGG AAAAGAAACT AACTAGGATT CCCCTAGTAA CGGCGAGTGA AGCGGGAAGA
GCCCAGCACC GAATCCCCCA GCGTCTGGCC GCTGACGGGA AATGTGGTGT TAGCGGGAGC
CCATCGTTGT CGCGTTCCGC CGCCAAAGTC CACCTGATCG TGGCATCCCA GAGCGGGTGA
GAGGCCCGTG ACGGCGGCGG ACGCGTTCGA CTCTCTCCAG GAGTCGGGTT GTTTGAGAAT
GCAGCCCAAA GCGGGTGGTA AACTCCATCT AAGGCTAAAT ACAGACACGA GTCCGATAGC
AAACAAGTAC CGTGAGGGAA AGTTGAAAAG AACTTTGAAG AGAGAGTTCA AGAGTACGTG
AAACCGTATA GAGTCAAACG GGTGGACCCG CGAGGTGTCG GCCCGGGGAA TTCAGCGGTG
CACTTTCTCC GACGAGAGCC ACGACCGGGT CGGCCACTCC GCGCGCGTCA AGGTGACCCA
CGTCCCTGGG AGCTTACAGG TCGCTGCGAT GGAGTCGGCA CGCC---GGC CCGAGGACGC
GCCGCGCGTC AGGCTCCGGA CCGTCCCGGT GCGTACGACG T--TTTCGCA GTGTGCCGAG
ACCGCGCGCC GTTCCGGATG CAGGGGTCGG TGGCGAATCG GTCGGTACTC CACCCGACCC
GTCTTGAAAC ACGGACCAAG GAGTCTAACA TGTGCGCGAG TCACAGGGAC TCGAATCCCG
AAGGCGCAAT GAAAGTGAAG GTCGGCCTCG GCCGTCCGAG GCAGGATCCC GTCCCGGGCG
CACTGCCGGC CCGTCTCGAT AGCGA----G TT-------- --CTCGTCTG CGAGGCGGAG
CAAGAGCGTA CACGTTGGTA CCCGAAAGAT GGTGAACTAT GCCTGAGCAG GACGAAGTCA
GAGGAAACTC TGATGGAGGT CCGCAGCGAT TCACGTAAAT CCACCGGAGG AAAGGCTCCC
CGCAAACAGC TGGCCACCAA GGCTGCACGC AAGAGTGCTC CGGCCACCGG CGGCGTGAAG
AAGCCCCACA GGTACAGGCC CGGAACCGTC GCCCTGAGAG AGATCAGGAG GTACCAGAAG
AGCACCGAGC TGCTCATCCG CAAGCTGCCA TTCCAGCGCC TGGTGAGAGA GATTGCCCAG
GACTTCAAGA CCGACCTGCG CTTCCAGAGC TCTGCCGTCA TGGCTCTGCA GGAAGCCAGC
GAAGCCTACC TGGTCGGCCT GTTCGAGGAC ACCAACCTGT GTGCCATCCA CGCCAAGCGT
-ACTCTTTAT TTTATTTTTG GGGTTTGAGC AGGTCTTGTT GGAAGTTCTC TCAGAGTTTT
AATTCGGGTT GAACTTTCAC GTCCTGGAAG ATTTTTGGGT GATGATCACT TGTATAATGT
TGTTGTTACA GCGCATGCTT TTGTTATAAT TTTTTTTTTG GTAATGCCTA TAATAGTTGG
GGGATTCGGA AATTGGTTGG TCCCTCTTAT ACTTGTTTGT CCTGATATGG CTTTTCCTCG
GATGAACAAT ATGAGATTTT GGTTACTTCC GCCTGCTCTA TCTCTTTTGT TGGGTTCTTC
TTTTGTTGAA GGAGGGGTTG GTTCTGGATG AACAGTTTAT CCCCCTTTGG CGGGAAATGT
TAGTCATGGG GGTGGTTCAA TGGATTTTGC TATTTTCTCT CTTCATCTTG CTGGTGTTTC

TTCTATTTTA GGGGCTATTA ATTTTTTGGT AAGTATTGGA AATATGCGAT CAGAAATTAT
GGGTTTAAAA CGGGTTACAT TGTTTTGCTG GTCTATTGGA ATTACTGCTT TTCTTTTAGT
GGTGGCAATA CCAGTTTTGG CTGGGGCAAT TACTATGCTT TTATTTGATC GCAACTTTAA
TACTTCATTT TTTGATCCTG CAGGGGGAGG AGATCCTATT TTATTTGTTC ATCTATTTT

>Kellia_porculus
TAGTCATATG CTTGTCTCAA AGATTAAGCC ATGCATGTCT AAGTACACGC CAGATTCATG
GTGAAACTGC GAATGGCTCA TTAAATCAGT TATGGTTCCT TAGATCGTAC AATCCTACTT
GGATAACCGT GGCAATTCTA GAGCTAATAC ATGCGTCAAA GCTCCGACCT TCGGGGAAGA
GCGCTTTTGT TAGCAAGACC AACCCGATCG GCTGTTGACT CTGAACAACT TTGTGCTGAT
CGCACGGCCT AGCGCCGGCG ATGTATCTTT CGAATGTCTG CCCTATCAAC TGTCGATGGT
ACGTGCTATG CCTACCATGG TTGTAACGGG TAACGGGGAA TCAGGGTTCG ATTCCGGAGA
GGGAGCATGA GAAACGGCTA CCACATCCAA GGAAGGCAGC AGGCGCGCAA ATTACCCAAT
CCCGACACGG GGAGGTAGTG ACGAAAAATA ACAATACGGG ACTCTTTCGA GGCCCCGTAA
TTGGAATGAG TACACTTTAA ATCCTTTAAC GAGGATCCAT TGGAGGGCAA GTCTGGTGCC
AGCAGCCGCG GTAATTCCAG CTCCAATAGC GTATATTAAA GTTGTTGCAG TTAAAAAGCT
CGTAGTTGGA TCTCGGGTGC AGGCTTGCGG TCCGTCTCGC GACGGCTGCT CGTCCTGGCA
GCCTAGCTGC GGTATCCCTT GGTGCTCTTG ACTGAGTGTC GGTGGCCGGA ACGTTTACTT
TGATGAAATT AGAGTGTTCA AAGCGCGGGC GCTTGCCCGC ATAATGGTGC ATGGAATGAT
AGAATAGGAC CTCGGTTCTA TTTTGTTGGT TTTCGGAGAG GTAATGATTG AGAGGGACAG
ACGGGGGCAT TCGTATTGCG GCGTTAGAGG TGAAATTCTT GGATCGCCGC AAGACGGACT
ACAGCGAAAG CATTTGCCAA GAATGTTTTC CTTAATCAAG AACGAAAGTC AGAGGCTCGA
AGACGATCAG ATACCGTCGT AGTTCTGACC ATAAACCATG CCGACTGGCA ATCCGCCGGA
GTTACTACAA TGACTCGGCG AGCAGCCCCC GGGAAACCAA AGTTTCTGGG TTCCGGGGGG
AGTATGGTTG CAAAGCTGAA ACTTAAAGGA ATTGACGGAA GGGCACCACC AGGAGTGGAG
CCTGTGGCTT AATTTGACTC AACACGGGAA ACCTCACCCG GCCCGGACAC CGTTAGGATT
GACAGATTGA GAGCTCTTTC TTGATTCGGT GGGTGGTGGT GCATGGCCGT TCTTAGTTGG
TGGAGCGATT TGTCTGGTTA ATTCCGATAA CGAACGAGAC TCTAGCCTAC TAAATAGTTC
GAGGATATAT AACCTCGCAA CTTCTTAGAG GGACAGGTGG CGTATAGCCA CACGAGATTG
AGCAATAACA GGTCTGTGAT GCCCTTAGAT GTCCGGGGCC GCACACGCGC TACATTGAAT
GGATCAACGT GCGTCTAGCC TTGCCCGAAA GGGCTGGGAA ACCCGTTGAA ACCCATTCGT
GATAGGGACT GGGGCTTGCA ATTATTTCCC ATGAACGAGG AATTCCTAGT AAGCGCGAGT
CATCAGCTCG CGTTGATTGC GTCCCTGCCC TTTGTACACA CCGCCCGTCG CTACTACCGA
TCGCTCCAGT TAATGAACGC CTCGGATTGG TTAAGCGGGT TTCGGCCTGC TCGCGCGCCG
AGAAGACGTG TAAATTATCT GGAGTAGAGG GAGTAAAAGT CGTAACAAGG TATCCATCAA
TAAGCGGAGG AAAAGAGACT AACTAGGATT CCCCTAGTAA CGGCGAGTGA AGCGGGAAGA
GCCCAGCACC GAATCCCCCG GCGTCTGGTC GCCGACGGGA CCTGTGGTGT TAGCGGGAGT
CTTTTGTCAG CGTGCTTCGG CACCAAAGTC CACCTGATCG TGGCTACCCC TGGCGGGTGA
GAGGCCCGTT CCGGTGTCGG ACGCGTTCGA CTCTCTCCAG GAGTCGGGTT GTTTGAGAAT
GCAGCCCAAA GTGGGTGGTA AACTCCACCT AAGGCTAAAT ACCGACACGA GTCCGATAGA
GGACAAGTAC CGTGAGGGAA AGTTGAAAAG AACTTTGAAG AGAGAGTTCA AGAGTACGTG
AAACCGCATA GAGGCAAACG GGTGGATCCG CAAGGTGTTG GACCGGGGAA TTCAGCGGTG
CACTTTCTCC GACGAGAGCC ACGACCGGGT CCGCTGCTCG GGATTCGCGA AGGTTTCCAG
GCG-GTTGGG AGCTTACAGC GCGTCGTTCT CGAGTC-GTA CGTCGCGGGC CTGAGGACGC
GCCGCGCGCC CGGTGCTGGG TCGTCTTCGT GCGTTCGACT GTCT-CGGCA GTGCGTTGCG
ACCGCGTGCG GTCCCGG-TG CTTGGGTCGG TGGCGAATCA GTCGGTGCTC CACCCGACCC
GTCTTGAAAC ACGGACCAAG GAGTCTAACA TGTGCGCGAG TCATGGGGAC CCGAATCCCG
AAGGCGCAAT GAAAGTGAAG GCCGCCTACG GCTGGCCGAG GCAGGATCCC GTCG-GGGCG
CACTGCCGGC CCGTCTCGAT AGCGGCGCCG TCGGGTCGCG CGTTTGTCTA CGAGGCGGAG
CAAGAGCGTA CACGTTGGTA CCCGAAAGAT GGTGAACTAT GCCTGAGCAG GACGAAGTCA

GAGGAAACTC TGATGGAGGT CCGCAGCGAT TCTCGAAAAT NCACTGGTGG GAAAGCCCCC
AGGAAACAAC TGGCCACCAA GGCCGCACGT AAGAGTGCAC CGGCTACTGG TGGGGTAAAG
AAGCCCCATA GGTACAGGCC TGGAACCGTC GCTCTCCGTG AGATCAGACG ATACCAGAAG
AGCACCGAGC TTCTTATCCG AAAGCTGCCC TTCCAGCGTC TCGTCCGTGA GATCGCCCAG
GACTTCAAGA CTGACCTCCG ATTCCAGAGC TCAGCCGTTA TGGCCCTGCA GGAGGCTAGC
GAGGCTTACC TGGTTGGACT GTTTGAGGAC ACCAACTTGT GCGCTATCCA CGCCAAGAGA
AACTCTTTAC TTTTTATTTG GAATTTGATC TGGGTTAGTA GGAACGTCTT TCAGTGTTTT
AATTCGATTA GAATTATCAC GCCCCGGGGC ATTCTTAGGT GATGATCATT TATATAATGT
CATTGTTACA GCCCACGCAT TTGTTATAAT TTTTTTCCTT GTTATACCAA TAATAGTAGG
CGGATTTGGG AATTGATTAG TCCCCCTAAT ATTAACCTCT CCTGATATAG CGTTTCCGCG
GATAAACAAT ATAAGGTTTT GACTTCTTCC TCCGGCTTTA TTTTTACTTT TAAGCTCGGC
TTTTGTAGAA AGAGGGGTTG GAACGGGATG AACGGTTTAT CCTCCTTTAT CGGCAAATGT
TGCTCATAGG GGAGGGTCGG TAGATTATGG AATTTTCTCT CTTCACTTAG CGGGTGTTTC
TTCTATTTTA GGGGCTATTA ATTTTTTAGC CACAACAATT AATATGCGCC CAGAAATTAT
AGAATTAAAA CGTGTTACTT TATTTGTTTG ATCTATTGCT ATCACCGCGT TTTTACTTGT
TGTTGCGATG CCGGTTTTAG CAGGTGCTAT TACTATGCTT TTAACGGATC GAAATTTTAA
TACATCGTTT TTCGATCCGT CAGGAGGCGG GGACCCTATT TTATTTGTTC ATTTGTTTT

>Lasaea_undulata
TAGTCATATG CTTGTCTCAA AGATTAAGCC ATGCATGTCT AAGTACACGC CAGATTAATG
GTGAAACTGC GAATGGCTCA TTAAATCAGT TATGGTTCCT TAGATCGTAC AATCCTACTT
GGATAACTGT GGCAATTCTA GAGCTAATAC ATGCGTCAAA GCTCCGACCT CAGGGGAAGA
GCGCTTTTGT TAGCAAAACC AATCCGGCCG GTTGTTGACT CTGAACAACT TTGTGCTGAT
CGCACGGCCT AGCGCCGGCG ACGTATCTTT CGAATGTCTG CCCTATCAAC TGTCGATGGT
ACGTGCTATG CCTACCATGG TTGTAACGGG TAACGGGGAA TCAGGGTTCG ATTCCGGAGA
GGGAGCATGA GAAACGGCTA CCACATCCAA GGAAGGCAGC AGGCGCGCAA ATTACCCAAT
CCCGACACGG GGAGGTAGTG ACGAAAAATA ACAATACGGG ACTCTTTCGA GGCCCCGTAA
TTGGAATGAG TACACTTTAA ATCCTTTAAC GAGGATCCAT TGGAGGGCAA GTCTGGTGCC
AGCAGCCGCG GTAATTCCAG CTCCAATAGC GTATATTAAA GTTGTTGCAG TTAAAAAGCT
CGTAGTTGGA TCTCGGGTGC AGGCTTGCGG TCCGCCTCGC GGCGGCTGCT CGTCCTGGCA
GCCTAGCTGC GGTATCCCTT GGTGCTCTTG ATTGAGTGTC GGTGGCCGGA ACGTTTACTT
TGATGAAATT AGAGTGTTCA AAGCGCGGGC GTTTGCTCGT ATAATGGTGC ATGGAATGAT
AGAATAGGAC CTCGGTTCTA TTTTGTTGGT TTTCGGAGAG GTAATGATTG AGAGGGACAG
ACGGGGGCAT TCGTATTGCG GCGTTAGAGG TGAAATTCTT GGATCGCCGC AAGACGGACT
ACAGCGAAAG CATTTGCCAA GAATGTTTTC CTTAATCAAG AACGAAAGTC AGAGGCTCGA
AGACGATCAG ATACCGTCGT AGTTCTGACC ATAAACCATG CCGACTGGCA ATCCGCCGGA
GTTACTACAA TGACTCGGCG AGCAGCCCCC GGGAAACCAA AGTTTCTGGG TTCCGGGGGG
AGTATGGTTG CAAAGCTGAA ACTTAAAGGA ATTGACGGAA GGGCACCACC AGGAGTGGAG
CCTGTGGCTT AATTTGACTC AACACGGGAA ACCTCACCCG GCCCGGACAC CGTTAGGATT
GACAGATTGA GAGCTCTTTC TTGATTCGGT GGGTGGTGGT GCATGGCCGT TCTTAGTTGG
TGGAGCGATT TGTCTGGTTA ATTCCGATAA CGAACGAGAC TCTAGCCTGC TAAATAGTTC
GAGGATCTAT AACCTCGCAA CTTCTTAGAG GGACAGGTGG CGTATAGCCA CACGAGATTG
AGCAATAACA GGTCTGTGAT GCCCTTAGAT GTCCGGGGCC GCACACGCGC TACATTGAAT
GGATCAACGT GCGTCTAGCC TTGCCCGAGA GGGCTGGGAA ACCCGTTGAA ACCCATTCGT
GATAGGGACT GGGGCTTGCA ATTATTTCCC ATGAACGAGG AATTCCTAGT AAGCGCGAGT
CATCAGCTCG CGTTGATTGC GTCCCTGCCC TTTGTACACA CCGCCCGTCG CTACTACCGA
TCGCTCCTGT TAATGAACGC CTCGGATTGG TTAAGCGGGT TTCGGCCTGC TCGCGTGCCG
AGAAGACGTG TAAATTATCA GGAGTAGAGG GAGTAAAAGT CGTAACAAGG TATCCATAAA
TAAGCGGAGG AAAAGAGACT AACTAGGATT CCCCTAGTAA CGGCGAGTGA AGCGGGAAGA
GCCCAGCACC GAATCCCCCG GCGTCTGGCC GTCGACGGGA CCTGTGGTGT TAGCGGGAGT

CTTTTGTCGG CGTGCTTCGG CACCCAAGTC CACCTGATCG TGGCAACCCC TGGCGGGTGA
GAGGCCCGTT CCGGTGTCGG ACGCGCTCGA CTCTCTCCAG GAGTCGGGTT GTTTGAGAAT
GCAGCCCAAA GTGGGTGGTA AACTCCACCT AAGGCTAAAT ACAGACACGA GTCCGATAGA
GGACAAGTAC CGTGAGGGAA AGTTGAAAAG AACTTTGAAG AGAGAGTTCA AGAGTACGTG
AAACCGCATA GAGGCAAACG GGTGGATCCG CAAGGTGTTG GACCGGGGAA TTCAGCGGTG
CACTTTCCCC GACGAGAGCC ACGACCGGGT TCGCTGCTCG GGATGTTCGA AGGTGTCCAG
GCGGGCTGGG AG-TTACAGC GAACCGACGC CGAGTCTGTA TGTCGCGAGC CCGAGGACGC
GCCGCTCGTC CGGTGCCGGG TCGTCTTCGC GCGTTCCACT GTCT-CGGCA GTGCGCTGCG
ACCGCGTGCG GTCCCGG-TG CTTGGGTCGG TGGCGAATCA GTCGGTACTC CACCCGACCC
GTCTTGAAAC ACGGACCAAG GAGTCTAACA TGTGCGCGAG TCATGGGGAC TCGAATCCCG
AAGGCGCAAT GAAAGTGAAG GCCGCCCACG -GTGGCCGAG GCAGGATCCC GTCG-GGGCG
CACTGCCGGC CCGTCTCGAT AGCGGCGCGC TCGCGAGCTG CGTTCGTCTA CGAGGCGGAG
CAAGAGCGTA CACGTTGGTA CCCGAAAGAT GGTGAACTAT GCCTGAGCAG GACGAAGTCA
GAGGAAACTC TGATGGAGGT CCGCAGCGAT TCCCGAAAAT CCACTGGTGG GAAAGCCCCC
AGGAAACAGC TGGCCACTAA GGCCGCACGT AAGAGTGCAC CGGCCACTGG TGGAGTGAAG
AAACCCCACA GATACAGGCC CGGAACCGTG GCTCTCCGTG AGATCAGGAG GTACCAAAAG
AGCACCGAGC TTCTGATCAG AAAATTGCCC TTCCAGCGAC TCGTCCGTGA GATCGCCCAG
GACTTCAAGA CAGACCTGCG ATTCCAGAGC TCTGCCGTCA TGGCTTTGCA AGAGGCCAGC
GAGGCTTACT TGGTCGGACT GTTCGAGGAC ACCAACTTGT GTGCTATCCA CGCCAAGCGT
---------- ---------- ---------- ---------- ----CTTCTT TTAGGGTTTT
GATTCGCCTA GAGTTATCCC GTCCTGGTGC TTTTTTGGGG GATGACCATT TGTACAATGT
GATTGTTACA GCTCATGCTT TTGTGATAAT TTTTTTTTTA GTAATACCAA TAATGGTAGG
CGGTTTTGGG AACTGGTTGG TGCCATTAAT ACTGACTTCT CCTGATATAG CTTTTCCTCG
TATAAATAAT ATAAGCTTTT GGCTTTTGCC TCCTGCTTTA TTTTTACTTT TGAGGTCTGC
TTTTGTGGAA AGTGGTGTGG GAACTGGTTG GACAGTGTAC CCCCCTCTTT CAGCTAATAT
CGCACATAGG GGTGGGTCTG TAGATTTTGG TATTTTTTCT CTTCATTTAG CTGGGGTGTC
TTCTATCTTA GGTGCTATCA ATTTTCTTGC TAGGACTGTC AATATACGAC CAGAAATCAT
AGAACTAAAA CGAGTTACTT TGTTTGTTTG GTCTATTGCT ATCACAGCTT TCTTGTTGGT
AGTGGCGATG CCGGTTTTAG CTGGTGCAAT T--------- ---------- ----------
---------- ---------- ---------- ---------- ---------- ---------

>Litigella_pacifica
TAGTCATATG CTTGTCTCAA AGATTAAGCC ATGCATGTCT AAGTACACGC CAGATACAAG
GTGAAACTGC GAATGGCTCA TTAAATCAGT TATGGTTCCT TAGATCGTAC AATCCTACTT
GGATAACTGT GGCAATTCTA GAGCTAATAC ATGCATCAAA GCTCCGACCT CACGGGAAGA
GCGCTTTTGT TAGCAAAACC AATCCGGCCG GTTGTTGACT CTGAACAACT TTGTGCTGAT
CGCACGGCCT CGAGCCGGCG ACGTATCTTT CGAATGTCTG CCCTATCAAC TGTCGATGGT
ACGTGCTATG CCTACCATGG TTGTAACGGG TAACGGGGAA TCAGGGTTCG ATTCCGGAGA
GGGAGCATGA GAAACGGCTA CCACATCCAA GGAAGGCAGC AGGCGCGCAA ATTACCCAAT
CCCGACACGG GGAGGTAGTG ACGAAAAATA ACAATACGGG ACTCTTTCGA GGCCCCGTAA
TTGGAATGAG TACACTTTAA ATCCTTTAAC GAGGATCCAT TGGAGGGCAA GTCTGGTGCC
AGCAGCCGCG GTAATTCCAG CTCCAATAGC GTATATTAAA GTTGTTGCAG TTAAAAAGCT
CGTAGTTGGA TCTCGGGTGC CGGCTTGCGG TCCGCCTCGC GGTGGCTGCT CGTCCGGGCA
GCCTAGCTGC GGTGTCCCTT GGTGCTCTTG ATTGAGTGTC GGTGGCCGCA ACGTTTACTT
TGATGAAATT AGAGTGTTCA AAGCGTGGGC GTTTGCCCGC ATAATGGTGC ATGGAATGAT
GGAATAGGAC CTCGGTTCTA TTTTGTTGGT TTTCGGAGAG GTAATGATTG AGAGGGACAG
ACGGGGGCAT TCGTATTGCG GCGTTAGAGG TGAAATTCTT GGATCGCCGC AAGACGGACG
AGAGCGAAAG CATTTGCCAA GAATGTTTTC CTTAATCAAG AACGAAAGTC AGAGGCTCGA
AGACGATCAG ATACCGTCGT AGTTCTGACC ATAAACCATG CCAACTGGCA ATCCGCCGGA
GTTACTACAA TGACTCGGCG AGCAGCCCCC GGGAAACCAA AGTTTCTGGG TTCCGGGGGG

AGTATGGTTG CAAAGCTGAA ACTTAAAGGA ATTGACGGAA GGGCACCACC AGGAGTGGAG
CCTGTGGCTT AATTTGACTC AACACGGGAA ACCTCACCCG GCCCGGACAC CGTAAGGATT
GACAGATTGA GAGCTCTTTC TTGATTCGGT GGGTGGTGGT GCATGGCCGT TCGTAGTTGG
TGGAGCGATT TGTCTGGTTA ATTCCGATAA CGAACGAGAC TCTAGCCTAC TAAATAGTTC
GAGGCTATAT AGCCTCGCAA CTTCTTAGAG GGACAGGTGG CGTATAGCCA CACGAGATTG
AGCAATAACA GGTCTGTGAT GCCCTTAGAT GTTCGGGGCC GCACACGCGC TACATTGAAT
GGATCAACGT GCGTCTAGCC TTGCCCGAAA GGGCTGGGAA ACCCGTTGAA ACCCATTCGT
GATAGGGACT GGGGCTTGCA ATTATTTCCC ATGAACGAGG AATTCCTAGT AAGCGCGAGT
CATCAGCTCG CGTTGATTGC GTCCCTGCCC TTTGTACACA CCGCCCGTCG CTACTACCGA
TCGCTCCAGT TAATGAACGC TTCGGATTGG TTAAGCGGGT TTCGGCCTGC TCGCGTGCCG
AGAAGATGTG TGAATTATCC GGGGTAGAGG TCGTAAAAGT CGTAACAAGG TATCCATTAA
TAAGCGGAGG AAAAGAGACT AACTAGGATT CCCCTAGTAA CGGCGAGTGA AGCGGGAAGA
GCCCAGCACC GAATCCCCCA GCGTCTGGCC GCTGACGGGA AATGTGGTGT TAGCGGGAGT
CCTACGTCGG CGTGTTCGGT TGCCAAAGTC CACCTGATCG TGGCAACCCC AGGCGGGTGA
GAGGCCCGTT TCGGTGACCG GCGCGTTGGA CTCTCTCCAG GAGTCGGGTT GTTTGAGAAT
GCAGCCCAAA GTGGGTGGTA AACTCCATCT AAGGCTAAAT ACTGACACGA GTCCGATAGA
GGACAAGTAC CGTGAGGGAA AGTTGAAAAG AACTTTGAAG AGAGAGTTCA AGAGTACGTG
AAACCGCATA GAGTCAAACG GGTGGATCCG CAAGGTGTTG GACCGGGGAA CTCAGC----
---------- ---------- ---------- ---------- ---------- ----------
---------- ---------- ---------- ---------- ---------- -----GACGC
GCCGTGCGCT CGGCTCCGGG CCGTCCTGGC GTGTTCGACC T--TTCAGCA GTGTGCCGTA
ACCGCTCGCC GGTTCGGTGT TGAGGGTCGG TGGCGAATCA GTCGGTACTC CACCCGACCC
GTCTTGAAAC ACGGACCAAG GAGTCTAACA TGTGCGCGAG TCATAGGGAC CCGAATCCCG
AAGGCGCAAT GAAAGTGAAG GTCGTCCTCG GTCGGCCGAG GCAGGATCCC GTCGCGGGCG
CACTGCCGGC CCGTCTCGAT AGCGGCGGTG GGTCGGCCTG CCTTCGTCTG CGAGGCGGAG
CAAGAGCGTA CACGTTGGTA CCCGAAAGAT GGTGAACTAT GCCTGAGCAG GACGAAGTCA
GAGGAAACTC TGATGGAGGT CCGCAGCGAT TCTCGAAAAT CTACGGGAGG AAAGGCTCCC
AGGAAGCAGC TGGCCACCAA GGCTGCACGT AAGAGTGCCC CAGCCACCGG TGGAGTGAAG
AAACCCCACA GGTACAGGCC CGGTACCGTG GCCCTCCGAG AGATCAGGAG ATACCAGAAG
AGCACCGAGC TCCTGATCAG AAAACTGCCA TTCCAGCGTC TGGTCCGTGA GATCGCCCAG
GACTTCAAGA CTGACCTCCG CTTCCAGAGC TCTGCTGTCA TGGCCCTGCA GGAGGCAAGC
GAGGCCTACC TCGTTGGTCT GTTCGAGGAC ACNAACTTGT GCGCCATCCA CGCCAAGCGT
TACATTATAT TTTTTATTTG GTATTTGATC AGGTTTGGTA GGTACTTCAT TTAGAGTATT
AATTCGTTTG GAGCTTTCTC GTCCTGGCGC ATTTCTGGGT GACGATCATT TGTATAATGT
AATTGTTACT GCCCATGCAT TTGTTATAAT TTTTTTCTTG GTTATACCAA TAATAGTGGG
AGGATTCGGT AATTGATTGG TTCCTTTAAT ATTAACTTCT CCTGATATAG CATTCCCTCG
TATAAATAAT ATGAGGTTTT GGTTATTACC ACCTGCTATA TTTCTTTTAT TAAGCTCTGC
TTTTGTTGAA AGTGGTGCAG GTACTGGTTG AACTGTCTAT CCACCATTAT CTGGAAATAT
TACTCATAGT GGCGGTTCTG TGGATTATGC AATTTTTTCT CTTCATTTGG CTGGTGTTTC
TTCTATTTTA GGAGCAATCA ATTTTTTAGC TAGAACTATT AATATGCGTC CTGAGGTGAT
AGAGTTGAAG CGGGTGACTT TATTTGTTTG ATCAATTGCT ATTACTGCAT TTTTGTTAGT
TGTTGCTATA CCTGTGTTGG CTGGTGCAAT TACAATATTA TTAACAGATC GGAATTTTAA
CACTTCTTTT TTTGATCCTT CTGGTGGCGG AGATCCAATT TTATTTGTTC ATTTGTTTT

>Melliteryx_puncticulata
TAGTCATATG CTTGTCTCAA AGATTAAGCC ATGCATGTCT AAGTACACGC CAGATTAATG
GTGAAACTGC GAATGGCTCA TTAAATCAGT TATGGTTCCT TAGATCGTAC AATCCTACTT
GGATAACCGT GGCAATTCTA GAGCTAATAC ATGCGTCAAA GCTCCGACCT TCGGGGAAGA
GCGCTTTTGT TAGCAAAACC AATCCGGTCG GTTGTTGACT CTGAACAACT TTGTGCTGAT
CGCACGGCCT AGCGCCGGCG ATGTATCTTT CGAATGTCTG CCCTATCAAC TGTCGATGGT

ACGTGCTATG CCTACCATGG TTGTAACGGG TAACGGGGAA TCAGGGTTCG ATTCCGGAGA
GGGAGCATGA GAAACGGCTA CCACATCCAA GGAAGGCAGC AGGCGCGCAA ATTACCCAAT
CCCGACACGG GGAGGTAGTG ACGAAAAATA ACAATACGGG ACTCTTTCGA GGCCCCGTAA
TTGGAATGAG TACACTTTAA ATCCTTTAAC GAGGATCAAT TGGAGGGCAA GTCTGGTGCC
AGCAGCCGCG GTAATTCCAG CTCCAATAGC GTATATTAAA GTTGTTGCAG TTAAAAAGCT
CGTAGTTGGA TCTCGGGTGC AGGCTTGCGG TCCGCCTCGC GGCGGCTGCT CGTCCTGGCA
GCCTAGCTGC GGTATCCCTT GGTGCTCTTG ACTGAGTGTC GGTGGCCGGA ACGTTTACTT
TGATGAAATT AGAGTGTTCA AAGCGCGGGC GCTTGCCCGC ATAATGGTGC ATGGAATGAT
AGAATAGGAC CTCGGTTCTA TTTTGTTGGT TTTCGGAGAG GTAATGATTG AGAGGGACAG
ACGGGGGCAT TCGTATTGCG GCGTTAGAGG TGAAATTCTT GGATCGCCGC AAGACGGACT
ACAGCGAAAG CATTTGCCAA GAATGTTTTC CTTAATCAAG AACGAAAGTC AGAGGCTCGA
AGACGATCAG ATACCGTCGT AGTTCTGACC ATAAACCATG CCGACTGGCA ATCCGCCGGA
GTTACTACAA TGACTCGGCG AGCAGCCCCC GGGAAACCAA AGTTTCTGGG TTCCGGGGGG
AGTATGGTTG CAAAGCTGAA ACTTAAAGGA ATTGACGGAA GGGCACCACC AGGAGTGGAG
CCTGTGGCTT AATTTGACTC AACACGGGAA ACCTCACCCG GCCCGGACAC CGTTAGGATT
GACAGATTGA GAGCTCTTTC TTGATTCGGT GGGTGGTGGT GCATGGCCGT TCTTAGTTGG
TGGAGCGATT TGTCTGGTTA ATTCCGATAA CGAACGAGAC TCTAGCCTAC TAAATAGTTC
GAGGATATAT AACCTCGCAA CTTCTTAGAG GGACAGGTGG CGTATAGCCA CACGAGATTG
AGCAATAACA GGTCTGTGAT GCCCTTAGAT GTCCGGGGCC GCACACGCGC TACATTGAAT
GGATCAACGT GCGTCTAGCC TTGCCCGAAA GGGCTGGGAA ACCCGTTGAA ACCCATTCGT
GATAGGGACT GGGGCTTGCA ATTATTTCCC ATGAACGAGG AATTCCTAGT AAGCGCGAGT
CATCAGCTCG CGTTGATTGC GTCCCTGCCC TTTGTACACA CCGCCCGTCG CTACTACCGA
TCGCTCCAGT TAATGAACGC CTCGGATTGG TTAAGCGGGT TTCGGCCTGC TCGCGTGCCG
AGAAGACGTG TAAATTATCT GGAGTAGAGG GAGTAAAAGT CGTAACAAGG TATCCATCAA
TAAGCGGAGG AAAAGAGACT AACTAGGATT CCCCCAGTAA CGGCGAGTGA AGCGGGAAGA
GCCCAGCACC GAATCCCCCG GCGTCTGGCC GCCGACGGGA CCTGTGGTGT TAGCGGGAGT
CTTTTGTCGG CGTGCTTCGG CACCAAAGTC CACCTGATCG TGGCAACCCC TGGCGGGTGA
GAGGCCCGTT CCGGTGTCGG ACGCGTCCGA CTCTCTCCAG GAGTCGGGTT GTTTGAGAAT
GCAGCCCAAA GTGGGTGGTA AACTCCACCT AAGGCTAAAT ACGGACACGA GTCCGATAGA
GGACAAGTAC CGTGAGGGAA AGTTGAAAAG AACTTTGAAG AGAGAGTTCA AGAGTACGTG
AAACCGCATA GAGGCAAACG GGTGGATCCG CAAGGTGTTG GACCGGGGAA TTCAGCGGTG
CACTTTCTCC GACGAGAGCC ACGACCCGGG TCGCTGCTCG AGATGCGTGA AGGTTTCCAG
GCGCCTCGGG AGCTTATAGC GCGCCGTTCT CGAGTCTGTA TGTCGCGGGC CCGAGGACGC
GCCGCGCGCC CGGCACCGGG TCGTCTTCGC GCGTTCGACT GTTCTCGGCA GTGCGTTGCG
ACCGCGTGCG GTCCCGGCG- TGAGGGTCGG TGGCGAATCA GTCGGTGCTC CACCCGACCC
GTCTTGAAAC ACGGACCAAG GAGTCTAACA TGTGCGCGAG TCATGGGGAC CCGAATCCCG
AAGGCGCAAT GAAAGTGAAG GCCGCCCTCG GCTGGCCGAG GCAGGATCCC GTCGCGGGCG
CACTGCCGGC CCGTCTCGAT AGCGGCGCGG TCGCGGCCTG CGTTCGTCTA CGAGGCGGAG
CAAGAGCGTA CACGTTGGTA CCCGAAAGAT GGTGAACTAT GCCTGAGCAG GACGAAGTCA
GAGGAAACTC TGATGGAGGT CCGCAGCGAT TCAAGAAAGT CCACTGGTGG GAAAGCCCCC
AGGAAACAGT TGGCCACCAA GGCAGCACGT AAAAGTGCAC CGGCCACTGG TGGTGTTAAG
AAGCCACACA GATACAGGCC AGGAACTGTG GCNCTCCGTG AGATCAGGAG GTACCNNAAG
AGCACCGAGC TCCTCATCCG AAAACTGCCA TTCCAGCGTC TCGTCCGTGA AATCGCACAG
GACTTCAAGA CCGACCTCCG CTTCCAGAGC TCCGCCGTGA TGGCCCTGCA GGAGGCCAGC
GAGGCTTACC TCGTCGGTCT ---------- ---------- ---------- ----------
AACACTGTAT TTTTTATTTG GAATTTGGTC TGGTTTGGTG GGGACTTCTT TTAGGGTATT
AATTCGTCTA GAGTTGTCAC GACCAGGGGC ATTTTTGGGG GATGACCATT TATATAATGT
TATTGTTACA GCCCATGCGT TTGTAATAAT TTTTTTTTTA GTGATGCCTA TAATGGTTGG
AGGATTTGGT AATTGATTGG TGCCTTTAAT ATTAACTTCA CCTGATATAG CTTTTCCTCG
GATAAACAAT ATAAGGTTTT GGTTATTACC TCCTGCTTTG TTTTTACTTT TAAGTTCGGC

TTTTGTTGAA AGAGGTGTAG GAACGGGATG AACGGTTTAT CCTCCTTTAG CGGCTAATGT
GGCTCATAGA GGGGGGTCTG TAGATTATGG GATTTTTTCC CTTCATTTAN CTGGAGTGTC
ATCTATTTTG GGGGCTATTA ATTTTTTAGC AAGAACTGTT AATATGCGTC CGGAAATTAT
AGAATTTAAG CGAGTTACTT TATTTGTTTG GTCAGTAGCT ATCACGGCTT TTTTATTGGT
TGTTGCTATG CCAGTTTTAG CTGGGGCAAT TACTATGCTT TTGACAGATC GAAACTTCAA
TACATCTTTC TTTGATCCTT CTGGAGGAGG AGATCCTATT CTGTTTGTTC ATTTATTTT

>Montacutona_sp
TAGTCATATG CTTGTCTCAA AGATTAAGCC ATGCATGTCT AAGTACACGC CAGATTAATG
GTGAAACTGC GAATGGCTCA TTAAATCAGT TATGGTTCCT TAGATCGTAC AATCCTACTT
GGATAACTGT GGCAATTCTA GAGCTAATAC ATGCGTCAAA GCTCCGACCT TCGGGGAAGA
GCGCTTTTGT TAGCAAGACC AATCCGGTCG GTTGTTGACT CTGAACAACT TTGTGCTGAT
CGCACGGCCT AGCGCCGGCG ACGTATCTTT CGAATGTCTG CCCTATCAAC TGACGATGGT
ACGTGCTATG CCTACCATGG TTGTAACGGG TAACGGGGAA TCAGGGTTCG ATTCCGGAGA
GGGAGCATGA GAAACGGCTA CCACATCCAA GGAAGGCAGC AGGCGCGCAA ATTACCCAAT
CCCGACACGG GGAGGTAGTG ACGAAAAATA ACAATACGGG ACTCTTTCGA GGCCCCGTAA
TTGGAATGAG TACACTTTAA ATCCTTTAAC GAGGATCCAT TGGAGGGCAA GTCTGGTGCC
AGCAGCCGCG GTAATTCCAG CTCCAATAGC GTATATTAAA GTTGCTGCAG TTAAAAAGCT
CGTAGTTGGA TCTCGGGTGT AGGCTTGCGG TCCGCCTCGC GGCGGCTGCT CGTCCTGACA
GCCTAGCTTC GGTGTCCCTT GGTGCTCTTG ACTGAGTGTC GGTGGCCGGA ACGTTTACTT
TGATGAAATT AGAGTGTTTA AAGCGTGGGC GTTTGCCCGT ATAATGGTGC ATGGAATGAT
AGAATAGGAC CTCGGTTCTA TTTTGTTGGT TTTCGGAGAG GTAATGATTG AGAGGGACAG
ACGGGGGCAT TCGTATTGCA GCGTTAGAGG TGAAATTCTT GGATCGTTGC AAGACGGCCG
ACAGCGAAAG CATTTGCCAA GAATGTTTTC CTTAATCAAG AACGAAAGTC AGAGGCTCGA
AGACGATCAG ATACCGTCGT AGTTCTGACC ATAAACTATG CCAACTGGCA ATCCGCCGGA
GTTACTACAA TGACTCGGCG AGCAGCCCCC GGGAAACCAA AGTTTCTCGG TTCCGGGGGG
AGTATGGTTG CAAAGCTGAA ACTTAAAGGA ATTGACGGAA GGGCACCACC AGGAGTGGAG
CCTGTGGCTT AATTTGACTC AACACGGGAA ACCTCACCCG GCCCGGACAC CGTTAGGATT
GACAGATTGA GAGCTCTTTC TTGATTCGGT GGGTGGTGGT GCATGGCCGT TCTTAGTTGG
TGGAGCGATT TGTCTGGTTA ATTCCGATAA CGAACGAGAC TCTAGCCTAC TAAATAGTTC
GAGGATATAC AACCTCGCAA CTTCTTAGAG GGACAGGTGG CGTTTAGCCA CACGAGATTG
AGCAATAACA GGTCTGTGAT GCCCTTAGAT GTCCGGGGCC GCACACGCGC TACATTGAAT
GGATCAACGT GCGTTTAGCC TTGCCCGAAA GGGCTGGGAA ACCCGCTGAA CCCCATTCGT
GATAGGGACT GGGGCTTGCA ATTATTTCCC ATGAACGAGG AATTCCTAGT AAGCGCGAGT
CATCAGCTCG CGTTGATTGC GTCCCTGCCC TTTGTACACA CCGCCCGTCG CTACTACCGA
TCGTTCCAGT TAATGAACAC TTCGGATTGG TTAAGCGGGT TTCGGCCTGC TCGCGTGCCG
AGAAGACTTG TGAATTATCT GGGATAGAGG TCGTAAAAGT CGTAACAAGG TATCCATTAA
TAAGCGGAGG AAAAGAGACT AACTAGGATT CCCCTAGTAA CGGCGAGTGA AGCGGGAAAA
GCCCAGCACC GAATCCCCCG GCATCTGGCT GCCGACGGGA CATGTGGTGT TAGTGGGAGC
CTTGTGTCGG CGTGTTTCGG CACCAAAGTC CACTTGATCG TGGCAATCCA TGGCGGGTGA
GAGACCCGTT CCGGTGTCGA GCGCGTCGGA CTCTCTCCAG GAGTCGGGTT GTTTGAGAAT
GCAGCCCAAA GTGGGTGGTA AACTCCATCT AAGGCTAAAT ACTGACACGA GTCCGATAGA
GGACAAGTAC CGTGAGGGAA AGTTGAAAAG AACTTTGAAG AGAGAGTTCA AGAGTACGTG
AAACCGCATA GAGTCAAACG GGTGGATCCG CAAGGTGTTG GCCCGGGGAA TTCAGCGGTG
CACTTTCTCC GACGAGAGCC ACGACCGGGT CAGCTGCTCG CAGTGCGAGA AGGTTTTCCC
GGGTGCTGGG AGCTTACAGC TCGTCGGCCT CGGGTCTGTA TGTTGCCGAC CCGAGGACGC
GCCGCGCGTC CGGCTCCGGG CCGTCCGTGT GCGTTCGACC TTT---CGTA GAGTGCGGTA
ACCGCGTGCG GGCTCGGGTG CTAGGGTCAG TGGCGAATCA GTCGGTCCTC CACCCGACCC
GTCTTGAAAC ACGGACCAAG GAGTCTAACA TGTGCGCGAG TCATAGGGAC CCGAATCCCG
AAGGCGCAAT GAAAGTGAAG GCCGCCCACG GCTGGCCGAG GCAGGATCCC GTCGCGGGCG

CACTGCCGGC CCGTCTCGAT AGCGGCGC-G GCGTG-CCGT CGTTCGTCTA CGAGGCGGAG
CAAGAGCGTA CACGTTGGTA CCCGAAAGAT GGTGAACTAT GCCTGAGCAG GACGAAGTCA
GAGGAAACTC TGATGGAGGT CCGCAGCGAT TCTAGAAAAT CCACTGGTGG CAAGGCCCCA
CGAAAACAGT TGGCTACCAA GGCCGCACGT AAGAGTGCCC CAGCCACCGG TGGTGTCAAG
AAACCACACA GGTACAGGCC TGGAACCGTC GCTCTCCGTG AGATCAGAAG GTACCAGAAG
AGCACCGAGC TGCTGATCAG AAAATTGCCC TTCCAGCGTC TCGTCCGCGN GATCGCCCAG
GACTTCAAGA CCGATCTCCG ATTCCAGAGC TCTGCCGTCA TGGCTCTCCA GGAGGCCAGC
GAGGCTTACC TGGTCGGTCT GTTNGAGGAC ACCAACTTGT GCGCTATCCA CGCCAAGCGT
AACTCTATAT TTTATTTTCG GAATTTGATC AGGAATAGTA GGTACATCTT TCAGAATTTT
AATTCGTTTA GAACTATCTC GTCCTGGGGC TTTCTTAGGT GATGATCATT TATACAATGT
AATTGTGACA GCTCACGCTT TCGTAATGAT TTTTTTTTTA GTAATACCTA TAATGGTCGG
AGGTTTCGGA AATTGGTTAG TTCCTTTAAT ATTAACTTCT CCCGACATAG CATTCCCTCG
AATAAATAAT ATAAGTTTCT GGTTGTTACC TCCTGCTTTA TTTCTTTTAT TGAGGTCTGC
TTTTGTAGAG AGGGGTGTTG GAACAGGATG AACAGTTTAT CCTCCTTTGT CTAGAAATGT
AACTCATAGA GGTGGTTCCG TTGATTACGG AATTTTTTCT TTACATTTGG CTGGTGTTTC
ATCCATTTTA GGGGCAATTA ATTTCTTAGC AACAACTGTC AATATACGTC CTGAAATTAT
GGAATTAAAG CGTGTAACCT TGTTTGTTTG GTCAATTGCA ATTACAGCCT TTTTGTTAGT
AGTAGCTATA CCTGTCTTAG CTGGAGCTAT CACAATGTTA CTTACTGATC GAAATTTTAA
CACATC---- ---------- ---------- ---------- ---------- ---------

>Mysella_aff_bidentata
TAGTCATATG CTTGTCTCAA AGATTAAGCC ATGCATGTCT AAGTACACGC CAGTATAATG
GTGAAACTGC GAATGGCTCA TTAAATCAGT TATGGTTCCT TAGATCGTAC AATCCTACTT
GGATAACTGT GGCAATTCTA GAGCTAATAC ATGCGTCAAA GCTCCGACCT TAGGGGAAGA
GCGCTTTTGT TAGCAAGACC AATCCGGTCG GTTGTTGACT CTGAACAACT TTGTGCTGAT
CGCACGGCCT AGCGCCGGCG ACGTATCTTT CGAATGTCTG CCCTATCAAC TGACGATGGT
ACGTGCTATG CCTACCATGG TTGTAACGGG TAACGGGGAA TCAGGGTTCG ATTCCGGAGA
GGGAGCATGA GAAACGGCTA CCACATCCAA GGAAGGCAGC AGGCGCGCAA ATTACCCAAT
CCCGACACGG GGAGGTAGTG ACGAAAAATA ACAATACGGG ACTCTTTCGA GGCCCCGTAA
TTGGAATGAG TACACTTTAA ATCCTTTAAC GAGGATCCAT TGGAGGGCAA GTCTGGTGCC
AGCAGCCGCG GTAATTCCAG CTCCAATAGC GTATATTAAA GTTGCTGCAG TTAAAAAGCT
CGTAGTTGGA TCTCGGGTGT AGGCTTGCGG TCCGCCTCGC GGCGGCTGCT CGTCCTGACA
GCCTAGCTTC GGTGTCCCTT GGTGCTCTTG ATTGAGTGTC GGTGGCCGGA ACGTTTACTT
TGATGAAATT AGAGTGCTTA AAGCGTGGGC GTTTGCCCGT ATAATGGTGC ATGGAATGAT
AGAATAGGAC CTCGGTTCTA TTTTGTTGGT TTTCGGAGAG GTAATGATTG AGAGGGACAG
ACGGGGGCAT TCGTATTGCA GCGTTAGAGG TGAAATTCTT GGATCGTTGC AAGACGGCCG
ACAGCGAAAG CATTTGCCAA GAATGTTTTC CTTAATCAAG AACGAAAGTC AGAGGCTCGA
AGACGATCAG ATACCGTCGT AGTTCTGACC ATAAACTATG CCAACTGGCA ATCCGCCGGA
GTTACTACAA TGACTCGGCG AGCAGCCCCC GGGAAACCAA AGTTTCTCGG TTCCGGGGGG
AGTATGGTTG CAAAGCTGAA ACTTAAAGGA ATTGACGGAA GGGCACCACC AGGAGTGGAG
CCTGTGGCTT AATTTGACTC AACACGGGAA ACCTCACCCG GCCCGGACAC CGTTAGGATT
GACAGATTGA GAGCTCTTTC TTGATTCGGT GGGTGGTGGT GCATGGCCGT TCTTAGTTGG
TGGAGCGATT TGTCTGGTTA ATTCCGATAA CGAACGAGAC TCTAGCCTAC TAAATAGTTC
GAGGATATAC AACCTCGCAA CTTCTTAGAG GGACAGGTGG CGTTTAGCCA CACGAGATTG
AGCAATAACA GGTCTGTGAT GCCCTTAGAT GTCCGGGGCC GCACACGCGC TACATTGAAT
GGATCAGCGT GCGTCTAGCC TTGCCCGAAA GGGCTGGGAA ACCCCTTGAA CCCCATTCGT
GATAGGGATT GGGGCTTGCA ATTATTTCCC ATGAACGAGG AATTCCTAGT AAGCGCGAGT
CATCAGCTCG CGTTGATTGC GTCCCTGCCC TTTGTACACA CCGCCCGTCG CTACTACCGA
TCGTTCCAGT TAATGAACAC TTCGGATTGG TTAAGCGGG- TTCTGCCTGC TCGCGTGCCG
AGAAGACTTG TGAATTATCT GGGATAGAGG TCGTAAAAGT CGTAACAAGG TATCCATTAA

TAAGCGGAGG AAAAGAGACT AACTAGGATT CCCCTAGTAA CGGCGAGTGA AGCGGGAAGA
GCCCAGCACC GAATCCCCCG ACGTCTGGCT GTCGATGGGA CATGTGGTGT TAGCGGGAGC
CTTGTGTCGG CGTGTTTCGG CACCAAAGTC CACTTGATCG TGGCAATCCA GGGCGGGTGA
GAGACCCGTT CCGGTGTCGA ACGCGTCGGA CTCTCTCCAG GAGTCGGGTT GTTTGAGAAT
GCAGCCCAAA GTGGGTGGTA AACTCCATCT AAGGCTAAAT ACAGACACGA GTCCGATAGA
GGACAAGTAC CGTGAGGGAA AGTTGAAAAG AACTTTGAAG AGAGAGTTCA AGAGTACGTG
AAACCGCATA GAGTCAAACG GGTGGATCCG CAAGGTGTTG GCCCGGGGAA TTCAGCGGTG
CACTTTCTCC GACGAGAGCC ACGACCGGGT TCGCTGCTCG CAGTGCGAGA AGGTATCCCC
TCGCGCTGGG AACGTATAGC TCGTCGGCCT CGAGTCTGCA TGTCGTGGAC CCGAGGACGC
GCCGCGTGCC CGGTCCCGGG CCGTCCGTGT GCGTTCGACC TCT---CGTA GAGTGCCGTA
ACTGCGTGTG GGCTCGGGTG CTAGGGTCAG TGGCGAATCA GTCGGTCCTC CACCCGACCC
GTCTTGAAAC ACGGACCAAG GAGTCTAACA TGTGCGCGAG TCATAGGGAC CCGAATCCCG
AAGGCGCAAT GAAAGTGAAG GCCGCCCTCG GCTGGCCGAG GCAGGATCCC GTCGCGGGCG
CACTGCCGGC CCGTCTCGAT AGCGGTGC-G GTGGA-CCGT CATTCGTCTA CGAGGCGGAG
CAAGAGCGTA CACGTTGGTA CCCGAAAGAT GGTGAACTAT GCCTGAGCAG GACGAAGCCA
GAGGAAACTC TGGTGGAGGT CCGCAGCGAT TCAAGAAAGT CCACTGGTGG CAAAGCCCCA
AGGAAACAGC TGGCCACAAA GGCTGCACGA AAGAGTGCAC CAGCCACTGG TGGCGTGAAG
AAGCCACACA GATACAGGCC TGGAACCGTC GCTCTCCGTG AGATCAGACG GTACCAGAAG
AGCACTGAAC TTCTGATCAG AAAGTTGCCC TTCCAGCGTC TGGTCCGTGA GATCGCTCAG
GACTTCAAGA CCGATCTCCG ATTCCAGAGC TCCGCTGTCA TGGCTCTGCA GGAGGCTAGT
GAAGCTTACC TTGTTGGGCT TTTTGAGGAC ACCAACTTGT GCGCCATCCA CGCCAAGCGT
CACTTTATAT TTTCTTTTTG GAATTTGATC TGGATTAGTT GGAACCTCTT TTAGAGTTTT
AATTCGATTA GAATTATCTC GACCGGGAGC ATTTTTAGGA GATGACCATT TATATAATGT
TATTGTTACT GCTCATGCAT TTGTAATAAT TTTTTTTTTA GTAATACCTA TAACTGTTGG
AGGTTTTGGA AATTGGATAG TTCCTTTAAT ATTAACATCC CCAGATATAG CATTTCCTCG
TATAAACAAT ATAAGGTTCT GGTTACTTCC TCCCGCTTTA TTTTTACTTT TAAGTTCAGC
TTTTGTTGAA AGAGGTGTGG GAACTGGTTG AACTGTTTAT CCTCCTTTAT CAGGGAATAT
TACTCATGGA GGCGGTTCAG TTGATTATGG AATTTTTTCT CTTCATTTAG CTGGTGTTTC
CTCAATTTTA GGAGCTATTA ATTTTTTAGC TACTACAATT AATATACGTC CTGAAATTAT
GGAGTTAAAG CGAGTTACTT TATTTGTTTG ATCAATTGCA TTAACTGCTT TTTTATTAGT
AGTAGCGATA CCTGTTTTAG CTGGTGCTAT TACTATATTA TTAACTGATC GAAATTTTAA
TACATCTTTT TTTGACCCTT CAGGAGGTGG AGATCCTATT CTTTTTGTTC ATTTATTTT

>Neaeromya_rugifera
TAGTCATATG CTCGTCTTAA AGATTAAGCC ATGCACGTCT AAGTGCATGC CACAGTAACG
GCGAAACTGC GAATGGCTCA TTAAATCAGT TATGGTTCCT TAGATCGTAT CACCCGACGT
GGATAACCGT GGCAATTCTA GAGCTAATAC ATGCTACAAA GCTCCAACCC CGGGGGAAGA
GCGCACTTGT TAGCAAAACC AATCCGGCCG GCTGTTGACT CTGAACAACT T--TGCCGAT
CGCACGGCCT TGCGCCTGCG ACGTATCTAT CGAATGTCTG CCCTATCAAC TGTCGATGGT
ACGTGCTATG CCTACCATGG TGTTGACGGG TAACGGGGAA TCAGGGTTCG ATTCCGGAGA
GGGAGCATGA GAAACGGCTA CCACATCCAA GGAAGGCAGC AGGCACGCAA ATTACCCACT
CCCGACACGG GGAGGTAGTG ACGAAAAATA ACAATACGCG ACTCCTAAAC GGTCGCGGAA
TTGGAATGAG TACACTTTAA ATCCTTTATC GAGGATCCAT TGGAGGGCAA GTCTGGTGCC
AGCAGCCGCG GTAATTCCAG CTCCAATAGC GTATATTAAA GTTGTTGCAG TTAAAAAGCT
CGTAGTTGGA TCTCGGCAGC AGGCGTGCGG CGCTCCTTGA GGCGCCTGCA CGTCCCGTCA
GCCTAGGGGC GGT-CCCGGC GCTGCTCTTC ACCGAGTGGC GGTGGCCGCC AGGTTTACTT
TGATGAAATT AGAGTGTTCA AAGCACGGGC AGTTGCCCGC ATAATTGTGC ATGGAATAAT
GGAATAGGAC GCCGTCGCTA TTTTGTTGGT TTGCCGAGAG GTAATGATTG ACAGGGACAG
CCGGGGGCAT TCGTATTGCA GGGGGAGAGG TGAAATTCTG AGATCCTTGC AAGACGACCG
AGAGCGAAAG CACTTGCCAA GAGTGTCTTC ATTAATCAAG AACGAAAGTC AGAGGCTCGA

AGACGATCAG ATACCGTCGT AGTTCTGACC ATAAACCATG CCAACTGGTA ATCCGCCGCA
GTTACTACAA TGACTCGGCG TGCAGCCCCC GGGAAACCAA AGTGTCGTGG TTCCGGGGGC
AGTATGGTTG CAAAGCTGAA ACTTAAAGGA ATTGACGGAA GGGCACCACC AGGAGTGGAG
CCTGTGGCTT AATTTGACTC AACACGGGAA ACCTCACCCG GTTCGGACAC CGAAAGGATT
GACAGATTGA GAGCTCTTTC TTGATTCGGT GAGTGGTGGT GCATGGCCGT TCTTAGTTCG
TGGAGCGATT TGTCTGGTTA ATTCCGATAA CGAACGAGAC TCTAGCCTGC TAACTAGTTC
GGGTCTACGC GGCGTAGCGA CTTCTTAGAG GGACAGGTGG CAAACAGCCA CATGAGATTG
AGCAATAACA GGTCTGTGAT GCCCTTAGAT GTCCGGGGCC GCACACGCGC TACATTGAAT
GGAGCAACGT GCGGCTCGCC TCGCCCGAAA GGGTAGGGAA ATCACTGGAA ACCCATTCTC
GGCAGGGACT GGGGTTTGCA ATTGTTTCCC ACGAACGAGG AATCCCTCGT AAGCGCGAGT
CATTAGCTCG CGCTGATTAC GTCCCTGCCC TTTGTACACA CCGCCCGTCG CTACTACCGA
TCGTTCCGGT CAATGAACGC CTCGGATTGG CTGTCCG--- -TC-ACCCGC TCGTGCGCCG
AGAAGACGCG TGAACTGTCC GGGATAGAGG TCGTAAAAGT CGTAACAAGG TATCCATTAA
TAAGCGGAGG AAAAGAAACT AACTAGGATT CCCCTAGTAA CGGCGAGTGA AGCGGGATTA
GCCCAGCACC GAATCCCCAG GCGACTGGCC GCCCACGGGA CATGTGGTGT TAGCGGGAGT
CTCTAGTCGG CGCTGTACGG CATCAAAGTC CACCTGATCG TGGCAACCCA GTGCGGGTGA
GAGGCCCGTG CAGGTGTCGT ACGCGTCGGA CCCTCTCCAG GAGTCGGGTT GTTTGAGAGT
GCAGCCCAAA GTGGGTGGTA AACTCCATCT AAGGCTAAAT ACGAACACGA GTCCGATAGA
GGACAAGTAC CGTGAGGGAA AGTTGAAAAG AACTTTGAAG AGAGAGTTCA AGAGTACGTG
AAACCGCATA GAGTTAAACG GGTGGACCCG CAA-GTGCTG GTCCGGGGAA TTCAGCGGTG
CACTTTCTCC GAAACTAGCC ATGACCGGGC GCACTGCACG GAGCGCAGGA AGGTTTCCGA
ACGCCTC-GG AGCTTACAGC CTGCCGCTCG ACATCGTGTA CGTCACGCGC CCGAGGACGC
GCATCGTGCT CTTGCCCGTG TCGCTCTCGT GCGGTCGACC TTCGTCAGCA GGGTGCCGTA
ACCGCGTGCC GCGGCGTGGG CTAGGGTCAA TGGCTAAGCC GTCGGTACTC CACCCGACCC
GTCTTGAAAC ACGGACCAAG GAGTCTAACA TGTGCGCGAG TCAAAGGGAC TCGAATCCCC
ATGGCGCAAT GAAAGTGAAG GTCGGACTCG TTCGACCGAG GCAAGATCCC TTCGGTGGCG
CACTGCCGGC CCGTCTCGTT AG-GGCGCGC GCGGGGGTGG CGTGCGTCTG AGAGGCGGAG
CAAGAGCGTA CACGTTGGTA CCCGAAAGAT GGTGAACTAT GCCCGAGCAG GACGAAGTCA
GAGGAAACTC TGATGGAGGT CCGCAGCGAT TC???????? ?????????? ??????????
?????????? ?????????? ?????????? ?????????? ?????????? ??????????
?????????? ?????????? ?????????? ?????????? ?????????? ??????????
?????????? ?????????? ?????????? ?????????? ?????????? ??????????
?????????? ?????????? ?????????? ?????????? ?????????? ??????????
?????????? ?????????? ?????????? ?????????? ?????????? ??????????
TACTTTATAT TTTTTATTTG GAATTTGAAG TGGTTTGGTT GGTACTTCTT TTAGAGTTTT
GATTCGATTA GAGCTTTCTC GTCCAGGGGC TTTTTTAGGG GATGATCATT TATATAACGT
TATTGTGACT GCTCATGCTT TTGTTATAAT TTTTTTCTTA GTAATACCTA TAATAGTAGG
TGGTTTTGGA AATTGACTAG TTCCTTTAAT GCTAACTTCT GTTGATATAG CTTTTCCTCG
AATAAACAAT ATAAGTTTTT GGCTTTTGCC TCCTGCTTTA TTTTTACTTT TGAGCTCGGC
TTTTGTAGAA AGTGGGGTTG GTACTGGATG GACAGTCTAT CCACCTCTTT CTTCAAATAT
CACACATAGG GGAGGCTCAG TTGATTATGG TATTTTTTCT TTACATTTAG CTGGTGTTTC
TTCTATCCTT GGTGCTATTA ATTTTTTGGC TAGGACTGTG AATATACGTC CTGAAATCAT
GGAATTTAAA CGGGTTACTT TATTTGTGTG GTCGATCGCT ATTACTGCTT TTTTACTTGT
TGTTGCAATA CCTGTTTTAG CTGGTGCTAT CACTATGCTT TTAACTGATC GAAATTTTAA
TACTTCTTTT TTTGATCCTT CAGGTGGTGG TGACCCTATT TTATTTGTTC ATTTATTTT

>Nipponomontacuta_actinariophila
TAGTCATATG CTTGTCTCAA AGATTAAGCC ATGCATGTCT AAGTACACGC CAGATTAATG
GTGAAACTGC GAATGGCTCA TTAAATCAGT TATGGTTCCT TAGATCGTAC AATCCTACTT
GGATAACTGT GGCAATTCTA GAGCTAATAC ATGCGTCAAA GCTCCGACCT TCGGGGAAGA

GCGCTTTTGT TAGCAAGACC AATCCGGTCG GTTGTTGACT CTGAACAACT TTGTGCTGAT
CGCACGGCCT AGCGCCGGCG ACGTATCTTT CGAATGTCTG CCCTATCAAC TGACGATGGT
ACGTGCTATG CCTACCATGG TTGTAACGGG TAACGGGGAA TCAGGGTTCG ATTCCGGAGA
GGGAGCATGA GAAACGGCTA CCACATCCAA GGAAGGCAGC AGGCGCGCAA ATTACCCAAT
CCCGACACGG GGAGGTAGTG ACGAAAAATA ACAATACGGG ACTCTTTCGA GGCCCCGTAA
TTGGAATGAG TACACTTTAA ATCCTTTAAC GAGGATCCAT TGGAGGGCAA GTCTGGTGCC
AGCAGCCGCG GTAATTCCAG CTCCAATAGC GTATATTAAA GTTGCTGCAG TTAAAAAGCT
CGTAGTTGGA TCTCGGGTGT AGGCTTGCGG TCCGCCTCGC GGCGGCTGCT CGTCCTGACA
GCCTAGCTTC GGTGTCCCTT GGTGCTCTTG ACTGAGTGTC GGTGGCCGGA ACGTTTACTT
TGATGAAATT AGAGTGTTTA AAGCGTGGGC GTTTGCCCGT ATAATGGTGC ATGGAATGAT
AGAATAGGAC CTCGGTTCTA TTTTGTTGGT TTTCGGAGAG GTAATGATTG AGAGGGACAG
ACGGGGGCAT TCGTATTGCA GCGTTAGAGG TGAAATTCTT GGATCGTTGC AAGACGGCCG
ACAGCGAAAG CATTTGCCAA GAATGTTTTC CTTAATCAAG AACGAAAGTC AGAGGCTCGA
AGACGATCAG ATACCGTCGT AGTTCTGACC ATAAACTATG CCAACTGGCA ATCCGCCGGA
GTTACTACAA TGACTCGGCG AGCAGCCCCC GGGAAACCAA AGTTTCTCGG TTCCGGGGGG
AGTATGGTTG CAAAGCTGAA ACTTAAAGGA ATTGACGGAA GGGCACCACC AGGAGTGGAG
CCTGTGGCTT AATTTGACTC AACACGGGAA ACCTCACCCG GCCCGGACAC CGTTAGGATT
GACAGATTGA GAGCTCTTTC TTGATTCGGT GGGTGGTGGT GCATGGCCGT TCTTAGTTGG
TGGAGCGATT TGTCTGGTTA ATTCCGATAA CGAACGAGAC TCTAGCCTAC TAAATAGTTC
GAGGATATAC AACCTCGCAA CTTCTTAGAG GGACAGGTGG CGTTTAGCCA CACGAGATTG
AGCAATAACA GGTCTGTGAT GCCCTTAGAT GTCCGGGGCC GCACACGCGC TACATTGAAT
GGATCAACGT GCGTCTAGCC TTGCCCGAAA GGGCTGGGAA ACCCGTTGAA CCCCATTCGT
GATAGGGACT GGGGCTTGCA ATTATTTCCC ATGAACGAGG AATTCCTAGT AAGCGCGAGT
CATCAGCTCG CGTTGATTGC GTCCCTGCCC TTTGTACACA CCGCCCGTCG CTACTACCGA
TCGTTCCAGT TAATGAACAC CTCGGATTGG TTAAGCGGGT TTCGGCCTGC TCGCGTGCCG
AGAAGACTTG TGAATTATCT GGGATAGAGG TCGTAAAAGT CGTAACAAGG TATCCATTAA
TAAGCGGAGG AAAAGAGACT AACTAGGATT CCCCTAGTAA CGGCGAGTGA AGCGGGAAAA
GCCCAGCACC GAATCCCCCG GCATCTGGCT GCCGACGGGA CATGTGGTGT TAGCGGGAGC
CTTATGTCGG CGTGTTTCGG CACCAAAGTC CACTTGATCG TGGCAATCCA TGGCGGGTGA
GAGACCCGTT CCGGTGTCGA GCGCGTCGGA CTCTCTCCAG GAGTCGGGTT GTTTGAGAAT
GCAGCCCAAA GTGGGTGGTA AACTCCATCT AAGGCTAAAT ACTGACACGA GTCCGATAGA
GGACAAGTAC CGTGAGGGAA AGTTGAAAAG AACTTTGAAG AGAGAGTTCA AGAGTACGTG
AAACCGCATA GAGTCAAACG GGTGGATCCG CAAGGTGTTG GCCCGGGGAA TTCAGCGGTG
CACTTTCTCC GACGAGAGCC ACGACCGGGT CAGCTGCTCG CAGTGCGAGA AGGTTTTTCC
GGGCGTTGAG AGCTTACAGC TCGTCGGCCT CGGGTCTGTA TGTTGCCGAC CCGAGGACGC
GCCGCGCGTC CGGCTCCGGG CCGTCCGTGT GCGTTCGACC TTT---CGTA GAGTGCAGTA
ACCGCGCGCG GGCTCGGGTG CTAGGGTCAG TGGCGAATCA GTCGGTCCTC CACCCGACCC
GTCTTGAAAC ACGGACCAAG GAGTCTAACA TGTGCGCGAG TCATAGGGAC CCGAATCCCG
AAGGCGCAAT GAAAGTGAAG GCCGCCTACG GCTGGCCGAG GCAGGATCCC GTCGCGGGCG
CACTGCCGGC CCGTCTCGAT AGCGGCGC-G GCGTG-CCGT CGTTCGTCTA CGAGGCGGAG
CAAGAGCGTA CACGTTGGTA CCCGAAAGAT GGTGAACTAT GCCTGAGCAG GACGAAGTCA
GAGGAAACTC TGATGGAGGT CCGCAGCGAT TCTCGAAAAT CAACTGGTGG CAAGGCCCCA
CGAAAGCAGC TGGCCACCAA GGCCGCTCGT AAGAGTGCCC CTGCCACTGG CGGCGTCAAG
AAGCCACACA GATACAGGCC TGGAACCGTC GCCCTCCGTG AGATCAGAAG GTACCAGAAG
AGCACCGAGC TGCTGATCAG AAAATTGCCC TTCCAGCGTC TCGTCCGTGA GATCGCCCAG
GACTTCAAGA CCGATCTCCG ATTCCAGAGC TCTGCCGTCA TGGCTCTCCA GGAGGCCAGC
GAGGCTTACC TGGTCGGTCT GTTCGAGGAC ACCAACTTGT GCGCTATCCA CGCCAAGCGT
TACTTTGTAT TTTATTTTTG GAATCTGGTC TGGTATAGTG GGGACATCTT TTAGAGTTTT
AATTCGTCTA GAATTATCTC GTCCGGGTGC TTTTTTAGGA GATGATCATT TGTATAACGT
TATCGTAACA GCTCACGCTT TCGTTATAAT TTTTTTTTTA GTAATACCAA TAATGGTAGG

GGGATTTGGA AATTGATTAG TTCCTTTAAT GTTAACTTCT CCTGATATGG CTTTTCCTCG
TATAAATAAC ATAAGGTTTT GGCTTTTGCC GCCTGCTTTG TTTTTACTCT TGAGATCTGC
TTTTGTAGAA AGAGGCGTTG GGACTGGGTG GACTGTTTAT CCCCCGCTTT CAAGTAATGT
AACACATAGA GGGGGTTCTG TGGATTATGG AATTTTTTCT TTACATTTAG CAGGTGTTTC
TTCTATTTTG GGCGCAATTA ACTTTTTAGC AACAACTGTA AATATACGTC CCGAAATTAT
AGAATTAAAA CGTGTTACTT TATTTGTGTG GTCTATCGCT ATTACCGCTT TCTTGTTGGT
AGTGGCTATG CCAGTTTTAG CTGGGGCTAT CACAATATTA TTGACCGATC GTAATTTTAA
TACATCATTC TTTGATCCTT CAGGTGGAGG TGATCCTATT CTTTTTGTGC ATTTGTTTT

>Nipponomysella_oblongata
TAGTCATATG CTTGTCTCAA AGATTAAGCC ATGCATGTCT AAGTACACGC CAGATTCATG
GTGAAACTGC GAATGGCTCA TTAAATCAGT TATGGTTCCT TAGATCGTAC AATCCTACTT
GGATAACTGT GGCAATTCTA GAGCTAATAC ATGCGTCAAA GCTCCGACCT TCGGGGAAGA
GCGCTTTTGT TAGCAAGACC AATCCGGTCG GTTGTTGACT CTGAACAACT TTGTGCTGAT
CGCACGGCCT AGCGCCGGCG ACGTATCTTT CGAATGTCTG CCCTATCAAC TGACGATGGT
ACGTGCTATG CCTACCATGG TTGTAACGGG TAACGGGGAA TCAGGGTTCG ATTCCGGAGA
GGGAGCATGA GAAACGGCTA CCACATCCAA GGAAGGCAGC AGGCGCGCAA ATTACCCAAT
CCCGACACGG GGAGGTAGTG ACGAAAAATA ACAATACGGG ACTCTTTCGA GGCCCCGTAA
TTGGAATGAG TACACTTTAA ATCCTTTAAC GAGGATCCAT TGGAGGGCAA GTCTGGTGCC
AGCAGCCGCG GTAATTCCAG CTCCAATAGC GTATATTAAA GTTGCTGCAG TTAAAAAGCT
CGTAGTTGGA TCTCGGGTGT AGGCTTGCGG TCCGCCTCGC GGCGGCTGCT CGTCCTGACA
GCCTAGCTTC GGTGTCCCTT GGTGCTCTTG ATTGAGTGTC GGTGGCCGGA ACGTTTACTT
TGATGAAATT AGAGTGTTTA AAGCGTGGGC GTTTGCCCGT ATAATGGTGC ATGGAATGAT
AGAATAGGAC CTCGGTTCTA TTTTGTTGGT TTTCGGAGAG GTAATGATTG AGAGGGACAG
ACGGGGGCAT TCGTATTGCA GCGTTAGAGG TGAAATTCTT GGATCGTTGC AAGACGGCCG
ACAGCGAAAG CATTTGCCAA GAATGTTTTC CTTAATCAAG AACGAAAGTC AGAGGCTCGA
AGACGATCAG ATACCGTCGT AGTTCTGACC ATAAACTATG CCAACTGGCA ATCCGCCGGA
GTTACTACAA TGACTCGGCG AGCAGCCCCC GGGAAACCAA AGTTTCTCGG TTCCGGGGGG
AGTATGGTTG CAAAGCTGAA ACTTAAAGGA ATTGACGGAA GGGCACCACC AGGAGTGGAG
CCTGTGGCTT AATTTGACTC AACACGGGAA ACCTCACCCG GCCCGGACAC CGTTAGGATT
GACAGATTGA GAGCTCTTTC TTGATTCGGT GGGTGGTGGT GCATGGCCGT TCTTAGTTGG
TGGAGCGATT TGTCTGGTTA ATTCCGATAA CGAACGAGAC TCTAGCCTAC TAAATAGTTC
GAGGATATAC AACCTCGCAA CTTCTTAGAG GGACAGGTGG CGTTTAGCCA CACGAGATTG
AGCAATAACA GGTCTGTGAT GCCCTTAGAT GTCCGGGGCC GCACACGCGC TACATTGAAT
GGATCAACGT GCGTCTAGCC TTGCCCGAAA GGGCTGGGAA ACCCGTTGAA CCCCATTCGT
GATAGGGACT GGGGCTTGCA ATTATTTCCC ATGAACGAGG AATTCCTAGT AAGCGCGAGT
CATCAGCTCG CGTTGATTGC GTCCCTGCCC TTTGTACACA CCGCCCGTCG CTACTACCGA
TCGTTCCAGT TAATGAACAC TTCGGATTGG TTAAGCGGGT TTCGGCCTGC TCGCGTGCCG
AGAAGACTTG TGAATTATCT GGGATAGAGG TCGTAAAAGT CGTAACAAGG TATCCATTAA
TAAGCGGAGG AAAAGAGACT AACTAGGATT CCCCTAGTAA CGGCGAGTGA AGCGGGAAGA
GCCCAGCACC GAATCCCCCG GCCTCTGGCG GCCGACGGGA CATGTGGTGT TAGTGGGAGC
TTTGTGTCGG CGTGTTTCGG CACCAAAGTC CACTTGATCG TGGCAATCCA TGGCGGGTGA
GAGACCCGTT CCGGTGTCGA ACGCGTCGGA CTCTCTCCAG GAGTCGGGTT GTTTGAGAAT
GCAGCCCAAA GTGGGTGGTA AACTCCATCT AAGGCTAAAT ACTGACACGA GTCCGATAGA
GGACAAGTAC CGTGAGGGAA AGTTGAAAAG AACTTTGAAG AGAGAGTTCA AGAGTACGTG
AAACCGCATA GAGTCAAACG GGTGGATCCG CAAGGTGTTG GCCCGGGGAA TTCAGCGGTG
CACTTTCTCC GACGAGAGCC ACGACCGGGT CTGCTGCTCG CAGCGCGAGA AGGTTTCCCC
GCGCGCTGGG AGCATACAGC TCGTCGGCCT CGAGTCTATA TGTCGCGGAC CCGAGGACGC
GCCGCGCGTC CGGCTCCGGG CCGTCCGTGT GCGTTCGACC T-TCTTCGTA GAGTGCCGTA
ACTGCGTGTG GGCTCGGGTG CTAGGGTCAG TGGCGAATCA GTCGGTACTC CACCCGACCC

GTCTTGAAAC ACGGACCAAG GAGTCTAACA TGTGCGCGAG TCATAGGGAC CCGAATCCCG
AAGGCGCAAT GAAAGTGAAG GCCGCCTACG GCTGGCCGAG GCAGGATCCC GTCGCGGGCG
CACTGCCGGC CCGTCTCGAT AGCGGTGTAG CAGGTGCCAA CATTCGTCTA CGAGGCGGAG
CAAGAGCGTA CACGTTGGTA CCCGAAAGAT GGTGAACTAT GCCTGAGCAG GACGAAGTCA
GAGGAAACTC TGATGGAGGT CCGCAGCGAT TCTAGAAAGT CCACTGGTGG CAAGGCCCCA
CGAAAACAAT TGGCCACCAA GGCCGCACGT AAGAGTGCGC CTGCCACTGG TGGTGTCAAG
AAACCACACA GATACAGGCC TGGAACCGTC GCTCTCCGTG AGATCAGAAG GTACCAGAAG
AGCACCGAGC TCCTGATCAG AAAATTGCCC TTCCAGCGCC TGGTCCGCGA GATNNNNNNN
GACTTCAAGA CTGATCTCCG ATTCCAGAGC TCCGCTGTCA TGGCTCTCCA GGAGGCTAGC
GAGGCTTACC TCGTCGGTCT TTTCGAGGAC ACCAACCTGT GCGCTATCCA CGCCAAGCGT
TAGTCTTTAT TTTATTTTTG GTATTTGGTC TGGGATGGTT GGAACTTCTT TTAGGGTTTT
AATTCGTTTA GAATTATCAC GGCCCGGTGC TTTTTTAGGA GACGATCATT TGTATAATGT
TATTGTAACG GCTCACGCCT TCGTTATAAT TTTTTTTTTA GTAATACCTA TAATAGTAGG
GGGGTTTGGG AATTGGTTGG TTCCTTTAAT ATTAACTTCT CCTGACATGG CTTTTCCTCG
TATGAATAAT ATAAGGTTTT GGCTTCTTCC TCCCGCTTTA TTTTTACTTT TAAGATCTGC
TTTTGTAGAG AGAGGGGTAG GAACTGGGTG AACAGTTTAT CCTCCTTTAT CAAGAAATGT
GACTCACAGA GGGGGTTCTG TGGACTATGG AATTTTTTCT CTCCATTTAG CAGGTGTATC
TTCGATTTTA GGAGCTATTA ATTTTCTTGC GACAACAGTA AATATACGGC CTGAAATTAT
GGAGTTAAAA CGTGTTACAT TATTTGTGTG GTCAATTGCT ATCACTGCTT TTTTGCTTGT
AGTAGCAATA CCTGTTTTAG CTGGGGCTAT TACTATACTA TTAACTGATC GTAATTTTAA
TACTTCTTTT TTTGATCCTT CAGGGGGGGG CGATCCAATT TTGTTT---- ---------

>Nipponomysella_subtruncata
TAGTCATATG CTTGTCTCAA AGATTAAGCC ATGCATGTCT AAGTACACGC CAGATCAACG
GTGAAACTGC GAATGGCTCA TTAAATCAGT TATGGTTCCT TAGATCGTAC AATCCTACTT
GGATAACTGT GGCAATTCTA GAGCTAATAC ATGCATCTAA GCTCCGACCC TTGGGGAAGA
GCGCTTTTGT TAGCAAAACC AATCCGGCCG GTTGTTGACT CTGAACAACT TTGTGCCGAT
CGCACGGCCT AGCGCCGGCG ACGTATCTTT CGAATGTCTG CCCTATCAAC TGACGATGGT
ACGTGCTATG CCTACCATGG TTGTAACGGG TAACGGGGAA TCAGGGTTCG ATTCCGGAGA
GGGAGCATGA GAAACGGCTA CCACATCCAA GGAAGGCAGC AGGCGCGCAA ATTACCCAAT
CCCGACACGG GGAGGTAGTG ACGAAAAATA ACAATACGGG ACTCTTTCGA GGCCCCGTAA
TTGGAATGAG TACACTTTAA ATCCTTTAAC GAGGATCCAT TGGAGGGCAA GTCTGGTGCC
AGCAGCCGCG GTAATTCCAG CTCCAATAGC GTATATTAAA GTTGCTGCAG TTAAAAAGCT
CGTAGTTGGA TCTCGGGTGC AGGCTTGCGG TCCGCCTAAC GGTGGCTGCT CGTCCTGGCA
GCCTAGCTGC GGTAACCCTT GGTGCTCTTT ACCGAGTGTC GGTTGCCGTA ACGTTTACTT
TGATGAAATT AGAGTGTTCA AAGCGCGGGC GTTTGCCCGC ATAATGGTGC ATGGAATAAT
GGAATAGGAC CCCGGTTCTA TTTTCATGGT TTTGAGAGTG GTAATGATTG AGAGGGACGG
CCGGGGGCAT TCGTATTGCG GCGTTAGAGG TGAAATTCTT GGATCGCCGC AAGACGGTCG
AGAGCGAAAG CATTTGCCAA GAATGTTTTC CTTAATCAAG AACGAAAGTC AGAGGCTCGA
AGACGATCAG ATACCGTCGT AGTTCTGACC ATAAACCATG CCAACTGGCA ATCCGCCGGA
GTTACTACAA TGACTCGGCG AGCAGCCCCC GGGAAACCAA AGTTTCTGGG TTCCGGGGGG
AGTATGGTTG CAAAGCTGAA ACTTAAAGGA ATTGACGGAA GGGCACCACC AGGAGTGGAG
CCTGTGGCTT AATTTGACTC AACACGGGGA ACCTCACCCG GCCCGGACAC CGTAAGGATT
GACAGATTGA GAGCTCTTTC TTGATTCGGT GGGTGGTGGT GCATGGCCGT TCGTAGTTGG
TGGAGCGATT TGTCTGGTTA ATTCCGATAA CGAACGAGAC TCTAGCCTAC TAAATAGTTC
GGGGCTGTAT AGCCCCGCAA CTTCTTAGAG GGACAGGTGG CGTATAGCCA CACGAGATTG
AGCAATAACA GGTCTGTGAT GCCCTTAGAT GTTCGGGGCC GCACACGCGC TACATTGAAT
GGATCAACGT GCGTCTAGCC TTGCCCGAAA GGGCTGGGAA ACCCGTTGAA ACCCATTCGT
GATAGGGACT GGGGCTTGCA ATTATTTCCC ATGAACGAGG AATTCCTAGT AAGCGCGAGT
CATCAGCTCG CGTTGATTGC GTCCCTGCCC TTTGTACACA CCGCCCGTCG CTACTACCGA

TCGCTCCAGT TAATGAACGC TTCGGATTGG TCAAGCGGGT TTCGGCATGC TCGCGTGCCG
AGAAGATGTG TGAATTATCC GGGGTAGAGG TCGTAAAAGT CGTAACAAGG TATCCATTAA
TAAGCGGAGG AAAAGAGACT AACTAGGATT CCCCTAGTAA CGGCGAGTGA AGCGGGAAGA
GCCCAGCACC GAATCCCCCA GCTTCTGGCA GCTGACGGGA CATGTGGTGT TAGCGGGAGC
CCCACGTCGG CGTGTCCGGT CGGCCAAGTC CACCTGATCG TGGCGACCCC AGGCGGGTGA
GAGGCCCGTT TTGGCGACCG GCGCGTCGGA CTCTCTCCAG GAGTCGGGTT GTTTGAGAAT
GCAGCCCAAA GTGGGTGGTA AACTCCATCT AAGGCTAAAT ACTGACACGA GTCCGATAGA
GGACAAGTAC CGTGAGGGAA AGTTGAAAAG AACTTTGAAG AGAGAGTTCA AGAGTACGTG
AAACCGCATA GAGGCAAACG GGTGGACCCG CAAGGTGTTG GACCGGGGAA TTCAGCGGCG
CACTTTCTCC GACGAGAGCC ACGACCGGGA TTGCTGCCCG AGGCGCGACA AGGTTTTCCG
GGGTTCGGGG AGCTTACAGG TCGCCAACCT CGGGCCGGCA C---GCGATC CCGAGGACGC
GCCGTGCGCT CGGC-CCGGG CCGTCCTGGC GTGTTCGACT T--TCGTGCA GTGTGCCGAG
ACCGCTCGCC GGCTCGGGTG TGAGGGTCTG TGGCGAATCA GTCGGTACTC CACCCGACCC
GTCTTGAAAC ACGGACCAAG GAGTCTAACA TGTGCGCGAG TCGTAGGGAC CCGAATCCCG
AAGGCGAAAT GAAAGTGAAG GTCGGCCTAG GCCGTCCGAG GCAGGATCCC ATCACTGGCG
CACTGCCGGC CCGTCTCGCT AGCGCGCC-- --GGGGCT-- --GTCGTCTG CGAGGCGGAG
CAAGAGCGTA CACGTTGGTA CCCGAAAGAT GGTGAACTAT GCCTGAGCAG GACGAAGTCA
GAGGAAACTC TGATGGAGGT CCGCAGCGAT TCCCGAAAGT CCACCGGAGG AAAGGCTCCC
AGGAAACAGC TGGCCACTAA GGCAGCAAGA AAGAGTGCCC CAGCCACCGG TGGCGTGAAG
AAGCCCCACA GGTACAGGCC CGGCACCGTG GCTCTCCGTG AGATCAGGAG GTACCAGAAG
AGCACCGAGC TCCTGATCAG GAAGCTGCCA TTCCAGCGTC TTGTGCGCGA GATCGCCCAG
GACTTCAAGA CCGACCTCCG CTTCCAGAGC TCCGCCGTCA TGGCGCTGCA GGAGGCCAGC
GAGGCCTACC TGGTCGGTCT CTTCGAGGAC ACCAACCTGT GCGCCATCCA CGCCAAGCGT
---------- ---------- ---------- ---------- ---ACTTCTT TTAGTGTTCT
AATTCGTTTG GAGTTGTCTC GACCGGGGGC CTTTTTAGGT GATGATCATT TGTATAATGT
TATCGTTACT GCTCATGCTT TTGTTATGAT TTTTTTCCTG GTTATGCCTA TAATGGTGGG
GGGTTTTGGT AACTGGCTTG TGCCGTTGAT GCTGACGTCG CCGGACATAG CTTTTCCTCG
GATGAACAAT ATAAGGTTTT GGTTACTTCC TCCAGCACTT TTTCTTCTTC TTAGTTCAGC
ATTTGTGGAG AGGGGGGCAG GAACTGGGTG AACTGTATAC CCGCCTTTAT CTAGTAATAT
TACTCATAGG GGTGGGTCGG TTGATTATGC CATTTTTTCT CTTCATTTAG CGGGTGTTTC
TTCAATTCTT GGTGCTATTA ATTTTTTAGC TAGCACTATT AATATGCGCC CAGAGGTGAT
GGAGTTAAAG CGTGTAACTT TATTTGTCTG ATCAATTGCC ATCACGGCAT TCTTGTTGGT
GGTGGCGATA CCGGTGTTGG CGGGTGCTAT TACAATATTA CTTACTGATC GGAACTTCAA
CACATCTTTT TTCGACCCTT CA-------- ---------- ---------- ---------

>Parabornia_matsumotoi
TAGTCATATG CTTGTCTCAA AGATTAAGCC ATGCATGTCT AAGTACACGC CACTGTAAAG
GTGAAACTGC GAATGGCTCA TTAAATCAGT TATGGTTCCT TAGATCGTAC AATCCTACTT
GGATAACCGT GGCAATTCTA GAGCTAATAC ATGCGTCAAA GCTCCGACCT TCGGGGAAGA
GCGCTTTTGT TAGCAAAACC AATCCGGTCG GTTGTTGACT CTGGACAACT TTGTGCTGAT
CGCACGGCCT AGCGCCGGCG ATGTATCTTT CGAATGTCTG CCCTATCAAC TGTCGATGGT
ACGTGCTATG CCTACCATGG TTGTAACGGG TAACGGGGAA TCAGGGTTCG ATTCCGGAGA
GGGAGCATGA GAAACGGCTA CCACATCCAA GGAAGGCAGC AGGCGCGCAA ATTACCCAAT
CCCGACACGG GGAGGTAGTG ACGAAAAATA ACAATACGGG ACTCTTTCGA GGCCCCGTAA
TTGGAATGAG TACACTTTAA ATCCTTTAAC GAGGATCCAT TGGAGGGCAA GTCTGGTGCC
AGCAGCCGCG GTAATTCCAG CTCCAATAGC GTATATTAAA GTTGTTGCAG TTAAAAAGCT
CGTAGTTGGA TCTCGGGTGC AGGCTTGCGG TCCGCCTCGC GGCGGCTGCT CGTCCTGGCA
GCCTAGCTGC GGTATCCCTT GGTGCTCTTG ATTGAGTGTC GGTGGCCGGA ACGTTTACTT
TGATGAAATT AGAGTGTTCA AAGCACGGGC GCTTGCCCGC ATAATGGTGC ATGGAATGAT
AGAATAGGAC CTCGGTTCTA TTTTGTTGGT TTTCGGAGAG GTAATGATTG AGAGGGACAG

ACGGGGGCAT TCGTATTGCG GCGTTAGAGG TGAAATTCTT GGATCGCCGC AAGACGGACT
ACAGCGAAAG CATTTGCCAA GAATGTTTTC CTTAATCAAG AACGAAAGTC AGAGGCTCGA
AGACGATCAG ATACCGTCGT AGTTCTGACC ATAAACCATG CCAACTGGCA ATCCGCCGGA
GTTACTACAA TGACTCGGCG AGCAGCCCCC GGGAAACCAA AGTTTCTGGG TTCCGGGGGG
AGTATGGTTG CAAAGCTGAA ACTTAAAGGA ATTGACGGAA GGGCACCACC AGGAGTGGAG
CCTGTGGCTT AATTTGACTC AACACGGGAA ACCTCACCCG GCCCGGACAC CGTTAGGATT
GACAGATTGA GAGCTCTTTC TTGATTCGGT GGGTGGTGGT GCATGGCCGT TCTTAGTTGG
TGGAGCGATT TGTCTGGTTA ATTCCGATAA CGAACGAGAC TCTAGCCTGC TAAATAGTTC
GAGGATATAT AACCTCGCAA CTTCTTAGAG GGACAGGTGG CGTATAGCCA CATGAGATTG
AGCAATAACA GGTCTGTGAT GCCCTTAGAT GTCCGGGGCC GCACACGCGC TACATTGAAT
GGATCAACGT GCGTCTAGCC TTGCCCGAAA GGGCTGGGAA ACCCGTTGAA ACCCATTCGT
GATAGGGACT GGGGCTTGCA ATTATTTCGC ATGAACGAGG AATTCCTAGT AAGCGCGAGT
CATCAGCTCG CGTTGATTGC GTCCCTGCCC TTTGTACACA CCGCCCGTCG CTACTACCGA
TCGCTCCAGT TAATGAACGC CTCGGATTGG TTAAGCGGGT TTCGGCCTGC TCGCGTGCCG
AGAAGACGTG TAAATTATCT GGAGTAGAGG GAGTAAAAGT CGTAACAAGG TATCCATCAA
TAAGCGGAGG AAAAGAGACT AACTAGGATT CCCCTAGTAA CGGCGAGTGA AGCGGGAAGA
GCCCAGCACC GAATCCCCCG GCGTCTGGCC GCCGACGGGA CCTGTGGTGT TAGCGGGAGT
CTTTTGTCGG CGTGCTTCGG CACCAAAGTC CACCTGATCG TGGCAACCCC TGGCGGGTGA
GAGGCCCGTT CCGGTGTCGG ACGCGCTCGA CTCTCTCCAG GAGTCGGGTT GTTTGAGAAT
GCAGCCCAAA GTGGGTGGTA AACTCCACCT AAGGCTAAAT ACAGACACGA GTCCGATAGA
GGACAAGTAC CGTGAGGGAA AGTTGAAAAG AACTTTGAAG AGAGAGTTCA AGAGTACGTG
AAACCGCATA GAGGCAAACG GGTGGATCCG CAAGGTGTTG GACCGGGGAA TTCAGCGGTG
CACTTTCTCC GACGAGAGCC ACGACCGGGT CTGCTGCTCG AGGTGTGCGA AGGTTTCCAG
GCGTCTCGGG AGCTTATAGC GCACCGTCCT CGAGTCTGTA TGTCGCGGGC CCGAGGACGC
GCCGCGCGTT GGGCACCGGG TCGTCTTCGT GCGTTCGACT GTTCTCGGCA GTGCGTCGCG
ACCGCGCGCG GTTCCGGCG- TGAGGGTCGG TGGCGAATCA GTCGGCACTC CACCCGACCC
GTCTTGAAAC ACGGACCAAG GAGTCTAACA TGTGCGCGAG TCATGGGGAC CCGAATCCCG
AAGGCGCAAT GAAAGTGAAG GCCGCCCTCG GCTGGCCGAG GCAGGATCCC GTCGTGGGCG
CACTGCCGGC CCGTCTCTAT AGCGGCGCGG TCGCGGCCTG CGTTCGTCTA CGAGGCGGAG
CAAGAGCGTA CACGTTGGTA CCCGAAAGAT GGTGAACTAT GCCTGAGCAG GACGAAGTCA
GAGGAAACTC TGATGGAGGT CCGCAGCGAT TCTAGAAAGT CCACTGGTGG GAAAGCCCCC
AGGAAACAGT TGGCCACAAA GGCTGCACGT AAAAGTGCAC CTGCCACTGG TGGTGTGAAG
AAGCCACACA GATACAGGCC CGGAACTGTA GCTCTCCGTG AAATCAGGAG ATACCAGAAG
AGCACTGAAC TTCTCATCCG AAAACTGCCA TTCCAGCGTC TCGTCCGTGA AATCGCACAG
GACTTCAAGA CCGACCTCCG ATTCCAGAGC TCCGCAGTTA TGGCCCTGCA GGAGGCTAGC
GAGGCTTACC TCGTTGGTCT GTTCGAGGAC ACCAACTTGT GCGCTATCCA CGCCAAGCGT
---------- ---------- ---------- ---------- ---------- --AGGGTNTT
AATTCGTTTG GAGTTATCTC GACCAGGAGC ATTTTTAGGA GATGACCATT TGTATAATGT
CATCGTTACT GCGCATGCTT TTGTTATGAT TTTTTTCTTA GTTATACCAA TGATGGTAGG
GGGGTTTGGG AACTGGTTAG TGCCATNAAT ACTTACTTCC CCTGACATGG CCTTTCCTCG
GATGAACAAT ATGAGGTTTT GATTACTTCC GCCTGCCTTG TTTCTCCTTT TGAGCTCTGC
TTTTGTTGAG AGGGGAGTGG GGACAGGGTG AACTGTTTAT CCTCCTTTAT CAGCTAACGT
AGCTCACAGG GGAGGTTCAG TTGATTACGG GATTTTTTCT CTTCATTTGG CAGGTGTTTC
TTCTATTTTA GGGGCAATCA ATTTTTTAGC AAGGACAGTA AATATGCGCC CTGAAATTAT
AGAATTTANA CGGGTTACCT TGTTTGTGTG GTCGATCGCC ATCACAGCTT TTTTACTGGT
TGTTGCAATA CCTGTGTTGG CGGGGGCTAT TACGATGTTG TTAACAGATC GAAACTTTAN
TACCTCGT-- ---------- ---------- ---------- ---------- ---------

>Peregrinamor_gastrochaenans
TAGTCATATG CTTGTCTCAA AGATTAAGCC ATGCATGTCT AAGTACACGC CAGATACATG

GTGAAACTGC GAATGGCTCA TTAAATCAGT TATGGTTCCT TAGATCGTAC AATCCTACTT
GGATAACTGT GGCAATTCTA GAGCTAATAC ATGCGTCAAA GCTCCGACCT TCGGGGAAGA
GCGCTTTTGT TAGCAAAACC AAACCGGTCG GTTGTTGACT CTGGACAACT T--TGCCGAT
CGCACGGCCT TGCGCCGGCG ACGTATCTTT TGAATGTCTG CCCTATCAAC TGACGATGGT
ACGTGCTATG CCTACCATGG TTGTAACGGG TAACGGGGAA TCAGGGTTCG ATTCCGGAGA
GGGAGCATGA GAAACGGCTA CCACATCTAA GGAAGGCAGC AGGCGCGCAA ATTACCCAAT
CCCGACACGG GGAGGTAGTG ACGAAAAATA ACAATACGGG ACTCTTTCGA GGCCCCGTAA
TTGGAATGAG TACACTTTAA ATCCTTTAAC GAGGATCCAT TGGAGGGCAA GTCTGGTGCC
AGCAGCCGCG GTAATTCCAG CTCCAATAGC GTATATTAAA GTTGCTGTAG TTAAAAAGCT
CGTAGTTGGA TCTCGGGTGC AGGCTTGCGG TCCGCCTCGC GGCGGCTGCT CGTCCTAGCA
GCCTAGCTTC GGTGTCCCTT GGTGCTCTTG ACCGAGTGTC GGTGGCCGGA ACGTTTACTT
TGATGAAATT AGAGTGTTCA GAGCGCGGGC GTTTGCCCGT ATAATGGTGC ATGGAATGAT
AGAATAGGAC CTCGGTTCTA TTTTGTTGGT TTTCGGAGAG GTAATGATCA AGAGGGACAG
ACGGGGGCAT TCGTATTGCA GCGTTAGAGG TGAAATTCTT GGATCGTTGC AAGACGGACA
ACAGCGAAAG CATTTGCCAA GAATGTTTTC CTTAATCAAG AACGAAAGTC AGAGGCTCGA
AGACGATCAG ATACCGTCGT AGTTCTGACC ATAAACCATG CCAACTGGCA ATCCGCCGGA
GTTACTACAA TGACTCGGCG AGCAGCCCCC GGGAAACCAA AGTTTCTGGG TTCCGGGGGG
AGTATGGTTG CAAAGCTGAA ACTTAAAGGA ATTGACGGAA GGGCACCACC AGGAGTGGAG
CCTGTGGCTT AATTTGACTC AACACGGGAA ACCTCACCCG GCCCGGACAC CGTTAGGATT
GACAGATTGA GAGCTCTTTC TTGATTCGGT GGGTGGTGGT GCATGGCCGT TCTTAGTTGG
TGGAGCGATT TGTCTGGTTA ATTCCGATAA CGAACGAGAC TCTAGCCTAC TAAATAGTTC
GAGGATATAT TACCTCGCAA CTTCTTAGAG GGACAGGTGG CGTTTAGCCA CACGAGATTG
AGCAATAACA GGTCTGTGAT GCCCTTAGAT GTTCGGGGCC GCACACGCGC TACATTGAAT
GGATCAACGT GCGTCTAGCC TTGCCCGAAA GGGCTGGGAA ACCCGTTGAA CCCCATTCGT
GATAGGGATT GGGGCTTGCA ATTATTTCCC ATGAACGAGG AATTCCTAGT AAGCGCGAGT
CATCAGCTCG CGTTGATTGC GTCCCTGCCC TTTGTACACA CCGCCCGTCG CTACTACCGA
TCGTTCCAGT TAATGAACGC CTCGGATTGG TCAAGCGGG- -GAAACCTGC TCGCGTGCCG
AGAAGAAGTG TAAGTTATCT GGGATAGAGG TCGTAAAAGT CGTAACAAGG TATCCATTAA
TAAGCGGAGG AAAAGAGACT AACTAGGATT CCCCTAGTAA CGGCGAGTGA AGCGGGAAGA
GCCCAGCACC GAATCCCCCG GCGTCTGGCC GCCGACGGGA CATGTGGTGT TAGCGGGAGT
CTTTTGTCGG CGCGTTCTGG CACCAAAGTC CACCTGATCG TGGCAATCCA GTGCGGGTGA
GAGGCCCGTT CCGGTGCCGG TCACGTCGGA CTCTCTCCAG GAGTCGGGTT GTTTGAGAAT
GCAGCCCAAA GTGGGTGGTA AACTCCATCT AAGGCTAAAT ACAGACACGA GTCCGATAGA
GGACAAGTAC CGTGAGGGAA AGTTGAAAAG AACTTTGAAG AGAGAGTTCA AGAGTACGTG
AAACCGCATA GAGTCAAACG GGTGGATCCG CAAGGTGTTG GACCGGGGAA TTCAGCGGTG
CACTTTCTCC GACGAGAGCC ACGACCGGGT TCGCTGTCCG TGATGCGGGA AGGTTCCTCT
GCGTCCTGAG AGT-TATAGC CCGTCGGCTC CGGACATCTA TGTCGCGGAC CCGAGGACGC
GCCGCGCGTC GGGTTCCGGG TCGTCCTCGT GCGTTCGACT TTCGTCGGTA GTTCTTCGAG
ACCGCGCGCT GGCTCGGCGG TTAGGGTCAG TGGCGAATCA GTCGGTCCTC CACCCGACCC
GTCTTGAAAC ACGGACCAAG GAGTCTAACA TGTGCGCGAG TCGTAGGGAC TCGAATCCCG
AAGGCGCAAT GAAAGTGAAG GCCGCCTACG GTTGGCCGAG GCAGGATCCC GTCGCGGGCG
CACTGCCGGC CCGTCTCGAT AGCGGCGTAG GCGTGCCCAA CGTTCGTCTA CGAGGCGGAG
CAAGAGCGTA CACGTTGGTA CCCGAAAGAT GGTGAACTAT GCCTGAGCAG GACGAAGTCA
GAGGAAACTC TGATGGAGGT CCGCAGCGAT TC???????? ?????????? ??????????
?????????? ?????????? ?????????? ?????????? ?????????? ??????????
?????????? ?????????? ?????????? ?????????? ?????????? ??????????
?????????? ?????????? ?????????? ?????????? ?????????? ??????????
?????????? ?????????? ?????????? ?????????? ?????????? ??????????
?????????? ?????????? ?????????? ?????????? ?????????? ??????????
TACTTTATAT TTTTTATTTG GGATTTGGTC AGGGCTTGTT GGTACCTCTT TTAGGGTGTT

AATTCGTTTG GAGCTTTCTC GTCCTGGAGC TTTTTTGGGT GATGACCATT TATACAATGT
TATCGTTACA GCTCACGCTT TTGTGATAAT TTTTTTTTTG GTTATGCCAA TAATGGTAGG
GGGTTTTGGT AATTGGTTGG TACCCCTTAT GTTAACTTCT CCTGATATGG CTTTTCCTCG
TATAAACAAT ATAAGTTTTT GGTTGCTTCC TCCTGCTTTG TTTTTGCTTT TAAGCTCTGC
TTTTGTTGAG AGAGGAGTTG GAACTGGTTG AACTGTGTAT CCTCCTTTAT CTGGCAATAT
AACTCATAGA GGAGGTTCTG TTGATTATGG AATTTTTTCT CTTCACTTGG CTGGGGTCTC
TTCTATTTTA GGGGCTATTA ATTTTTTGGC TACTACTGTG AATATACGTC CTGAGGTAAT
GGAGTTTAAG CGTGTTACTT TGTTTGTTTG ATCTATCGCC ATTACTGCTT TCTTGCTTGT
GGTTGCTATG CCAGTCTTAG CTGGGGCTAT TACAATATTA CTAACTGATC GCAACTTTAA
CACTTCTTTT TTTGATCCTT CTGGAGGGGG AGATCC---- ---------- ---------

>Peregrinamor_ohshimai
TAGTCATATG CTTGTCTCAA AGATTAAGCC ATGCATGTCT AAGTACACGC CAGATACATG
GTGAAACTGC GAATGGCTCA TTAAATCAGT TATGGTTCCT TAGATCGTAC AATCCTACTT
GGATAACTGT GGCAATTCTA GAGCTAATAC ATGCGTCAAA GCTCCGACCT TCGGGGAAGA
GCGCTTTTGT TAGCAAAACC AATCCGGTCG GTTGTTGACT CTGGACAACT T--TGCCGAT
CGCACGGCCT TGCGCCGGCG ACGTATCTTT TGAATGTCTG CCCTATCAAC TGACGATGGT
ACGTGCTATG CCTACCATGG TTGTAACGGG TAACGGGGAA TCAGGGTTCG ATTCCGGAGA
GGGAGCATGA GAAACGGCTA CCACATCTAA GGAAGGCAGC AGGCGCGCAA ATTACCCAAT
CCCGACACGG GGAGGTAGTG ACGAAAAATA ACAATACGGG ACTCTTTCGA GGCCCCGTAA
TTGGAATGAG TACACTTTAA ATCCTTTAAC GAGGATCCAT TGGAGGGCAA GTCTGGTGCC
AGCAGCCGCG GTAATTCCAG CTCCAATAGC GTATATTAAA GTTGCTGTAG TTAAAAAGCT
CGTAGTTGGA TCTCGGGTGC AGGCTTGCGG TCCGCCTCGC GGCGGCTGCT CGTCCTAGCA
GCCTAGCTTC GGTGTCCCTT GGTGCTCTTG ACCGAGTGTC GGTGGCCGGA ACGTTTACTT
TGATGAAATT AGAGTGTTCA GAGCGCGGGC GTTTGCCCGT ATAATGGTGC ATGGAATGAT
AGAATAGGAC CTCGGTTCTA TTTTGTTGGT TTTCGGAGAG GTAATGATCA AGAGGGACAG
ACGGGGGCAT TCGTATTGCA GCGTTAGAGG TGAAATTCTT GGATCGTTGC AAGACGGACA
ACAGCGAAAG CATTTGCCAA GAATGTTTTC CTTAATCAAG AACGAAAGTC AGAGGCTTGA
AGACGATCAG ATACCGTCGT AGTTCTGACC ATAAACCATG CCAACTGGCA ATCCGCCGGA
GTTACTACAA TGACTCGGCG AGCAGCCCCC GGGAAACCAA AGTTTCTGGG TTCCGGGGGG
AGTATGGTTG CAAAGCTGAA ACTTAAAGGA ATTGACGGAA GGGCACCACC AGGAGTGGAG
CCTGTGGCTT AATTTGACTC AACACGGGAA ACCTCACCCG GCCCGGACAC CGTTAGGATT
GACAGATTGA GAGCTCTTTC TTGATTCGGT GGGTGGTGGT GCATGGCCGT TCTTAGTTGG
TGGAGCGATT TGTCTGGTTA ATTCCGATAA CGAACGAGAC TCTAGCCTAC TAAATAGTTC
GAGGATATAT TACCTCGCAA CTTCTTAGAG GGACAGGTGG CGTTTAGCCA CACGAGATTG
AGCAATAACA GGTCTGTGAT GCCCTTAGAT GTTCGGGGCC GCACACGCGC TACATTGAAT
GGATCAACGT GCGTCTAGCC TTGCCCGAAA GGGCTGGGAA ACCCGTTGAA CCCCATTCGT
GATAGGGATT GGGGCTTGCA ATTATTTCCC ATGAACGAGG AATTCCTAGT AAGCGCGAGT
CATCAGCTCG CGTTGATTGC GTCCCTGCCC TTTGTACACA CCGCCCGTCG CTACTACCGA
TCGTTCCAGT TAATGAACGC CTCGGATTGG TCAAGCGGG- -GAGACCTGC TCGCGTGCCG
AGAAGAAGTG TAAGTTATCT GGGATAGAGG TCGTAAAAGT CGTAACAAGG TATCCATTAA
TAAGCGGAGG AAAAGAGACT AACTAGGATT CCCCTAGTAA CGGCGAGTGA AGCGGGAAGA
GCCCAGCACC GAATCCCCCG GCGTCTGGCC GCCGACGGGA CATGTGGTGT TAGCGGGAGT
CTATTGTCGG CGCGTTCTGG CACCAAAGTC CACCTGATCG TGGCAATCCA GTGCGGGTGA
GAGGCCCGTT CCGGTGCCGG TCACGTCGGA CTCTCTCCAG GAGTCGGGTT GTTTGAGAAT
GCAGCCCAAA GTGGGTGGTA AACTCCATCT AAGGCTAAAT ACAGACACGA GTCCGATAGA
GGACAAGTAC CGTGAGGGAA AGTTGAAAAG AACTTTGAAG AGAGAGTTCA AGAGTACGTG
AAACCGCATA GAGTCAAACG GGTGGATCCG CAAGGTGTTG GACCGGGGAA TTCAGCGGTG
CACTTTCTCC GACGAGAGCC ACGACCGGGT TTGCTGTCCG CGGTGCGGGA AGGTGCCTCT
GCGTCCT-GA GAGTTATAGC CCGTCGGCTC CGGACATCTA TGTCGCGGAC CCGAGGACGC

GCCGCGCGTC GGGTTCCGGG TCGTCCTCGT GCGTTCGACT TTCGTCGGTA GTTCTTCGAG
ACCGCGCGCT GGCTCGGCGG TCAGGGTCAG TGGCGAATCA GTCGGTCCTC CACCCGACCC
GTCTTGAAAC ACGGACCAAG GAGTCTAACA TGTGCGCGAG TCGTAGGGAC TCGAATCCCG
AAGGCGCAAT GAAAGTGAAG GCCGCCTACG GTTGGCCGAG GCAGGATCCC GTCGCGGGCG
CACTGCCGGC CCGTCTCGAT AGCGGCGTAG GCGTGCCCAA CGTTCGTCTA CGAGGCGGAG
CAAGAGCGTA CACGTTGGTA CCCGAAAGAT GGTGAACTAT GCCTGAGCAG GACGAAGTCA
GAGGAAACTC TGATGGAGGT CCGCAGCGAT TCCCGAAAAT CCACTGGTGG GAAAGCCCCC
AGGAAACAAT TGGCCACAAA GGCCGCACGT AAAAGTGCAC CGGCCACAGG TGGTGTGAAG
AAACCACACA GATACAGGCC CGGAACCGTC GCCCTCCGTG AAATCAGGAG ATACCAGAAG
AGCACCGAAC TTTTGATCAG AAAGTTGCCC TTCCAGCGAC TCGTCCGTGA GATCGCCCAG
GACTTCAAGA CTGACCTCCG GTTCCAGAGC TCTGCCGTCA TGGCCCTTCA GGAGGCTAGT
GAGGCCTACT TGGTTGGACT GTTTGAGGAC ACCAACTTGT GCGCCATCCA CGCCAAGCGT
---------- ---------- ------GATC TGGTTTGGTA GGTACTTCTT TTAGNGTGCT
AATTCGATTG GANCTNTTAN GNCCNGGTGC TTTTTTAGGN GATGACCATN TTTATAATGN
NATTGTTACG GCTCATGCTT TTGTGATAAT TTTTTTTTTA GTTATGCCGA TAATGGTAGG
AGGATTTGGT AATTGATTGG TTCCTTTAAT ACTCACTTCC CCGGACATAG CTTTTCCTCG
AATGAATAAT ATGAGTTTTT GGTTGCTTCC TCCTGCCTTG TTTCTTTTAT TGAGATCAGC
TTTTGTGGAN AGAGGAGTTG GTACGGGTTG AACNGTTTAT CCTCCTTTAT CTGGTAATGT
AACCCATAGA GGAGGTTCTG TNGATTATGG GATTTTNTCT CTTCATTTAG CAGGTGTTTC
TTCTATTTTG GGGGCTATTA ATTTTTTGGC AACAACCGTT AACATGCGTC CAGAGGTTAT
AGAATTNAAG CGAGTAACTT TATTTGTTTG ATCTATTGCT ATTACGGCTT TTCTCTTGGT
GGTTGCAATG CCNGTTTTAG CGGGAGCTAT TACAATACTG TTAACTGATC GTAATTTTAA
CACTTCTTTT TTCGATCCTT CTGGAGGTGG GGANCCAATT NNGTTTGTTC ATTTATTTT

>Pseudopythina_ochetostomae
TAGTCATATG CTTGTCTCAA AGATTAAGCC ATGCATGTCT AAGTACACGC CAGATACATG
GTGAAACTGC GAATGGCTCA TTAAATCAGT TATGGTTCCT TAGATCGTAC AATCCTACTT
GGATAACTGT GGCAATTCTA GAGCTAATAC ATGCGTCAAA GCTCCGACCT CACGGGAAGA
GCGCTTTTGT TAGCAAAACC AATCTGGTCA GTTGTTGACT CTGGACAACT T--TGCCGAT
CGCACGGCCT TGCGCCGGCG ACGTATCTTT TGAATGTCTG CCCTATCAAC TGACGATGGT
ACGTGCTATG CCTACCATGG TTGTAACGGG TAACGGGGAA TCAGGGTTCG ATTCCGGAGA
GGGAGCATGA GAAACGGCTA CCACATCTAA GGAAGGCAGC AGGCGCGCAA ATTACCCAAT
CCCGACACGG GGAGGTAGTG ACGAAAAATA ACAATACGGG ACTCTTTCGA GGCCCCGTAA
TTGGAATGAG TACACTTTAA ATCCTTTAAC GAGGATCCAT TGGAGGGCAA GTCTGGTGCC
AGCAGCCGCG GTAATTCCAG CTCCAATAGC GTATATTAAA GTTGCTGTAG TTAAAAAGCT
CGTAGTTGGA TCTCGGGTGC AGGCTTGCGG TCCGCCTCGC GGCGGCTGCT CGTCCTAGCA
GCCTAGCTTC GGTGTCCCTT GGTGCTCTTG ACCGAGTGTC GGTGGCCGGA ACGTTTACTT
TGATGAAATT AGAGTGTTCA GAGCGCGGGC GTTTGCCCGT ATAATGGTGC ATGGAATGAT
AGAATAGGAC CTCGGTTCTA TTTTGTTGGT TTTCGGAGAG GTAATGATCA AGAGGGACAG
ACGGGGGCAT TCGTATTGCA GCGTTAGAGG TGAAATTCTT GGATCGTTGC AAGACGGACG
ACAGCGAAAG CATTTGCCAA GAATGTTTTC CTTAATCAAG AACGAAAGTC AGAGGCTCGA
AGACGATCAG ATACCGTCGT AGTTCTGACC ATAAACCATG CCAACTGGCA ATCCGCCGGA
GTTACTACAA TGACTCGGCG AGCAGCCCCC GGGAAACCAA AGTTTCTGGG TTCCGGGGGG
AGTATGGTTG CAAAGCTGAA ACTTAAAGGA ATTGACGGAA GGGCACCACC AGGAGTGGAG
CCTGTGGCTT AATTTGACTC AACACGGGAA ACCTCACCCG GCCCGGACAC CGTTAGGATT
GACAGATTGA GAGCTCTTTC TTGATTCGGT GGGTGGTGGT GCATGGCCGT TCTTAGTTGG
TGGAGCGATT TGTCTGGTTA ATTCCGATAA CGAACGAGAC TCTAGCCTAC TAAATAGTTC
GAGGATATAT TACCTCGCAA CTTCTTAGAG GGACAGGTGG CGTATAGCCA CACGAGATTG
AGCAATAACA GGTCTGTGAT GCCCTTAGAT GTTCGGGGCC GCACACGCGC TACATTGAAT
GGATCAACGT GCGTCTAGCC TTGCCCGAAA GGGCTGGGAA ACCCGTTGAA CCCCATTCGT

GATAGGGATT GGGGCTTGCA ATTATTTCCC ATGAACGAGG AATTCCTAGT AAGCGCGAGT
CATCAGCTCG CGTTGATTGC GTCCCTGCCC TTTGTACACA CCGCCCGTCG CTACTACCGA
TCGTTCCAGT TAATGAACGC CTCGGATTGG TTAAGCGGG- -GAAACCTGC TCGCGTGCCG
AGAAGAAGTG TAAGTTATCT GGGATAGAGG TCGTAAAAGT CGTAACAAGG TATCCATTAA
TAAGCGGAGG AAAAGAGACT AACTAGGATT CCCCTAGTAA CGGCGAGTGA AGCGGGAAGA
GCCCAGCACC GAATCCCCCG GCGTCTGGCC GCCGACGGGA CATGTGGTGT TAGCGGGAGT
CTGTTGTCGG CGCGTTCTGG CACCAAAGTC CACCGGGTCG TGGCAATCCA GTGCGGGTGA
GAGGCCCGTT CCGGTGCCGG TCACGTCGGA CTCTCTCCAG GAGTCGGGTT GTTTGAGAAT
GCAGCCCAAA GTGGGTGGTA AACTCCATCT AAGGCTAAAT ACAGACACGA GTCCGATAGA
GGACAAGTAC CGTGAGGGAA AGTTGAAAAG AACTTTGAAG AGAGAGTTCA AGAGTACGTG
AAACCGCATA GAGTCAAACG GGTGGATCCG CAAGGTGTTG GACCGGGGAA TTCAGCGGTG
CACTTTCTCC GACGAGAGCC ACGACCGGGT TCGCTGTTCG CGGTGCGGGA AGGTTTCCCG
GCGCCT-GGG AGT-TACAGC CCGTCGGCCC TGGACATCTA TGTCGCGGAC CCGAGGACGC
GCCGCGCGTC GGGTCCCGGG TCGTCCTCGT GCGTTCGACT TTCGTCGGTA GTTCTTCGAG
ACCGCGCGCT GGCTCGGCGG CCAGGGTCAG TGGCGAATCA GTCGGTACTC CACCCGACCC
GTCTTGAAAC ACGGACCAAG GAGTCTAACA TGTGCGCAAG TCGTAGGGAC TCGAATCCCG
AAGGCGCAAT GAAAGTGAAG GCCGCCTACG GTTGGCCGAG GCAGGATCCC GTCGCGGGCG
CACTGCCGGC CCGTCTCGAT AGCGGCGTAG GCGTGCCCAA CGTTCGTCTA CGAGGCGGAG
CAAGAGCGTA CACGTTGGTA CCCGAAAGAT GGTGAACTAT GCCTGAGCAG GACGAAGTCA
GAGGAAACTC TGATGGAGGT CCGCAGCGAT TCTAGAAAGT CCACTGGAGG GAAAGCCCCC
AGGAAACAGC TGGCCACCAA GGCCGCACGT AAGAGTGCAC CGGCTACCGG TGGTGTGAAG
AAGCCACACA GATACAGGCC CGGAACCGTT GCCCTCCGTG AAATCAGACG ATACCAGAAG
AGCACTGAAC TGCTCATCAG AAAATTGCCC TTCCAGCGTC TCGTCCGTGA GATCGCCCAG
GACTTCAAGA CTGACCTGCG ATTCCAGAGC TCTGCCGTCA TGGCCCTTCA GGAGGCCAGT
GAGGCTTACT TGGTCGGACT TTTCGAGGAC ACCAACTTGT GCGCTATCCA CGCCAAGCGT
?????????? ?????????? ?????????? ?????????? ?????????? ??????????
?????????? ?????????? ?????????? ?????????? ?????????? ??????????
?????????? ?????????? ?????????? ?????????? ?????????? ??????????
?????????? ?????????? ?????????? ?????????? ?????????? ??????????
?????????? ?????????? ?????????? ?????????? ?????????? ??????????
?????????? ?????????? ?????????? ?????????? ?????????? ??????????
?????????? ?????????? ?????????? ?????????? ?????????? ??????????
?????????? ?????????? ?????????? ?????????? ?????????? ??????????
?????????? ?????????? ?????????? ?????????? ?????????? ??????????
?????????? ?????????? ?????????? ?????????? ?????????? ??????????
?????????? ?????????? ?????????? ?????????? ?????????? ?????????

>Pseudopythina_subsinuata
TAGTCATATG CTTGTCTCAA AGATTAAGCC ATGCATGTCT AAGTACACGC CAGATACATG
GTGAAACTGC GAATGGCTCA TTAAATCAGT TATGGTTCCT TAGATCGTAC AATCCTACTT
GGATAACTGT GGCAATTCTA GAGCTAATAC ATGCGTCAAA GCTCCGACCT TAGGGGAAGA
GCGCTTTTGT TAGCAAAACC AATCTGGTCA GTTGTTGACT CTGGACAACT T--TGCCGAT
CGCACGGCCT TGCGCCGGCG ACGTATCTTT TGAATGTCTG CCCTATCAAC TGACGATGGT
ACGTGCTATG CCTACCATGG TTGTAACGGG TAACGGGGAA TCAGGGTTCG ATTCCGGAGA
GGGAGCATGA GAAACGGCTA CCACATCTAA GGAAGGCAGC AGGCGCGCAA ATTACCCAAT
CCCGACACGG GGAGGTAGTG ACGAAAAATA ACAATACGGG ACTCTTTCGA GGCCCCGTAA
TTGGAATGAG TACACTTTAA ATCCTTTAAC GAGGATCCAT TGGAGGGCAA GTCTGGTGCC
AGCAGCCGCG GTAATTCCAG CTCCAATAGC GTATATTAAA GTTGCTGTAG TTAAAAAGCT
CGTAGTTGGA TCTCGGGTGC AGGCTTGCGG TCCGCCTCGC GGCGGCTGCT CGTCCTAGCA
GCCTAGCTTC GGTGTCCCTT GGTGCTCTTG ACCGAGTGTC GGTGGCCGGA ACGTTTACTT

TGATGAAATT AGAGTGTTCA GAGCGCGGGC GTTTGCCCGT ATAATGGTGC ATGGAATGAT
AGAATAGGAC CTCGGTTCTA TTTTGTTGGT TTTCGGAGAG GTAATGATCA AGAGGGACAG
ACGGGGGCAT TCGTATTGCA GCGTTAGAGG TGAAATTCTT GGATCGTTGC AAGACGGACG
ACAGCGAAAG CATTTGCCAA GAATGTTTTC CTTAATCAAG AACGAAAGTC AGAGGCTCGA
AGACGATCAG ATACCGTCGT AGTTCTGACC ATAAACCATG CCAACTGGCA ATCCGCCGGA
GTTACTACAA TGACTCGGCG AGGCGCCCCC GGGAAACCAA AGTTTCTGGG TTCCGGGGGG
AGTATGGTTG CAAAGCTGAA ACTTAAAGGA ATTGACGGAA GGGCACCACC AGGAGTGGAG
CCTGTGGCTT AATTTGACTC AACACGGGAA ACCTCACCCG GCCCGGACAC CGTTAGGATT
GACAGATTGA GAGCTCTTTC TTGATTCGGT GGGTGGTGGT GCATGGCCGT TCTTAGTTGG
TGGAGCGATT TGTCTGGTTA ATTCCGATAA CGAACGAGAC TCTAGCCTAC TAAATAGTTC
GAGGATATAT TACCTCGCAA CTTCTTAGAG GGACAGGTGG CGTATAGCCA CACGAGATTG
AGCAATAACA GGTCTGTGAT GCCCTTAGAT GTTCGGGGCC GCACACGCGC TACATTGAAT
GGATCAACGT GCGTCTAGCC TTGCCCGAAA GGGCTGGGAA ACCCGTTGAA CCCCATTCGT
GATAGGGATT GGGGCTTGCA ATTATTTCCC ATGAACGAGG AATTCCTAGT AAGCGCGAGT
CATCAGCTCG CGTTGATTGC GTCCCTGCCC TTTGTACACA CCGCCCGTCG CTACTACCGA
TCGTTCCAGT TAATGAACGC CTCGGATTGG TTAAGCGGG- -GAAACCTGC TCGCGCGCCG
AGAAGAAGTG TAAGTTATCT GGGATAGAGG TCGTAAAAGT CGTAACAAGG TATCCATTAA
TAAGCGGAGG AAAAGAGACT AACTAGGATT CCCCTAGTAA CGGCGAGTGA AGCGGGAAGA
GCCCAGCACC GAATCCCCCG GCGTCTGGCC GCCGACGGGA CATGTGGTGT TAGCGGGAGT
CTTTTGTCGG CGCGTTCTGG CACCAAAGTC CACCGGGTCG TGGCAATCCA TTGCGGGTGA
GAGGCCCGTT CCGGTGCCGG TTCCGTCGGA CTCTCTCCAG GAGTCGGGTT GTTTGAGAAT
GCAGCCCAAA GTGGGTGGTA AACTCCATCT AAGGCTAAAT ACAGACACGA GTCCGATAGA
GGACAAGTAC CGTGAGGGAA AGTTGAAAAG AACTTTGAAG AGAGAGTTCA AGAGTACGTG
AAACCGCATA GAGTCAAACG GGTGGATCCG CAAGGTGTTG GACCGGGGAA TTCAGCGGTG
CACTTTCTCC GACGAGAGCC ATGACCGGGT TCGCTGTCCG TGGTGCGGGA AGGTTTCCCG
GCGTCT-GGG AGT-TACAGC CCGTCGGCCC CGGACATCTA CGTCGCGGAC CCGAGGACGC
GCCGCGCGTC GGGTTCCGGG TCGTCCTGGT GCGTTCAACT TTCGTCGGCA GTTCTTCGAG
ACCGCGCGCC GGCTCGGCAA CTATGGTCAG TGGCGAATCA GTCGGTCCTC CACCCGACCC
GTCTTGAAAC ACGGACCAAG GAGTCTAACA TGTGCGCGAG TCGTAGGGAC TCGAATCCCG
AAGGCGCAAT GAAAGTGAAG GCCGCCTACG GTTGGCCGAG GCAGGATCCC GTCGCGGGCG
CACTGCCGGC CCGTCTCGAT AGCGGCGTAG GCGTGCCCAA CGTTCGTCTA CGAGGCGGAG
CAAGAGCGTA CACGTTGGTA CCCGAAAGAT GGTGAACTAT GCCTGAGCAG GACGAAGTCA
GAGGAAACTC TGATGGAGGT CCGCAGCGAT TCTAGAAAGT CCACTGGTGG GAAAGCCCCC
AGGAAACAAT TGGCCACCAA GGCCGCACGT AAGAGTGCAC CAGCTACCGG CGGTGTAAAG
AAGCCACACA GATACAGGCC CGGAACTGTC GCCCTCCGTG AAATCAGACG ATACCAGAAG
AGCACTGAAT TGCTCATCAG AAAGTTGCCA TTCCAGCGTC TCGTCCGCGA GATCGCTCAG
GACTTCAAGA CTGACTTGAG ATTCCAGAGC TCTGCCGTCA TGGCTCTCCA GGAAGCCAGT
GAGGCTTACT TGGTCGGGCT TTTCGAGGAC ACCAACTTGT GCGCTATCCA CGCCAAGCGT
CACCTTGTAT TTTTTATTTG GTATTTGATC TGGGTTGGTA GGTACTTCTT TTAGGGTATT
AATTCGTTTA GAGCTATCTC GCCCAGGCGC TTTTTTAGGG GATGATCATT TATATAACGT
AATTGTTACA GCTCACGCTT TTGTTATAAT TTTTTTTTTG GTAATACCTA TGATGGTAGG
AGGATTTGGG AATTGGTTAG TTCCTTTAAT ATTAACTTCT CCAGACATAG CCTTCCCTCG
AATAAATAAT ATAAGGTTTT GACTATTACC TCCTGCTTTG TTTTTATTAT TAAGATCTGC
TTTCGTGGAA AGAGGAGTGG GGACCGGGTG AACTGTTTAT CCGCCTTTAT CTGGGAATGT
AACTCATAGG GGGGGTTCAG TTGATTATGG AATTTTTTCT CTTCATCTTG CTGGTGTTTC
TTCTATTTTA GGAGCTATTA ATTTTTTGGC TACTATGGTT AATATACGTC CAGAGATTAT
AGAATTGAAG CGAGTAACTT TGTTTGTTTG GTCTATTGGA ATTACGGCTT TTTTATTAGT
AGTGGCTATA CCTGTTTTGG CTGGAGCTAT TACAATATTA TTAACGGATC GCAATTTTAA
TACTTCATTT TTTGATCCTT CTGGTGGGGG GGATCCTA-- ---------- ---------

>Pseudopythina_macrophthalmensis
TAGTCATATG CTTGTCTCAA AGATTAAGCC ATGCATGTCT AAGTACACGC CAGATAAATG
GTGAAACTGC GAATGGCTCA TTAAATCAGT TATGGTTCCT TAGATCGTAC AATCCTACTT
GGATAACTGT GGCAATTCTA GAGCTAATAC ATGCGTCAAA GCTCCGACCT CACGGGAAGA
GCGCTTTTGT TAGCAAAACC AATCTGGTCA GTTGTTGACT CTGGACAACT T--TGCCGAT
CGCACGGCCT TGCGCCGGCG ACGTATCTTT TGAATGTCTG CCCTATCAAC TGACGATGGT
ACGTGCTATG CCTACCATGG TTGTAACGGG TAACGGGGAA TCAGGGTTCG ATTCCGGAGA
GGGAGCATGA GAAACGGCTA CCACATCTAA GGAAGGCAGC AGGCGCGCAA ATTACCCAAT
CCCGACACGG GGAGGTAGTG ACGAAAAATA ACAATACGGG ACTCTTTCGA GGCCCCGTAA
TTGGAATGAG TACACTTTAA ATCCTTTAAC GAGGATCCAT TGGAGGGCAA GTCTGGTGCC
AGCAGCCGCG GTAATTCCAG CTCCAATAGC GTATATTAAA GTTGCTGTAG TTAAAAAGCT
CGTAGTTGGA TCTCGGGTGC AGGCTTGCGG TCCGCCTCGC GGCGGCTGCT CGTCCTAGCA
GCCTAGCTTC GGTGTCCCTT GGTGCTCTTG ACCGAGTGTC GGTGGCCGGA ACGTTTACTT
TGATGAAATT AGAGTGTTCA GAGCGCGGGC GTTTGCCCGT ATAATGGTGC ATGGAATGAT
GGAATAGGAC CTCGGTTCTA TTTTGTTGGT TTTCGGAGAG GTAATGATCA AGAGGGACAG
ACGGGGGCAT TCGTATTGCA GCGTTAGAGG TGAAATTCTT GGATCGTTGC AAGACGGACA
ACAGCGAAAG CATTTGCCAA GAATGTTTTC CTTAATCAAG AACGAAAGTC AGAGGCTCGA
AGACGATCAG ATACCGTCGT AGTTCTGACC ATAAACCATG CCGACTGGCA ATCCGCCGGA
GTTACTACAA TGACTCGGCG AGCAGCCCCC GGGAAACCAA AGTTTCTGGG TTCCGGGGGG
AGTATGGTTG CAAAGCTGAA ACTTAAAGGA ATTGACGGAA GGGCACCACC AGGAGTGGAG
CCTGTGGCTT AATTTGACTC AACACGGGAA ACCTCACCCG GCCCGGACAC CGTTAGGATT
GACAGATTGA GAGCTCTTTC TTGATTCGGT GGGTGGTGGT GCATGGCCGT TCTTAGTTGG
TGGAGCGATT TGTCTGGTTA ATTCCGATAA CGAACGAGAC TCTAGCCTAC TAAATAGTTC
GAGGATATAT TACCTCGCAA CTTCTTAGAG GGACAGGTGG CGTATAGCCA CACGAGATTG
AGCAATAACA GGTCTGTGAT GCCCTTAGAT GTTCGGGGCC GCACACGCGC TACATTGAAT
GGATCAACGT GCGTCTAGCC TTGCCCGAAA GGGCTGGGAA ACCCGTTGAA CCCCATTCGT
GATAGGGATT GGGGCTTGCA ATTATTTCCC ATGAACGAGG AATTCCTAGT AAGCGCGAGT
CATCAGCTCG CGTTGATTGC GTCCCTGCCC TTTGTACACA CCGCCCGTCG CTACTACCGA
TCGTTCCAGT TAATGAACGC CTCGGATTGG TTAAGCGGG- -GAAACCTGC TCGCGTGCCG
AGAAGAAGTG TAAGTTATCT GGGATAGAGG TCGTAAAAGT CGTAACAAGG TATCCATTAA
TAAGCGGAGG AAAAGAGACT AACTAGGATT CCCCTAGTAA CGGCGAGTGA AGCGGGAAGA
GCCCAGCACC GAATCCCCCG GCGTCTGGCT GCCGACGGGA CATGTGGTGT TAGCGGGAGT
CTTTTGTCGG CGCGTTCTGG CACCAAAGTC CACCGGGTCG TGGCAATCCA TTGCGGGTGA
GAGGCCCGTT CCGGTGCCGG TTCCGTCGGA CTCTCTCCAG GAGTCGGGTT GTTTGAGAAT
GCAGCCCAAA GTGGGTGGTA AACTCCATCT AAGGCTAAAT ACAGACACGA GTCCGATAGA
GGACAAGTAC CGTGAGGGAA AGTTGAAAAG AACTTTGAAG AGAGAGTTCA AGAGTACGTG
AAACCGCATA GAGTCAAACG GGTGGATCCG CAAGGTGTTG GACCGGGGAA TTCAGCGGTG
CACTTTCTCC GACGAGAGCC ATGACCGGGT TCGCTGTTCG TGGTGCGGGA AGGTTTCCCG
GCGTCT-GGG AGT-TACAGC CCGTCGGCCC CGGACATCTA TGTCGTGGAC CCGAGGACGC
GCCGCGCGTT GGGCTCCGGG TCGTCCTAGT GCGTTCGACT TTCGTCGGTA GTTCTTCGAG
ACCGCGTGCT GGCTCGGCGG CTAGGGTCAG TGGCGAATCG GTCGGTCCTC CACCCGACCC
GTCTTGAAAC ACGGACCAAG GAGTCTAACA TGTGCGCGAG TCGTAGGGAC TCGAATCCCG
AAGGCGCAAT GAAAGTGAAG GCCGCCTACG GTTGGCCGAG GCAGGATCCC GTCGTGGGCG
CACTGCCGGC CCGTCTCGAT AGCGGCGTAG GCGTGCCCAA CGTTCGTCTA CGAGGCGGAG
CAAGAGCGTA CACGTTGGTA CCCGAAAGAT GGTGAACTAT GCCTGAGCAG GACGAAGTCA
GAGGAAACTC TGATGGAGGT CCGCAGCGAT TCCCGAAAGA GTACCGGTGG CAAAGCCCCC
AGGAAACAGC TGGCCACCAA GGCCGCAAGA AAGAGTGCAC CAGCTACCGG CGGTGTGAAG
AAACCACACA GATACAGGCC CGGAACTGTT GCTCTCCGTG AAATCAGACG ATACCAGAAG
AGCACTGAAC TGCTCATCAG AAAACTGCCC TTCCAGCGTC TCGTCCGTGA AATCGCTCAG
GACTTCAAGA CTGACCTTCG CTTCCAGAGC TCTGCCGTTA TGGCTCTCCA GGAGGCCAGT

GAGGCTTACT TGGTTGGACT TTTTGAGGAC ACAAACTTGT GCGCTATCCA CGCCAAGCGT
AACTCTTTAT TTTTTATTTG GAATCTGGTC AGGTATGGTA GGGACTTCTT TTAGAGTTTT
AATTCGATTG GAGTTATCTC GGCCAGGGGC TTTTTTAGGC GACGATCATC TTTATAATGT
GATCGTAACA GCCCACGCTT TTGTAATGAT TTTCTTCTTG GTTATACCTA TGATAGTAGG
GGGATTTGGA AATTGGCTAG TTCCTTTAAT ATTAGCGGCG CCAGACATAG CATTCCCTCG
GATAAATAAT ATAAGATTTT GGCTTCTTCC TCCTGCGCTG TTTTTACTTT TGAGATCAGC
GTTTGTAGAA AGAGGAGCTG GAACCGGGTG GACCGTTTAC CCTCCTTTAT CTGGGAACGT
AACCCATAGA GGGGGTTCTG TGGATTATGC AATTTTTTCT CTACATTTGG CGGGTGTGTC
ATCAATTTTA GGTGCTATTA ATTTTTTGGC TACAATAATA AATATGCGTC CTGAAATTAT
GGAGCTTAAA CGTGTCACTC TATTCATTTG ATCAATTGGT TTGACTGCTT TTCTGCTTGT
TGCAGCTATG CCCGTGTTAG CTGGGGCTAT TACAATGCTT CTTACGGATC GGAATTTTAA
CACATCTTTT TTTGACCCTT CTGGGGGTGG GGATCCAATT CTATTCGTCC ATTTGTTTT

>Pseudopythina_aff_ariake
TAGTCATATG CTTGTCTCAA AGATTAAGCC ATGCATGTCT AAGTACACGC CAGATACATG
GTGAAACTGC GAATGGCTCA TTAAATCAGT TATGGTTCCT TAGATCGTAC AATCCTACTT
GGATAACTGT GGCAATTCTA GAGCTAATAC ATGCGTCAAA GCTCCGACCT CACGGGAAGA
GCGCTTTTGT TAGCAAAACC AAACCGGTCG GTTGTTGACT CTGGACAACT T--TGCCGAT
CGCACGGCCT TGCGCCGGCG ACGTATCTTT TGAATGTCTG CCCTATCAAC TGACGATGGT
ACGTGCTATG CCTACCATGG TTGTAACGGG TAACGGGGAA TCAGGGTTCG ATTCCGGAGA
GGGAGCATGA GAAACGGCTA CCACATCTAA GGAAGGCAGC AGGCGCGCAA ATTACCCAAT
CCCGACACGG GGAGGTAGTG ACGAAAAATA ACAATACGGG ACTCTTTCGA GGCCCCGTAA
TTGGAATGAG TACACTTTAA ATCCTTTAAC GAGGATCCAT TGGAGGGCAA GTCTGGTGCC
AGCAGCCGCG GTAATTCCAG CTCCAATAGC GTATATTAAA GTTGCTGTAG TTAAAAAGCT
CGTAGTTGGA TCTCGGGTGC AGGCTTGCGG TCCGCCTCGC GGCGGCTGCT CGTCCTAGCA
GCCTAGCTTC GGTGTCCCTT GGTGCTCTTG ACCGAGTGTC GGTGGCCGGA ACGTTTACTT
TGATGAAATT AGAGTGTTCA GAGCGCGGGC GTTTGCCCGT ATAATGGTGC ATGGAATGAT
AGAATAGGAC CTCGGTTCTA TTTTGTTGGT TTTCGGAGAG GTAATGATCA AGAGGGACAG
ACGGGGGCAT TCGTATTGCA GCGTTAGAGG TGAAATTCTT GGATCGTTGC AAGACGGACG
ACAGCGAAAG CATTTGCCAA GAATGTTTTC CTTAATCAAG AACGAAAGTC AGAGGCTCGA
AGACGATCAG ATACCGTCGT AGTTCTGACC ATAAACCATG CCAACTGGCA ATCCGCCGGA
GTTACTACAA TGACTCGGCG AGCAGCCCCC GGGAAACCAA AGTTTCTGGG TTCCGGGGGG
AGTATGGTTG CAAAGCTGAA ACTTAAAGGA ATTGACGGAA GGGCACCACC AGGAGTGGAG
CCTGTGGCTT AATTTGACTC AACACGGGAA ACCTCACCCG GCCCGGACAC CGTTAGGATT
GACAGATTGA GAGCTCTTTC TTGATTCGGT GGGTGGTGGT GCATGGCCGT TCTTAGTTGG
TGGAGCGATT TGTCTGGTTA ATTCCGATAA CGAACGAGAC TCTAGCCTAC TAAATAGTTC
GAGGATATAT TACCTCGCAA CTTCTTAGAG GGACAGGTGG CGTATAGCCA CACGAGATTG
AGCAATAACA GGTCTGTGAT GCCCTTAGAT GTTCGGGGCC GCACACGCGC TACATTGAAT
GGATCAACGT GCGTCTAGCC TTGCCCGAAA GGGCTGGGAA ACCCGTTGAA CCCCATTCGT
GATAGGGATT GGGGCTTGCA ATTATTTCCC ATGAACGAGG AATTCCTAGT AAGCGCGAGT
CATCAGCTCG CGTTGATTGC GTCCCTGCCC TTTGTACACA CCGCCCGTCG CTACTACCGA
TCGTTCCAGT TAATGAACGC CTCGGATTGG TTAAGCGGG- -GAAACCTGC TCGCGTGCCG
AGAAGAAGTG TAAGTTATCT GGGATAGAGG TCGTAAAAGT CGTAACAAGG TATCCATTAA
TAAGCGGAGG AAAAGAGACT AACTAGGATT CCCCTAGTAA CGGCGAGTGA AGCGGGAAGA
GCCCAGCACC GAATCCCCCG GCATCTGGCT GCCGACGGGA CATGTGGTGT TAGCGGGAGT
CTTTTGTCGG CGCGTACTGG CACCAAAGTC CACCTGATCG TGGCAATCCA GTGCGGGTGA
GAGGCCCGTT CCGGTGCCGG TCACGTCGGA CTCTCTCCAG GAGTCGGGTT GTTTGAGAAT
GCAGCCCAAA GTGGGTGGTA GACTCCATCT AAGGCTAAAT ACAGACACGA GTCCGATAGA
GGACAAGTAC CGTGAGGGAA AGTTGAAAAG AACTTTGAAG AGAGAGTTCA AGAGTACGTG
AAACCGCATA GAGTCAAACG GGTGGATCCG CAAGGTGTTG GACCGGGGAA TTCAGCGGTG

CACTTTCTCC GACGAGAGCC ACGACCGGGT TCGCTGTTCG CGGTGCGGGA AGGTTTCCCG
GCGTCT-GGG AGT-TACAGC CCGTCGGCCC TGGACATCTA TGTCGCGGAC CCGAGGACGC
GCCGCGCGTC GGGTCTCGGG TCGTCCTCGT GCGTTCGACT TTCGTCGGTA GTTCTTCGAG
ACCGCGCGCT GGCTCGGTGG CCAGGGTCAG TGGCGAATCA GTCGGTACTC CACCCGACCC
GTCTTGAAAC ACGGACCAAG GAGTCTAACA TGTGCGCGAG TCGTAGGGAC TCGAATCCCG
AAGGCGCAAT GAAAGTGAAG GCTGCCCACG G-TGGCCGAG GCAGGATCCC GTCGTGGGCG
CACTGCCGGC CCGTCTCGAT AGCGGCGTAG GCGTGCCCAA CGTTCGTCTA CGAGGCGGAG
CAAGAGCGTA CACGTTGGTA CCCGAAAGAT GGTGAACTAT GCCTGAGCAG GACGAAGTCA
GAGGAAACTC TGATGGAGGT CCGCAGCGAT TCTAGAAAGT CCACTGGTGG CAAAGCCCCC
AGAAAACAAC TGGCCACCAA GGCCGCACGT AAGAGTGCAC CGGCTACTGG TGGTGTGAAG
AAACCACACA GATACAGGCC CGGAACCGTC GCTCTCCGTG AAATCAGACG ATACCAGAAG
AGCACTGAAC TGCTCATCAG AAAATTGCCC TTCCAGCGTC TGGTCCGTGA AATCGCCCAG
GACTTCAAGA CCGACCTGCG ATTCCAGAGC TCTGCCGTTA TGGCCCTTCA GGAGGCCAGC
GAGGCTTACT TGGTCGGACT TTTCGAGGAC ACCAACTTGT GCGCTATCCA CGCCAAGCGT
TACTTTATAC TTCTTATTTG GCATTTGGTC CGGTTTGGTT GGAACTTCTT TTAGAGTTCT
AATTCGGCTG GAGCTATCTC GCCCTGGGGC TTTTTTAGGA GACGATCATT TATATAATGT
CATTGTCACC GCCCACGCAT TTGTAATAAT TTTTTTTCTC GTTATACCTA TAATGGTGGG
TGGCTTTGGA AATTGGTTGG TTCCACTTAT ATTAACTTCT TCTGATATGG CTTTTCCTCG
TATAAATAAC CTCAGGTTTT GGTTGTTACC TCCTTCTTTA TTTCTCCTTC TAAGTTCTGC
CTTCGTTGAG AGAGGGGTGG GCACTGGGTG GACGGTGTAC CCTCCTTTAT CTGGGAACTT
AACACATAGA GGGGGGTCTG TGGATTATGG TATTTTTTCT TTACATTTAG CGGGTGTATC
TTCTATTTTG GGGGCCATCA ACTTCTTGGT AACAATGATT AACATACGTC CTGAGATTAT
TGAGTTGAAG CGGGTAACCC TTTTTGTATG GTCTATCGCT ATCACCGCTT TTTTACTGGT
AGCTGCCATG CCTGTTTTAG CTGGTGCTAT TACGATATTA CTTACTGATC GCAATTTTAA
TACTTCATTT TTCGACCCAT CTGGTGGTGG TGATCCCATT TTATTCGTTC ATTTATTTT

>Pseudopythina_aff_nodosa
TAGTCATATG CTTGTCTCAA AGATTAAGCC ATGCATGTCT AAGTACACGC CAGATTAATG
GTGAAACTGC GAATGGCTCA TTAAATCAGT TATGGTTCCT TAGATCGTAC AATCCTACTT
GGATAACTGT GGCAATTCTA GAGCTAATAC ATGCGTCAAA GCTCCGACCT TCGGGGAAGA
GCGCTTTTGT TAGCAAAACC AATC-GGCCG TCTGTTGACT CTGAACAACT T--TGCTGAT
CGCACGGCCT AGCGCCGGCG ACGTATCTTT TGAATGTCTG CCCTATCAAC TGACGATGGT
ACGTGCTATG CCTACCATGG TTGTAACGGG TAACGGGGAA TCAGGGTTCG ATTCCGGAGA
GGGAGCATGA GAAACGGCTA CCACATCTAA GGAAGGCAGC AGGCGCGCAA ATTACCCAAT
CCCGACACGG GGAGGTAGTG ACGAAAAATA ACAATACGGG ACTCTTTCGA GGCCCCGTAA
TTGGAATGAG TACACTTTAA ATCCTTTAAC GAGGATCCAT TGGAGGGCAA GTCTGGTGCC
AGCAGCCGCG GTAATTCCAG CTCCAATAGC GTATATTAAA GTTGTTGCAG TTAAAAAGCT
CGTAGTTGGA TCTCGGGTGC AGGCTTGCGG TCCGCCTCGC GGCGGCTGCT CGTCCTAGCA
GCCTAGCTTC GGTGTCCCTT GGTGCTCTTG ACCGAGTGTC GGTGGCCGGA ACGTTTACTT
TGATGAAATT AGAGTGTTTA AAGCGCGGGC GTTTGCCCGC ATAATGGTGC ATGGAATGAT
GGAATAGGAC CTCGGTTCTA TTTTGTTGGT TTTCGGAGAG GTAATGATCA AGAGGGACAG
ACGGGGGCAT TCGTATTGCA GCGTTAGAGG TGAAATTCTT GGATCGTTGC AAGACGGACG
ACAGCGAAAG CATTTGCCAA GAATGTTTTC CTTAATCAAG AACGAAAGTC AGAGGCTCGA
AGACGATCAG ATACCGTCGT AGTTCTGACC ATAAACCATG CCAACTGGCA ATCCGCCGGA
GTTACTACAA TGACTCGGCG AGCAGCCCCC GGGAAACCAA AGTTTCTGGG TTCCGGGGGG
AGTATGGTTG CAAAGCTGAA ACTTAAAGGA ATTGACGGAA GGGCACCACC AGGAGTGGAG
CCTGTGGCTT AATTTGACTC AACACGGGAA ACCTCACCCG GCCCGGACAC CGTTAGGATT
GACAGATTGA GAGCTCTTTC TTGATTCGGT GGGTGGTGGT GCATGGCCGT TCTTAGTTGG
TGGAGCGATT TGTCTGGTTA ATTCCGATAA CGAACGAGAC TCTAGCCTAC TAAATAGTTC
GAGGATATAT TACCTCGCAA CTTCTTAGAG GGACAGGTGG CGTATAGCCA CACGAGATTG

AGCAATAACA GGTCTGTGAT GCCCTTAGAT GTTCGGGGCC GCACACGCGC TACATTGAAT
GGATCAACGT GCGTCTAGCC TTGCCCGAAA GGGCTGGGAA ACCCGTTGAA ACCCATTCGT
GATAGGGATT GGGGCTTGCA ATTATTTCCC ATGAACGAGG AATTCCTAGT AAGCGCGAGT
CATCAGCTCG CGTTGATTGC GTCCCTGCCC TTTGTACACA CCGCCCGTCG CTACTACCGA
TCGTTCCAGT TAATGAACGC CTCGGATTGG TTAAGCGGG- -GAAACCTGC TCGCGCGCCG
AGAAGAAGTG TAAGTTATCT GGGATAGAGG TCGTAAAAGT CGTAACAAGG TATCCATTAA
TAAGCGGAGG AAAAGAGACT AACTAGGATT CCCCTAGTAA CGGCGAGTGA AGCGGGAAGA
GCCCAGCACC GAATCCCCCG GCGTCTGGCC GCCGACGGGA CATGTGGTGT TAGCGGGAGT
CTTGTGTCGG CGTGTTTCGG CACCAAAGTC CACCTGATCG TGGCAATCCA AGGCGGGTGA
GAGGCCCGTT CCGGTGTCGG ACGCGTCGGA CTCTCTCCAG GAGTCGGGTT GTTTGAGAAT
GCAGCCCAAA GTGGGTGGTA AACTCCATCT AAGGCTAAAT ACTGACACGA GTCCGATAGA
GGACAAGTAC CGTGAGGGAA AGTTGAAAAG AACTTTGAAG AGAGAGTTCA AGAGTACGTG
AAACCGCATA GAGTCAAACG GGTGGATCCG CAAGGTGTCG GACCGGGGAA TTCAGCGGTG
CACTTTCTCC GACGAGAGCC ACGACCGGGT CCGCTGCTCG CAGCGCGAGA AGGTTTCCCG
GAGCTT-GGG AGCATACAGC TCGTCGGCCT CAGGCATCTA TGTCGTGGAT CCGAGGACGC
GCCGCGCGTT GGGTCCCGGG TCGTCCTCGT GCGTTCGACT TTCGTGGGTA GTGCGTCGTA
ACCGCGTGCT GGCTCGGTGG --AGGGTCAG TGGCGAATCA GTCGGTCCTC CACCCGACCC
GTCTTGAAAC ACGGACCAAG GAGTCTAACA TGTGCGCGAG TCATAGGGAC TCGAATCCCG
AAGGCGCAAT GAAAGTGAAG GCCGCCTACG GTTGGCCGAG GCAGGATCCC GTCGTGGGCG
CACTGCCGGC CCGTCTCGAT AGCGGCGTAG GCGCGCCCAA CGTTCGTCTA CGAGGCGGAG
CAAGAGCGTA CACGTTGGTA CCCGAAAGAT GGTGAACTAT GCCTGAGCAG GACGAAGTCA
GAGGAAACTC TGATGGAGGT CCGCAGCGAT TCAAGAAAGT CCACTGGTGG CAAAGCCCCA
AGGAAACAAC TGGCCACCAA GGCCGCACGT AAAAGTGCAC CAGCTACTGG TGGTGTTAAG
AAACCACACA GGTACAGGCC CGGAACCGTC GCTCTTCGTG AAATCAGAAG GTACCAGAAG
AGCACTGAGC TTCTGATCAG AAAACTGCCC TTCCAGCGTC TCGTCCGTGN GATTGCTCAG
GACTTCAAGA CTGATCTCCG ATTTCAGAGC TCTGCCGTTA TGGCACTGCA GGAAGCCAGT
GAAGCTTACT TGGTCGGACT TTTTGAAGAC ACCAACTTGT GCGCCATTCA CGCCAAGCGT
AACGTTGTAC TTTCTCTTTG GTATTTGGTC TGGCTTGGTG GGAACTTCTT TTAGTGTTTT
AATTCGTTTG GAGTTATCTC GTCCAGGTGC TTTTCTAGGT GATGATCATT TATATAATGT
TATTGTGACT GCTCATGCTT TTGTTATGAT TTTTTTTTTA GTTATACCAA TAATGGTAGG
GGGGTTTGGT AATTGATTAG TACCATTAAT GTTGACTTCT CCGGACATAG CTTTCCCCCG
GATGAATAAT ATAAGGTTTT GGTTACTTCC TCCAGCACTC TTCCTTCTCC TTAGGTCGGC
ATTTGTGGAA AGGGGGGCAG GGACTGGGTG AACTGTATAT CCTCCCTTAT CTAGGAATAT
TACTCATAGG GGCGGGTCGG TTGATTACGC CATTTTCTCC CTTCATTTAG CGGGTGTTTC
GTCAATTCTT GGTGCTATTA ATTTTTTAGC TAGGACCATT AATATGCGCC CAGAGGTGAT
GGAGTTGAAG CGTGTGACTT TGTTTGTGTG GTCTATCGCT ATTACTGCAT TCCTGTTGGT
GGTGGCTATA CCGGTGTTGG CGGGTGCCAT TACGATGCTT CTTACTGATC GTAATTTTAA
CACTTC---- ---------- ---------- ---------- ---------- ---------

>Pythina_deshayesiana
TAGTCATATG CTTGTCTCAA AGATTAAGCC ATGCATGTCT AAGTACACGC CAGATTAATG
GTGAAACTGC GAATGGCTCA TTAAATCAGT TATGGTTCCT TAGATCGTAC AATCCTACTT
GGATAACCGT GGCAATTCTA GAGCTAATAC ATGCGTCAAA GCTCCGACCT TCGGGGAAGA
GCGCTTTTGT TAGCAAAACC AATCCGGTCG GTTGTTGACT CTGAACAACT TTGTGCTGAT
CGCACGGCCT AGCGCCGGCG ATGTATCTTT CGAATGTCTG CCCTATCAAC TGTCGATGGT
ACGTGCTATG CCTACCATGG TTGTAACGGG TAACGGGGAA TCAGGGTTCG ATTCCGGAGA
GGGAGCATGA GAAACGGCTA CCACATCCAA GGAAGGCAGC AGGCGCGCAA ATTACCCAAT
CCCGACACGG GGAGGTAGTG ACGAAAAATA ACAATACGGG ACTCTTTCGA GGCCCCGTAA
TTGGAATGAG TACACTTTAA ATCCTTTAAC GAGGATCAAT TGGAGGGCAA GTCTGGTGCC
AGCAGCCGCG GTAATTCCAG CTCCAATAGC GTATATTAAA GTTGTTGCAG TTAAAAAGCT

CGTAGTTGGA TCTCGGGTGC AGGCTTGCGG TCCGCCTCGC GGCGGCTGCT CGTCCTGGCA
GCCTAGCTGC GGTATCCCTT GGTGCTCTTG ACTGAGTGTC GGTGGCCGGA ACGTTTACTT
TGATGAAATT AGAGTGTTCA AAGCGTGGGC GCTTGCCCGC ATAATGGTGC ATGGAATGAT
AGAATAGGAC CTCGGTTCTA TTTTGTTGGT TTTCGGAGAG GTAATGATTG AGAGGGACAG
ACGGGGGCAT TCGTATTGCG GCGTTAGAGG TGAAATTCTT GGATCGCCGC AAGACGGACT
ACAGCGAAAG CATTTGCCAA GAATGTTTTC CTTAATCAAG AACGAAAGTC AGAGGCTCGA
AGACGATCAG ATACCGTCGT AGTTCTGACC ATAAACCATG CCGACTGGCA ATCCGCCGGA
GTTACTACAA TGACTCGGCG AGCAGCCCCC GGGAAACCAA AGTTTCTGGG TTCCGGGGGG
AGTATGGTTG CAAAGCTGAA ACTTAAAGGA ATTGACGGAA GGGCACCACC AGGAGTGGAG
CCTGTGGCTT AATTTGACTC AACACGGGAA ACCTCACCCG GCCCGGACAC CGTTAGGATT
GACAGATCGA GAGCTCTTTC TTGATTCGGT GGGTGGTGGT GCATGGCCGT TCTTAGTTGG
TGGAGCGATT TGTCTGGTTA ATTCCGATAA CGAACGAGAC TCTAGCCTAC TAAATAGTTC
GAGGATATAT AACCTCGCAA CTTCTTAGAG GGACAGGTGG CGTATAGCCA CACGAGATTG
AGCAATAACA GGTCTGTGAT GCCCTTAGAT GTCCGGGGCC GCACACGCGC TACATTGAAT
GGATCAACGT GCGTCTAGCC TTGCCCGAAA GGGCTGGGAA ACCCGTTGAA ACCCATTCGT
GATAGGGACT GGGGCTTGCA ATTATTTCCC ATGAACGAGG AATTCCTAGT AAGCGCGAGT
CATCAGCTCG CGTTGATTGC GTCCCTGCCC TTTGTACACA CCGCCCGTCG CTACTACCGA
TCGCTCCAGT TAATGAACGC CTCGGATTGG TTAAGCGGGT TTCGGCCTGC TCGCGTGCCG
AGAAGACGTG CAAATTATCT GGAGTAGAGG GAGTAAAAGT CGTAACAAGG TATCCATCAA
TAAGCGGAGG AAAAGAGACT AACAAGGATT CCCCCAGTAA CGGCGAGTGA AGCGGGAAAA
GCCCAGCACC GAATCCCCCG GCGTCTGGCC GCCGACGGGA CCTGTGGTGT TAGCGGGAGT
CTTTTGTCGG CGTGCTTCGG CACCAAAGTC CACCTGATCG TGGCAACCCC TGGCGGGTGA
GAGGCCCGTT CCGGTGTCGG ACGCGTTCGA CTCTCTCCAG GAGTCGGGTT GTTTGAGAAT
GCAGCCCAAA GTGGGTGGTA AACTCCACCT AAGGCTAAAT ACGGACACGA GTCCGATAGA
GGACAAGTAC CGTGAGGGAA AGTTGAAAAG AACTTTGAAG AGAGAGTTCA AGAGTACGTG
AAACCGCATA GAGGCAAACG GGTGGATCCG CAAGGTGTTG GACCGGGGAA TTCAGCGGTG
CACTTTCTCC GACGAGAGCC ACGACCGGGT CTGCTGCTCG AGGCGTGCGA AGGTTTCCAG
GCGCTCCGGG AGCTTACAGC GCGCCGCCAT CGAGTCTGTA TGTCGCGGGC CCGAGGACGC
GCCGCGCGCC TGGCACCGGG TCGTCTTTGC GCGTTCGACT GTTCTCGGCA GTGCGCTGCG
ACCGCGTGCG GTCCCGGCG- TGAGGGTCGG TGGCGAATCA GTCGGTGCTC CACCCGACCC
GTCTTGAAAC ACGGACCAAG GAGTCTAACA TGTGCGCGAG TCATGGGGAC CCGAATCCCG
AAGGCGCAAT GAAAGTGAAG GCCGCCCACG GCTGGCCGAG GCAGGATCCC GTCGCGGGCG
CACTGCCGGC CCGTCTCGAT AGCGGCGCGG TCGCGGCCTG CGTTCGTCTA CGAGGCGGAG
CAAGAGCGTA CACGTTGGTA CCCGAAAGAT GGTGAACTAT GCCTGAGCAG GACGAAGTCA
GAGGAAACTC TGATGGAGGT CCGCAGCGAT TC???????? ?????????? ??????????
?????????? ?????????? ?????????? ?????????? ?????????? ??????????
?????????? ?????????? ?????????? ?????????? ?????????? ??????????
?????????? ?????????? ?????????? ?????????? ?????????? ??????????
?????????? ?????????? ?????????? ?????????? ?????????? ??????????
?????????? ?????????? ?????????? ?????????? ?????????? ??????????
?????????? ?????????? ?????????? ?????????? ?????????? ??????????
?????????? ?????????? ?????????? ?????????? ?????????? ??????????
?????????? ?????????? ?????????? ?????????? ?????????? ??????????
?????????? ?????????? ?????????? ?????????? ?????????? ??????????
?????????? ?????????? ?????????? ?????????? ?????????? ??????????
?????????? ?????????? ?????????? ?????????? ?????????? ??????????
?????????? ?????????? ?????????? ?????????? ?????????? ??????????
?????????? ?????????? ?????????? ?????????? ?????????? ??????????
?????????? ?????????? ?????????? ?????????? ?????????? ??????????
?????????? ?????????? ?????????? ?????????? ?????????? ??????????

?????????? ?????????? ?????????? ?????????? ?????????? ?????????

>Salpocola_philippinensis
TAGTCATATG CTTGTCTCAA AGATTAAGCC ATGCATGTCT AAGTACACGC CAGATACAAG
GTGAAACTGC GAATGGCTCA TTAAATCAGT TATGGTTCCT TAGATCGTAC AATCCTACTT
GGATAACTGT GGCAATTCTA GAGCTAATAC ATGCATCAAA GCTCCGACCT CCGGGGAAGA
GCGCTTTTGT TAGCAAAACC AATCCGGCCG GTTGTTGACT CTGAACAACT TTGTGCTGAT
CGCACGGCCT AGCGCCGGCG ACGTATCTTT CGAATGTCTG CCCTATCAAC TGTCGATGGT
ACGTGCTATG CCTACCATGG TTGTAACGGG TAACGGGGAA TCAGGGTTCG ATTCCGGAGA
GGGAGCATGA GAAACGGCTA CCACATCCAA GGAAGGCAGC AGGCGCGCAA ATTACCCAAT
CCCGACACGG GGAGGTAGTG ACGAAAAATA ACAATACGGG ACTCTTTCGA GGCCCCGTAA
TTGGAATGAG TACACTTTAA ATCCTTTAAC GAGGATCCAT TGGAGGGCAA GTCTGGTGCC
AGCAGCCGCG GTAATTCCAG CTCCAATAGC GTATATTAAA GTTGTTGCAG TTAAAAAGCT
CGTAGTTGGA TCTCGGGTGC AGGCTTGCGG TCCGCCTCGC GGTGGCTGCT CGTCCTTGCA
GCCTAGCTGC GGTGTCCCTT GGTGCTCTTG ATTGAGTGTC GGTGGCCGCA ACGTTTACTT
TGATGAAATT AGAGTGTTCA AAGCGTGGGC GTTTGCCCGC ATAATGGTGC ATGGAATGAT
GGAATAGGAC CTCGGTTCTA TTTTGTTGGT TTTCGGAGAG GTAATGATTG AGAGGGACAG
ACGGGGGCAT TCGTATTGCG GCGTTAGAGG TGAAATTCTT GGATCGCCGC AAGACGGACG
AGAGCGAAAG CATTTGCCAA GAATGTTTTC CTTAATCAAG AACGAAAGTC AGAGGCTCGA
AGACGATCAG ATACCGTCGT AGTTCTGACC ATAAACCATG CCAACTGGCA ATCCGCCGGA
GTTACTACAA TGACTCGGCG AGCAGCCCCC GGGAAACCAA AGTTTCTGGG TTCCGGGGGG
AGTATGGTTG CAAAGCTGAA ACTTAAAGGA ATTGACGGAA GGGCACCACC AGGAGTGGAG
CCTGTGGCTT AATTTGACTC AACACGGGAA ACCTCACCCG GCCCGGACAC TGTAAGGATT
GACAGATTGA GAGCTCTTTC TTGATTCGGT GGGTGGTGGT GCATGGCCGT TCGTAGTTGG
TGGAGCGATT TGTCTGGTTA ATTCCGATAA CGAACGAGAC TCTAGCCTAC TAAATAGTTC
GAGGCTATAT AGCCTCGCAA CTTCTTAGAG GGACAGGTGG CGTATAGCCA CACGAGATTG
AGCAATAACA GGTCTGTGAT GCCCTTAGAT GTTCGGGGCC GCACACGCGC TACATTGAAT
GGATCAACGT GCGTCTAGCC TTGCCCGAAA GGGCTGGGAA ACCCGTTGAA ACCCATTCGT
GATAGGGACT GGGGCTTGCA ATTATTTCCC ATGAACGAGG AATTCCTAGT AAGCGCGAGT
CATCAGCTCG CGTTGATTGC GTCCCTGCCC TTTGTACACA CCGCCCGTCG CTACTACCGA
TCGCTCCAGT TAATGAACGC TTCGGATTGG TTAAGCGGGT TTCGGCCTGC TCGCGTGCCG
AGAAGATGTG TGAATTATCC GGGGTAGAGG TCGTAAAAGT CGTAACAAGG TATCCATTAA
TAAGCGGAGG AAAAGAGACT AACTAGGATT CCCCTAGTAA CGGCGAGTGA AGCGGGAAGA
GCCCAGCACC GAATCCCCCA GCGTCTGGCC GCTGACGGGA AATGTGGTGT TAGCGGGAGT
CCTACGTCGG CGTGTTCGGT TGCCAAAGTC CACCTGATCG TGGCAACCCC AGGCGGGTGA
GAGGCCCGTT TCGGTGACCG GCGCGTTGGA CTCTCTCCAG GAGTCGGGTT GTTTGAGAAT
GCAGCCCAAA GTGGGTGGTA AACTCCATCT AAGGCTAAAT ACTGACACGA GTCCGATAGA
GGACAAGTAC CGTGAGGGAA AGTTGAAAAG AACTTTGAAG AGAGAGTTCA AGAGTACGTG
AAACCGCATA GAGTCAAACG GGTGGATCCG CAAGGTGTTG GACCGGGGAA CTCAGCGGCG
CACTTTCTCC GACGAGAGCC ACGACCGGGT CCGCTGCTCG AGGTGTGACA AGGTTTTCCG
G-CGGTTGGG AGCTTACAGG TCGCCGCCCG CGAGCTGGCA C---GCGGTC CCGAGGACGC
GCCGTGCGCT CGGCTCTGGG CCGTCCTGGC GTGTTCGACC T--TTCAGCA GTGTGCCGTA
ACCGCTCGCC GGTTCGGTGT TGAGGGTCGG TGGCGAATCA GTCGGTACTC CACCCGACCC
GTCTTGAAAC ACGGACCAAG GAGTCTAACA TGTGCGCGAG TCATAGGGAC CCGAATCCCG
AAGGCGCAAT GAAAGTGAAG GTCGTCCTCG GTCGGCCGAG GCAGGATCCC GTCGCGGGCG
CACTGCCGGC CCGTCTCGAT AGCGGCGGTG GGTCGGCCTG CCTTCGTCTG CGAGGCGGAG
CAAGAGCGTA CACGTTGGTA CCCGAAAGAT GGTGAACTAT GCCTGAGCAG GACGAAGTCA
GAGGAAACTC TGATGGAGGT CCGCAGCGAT TCCCGAAAGT CCACAGGAGG AAAGGCTCCC
AGGAAGCAGC TGGCCACCAA GGCTGCACGT AAGAGTGCCC CAGCCACTGG TGGAGTGAAG
AAACCCCACA GGTACAGGCC CGGTACCGTG GCTCTTCGAG AGATCAGGAG ATACCAGAAG

AGCACTGAGC TCCTGATCAG AAAACTGCCA TTCCAGCGTC TGGTCCGTGA GATCGCCCAG
GACTTCAAGA CTGACCTCCG ATTCCAGAGC TCTGCCGTCA TGGCTCTGCA GGAGGCCAGC
GAGGCCTACC TTGTTGGTCT TTTTGAGGAC ACCAACTTGT GTGCCATCCA CGCCAAGCGT
AACTTTGTAT TTTTTGTTTG GTATTTGGTC TGGTTTGGTG GGTACTTCTT TCAGTGTGTT
AATTCGGTTG GAGCTTTCAC GACCTGGGGC TTTTTTAGGG GATGACCATT TATATAATGT
AATTGTGACC GCTCATGCGT TCGTAATAAT TTTTTTCTTA GTGATGCCTA TAATGGTTGG
GGGTTTTGGT AATTGGTTGG TGCCTTTAAT GTTAACGTCT CCTGACATGG CGTTTCCGCG
AATGAATAAT ATAAGGTTTT GGCTTTTACC ACCTGCCATA TTTTTATTGT TGAGTTCTGC
TTTTGTAGAG AGGGGGGCGG GTACTGGTTG GACGGTTTAT CCACCTTTAT CAGGAAATAT
CACTCACAGT GGGGGTTCGG TGGATTATGC TATTTTTTCT CTTCATTTGG CTGGTGTTTC
CTCCATTTTA GGGGCTATTA ATTTTTTGGC AAGAACTGTT AACATGCGGC CAGAGATTAT
AGAGTTGAAG CGTGTTACTT TGTTTGTTTG GTCTATTGCT ATTACGGCAT TTTTGTTGGT
AGTGGCTATG CCTGTTTTGG CTGGGGCAAT TACTATACTG TTAACGGATC GTAATTTCAA
TACTTCTTTT TTTGATCCTT CTGGAGGTGG TGACCCAATT CTTTTTGTGC ACTTGTTTT

>Basterotia_carinata
TAGTCATATG CTTGTCTCAA AGATTAAGCC ATGCATGTCC AAGTACACGC CGAATTCATG
GCGAAACTGC GAATGGCTCA TTAAATCAGT TATGGTTCCT TAGATCGTAC AATCCTACTC
GGATAACTGT GGCAATTCTA GAGCTAATAC GTGATGAGCC GCTCCGACCT TCGGGGAAGA
GCGCTTTTGT TAGTAAGACC AACCCGGCCG GTTGTTGACT CTGAACAACT TTGTGCTGAT
CGCACGGCCT CGCGCCGGCG ACGTATCTTT CGAATGTCTG ACCTATCAAC TGGCGATGGT
ACGTGCTATG CCTACCATGG TTTTAACGGG TAACGGGGAA TCAGGGTTCG GTTCCGGAGA
GGGAGCATGA GAAACGGCTA CCACATCCAA GGAAGGCAGC AGGCGCGCAA ATTACCCAAT
CCCGACACGG GGAGGTAGTG ACGAAAAATA ACAATACGGG ACTCTTTCGA GGCCCCGTAA
TTGGAATGAG TACACTTTAA ATCCTTTAAC GAGGATCCAT TGGAGGGCAA GTCTGGTGCC
AGCAGCCGCG GTAATTCCAG CTCCAATAGC GTATATTAAA GTTGCTGCAG TTAAAAAGCT
CGTAGTTGGA TCTCGGATGC AGGCTTGCGG TCCGCCTCGC GGCGGCTGCT CGTCCTGTCA
GCCTAGCTTC GGTGTCCCTT GGTGCTCTTG ACCGAGTGTC GGTGGCCGGA ACGTTTACTT
TGATGAAATT AGAGTGCTTA AAGCGCGGGC GATTGCCCGT ATAATGGTGC ATGGAATGAT
AGAAAAGGAC CTCTGTTCTA TTTTGTTGGT TTTCGGAGAG GTAATGATTG AGAGGGACGG
CCGGGGGCAT TCGTATTGCG GCGTTAGAGG TGAAATTCTT GGATCGTCGC AAGACGGACT
ACAGCGAAAG CATTTGCCAA GAATGTCTTC ATTAATCAAG AACGAAAGTC AGAGGCTCGA
AGACGATCAG ATACCGTCGT AGTTCTGACC ATAAACTATG CCAACTGGCA TTCCGCCGGA
GTTACTACAA TGACTCGGCG AGCAGCCCCC GGGAAACCAA AGTTTCTGGG TTCCGGGGGG
AGTATGGTTG CAAAGCTGAA ACTTAAAGGA ATTGACGGAA GGGCACCACC AGGAGTGGAG
CCTGTGGCTT AATTTGACTC AACACGGGAA ACCTCACCCG GCCCGGACAC CGTTAGGATT
GACAGATTGA GAGCTCTTTC TTGATTCGGT GGGTGGTGGT GCATGGCCGT TCTTAGTTGG
TGGAGCGATT TGTCTGGTTA ATTCCGATAA CGAACGAGAC TCTAGCCTAC TAAATAGTTC
GAGGATAGAC ATCCTCGCAA CTTCTTAGAG GGACAGGTGG CGTTTAGCCA CACGAGATTG
AGCAATAACA GGTCTGTGAT GCCCTTAGAT GTCCGGGGCC GCACACGCGC TACATTGAAT
GGATCAGCGT GCGTCTCGCC TTGCCCGAAA GGGCTGGGAA ACCCGTTGAA ACCCATTCGT
GATGGGGATT GGGGCTTGCA ATTATACCCC ATGAACGAGG AATTCCTAGT AAGCGCGAGT
CATCAGCTCG CGTTGATTGC GTCCCTGCCC TTTGTACACA CCGCCCGTCG CTACTACCGA
TCGTTCCTGT TAATGAACGC TTCGGATTGG TTAGGCGAGT TTCGGCTTGC TCACGCGCCG
AGAAGATTCG TAAATTATCA GGAATAGAGG TCGTAAAAGT CGTAACAAGG TATCCATCAA
TAAGCGGAGG AAAAGAGACT AACTAGGATT CCCCCAGTAA CGGCGAGTGA AGCGGGAAGA
GCCCAGCACC GAATCCCCCG GCGTCTGGTC GCCGGCGGGA ACTGTGGTGT CCGCGGGAGT
CTCGTGTCGG CGCGATTCGT CGCCAAAGTC CACCTGATCG TGGCAACCCA GTGCGGGTGA
GAGGCCCGTT ACGGTGACGG CCGCGTCGGA CGCTCTCCAG GAGTCGGGTT GTTTGAGAAT
GCAGCCCCAA GTGGGTGGTA AACTCCATCT AAGGCTAAAT ACTGACACGA GTCCGATAGA

GGACAAGTAC CGTGAGGGAA AGTTGAAAAG AACTTTGAAG AGAGAGTTCA AGAGTACGTG
AAACCGTATA GAGTCAAACG GGTGGATCCG CAAGGTGTCG CACCGGGGAA TTCAGCGGTG
CACTTTCTCC GGCGAGAGCC ACGACCGGGT TCGCGGCGCG CAGCGCGAGA AGGTGTGCCG
G--CACC-GG GATTTACAGC TCGTCGGCCT CGCGTCGTAT CGTCGCGGAT CCGAGGACGC
GCCGCGCGCC CGGTTCCGGG CCGTCTTCGC GGTTTCGACC TTTCCGGGCA GTGCGCTGAA
ACCGCTCGCG GGCTCGGTGT TGAGGGTCGG TGGCGAATCG GTCGGTACTC CACCCGACCC
GTCTTGAAAC ACGGACCAAG GAGTCTAACA TGTGCGCGAG TCACAGGGAC GCGAATCCCG
AAGGCGCAAT GAAAGTGAAG GCCGCCCTCG GCTGGCCCAG GCAGGATCCC GTCGTGGGCG
CACTGCCGGC CCGTCTCGAT AGCGGCGCGT GG-GGTCGCG CGTTCGTCTA CGAGGCGGAG
CTAGAGCGTA CACGTTGGTA CCCGAAAGAT GGTGAACTAT GCCTGAGCAG GACGAAGTCA
GAGGAAACTC TGATGGAGGT CCGCAGCGAT TCTAGAAAGT CCACTGGTGG AAAAGCTCCA
CGTAAACAAC TGGCCACAAA GGCCGCACGT AAGAGTGCAC CGGCCACTGG TGGTGTAAAG
AAACCACACA GGTACAGGCC TGGAACCGTC GCCCTCCGTG AAATCAGAAG GTACCAGAAG
AGCACCGAGC TTCTGATCAG GAAGCTGCCA TTCCAGCGTC TTGTCCGTGA GATCGCCCAG
GACTTCAAGA CTGATCTCCG CTTCCAGAGC TCTGCCGTCA TGGCACTGCA GGAGGCCAGC
GAGGCCTACT TGGTCGGACT GTTCGAGGAC ACGAACTTGT GCGCTATCCA CGCCAAGCGT
GACTTTATAT TTTATTTTTG GAATTTGATC TGGGTTGGTT GGTACGTCTT TTAGTGTACT
AATTCGTTTA GAATTATCCC GCCCTGGTGC TTTTTTAGGT GATGATCATT TGTATAATGT
GATTGTTACT GCCCATGCCT TTGTAATAAT TTTTTTCTTG GTTATGCCAA TGATAGTTGG
GGGTTTTGGA AATTGGTTAG TGCCTTTAAT ATTAACTTCT CCTGATATGG CTTTTCCTCG
AATGAATAAT ATAAGGTTTT GGTTATTACC TCCAGCCTTA TTTCTTTTGT TGAGATCTGC
TTTTGTTGAA AGAGGTGTAG GAACTGGGTG GACTGTTTAT CCTCCCCTAT CGTCTAATGT
TACACATAGT GGTGCTTCAG TAGATTATGG TATTTTTTCT CTTCATTTGG CGGGCGTTTC
TTCTATTTTA GGCGCTATTA ATTTTTTGGC TACTACCATT AATATGCGCC CTGAGATTAT
GGAGTTGAAG CGTGTGACGC TTTTTGTCTG GTCTGTTGCT ATCACTGCTT TCTTACTGGT
AGTTGCAATA CCTGTGCTGG CCGGTGCAAT TACAATGCTT TTAACGGATC GCAATTTTAA
TACGTCTTTT TTTGATCCTT CA-------- ---------- ---------- ---------

>Basterotia_gouldi
TAGTCATATG CTTGTCTCAA AGATTAAGCC ATGCATGTCT AAGTACACGC CAAATTCATG
GTGAAACTGC GAATGGCTCA TTAAATCAGT TATGGTTCCT TAGATCGTAC AATCCTACTC
GGATAACTGT GGCAATTCTA GAGCTAATAC GTGATGAGCC GCTCCGACCT TCGGGGAAGA
GCGCTTTTGT TAGTAAGACC AACCCGGCCG GTTGTTGACT CTGAACAACT TTGTGCTGAT
CGCACGGCCT CGCGCCGGCG ACGTATCTTT CGAATGTCTG ACCTATCAAC TGGCGATGGT
ACGTGCTATG CCTACCATGG TTTTAACGGG TAACGGGGAA TCAGGGTTCG GTTCCGGAGA
GGGAGCATGA GAAACGGCTA CCACATCCAA GGAAGGCAGC AGGCGCGCAA ATTACCCAAT
CCCGACACGG GGAGGTAGTG ACGAAAAATA ACAATACGGG ACTCTTTCGA GGCCCCGTAA
TTGGAATGAG TACACTTTAA ATCCTTTAAC GAGGATCCAT TGGAGGGCAA GTCTGGTGCC
AGCAGCCGCG GTAATTCCAG CTCCAATAGC GTATATTAAA GTTGCTGCAG TTAAAAAGCT
CGTAGTTGGA TCTCGGATGC AGGCTTGCGG TCCGCCTCGC GGCGGCTGCT CGTCCTGTCA
GCCTAGCTTC GGTGTCCCTT GGTGCTCTTG ACCGAGTGTC GGTGGCCGGA ACGTTTACTT
TGATGAAATT AGAGTGCTTA AAGCGCGGGC GTTTGCCCGT ATAATGGTGC ATGGAATGAT
GGAATAGGAC CTCTGTTCTA TTTTGTTGGT TTTCGGAGAG GTAATGATTG AGAGGGACGG
CCGGGGGCAT TCGTATTGCG GCGTTAGAGG TGAAATTCTT GGATCGTCGC AAGACGGACT
ACAGCGAAAG CATTTGCCAA GAATGTCTTC ATTAATCAAG AACGAAAGTC AGAGGCTCGA
AGACGATCAG ATACCGTCGT AGTTCTGACC ATAAACTATG CCAACTGGCA TTCCGCCGGA
GTTACTACAA TGACTCGGCG AGCAGCCCCC GGGAAACCAA AGTTTCTGGG TTCCGGGGGG
AGTATGGTTG CAAAGCTGAA ACTTAAAGGA ATTGACGGAA GGGCACCACC AGGAGTGGAG
CCTGTGGCTT AATTTGACTC AACACGGGAA ACCTCACCCG GCCCGGACAC CGTTAGGATT
GACAGATTGA GAGCTCTTTC TTGATTCGGT GGGTGGTGGT GCATGGCCGT TCTTAGTTGG

TGGAGCGATT TGTCTGGTTA ATTCCGATAA CGAACGAGAC TCTAGCCTAC TAAATAGTTC
GAGGATAGAC ATCCTCGCAA CTTCTTAGAG GGACAGGTGG CGTTTAGCCA CACGAGATTG
AGCAATAACA GGTCTGTGAT GCCCTTAGAT GTCCGGGGCC GCACACGCGC TACATTGAAT
GGATCAGCGT GCGTCTCGCC TTGCCCGAAA GGGCTGGGAA ACCCGTTGAA ACCCATTCGT
GATGGGGATT GGGGCTTGCA ATTATACCCC ATGAACGAGG AATTCCTAGT AAGCGCGAGT
CATCAGCTCG CGTTGATTGC GTCCCTGCCC TTTGTACACA CCGCCCGTCG CTACTACCGA
TCGTTCCTGT TAATGAACGC TTCGGATTGG TTAGGCGAGT TTCGGCTTGC TCACGCGCCG
AGAAGATTCG TAAATTATCA GGAATAGAGG TCGTAAAAGT CGTAACAAGG TATCCATCAA
TAAGCGGAGG AAAAGAGACT AACTAGGATT CCCCTAGTAA CGGCGAGTGA AGCGGGAAAA
GCCCAGCACC GAATCCCCCG GCGTCTGGTC GCCGGCGGGA ACTGTGGTGT CCGCGGGAGT
CTCGTGTCGG CGCGTTTCGT CTCCAAAGTC CACCTGATCG TGGCAACCCA GTGCGGGTGA
GAGGCCCGTT ACGGTGACGG TCGCGTCGGA CGCTCTCCAG GAGTCGGGTT GTTTGAGAAT
GCAGCCCAAA GCGGGTGGTA AACTCCATCT AAGGCTAAAT ACTGACACGA GTCCGATAGA
GGACAAGTAC CGTGAGGGAA AGTTGAAAAG AACTTTGAAG AGAGAGTTCA AGAGTACGTG
AAACCGTATA GAGTCAAACG GGTGGATCCG CAAGGTGTCG CACCGGGGAA TTCAGCGGTG
CACTTTCTCC GGCGAGAGCC ACGACCGGGT TCGCGGCGCG CAGCGCGAGA AGGTGTTCCG
G--CACT-GG GATTTACAGC TCGTCGGCCT CGCGTCGTAT CGTCGCGGAT CCGAGGACGC
GCCGCGCGCC CGGCTCCGGG CCGTCTTCGC GGTTTCTACC TTTCCGGGCA GTGCGCTGAA
ACCGCTCGCG GGCTCGGTGT AGAGGGTCGG TGGCGAATCG GTCGGTACTC CACCCGACCC
GTCTTGAAAC ACGGACCAAG GAGTCTAACA TGTGCGCGAG TCACAGGGAC GCGAATCCCG
AAGGCGCAAT GAAAGTGAAG GCCGCCCTCG GCTGGCCTAG GCAGGATCCC GTCGTGGGCG
CACTGCCGGC CCGTCTCGAT AGCGGCGCGT GG-GGTCGCG CGTTCGTCTA CGAGGCGGAG
CTAGAGCGTA CACGTTGGTA CCCGAAAGAT GGTGAACTAT GCCTGAGCAG GACGAAGTCA
GAGGAAACTC TGATGGAGGT CCGCAGCGAT TCTAGAAAGT CCACTGGTGG AAAAGCTCCA
CGTAAACAAC TGGCCACAAA GGCCGCACGT AAGAGTGCAC CGGCCACTGG TGGCGTGAAG
AAACCACATA GGTACAGGCC TGGAACCGTC GCCCTCCGTG AAATCAGAAG GTACCAGAAG
AGCACTGAGC TTCTGATCAG AAAACTGCCA TTCCAGCGTC TCGTCCGTGA GATCGCCCAG
GACTTCAAGA CTGATCTCCG CTTCCAGAGC TCTGCCGTCA TGGCACTGCA GGAGGCCAGC
GAGGCTTACT TGGTCGGACT TTTCGAGGAC ACCACCTTGT GCGCTATCCA CGCCAAG---
---------- ---------- ---------- ---------- --TACTTCCT TTAGTGTTTT
AATTCGTTTA GAGCTTTCAC GGCCTGGTGC GTTTTTGGGA GATGATCACC TTTATAATGT
TATCGTTACA GCTCATGCGT TTGTAATAAT TTTTTTTTTA GTTATGCCTA TAATGGTTGG
GGGGTTTGGT AATTGGTNAG TCCCTTTAAT GTTAACTTCT CCCGATATGG CTTTCCCTCG
TATAAATAAC ATGAGATTTT GGCTTTTACC TCCGGCGCTT TTTCTTTTAC TAAGGTCTGC
ATTTGTGGAG AGCGGGGTTG GGACTGGTTG GACGGTGTAT CCTCCTCTTT CTTCAAATGT
TACGCATAGT GGTGCCTCTG TTGATTATGG GATTTTTTCT CTCCATTTAG CTGGGGTATC
CTCTATTTTA GGGGCTATTA ATTTTTTGGC GACTACTATT AATATGCGGC CAGAAATTAT
GGAGTTGAAG CGGGTAACGC TTTTTGTTTG ATCTGTAGCT ATTACTGC-- ----------
---------- ---------- ---------- ---------- ---------- ----------
---------- ---------- ---------- ---------- ---------- ---------

>Basterotia_sp
TAGTCATATG CTTGTCTCAA AGATTAAGCC ATGCATGTCT AAGTACACGC CAAATTCATG
GCGAAACTGC GAATGGCTCA TTAAATCAGT TATGGTTCCT TAGATCGTAC AATCCTACTC
GGATAACTGT GGCAATTCTA GAGCTAATAC GTGACTAGCC GCTCCGACCT TCGGGGAAGA
GCGCTTTTGT TAGTAAGACC AACCCGGCCG GTTGTTGACT CTGAACAACT TTGTGCTGAT
CGCACGGCCT CGCGCCGGCG ACGTATCTTT CGAATGTCTG ACCTATCAAC TGGCGATGGT
ACGTGCTATG CCTACCATGG TTTTAACGGG TAACGGGGAA TCAGGGTTCG GTTCCGGAGA
GGGAGCATGA GAAACGGCTA CCACATCCAA GGAAGGCAGC AGGCGCGCAA ATTACCCAAT
CCCGACACGG GGAGGTAGTG ACGAAAAATA ACAATACGGG ACTCTTTCGA GGCCCCGTAA

TTGGAATGAG TACACTTTAA ATCCTTTAAC GAGGATCCAT TGGAGGGCAA GTCTGGTGCC
AGCAGCCGCG GTAATTCCAG CTCCAATAGC GTATATTAAA GTTGCTGCAG TTAAAAAGCT
CGTAGTTGGA TCTCGGATGC AGGCTTGCGG TCCGCCTCGC GGCGGCTGCT CGTCCTGTCA
GCCTAGCTTC GGTGTCCCTT GGTGCTCTTG ACCGAGTGTC GGTGGCCGGA ACGTTTACTT
TGATGAAATT AGAGTGCTTA AAGCGCGGGC GATTGCCCGT ATAATGGTGC ATGGAATGAT
AGAATAGGAC CTCTGTTCTA TTTTGTTGGT TTTCGGAGAG GTAATGATTG AGAGGGACGG
CCGGGGGCAT TCGTATTGCG GCGTTAGAGG TGAAATTCTT GGATCGTCGC AAGACGGACT
ACAGCGAAAG CATTTGCCAA GAATGTCTTC ATTAATCAAG AACGAAAGTC AGAGGCTCGA
AGACGATCAG ATACCGTCGT AGTTCTGACC ATAAACTATG CCAACTGGCA ATCCGCCGGA
GTTACTACAA TGACTCGGCG AGCAGCCCCC GGGAAACCAA AGTTTCTGGG TTCCGGGGGG
AGTATGGTTG CAAAGCTGAA ACTTAAAGGA ATTGACGGAA GGGCACCACC AGGAGTGGAG
CCTGTGGCTT AATTTGACTC AACACGGGAA ACCTCACCCG GCCCGGACAC CGTTAGGATT
GACAGATTGA GAGCTCTTTC TTGATTCGGT GGGTGGTGGT GCATGGCCGT TCTTAGTTGG
TGGAGCGATT TGTCTGGTTA ATTCCGATAA CGAACGAGAC TCTAGCCTAC TAAATAGTTC
GAGGATAGAC ATCCTCGCAA CTTCTTAGAG GGACAGGTGG CGTTTAGCCA CACGAGATTG
AGCAATAACA GGTCTGTGAT GCCCTTAGAT GTCCGGGGCC GCACACGCGC TACATTGAAT
GGATCAGCGT GCGTCTCGCC TTGCCCGAAA GGGCTGGGAA ACCCGTTGAA ACCCATTCGT
GATGGGGATT GGGGCTTGCA ATTATACCCC ATGAACGAGG AATTCCTAGT AAGCGCGAGT
CATCAGCTCG CGTTGATTGC GTCCCTGCCC TTTGTACACA CCGCCCGTCG CTACTACCGA
TCGTTCCTGT TAATGAACGC TTCGGATTGG TTAGGCGAGT TTCGGCTTGC TCACGCGCCG
AGAAGATTCG TAAATTATCA GGAATAGAGG TCGTAAAAGT CGTAACAAGG TATCCATCAA
TAAGCGGAGG AAAAGATACT AACTAGGATT CCCCTAGTAA CGGCGAGTGA AGCGGGAAGA
GCCCAGCACC GAATCCCCCG ACGTCTGGTC GCCGGCGGGA ACTGTGGTGT CCGCGGGAGT
CTCGTGTCGG CGTGCTTCGT CACCAAAGTC CACCTGATCG TGGCAACCCA GTGCGGGTGA
GAGGCCCGTT ACGGTGACGG TCGCGTCGGA CGCTCTCCAG GAGTCGGGTT GTTTGAGAAT
GCAGCCCAAA GCGGGTGGTA AACTCCATCT AAGGCTAAAT ACTGACACGA GTCCGATAGA
GGACAAGTAC CGTGAGGGAA TGTTGAAAAG GACTTTGAAG AGAGAGTTCA AGAGTACGTG
AAACCGCACA GAGTCAAACG GGTGGATCCG CAAGGTGTCG CACCGGGGAA TTCAGCGGTG
CACTTTCTCC GGCGAGAGCC ACGACCGGGT TCGCGGGGCG CAGTGCGAGA AGGTGTGCCG
G--CATC-GG GATTTACAGC TCGTCGGCCT CGCCTCGTAT CGTCGCGGAT CCGAGGACGC
GCCGCGCGCC CGGCTCCGGG CCGTCTTCGT GGGTTCGACC TTTCTGGGCA GTGCGCTGAA
ACTGCTCGCG GGCTCGGTGT TGAGGGTCGG TGGCGAATCG GTCGGTACTC CACCCGACCC
GTCTTGAAAC ACGGACCAAG GAGTCTAACA TGTGCGCGAG TCACAGGGAC CCGAATCCCG
AAGGCGCAAT GAAAGTGAAG GCCGCCCTCG GCTGGCCTAG GCAGGATCCC GTCGTGGGCG
CACTGCCGGC CCGTCTCGAT AGCGGCGCGT TG-GGCGGCG CGTTCGTCTA CGAGGCGGAG
CAAGAGCGTA CACGTTGGTA CCCGAAAGAT GGTGAACTAT GCCTGAGCAG GACGAAGTCA
GAGGAAACTC TGATGGAGGT CCGCAGCGAT TCTAGAAAGT CCACTGGTGG AAAAGCTCCA
CGTAAACAAT TGGCCACTAA GGCTGCACGT AAGAGTGCAC CGGCCACTGG TGGTGTGAAG
AAACCACACA GGTACAGGCC CGGAACCGTC GCTCTCCGTG AAATCAGAAG GTACCAGAAG
AGCACCGAGC TTTTGATCAG GAAACTGCCA TTCCAGCGAC TCGTCCGTGA GATCGCCCAG
GACTTCAAGA CTGATCTCCG CTTCCAGAGC TCTGCCGTTA TGGCACTGCA GGAGGCTAGC
GAGGCTTACT TGGTCGGACT CTTCGAGGAC ACTAACTTGT GCGCCATCCA CGCCAAGCGT
TACTCTGTAT TTTATTTTCG GTATTTGATC TGGGTTGGTA GGTACTTCTT TTAGTGTCCT
AATTCGTTTG GAATTATCTC GCCCTGGTGC TTTTTTAGGT GACGATCATT TATATAATGT
GATCGTTACC GCGCATGCGT TTGTAATAAT TTTTTTCTTG GTTATGCCTA TAATGGTTGG
GGGATTTGGT AATTGGTTGG TTCCTTTGAT ATTGGTTTCT CCAGACATGG CTTTCCCGCG
AATGAATAAT ATAAGGTTCT GGCTTTTGCC CCCTGCTTTG TTTTTACTAT TAAGTTCGGC
TTTTGTTGAG AGAGGGGTTG GAACAGGTTG AACTGTATAC CCTCCTCTTT CATCTAACGT
TACCCATAGA GGGGCTTCTG TTGATTATGG GATTTTTTCC TTACACTTAG CGGGTGTTTC
TTCTATTTTA GGAGCTATTA ACTTTTTAGC AACAACGGTA AATATACGAC CTGAAGTTAT

AGAGCTGAAG CGGGTTACAC TATTCGTTTG GTCAGTAGCC ATTACTGCAT TCCTATTGGT
TGTGGCTATG CCGGTCTTAG CTGGGGCTAT CACTATGTTG TTGACGGACC GAAATTTTAA
CACTTCTTTT TTTGATCCTT CTGGTGGGGG GGATCCTATT TTATTCGTCC ATTTATTTT

>Azonnus_minutus
GTGTTATATG CTTGTCTCAA AGATTAAGCC ATGCAGGTCT AAGTACAGGC C---CTAACG
GCGAAACTGC GAATGGCTCA TTAAATCAGT TATGGTTCCT TAGACATACC ACATCCACTT
GGACAACTGT GGAAAATCTA GAGCTAATAC ATGCGACAAA GCTCCGACCT CCGGGGAAGA
GCGCATTTGT TAGCAAAACC AACCCGGCCG GCTGGTGACT CTGAACAACC TTGGGCTGAT
CGCACGGCCT CGCGCCGGCG ACGCATCTTT CAAATGTCTG CCCTATCAAC TTTCGATGGT
ACGTGCTATG CGTACCATGG TTCTCACGGG TGACGGGGAA TCAGGGTTCG ATTCCGGAGA
GGGAGCCTGA GAAACGGCTA CCACATCCAA GGAAGGCAGC AGGCACGCAA ATTACCCAAT
GCCGACACGG CGAGGTAGTG ACGAAAAATA ACAATACGGG TCTCTTTCGA GGCCCCGTAA
TTGGAATGAG TACACTCTAA ATCCTTTAAC GAGGATCTAT TGGAGGGCAA GTCTGGTGCC
AGCAGCCGCG GTAATTCCAG CTCCAATAGC GTATATTAAA GTTGCTGCGT TTAAAAAGCT
CGTAGTTGGA TCTCGGTTCC AGGCCTGCGG TCCGCCTCGA GGCGGCTGCT CGTCCTGCGT
TCGACGTCGT GGTGTCCCTT GGTGCTCTTG ACTGAGTGTC GGCGGCCCGA ACGTTTACTT
TGAAAAAATT AGAGTGCTCA AAGCA--GGC GTATGCCTGA ATAATTCCGC ATGGAATAAT
GGAAAAGGAC CTCGGTTCTA TTTCGTTGGT TTGCGAAGAG GTAATGATTA ATAGGGACAG
CCGGGGGCAT ACGTATTGCG GCGGGAGAGG TGAAATTCGT GGATCGCCGC AAGACGAACG
ACAGCGAAAG CATTTGCCAA GAATGTTCTC ATTAATCAAG AACGAAAGTC AGAGGTTCGA
AGACGATCAG ATACCGTCGT AGTTCTGACC CTAAACGATG CCGACTGTCG ATCCGCCGGA
GTTACTACCA TGACTCGGCG GGAAGCCTCC GGGAAACCAA AGTCTTTGGG TTCCGGGGGG
AGTATGGTTG CAAAACTGAA ACTTAAAGGA ATTGACGGAA GGGCACCACC AGGAGTGGAG
CCTGTGGCTT AATTTGACTC AACACGGGAA ACCTCACCCG GTCTGGACAC TGCAAGGATT
GACAGATTGA GAGCTCTTTC TTGATTCGGT GAGTGGTGGT GCATGGCCGT TCTTAGTTGG
TGGAGCGATT TGTCTGGTCA ATTCCGATAA CGAACGAGAC TCTAGCCTAC TAAATAGTTC
GGGGACCCCC GGCCCCGCAA CTTCTTAGAG GGACAGGTGG CGTTTAGCCA CACGAGATTG
AGCAATAACA GGTCTGTGAT GCCCTTAGAT GTCCGGGGCC GCACATGCGC TACAATGAAC
GGTTCAACGT GCTTCTAGCC TTGCCCGAGA GGGCTGGGAA ATCCGCTGAA CCCCGTTCGT
ACCAGGGATT GGGGCTTGCA ATTGTTCCCC ATGAACGAGG AATTCCCAGT AAGCGCGAGT
CATTAGCTCG CGTTGATTAC GTCCCTGCCC TTTGTACACA CCGCCCGTCG CTACGACCGA
TTGTTCCAGT TAATGAGCGA TATGGATGAG TTGATCGGCT TGC----CGT GATTGGGCTG
AGAAGTTGGA CAAATTATCT GGGGTAGAGG ACGTAAAAGT CGTAACAAGG TATCCATCAG
TAAGCGGAGG AAAAGAAACT AACCAGGATT CCCCTAGTAA CGGCGAGTGA AGCGGGAAGA
GCCCAGCACC GAATCCCCCG GC-TCGAG-- GTCGGCGGGA AATGTGGTGT -ACAGGAAGC
CGATTG--GG CGGCGACGAG CGTCCAAGTC CTCCTGATCG TGGCTGCCCA GAGAGGGTGT
CAGGCCCGTG AGGGCGCCAG T--CGACCGT CCCTTCTTCG GAGTCGGGTT GTTTGGGAAT
GCAGCCCAAA GTGGGTGGTA AACTCCATCT AAGGCTAAAT ACTGGCACGA GTCCGATAGC
GGACAAGTAC CGTGAGGGAA AGTTGAAAAG AACTTTGAAG GGAGAGTTCA ACAGTACGTG
AAACCGCATA GAGGTAAACG GGTGGACCCG CAGTGT-TCG GCCCGGGGAA TTCAGCGGTG
CACTTTCTCC GCCGAGGGCC ACGACCGGCC CGGCGGTCAG AAGCCAGGGA AGGTGACTCG
GGAGGCT-GA GAGTTACAGC CCACCGCGGT ------GACA CGTCGCGGGG CCGAGGAAGC
GCCGCACCTC CGGTCCCGGG AGCTCCCCGT CCGTCCGACC T-----CGCA GTGCGCGGTA
ACCGCGGTTC CTCCCGGAGG GAAGGGTCAG TGGCGAATCG GTCGGTGCTC CACCCGACCC
GTCTTGAAAC ACGGACCAAG GAGTCTAACA TGTGCGCGAG TCACAGGGCT CCGAAACCCA
AGGGCGCAAC GAAAGTGAGG GAGGTCTCCG GCCGTCCCAG GCAGGATCCC GCCCCGGGCG
CACTGCCGGC CTGTCTCGGC GGC------- ---------- --ATCGTCCG CGAGGCGGAG
CAAGAGCGCA CACGTTGGGA CCCGAAAGAT GGTGAACTAT GCCTGAGCAG GACGAAGTCA
TGGGAAACTC TGATGGAGGT CCGTAGCGAT TCTAGAAAGT CCACNGGAGG AAAGGCTCCA

AGGAAACAGT TGGCCACTAA GGCCGCTCGT AAGAGCGCCC CAGCCACTGG NGGTGTGAAG
AAACCACACA GGTACAGGCC AGGAACCGTC GCCCTGAGAG AGATCAGNCG TTACCAGAAG
AGCACCGAGT TGCTCATCAG GAAACTGCCC TTCCAGCGTC TGGTTCGTGA NATTGCCCAG
GACTTCAAGA CTGACCTCCG ATTCCAGAGC TCTGCCGTTA TGGCACTGCA GGAGGCTAGC
GAAGCCTACC TTGTTGGTCT GTTCGAGGAC ACCAACTTGT GCGCCATCCA CGCCAAGCGT
TAGGTTGTAT TTTGTTTTAG GGCTGTGGTC CGGGTTAGTC GGGTTGGTGT ACAGGATGAT
GATGCGAACT GAGCTAATGC ACCCTGGTGC TTTTTATGGG ---GAATCTG TTTATAATGT
GTTAGTAACA TCCCATGGTT TACTAATAAT TTTTTTTATG GTTATGCCTT TAATAATTGG
GTTTTTTGGT AACTGGGCTG TCCCTTTACT TTTGGCGGCC CCAGATATAG TATTTGCACG
GTTAAATAAT TTAAGATTTT GACTTTTACC AGCAGCCACT GTTTTGCTTT TAATATCAAA
CGAAGTAGAA GAAGGTGTAG GTACTGGGTG GACTCTCTAC CCTCCTTTAT CTGCCTGGTT
AGGGCACCCT GCACCTGCTA TAGAGTTTAT GATTTTGGGG CTACATATTG CTGGTTTATC
TTCAATTTTT GCGAGAATTA ATTTTGTTAC AACAGGCGTA AATATGCGCC CTGAGGGTAT
TGCTCCCCAG CGAACTACTT TATTTGTAGT TTCTGTAATT ATTACTTCTT TTTTGCTGGT
TGTTGCTATA CCGGTGTTAG CCGCTGGATT GACTATACTT CTTACTGATC GAAATTTTAA
TACTTCTTTT TTTGATCCGG TAGGGGGTGG TGATCCGGTA TTGTTTATTC ATTTGTTTT

>Gastrochaena_cuneiformis
GTGTCATATG CTTGTCTCAA AGATTAAGCC ATGCATGTCT AAGTACACGC T--TTAAAAA
GTGAAACTGC GAATGGCTCA TTAAATCAGT TATGGTTCCT TAGATCGTAC AATCCTACTT
GGATAACTGT GGTAATTCTA GAGCTAATAC ATGTTGACAA GCTCCGACCT TCGGGGAAGA
GCGCTTTTGT TAGCAAGACC AATCCGATCG GTTGGTGACT CTGGACAACT TTGTGCTGAT
CGCAGGGCCA CGAGCCGGCG ACGTATCTTT CAAATGTCTG CCCTATCAAC TTTCGATGGT
ACGCGATATG CCTACCATGG TGATAACGGG TAACGGGGAA TCAGGGTTCG ATTCCGGAGA
GGGAGCATGA GAAACGGCTA CCACATCCAA GGAAGGCAGC AGGCGCGTAA ATTACCCAAT
CCTGACACAG GGAGGTAGTG ACGAAAAATA ACAATACGGG ACTCTTTCGA GGCCCCGTAA
TTGGAATGAG TACACTTTAA ATCCTTGAAC GAGGATCCAT TGGAGGGCAA GTCTGGTGCC
AGCAGCCGCG GTAATTCCAG CTCCAATAGC GTATATTAAA GTTGTTGCAG TTAAAAAGCT
CGTAGTTGGA TCTCGGGTAC GGGCTTGCGG TCCGCCTCGA GGCGGCTGCT TGTCTCGGCC
TACCTCATGG GAAGCTCCTT GGTGCTCTTG ACCGAGTGTC GGCAGCCAGG ACGTTTACTT
TGAAAAAATT AGAGTGTTTA AAGCA--GGC GTTTGCCTAG ATAATGGTGC ATGGAATAAT
GGAATAGGAC CTCGGTTCTA TTATGTTGGT TTTAGGAGAG GTAATGGTTA AGAGGGACGG
CCGGGGGCAT CCGTATTGCG GGGTTAGAGG TGAAATTCTT GGATCCTCGC AAGACGAACA
ACAGCGAAAG CATTTGCCAA GAATGTTTTC TTTAATCAAG AACGAAAGTC AGAGGTTCGA
AGACGATCAG ATACCGTCGT AGTTCTGACC ATAAACGATG CCAACTGGCA ATCCGCCGCA
GTTGCTTCAA TGACTCGGCG AGCAACCCCC GGGAAACCAA AGTTTCTGGG TTCCGGGGGG
AGTATGGTTG CAAAACTGAA ACTTAAAGGA ATTGACGGAA GGGCACCACC AGGAGTGGAG
CCTGTGGCTT AATTTGACTC AACACGGGAA AACTCACCCG GCCCGGACAC TGTAAGGATT
GACAGATTGA GAGCTCTTTC TTGATTCGGT GGGTGGTGGT GCATGGCCGT TCTTAGTTGG
TGGAGCGATT TGTCTGGTTA ATTCCGATAA CGAACGAGAC TCTAGCCTGC TAAATAGTTC
GAGGATCCTT GTCCTCGCAA CTTCTTAGAG GGACAAGTGG CGCTTAGCCA CACGAGATTG
AGCAATAACA GGTCTGTGAT GCCCTTAGAT GTTCGGGGCC GCACACGCGC TACACTGAAT
GAATCAACGT GCGTCATGCC TAGCCCGAGA GGGCCTGGAA ACCCGTTGAA CCTCATTCGT
GCTAGGGATT GGGGATTGTA ATTCTTCCCC ATGAACGAGG AATTCCCAGT AAGCGCGAGT
CATCAGCTCG CGTTGATTAC GTCCCTGCCC TTTGTACACA CCGCCCGTCG CTACTACCGA
TCGCTCTAGT TAATGAGCGC GTCGGATCGG TTAGGCG--- -CCGCGGTGC TCTAGTGCTG
AGAAGATGCG CAAATTATCT GGAGTAGAGG AAGTAAAAGT CGTAACAAGG TATTCATAAA
TAAGCGGAGG AAAAGAAACT AACAAGGATT CCCTCAGTAA CGGCGAGTGA AGCGGGAAGA
GCCCAGCACC GAATCCCCCA GCGTCT---C GCTGTCGGGA AATGTGGTGT -ACAGGAAGC
CGATTGCCGC CCTGTCGCGG CGCCCAAGTC CTCCTGATCG TGGCTACCCA AAGCGGGTGT

CAGGCCCGTT CCGGCGTCGG ACGGGGTGGC --CTTCCTAG GAGTCGGGTT GTTTGGGAAT
GCAGCCCAAA GTGGGTGGTA AACTCCATCT AAGGCTAAAT ACGGGCACGA GTCCGATAGT
AGACAAGTAC CGTGAGGGAA AGTTGAAAAG AACTTTGAAG AGAGAGTTCA AGAGTACGTG
AAACCGCATA GAGTCAAACG GGTGGATCCG CAAA--GTCG ACCCGGGGAA TTCAGCGGTG
CACTTTCTTC GTTGAGCGCC ACGACCGGTT CTGCGGTCAG AAGCGCGGGA AGGTGACCCG
T----CTGGG AG-TTATAGC CCGCCGATGC ------GACG CGCCGCGGGA CCGAGGACGC
-CTGAGTTCC CGGCCACGGG TCGTCTTCGC ACGTTCGACC T----TGGCA GTGTGTCGTA
ACCGCGTGCG GACTCCGTCG CTAGGGTCGG TGGCGAATCG GTCGGTCCTC CACCCGACCC
GTCTTGAAAC ACGGACCAAG GAGTCTAACA TGTGCGCGAG TCACAGGGTC TCGAAACCCG
AGGGCGCAAT GAAAGTGAAG GCCGCCTACG GCTGGCCTAG GCAGGATCCC GTGCCGGGCG
CACTGCCGGC CCGTCTCGGT AGC------- ---------- --ATTGCCTA CGAGGCGGAG
CTAGAGCGCA CACGTTGGGA CCCGAAAGAT GGTGAACTAT GCCTGAGTAG GACGAAGTCA
GAGGAAACTC TGATGGAGGT CCGTAGCGAT TCAAGAAAGT CCACCGGAGG AAAGGCTCCG
AGAAAACAGC TGGCCACCAA GGCTGCACGT AAGAGTGCAC CGGCGACAGG AGGAGTAAAG
AAACCACACA GATACAGGCC CGGAACTGTC GCTCTCCGTG AGATCAGACG TTACCAGAAG
AGCACAGAAC TTCTGATCAG GAAGCTTCCA TTCCAGCGTC TCGTTCGTGA AATCGCTCAG
GACTTCAAGA CTGATCTCCG ATTCCAGAGC TCCGCCGTCA TGGCTCTGCA GGAAGCTAGC
GAGGCTTACT TGGTCGGTCT CTTTGAGGAC ACCAACCTGT GCGCCATCCA CGCTAAGCGT
?????????? ?????????? ?????????? ?????????? ?????????? ??????????
?????????? ?????????? ?????????? ?????????? ?????????? ??????????
?????????? ?????????? ?????????? ?????????? ?????????? ??????????
?????????? ?????????? ?????????? ?????????? ?????????? ??????????
?????????? ?????????? ?????????? ?????????? ?????????? ??????????
?????????? ?????????? ?????????? ?????????? ?????????? ??????????
?????????? ?????????? ?????????? ?????????? ?????????? ??????????
?????????? ?????????? ?????????? ?????????? ?????????? ??????????
?????????? ?????????? ?????????? ?????????? ?????????? ??????????
?????????? ?????????? ?????????? ?????????? ?????????? ??????????
?????????? ?????????? ?????????? ?????????? ?????????? ?????????

>Irus_mitis
GTGTCATATG CTTGTCTCAA AGATTAAGCC ATGCATGTCT AAGTACACGC --CTTGAACG
GCGAAACTGC GAATGGCTCA TTAAATCAGT TATGGTTCCT TAGATCGTAC AATCCTACTT
GGATAACTGT GGCAATTCTA GAGCTAATAC ATGCAACACA GCTCCGACCT TGCGGGAAGA
GCGCTTTTGT TAGCAAAACC AATCCGGGCG GCTGGTGACT CTGAACAACT TTGTGCCGAT
CGTATGCCCT AGCGGCGACG ACGCGTCTTT CAAATGTCTG CCCTATCAAC TGACGATGGT
ACGTGCTATG CCTACCATGG TGATAACGGG TAACGGGGAA TCAGGGTTCG ATTCCGGAGA
GGGAGCATGA GATACGGCTA CCACATCCAA GGAAGGCAGC AGGCGCGCAA ATTACCCACT
CCCGACACGG GGAGGTAGTG ACGAAAAATA ACAATACGGG ACTCTTTCGA GGCCCCGTAA
TTGGAATGAG TACACTTTAA ATCCTTTAAC GAGGATCCAT TGGAGGGCAA GTCTGGTGCC
AGCAGCCGCG GTAATTCCAG CTCCAATAGC GTATATTAAA GTTGCTGCAG TTAAAAAGCT
CGTAGTTGGA TCTCGGGTGC AGGCTTGCGG TCCGTCTCGC GGCGGCTGCT CGTCCTGGCC
TCCTCGC--C GGCGTCCCTT GGTGCTCTTG ACTGAGTGTC GGCGGCCGGA ACGTTTACTT
TGAAGAAATT AGAGTGCTCA AAGCA--GGC CTTGGCCTGC ATAATGGTGC ATGGAATAAT
GGAATAGGAC CTCGGTTCTA TTTTGTTGGT TTTCGGAGAG GTAATGATTA ATAGGGACTG
ACGGGGGCAT TCGTATTGCG GCGCTAGAGG TGAAATTCTT AGACCGTCGC AAGACGAACT
ACAGCGAAAG CATTTGCCAA GCATGTTTTC ATTAATCAAG AACGAAAGTC AGAGGTTCGA
AGACGATCAG ATACCGTCGT AGTTCTGACC ATAAACGATG CCAACTGTCG ATCCGCCGGA
GTTGCTTCAA TGACTCGGCG GGCAGCCTCC GGGAAACCAA AGTTTCTGGG TTCCGGGGGG
AGTATGGTTG CAAAGCTGAA ACTTAAAGGA ATTGACGGAA GGGCACCACC AGGAGTGGAG

CCTGTGGCTT AATTTGACTC AACACGGGGA ACCTCACCCG GCCCGGACAC TGCAAGGATT
GACAGATTGA GAGCTCTTTC TTGATTCGGT GGGTGGTGGT GCATGGCCGT TCTTAGTTGG
TGGAGCGATT TGTCTGGTTA ATTCCGATAA CGAACGAGAC TCTAGCCTAC TAAATAGTTC
GGGGATCCTC GTCCCCGCAA CTTCTTAGAG GGACAAGTGG CGCTTAGCCA CACGAGATTG
AGCAATAACA GGTCTGTGAT GCCCTTAGAT GTTCGGGGCC GCACATGCGC TACACTGAAT
GGATCAGCGT GCGTCTTGCC TGACCCGAGA GGGTTGGGAA ACCCGTTGAA CCCCATTCGT
GCTAGGGATT GGGGCTTGCA ATTGTTCCCC ATGAACGAGG AATTCCCAGT AAGCGCGAGT
CATCAGCTCG CGTTGATTAC GTCCCTGCCC TTTGTACACA CCGCCCGTCG CTACTACCGA
TCGCTCCAGT TAATGAGCTC TTCGGATTGG TTAGGCC-GC CTCGGGTGGC TCTCGTGCCG
AGAAGATGCG CAAATTGACC GGAGTAGAGG AAGTAAAAGT CGTAACAAGG TATCCATCAG
TAAGCGGAGG AAAAGAAACT AACCAGGATT CCCCCAGTAA CGGCGAGTGA AGCGGGAAGA
GCCCAGCACC GAATCTCCCG GC-CTTGTCG GGCGGCGAGA AATGTGGTGT -ACAGGCGGC
CGATTGTCGG CG---TCGAG CGCCCAAGTC CTCCTGATTG TGGCTACCCA GAGCGGGTGT
CAGGCCC-TT TCGGCGCGAG ACCCGGCGGC --CCTCCTCG GAGTCGGGTT GTTTGGGAAT
GCAGCCCAAA GCGGGTGGTA AACTCCACCT AAGGCTAAAT ACTGGCACGA GTCCGATAGC
GGACAAGTAC CGTGAGGGAA AGTTGAAAAG AACTTTGAAG AGAGAGTTCA AGAGTACGTG
AAACCGCACA GAGTCAAACG GGTGGATCCG CAAA--GTCG ACCCGGGGAA TTCAGCGGTG
CACTTTCTCC GTCGAGTGCC ACGACCGATT CAGCGGTGAC GAGCGCGGCA AGGTGGCTCG
GCGCTTC-GA GTGTTATAGG CCGCCGCGGT ------GACA CGTCGCGGGA TCGAGGACGC
GCCGCGTCAG CGGCCTCGGG CCGTTCTGGG GAGACCGACT T----TCGCA GGGCGCCGAG
ACCGCTCCCC GGTCCGTACG CTCGGGTCAG TGGCGAATCG GTCGGTCCTC CNCCCGACCC
GTCTTGAAAC NCGGACCNAG GAGTNTAACN TGTGCGCGAG TCATGGGGTC CCGAAACCCA
AAGGCGCAAT GAAAGTGAAG GTCTCCTCCG GAGCNCCCNG GCAGGATCCC G-CGTGGGCG
CNCTGCCGGC CCNTCTNGGC GGC------- ---------- --ATNGTCCG CGAGGCGGAG
CAAGAGCGCA CACGTTGGGA CCCGAAAGAT GGTGAACTAT GTCTGAGTAG GGCGAAGTCA
GAG------- ---------- ---------- --TAGAAAAT CTACTGGAGG AAAAGCTCCT
CGCAAACAGC TGGCCACCAA GGCCGCTCGT AAGAGTGCAC CAGCCACTGG TGGTGTGAAA
AAGCCACACA GATACAGGCC AGGAACCGTC GCTCTCCGTG AAATCAGGCG TTACCAGAAG
AGCACCGAGC TTCTCATCCG AAAGCTTCCA TTCCAGCGTC TCGTACGTGA AATTGCTCAG
GACTTCAAGA CCGATCTCCG ATTCCAGAGC TCAGCTGTCT TGGCTCTCCA GGAAGCCAGC
GAAGCCTACT TGGTCGGTCT GTTTGAGGAC ACCAACTTGT GCGCCATCCA CGCCAAGCGT
GACTCTTTAT TTTGTGTTTT CTATTTGAGC CGGTTTAATG GGGACGGCTT TTAGGGTTAT
TATTCGGATG GAGCTTGCTA TGCCTGGAAA AATGTTGGAT GATGGTCAGT TATATAATGT
TATTGTTACG GCTCATGGAC TAGTGATAAT TTTTTTTCTA GTAATGCCGA TGATGATTGG
TGGTTTTGGT AATTGGCTGG TTCCTCTAAT GTTAGCTGTT CCAGATATAT CTTTACCTCG
TATAAACAAC TTAAGTTTTT GGTTGCTCCC TGTGTCAATG TTGCTATTGT TGGGATCAGC
TTACGTGGAT GGTGGTGCTG GGACAGGTTG AACTATCTAT CCTCCATTGT CTAGTGTTCT
TTCTCATTCT GGTTGTGCTA TGGATTATGT AATTTTTTCT CTTCATATTG GTGGGGCTTC
TTCTATTATG TCATCTATTA ATTTTGTAAC TACAGCCTTG TTTATACGAT GTGGGGTTAT
AAGACTTCTT CGGACTAGAA TGTTTGTATG GTGTGTTTGG GTTACCGGGT TTCTTCTGAT
TGTAGCTATA CCAGTTTTGG CGGGTGCCTT GACTATACTA TTAACTGATC GTAATTTTAA
TACATCATTT TTTGACCCAG TAGGTTTAGG TGATCCTATT TTATTTGTTC ATTTATTTT

>Meropesta_nicobarica
GNGTCATATG CTTGTCTCAA AGATTAAGCC ATGCATGTCT AAGTACACGC ---CTCAACG
GCGAGACTGC GAATGGCTCA TTAAATCAGT TATGGTTCCT TAGATCGTAC AATCCTACTT
GGATAACTGT GGCAATTCTA GAGCTAATAC ATGCAACACA GCTCCGACCC GCGGGGAAGA
GCGCTTTTGT TAGCAAAACC AATCCGGGCG GCTGGTGACT CCGAACAACT TTGTGCCGAT
CGCAGGCCCT AGCGGCGGCG ACGCATCTTT CAAGTGTCTG CCCTATCAAC TTTCGATGGT
ACGTGCTATG CCTACCATGG TGATAACGGG TAACGGGGAA TCAGGGTTCG ATTCCGGAGA

GGGAGCATGA GATACGGCTA CCACATCCAA GGAAGGCAGC AGGCGCGCAA ATTACCCACT
CCCGACACGG GGAGGTAGTG ACGAAAAATA ACAATACGGG ACTCTTTCGA GGCCTCGTAA
TTGGAATGAG TACACTTTAA ATCCTTTAAC GAGGATCCAT TGGAGGGCAA GTCTGGTGCC
AGCAGCCGCG GTAATTCCAG CTCCAATAGC GTATATTAAA GTTGCTGCAG TTAAAAAGCT
CGTAGTTGGA TCTCGGGTGC AGGCCTGCGG TCCGCCTCGC GGCGGCTGCT CGTCCTGACC
TCC--ACGCC GGTGCGCCCT GGTGCTCTTG ACTGAGTGTC GCCGGCCGGA ACGTTTACTT
TGAAGAAATT AGAGTGCTCA AAGCA--GGC CATCGCCTGA ATAATGGTGC ATGGAATAAT
GGAATAGGAC CTCGGTTCTA TTTTGCTGGT TTTCGGAGAG GTAATGATTA ATAGGGACAG
ACGGGGGCAT TCGTATTGCG GCGCTAGAGG TGAAATTCTT AGACCGTCGC AAGACGAACT
ACAGCGATAG CATTTGCCAA GCATGCTTTC ATTAATCAAG AACGAAAGTC AGAGGTTCGA
AGACGATCAG ATACCGTCGT AGTTCTGACC ATAAACGATG CCAACTGTCG ATCCGCCGGA
GTTGCTTCAA TGACTCGGCG GGCAGACTCC GGGAAACCAA AGTTTTTGGG TTCCGGGGGG
AGTATGGTTG CAAAGCTGAA ACTTAAAGGA ATTGACGGAA GGGCACCACC AGGAGTGGAG
CCTGTGGCTT AATTTGACTC AACACGGGGA ACCTCACCCG GCCCGGACAC CGTTAGGATT
GACAGATTGA GAGCTCTTTC TTGATTCGGT GGGTGGTGGT GCATGGCCGT TCTTAGTTGG
TGGAGCGATT TGTCTGGTTA ATTCCGATAA CGAACGAGAC TCTAGCCTGC TAAATAGCTC
GGGGATCCTT GTCCCCGCAG CTTCTTAGAG GGACAGGTGG CGCTTAGCCA CACGAGATTG
AGCAATAACA GGTCTGTGAT GCCCTTAGAT GTTCGGGGCC GCACATGCGC TACAATGAAT
GGATCAGCGT GCGTCTCGCC TGGTCCGAAA GGATCGGGAA ACCCGTTGAA CCCCATTCGT
ACTAGGGATT GGGGCTTGCA ATTGTTCCCC ATGAACGAGG AATTCCCAGT AGGCGCGAGT
CATCAGCTCG CGTCGATTAC GTCCCTGCCC TTTGTACACA CCGCCCGTCG CTACTACCGA
TCGCTCCAGT TAATGAGCTC TTCGGATTGG ATAGGCCTGC TTCGGTGGGC CATT-GGCCG
AGAAGATGCG CAAATTGACC GGGGTAGAGG TCGTAAAAGT CGTAACAAGG TATCCATCAG
TAAGCGGAGG AAAAGAAACT AACAAGGATT CCCCCAGTAA CGGCGAGTGA AGCGGGAAGA
GCCCAGCACC GAATCTCCCG GC--CAAACG GGCGGCGAGA AATGTGGTGT -ATAGGTGGC
CGATTGTCGT CGCG----AG CGCTCAAGTC CTCCTGATCG TGGCTGCCCA GAGCGGGTGT
CAGGCCCTTG GCGGCGCGAG CTTCGGCGGC --CCTCCTCT GAGTCGGGTT GTTTGGGAAT
GCAGCCCAAA GCGGGTGGTA AACTCCACCT AAGGCTAAAT ACAGGCACGA GTCCGATAGC
GGACAAGTAC CGTGAGGGAA AGTTGAAAAG CACTTTGAAG AGAGAGTTCA AGAGTACGTG
AAACCGCATA GAGGCAAACG GGTGGATCCG CAGA--GTCG ACCCGGGGAA TTCAGCGGTG
CACTTTCTCC GTCGAGTGCC ACGACCGGTT CGGCGGTCAG GAGCGAGGCA AGGTAGCTCG
C---GTC-GA GTGTTAAGGC CT-TCGAGGT ------GACA CGCTGCTGGA CCGAGGACGC
GCCGCGTCCG TGGCCTCGGG CCGTGCTGGA GCGTTCGACC G----TCGCA GTGCGCCGCA
ACCGCGCTTC GTCCCGTACG TCAGGGTCTG TGGCGAATCG GTCGGTCCTC CACCCGACCC
GTCTTGAAAC ACGGACCAAG GAGTCCAACA TGTGCGCGAG TCATGGGGCT CCGAAACCCA
AAGGCGCAAT GAAAGTGAAG GCGGTCTCCG -ACTGCCCAG GCAGGATCCC GTCGTGGGCG
CACTGCCGGC CCGTCTCGGC CGC------- ---------- --ATCGTCGG CGAGGCGGAG
CAAGAGCGCA CACGTTGGGA CCCGAAAGAT GGTGAACTAT GTCTGAGCAG GACGAAGTCA
GAGGAAACTC TGATGGAGGT CCGCAGCGAT TCTAGAAAAT CTACCGGAGG AAAGGCTCCA
AGGAAGCAGC TGGCTACCAA GGCCGCTCGT AAGAGTGCCC CAGCCACCGG TGGTGTGAAG
AAACCACACA GATACAGGCC AGGAACCGTC GCCCTTCGTG AAATCAGACG TTACCAGAAG
AGCACTGAAC TTCTCATCAG GAAACTGCCG TTCCAGCGTC TGGTCCGTGA AATTGCTCAG
GATTTCAAGA CTGACCTCCG ATTCCAGAGC TCCGCCGTGT TGGCTCTCCA GGAGGCTAGC
GAAGCCTACC TTGTTGGACT GTTCGAGGAC ACCAACTTGT GCGCTATCCA CGCCAAGCGT
?????????? ?????????? ?????????? ?????????? ?????????? ??????????
?????????? ?????????? ?????????? ?????????? ?????????? ??????????
?????????? ?????????? ?????????? ?????????? ?????????? ??????????
?????????? ?????????? ?????????? ?????????? ?????????? ??????????
?????????? ?????????? ?????????? ?????????? ?????????? ??????????
?????????? ?????????? ?????????? ?????????? ?????????? ??????????

?????????? ?????????? ?????????? ?????????? ?????????? ??????????
?????????? ?????????? ?????????? ?????????? ?????????? ??????????
?????????? ?????????? ?????????? ?????????? ?????????? ??????????
?????????? ?????????? ?????????? ?????????? ?????????? ??????????
?????????? ?????????? ?????????? ?????????? ?????????? ?????????

>Solen_strictus
--GTCATATG CTTGTCTCAA AGATTAAGCC ATGCATGTCT AAGTACAGAC --CTTCAATG
GTGAAACTGC GAAAGGCTCA TTAAATCAGT TATGGTTCCT TAGATCGTAC AGTCCTACTT
GGATAACTGT GGCAATTCTA GAGCTAATAC ATGCTACACA GCTCCGACCC TCGGGGAAGA
GCGCTTTTGT TAGCAAAACC AATCCGGCCG GCTGGTGACT CTGAACAACT TTGTGCTGAT
CGCACGGCCT CGCGCCGGCG ACGTATCTTT CAAATGTCTG CCCTATCAAC TTTCGATGGT
ACGTGCTATG CCTACCATGG TTGTAACGGG TAACGGGGAA TCAGGGTTCG ATTCCGGAGA
GGGAGCATGA GAAACGGCTA CCACATCCAA GGAAGGCAGC AGGCGCGCAA ATTACCCACT
CCTGACACGG GGAGGTAGTG ACGAAAAATA ACAATACGGG ACTCTTTCGA GGCCCCGTAA
TTGGAATGAG TACACTTTAA ACCTTTTAAC GAGGATCCAT TGGAGGGCAA GTCTGGTGCC
AGCAGCCGCG GTAATTCCAG CTCCAATAGC GTATATTAAA GTTGTTGCAG TTAAAAAGCT
CGTAGTTGGA TCTCGGGTCC AGGCCTGCGG TCCGCCTCGC GGCGGCTGCT CGTCCTGACC
TCCCAGC--C GGTGTCCCTT GGTGCTCTTG ANTGAGTGTC GGCGGCCGGA ACGTTTACTT
TGAAAAAATT AGAGTGTTCA AAGCA--GGC GTGTGCCTGT ATAATGGTGC ATGGAATGAT
GGAATAGGAC CTCGGTTCTA TTTTGTTGGT TTTCGGAGAG GTAATGATTA AGAGGGACAG
ACGGGGGCAT TCGTATTGCG GTGTTAGAGG TGAAATTCTT GGATCGCCGC AAGACGAACT
ACAGCGAAAG CATTTGCCAA GCATGTTTTC ATTAATCAAG AACGAAAGTC AGAGGTTCGA
AGACGATCAG ATACCGTCGT AGTTCTGACC ATAAACGATG CCGACTGGCG ATCCGCCGGA
GTTGCTTCAA TGACTCGGCG GGCAGCCCCC GGGAAACCTA AGTTTTTGGG TTCCGGGGGG
AGTATGGTTG CAAAGCTGAA ACTTAAAGGA ATTGACGGAA GGGCACCACC AGGAGTGGAG
CCTGTGGCTT AATTTGACTC AACACGGGGA AACTCACCCG GCCCGGACAC TGCAAGGATT
GACAGATTGA GAGCTCTTTC TTGATTCGGT GGGTGGTGGT GCATGGCCGT TCTTAGTTGG
TGGAGCGATT TGTCTGGTTA ATTCCGATAA CGAACGAGAC TCTAGCCTGC TAAATAGTTC
GAGGATGGCT GTCCTCGGAA CTTCTTAGAG GGACAAGTGG CGTTTAGCCA CACGAGATTG
AGCAATAACA GGTCTGTGAT GCCCTTAGAT GTTCGGGGCC GCACACGCGC TACACTGAAT
GCATCAGCGT GCGTCTCTCC TTGCCCGAGA GGGCTGGGAA ACCCGTTGAA CCGCATTCGT
GCTAGGGATT GGGGCTTGCA ATTCTTCCCC ATGAACGAGG AATTCCCAGT AAGCGCGAGT
CATCAGCTCG CGTTGATTAC GTCCCTGCCC TTTGTACACA CCGCCCGTCG CTACTACCGA
TCGTTCCCGT TAATGAGCGC CTCGGATTGG TTGAGCGGGT TTCGGCCTGC TCTCGTGCCG
AGAAGATGTG CAAATTATCA GGAATAGAGG AAGTAAAAGT CGTAACAAGG TATCCATAAA
TAAGCGGAGG AAAAGAAACT AACAAGGATT CCCCTAGTAA CGGCGAGTGA AGCGGGAAGA
GCCCAGCACC GAATCCCCCT GCGTCT---C GCAGGCGGGA AATGTGGTGT -ATAGGAAGC
CGATTGG-GC CGTGTCCGGA CGCCCAAGTC CTCCTGATCG TGGCTACCCA CAGCGGGTGT
CAGGCCCGTT CCGGCGACTG GCGCGGTG-- CCCTTCCTTG GAGTCGGGTT GTTTGGGAAT
GCAGCCCAAA GCGGGTGGTA AACTCCATCT AAGGCTAAAT ACGGTCGCGA GTCCGATAGC
GGACAAGTAC CGTGAGGGAA AGTTGAAAAG AACTTTGAAG AGAGAGTTCA AGAGTACGTG
AAACCGCATA GAGGCAAACG GGTGGATCCG CA--GTGTCT GCCCGGGGAA TTCAGCGGTG
CACTTTCTCC GTCGAGAGCC ACGACCGATT CCGCGGTCAT GAGCGCGGGA AGGTAGCTCG
CCCCTCCTGA GTGTTATAGC CCGCCGCGG- TGGACACGCT GGTCTTGGGA TCGAGGACGC
GCCGTGCCCC TCGCCTCGGG CCGTCCTGGT GAGTTCGAAC T-----TGCA GTGCGCCGTA
ACCGCTCGCC GGTTCGTCGG CTCGGGTCGG TGGCGACTCG GTCGGTACTC CACCCGACCC
GTCTTGAAAC ACGGACCAAG GAGTCTAACA TGTGCGCTAG TCATGGAGCC CCAAAGCTCA
AAGGCGCAAT GAAAGTGAAG GCAGCCTCCG GTTTGCCTAG GCAGGATCCC GCCTCGGGCG
CACTGCCGGC CCGTCTCGAC AGC------- ---------- --ATCGTCTG CGAGGCGGAG

CAAGAGCGCA CACGTTGGGA CCCGAAAGAT GGTGAACTAT GCCTGAGTAG GACGAAGTCA
GGGGAAACTC TGATGGAGGT CCGCAGCGAT TCTAGAAAGT CAACTGGAGG CAAGGCCCCA
CGTAAACAAC TGGCCACCAA GGCCGCACGT AAAAGTGCAC CAGCCACCGG TGGAGTGAAG
AAACCACACA GGTACAGGCC AGGTACCGTC GCTCTTCGTG AGATCAGGAG ATACCAGAAG
AGCACAGAAC TGCTCATCAG GAAACTGCCC TTCCAGCGTC TTGTACGTGA AATCGCCCAG
GACTTCAAGA CCGACCTCCG ATTCCAGAGC TCCGCCGTCA TGGCTCTGCA GGAGGCCAGC
GAAGCCTACT TGGTTGGTCT TTTCGAAGAC ACCAACTTGT GCGCCATCCA CGCCAAGCGT
---------- ---------- ---------- -----TGGTT GGTACCAGGT TAAGAATTTT
AATTCGGTTG GAGTTAGCTC GACCAGGGTC GTATTTAGGG GACGGTCACT TATATAATGT
GATCGTAACT GCTCACGCTT TTATTATGAT TTTTTTTCTT GTGATACCTA TGATGGTTGG
TGGGTTTGGG AATTGGTTAG TTCCTTTAAT GTTGACGTCT CCAGATATGT GTTTTCCTCG
AATAAATAAC ATGAGTTTTT GATTGTTGCC TCCTGCATTG TTTATGTTGC TGTTTTCTGG
GTTGGCCGGG ACGGGCGTTG GGGCAGGTTG GACTATTTAC CCCCCTCTTT CGGGTAACTT
AGCGCATGGT GATCAGTCTA TGGATTTTGC AATTTTTTCG ATGCATTTAG CCGGTGTTTC
CTCAATTCTC GGTGCTATTA ATTTTGTAAC TACAATAATT AATATGCGTC CTGGAATTAT
GGAGTTGAAA CGTGTTCCGT TATTTGTGTG GTCCGTGGCA ATTACTGCCT TCTTATTAAT
TATTGCTATG CCAGTACTAG CCGGGGCTTT AACCATGCTA TTGACTGATC GGCATTTTAA
CACCTCTTTT TTCGATCCGG GTGGTGGTGG TGATTCGATT TTGTTTGTAC ATCTG----

>Solemya_velum
---TCATATG CTTGTCTCAA AGATTAAGCC ATGCATGTCT AAGTGCACAC --TTTCAATA
GTGAAACCGC GAATGGCTCA TTAAATCAGT TGATGTTTAT TAGATCGTAC AATCCTACTT
GGATAACTGT GGTAATTCTA GAGCTAATAC ATGCAACCAA GCTCCGACCT CGAGGGAAGA
GCGCTTTTAT TAGCAAAACC AATCGGTTCG GGTGGTGACT CTGGATAACT TTGTGCTGAT
CGCACGGCCA CGAGCCAGCG ACGTATCTTT CAAATGTCTG CCCTATCAAC TGTCGATGGT
AGGAGATGTG CCTACCATGG TCGTAACGGG TAGCGGGGAA TCAGGGTTCG ATTCCGGAGA
GGGAGCATGA GAAACGGCTA CCACATCCAA GGAAGGCAGC AGGCGCGCAA ATTACCCACT
CCTGGCACGG GGAGGTAGTG ACGAAAAATA ACAATACGGG ACTCGTTCGA GGCCCCGTAA
TTGGAATGAG TACACTCTAA ATCCTTTAAC GAGGATCTAT TGGAGGGCAA GTCTGGTGCC
AGCAGCCGCG GTAATTCCAG CTCCAATAGC GTATATTAAA GTTGTTGCAG TTAAAAAGCT
CGTAGTTGGA TCTCGGGTTT GGGCTGGCGG TCCGCCTAGC GGCGGCTGCC CGTCCCGA--
-CCTACCTGC CGGCACCCTT GATGCTCTTG ACTGAGTGTC GGCGACCGGA ACGTTTACTT
TGAAAAAATT AGAGTGTTCA AAGCA--GGC CTATGCCTGA ATAATGGTGC ATGGAATAAT
GGAATAGGAC CTCGGTTCTA TTTTGTTGGT TTTCGGAGAG GTAATGATTA AGAGGGACAG
ACGGGGGCAT TCGTATTACG GTGTTAGAGG TGAAATTCTT GGATCGCCGT AAGACGAACT
ACTGCGAAAG CATTTGCCAA GAATGTTTTC ATTAATCAAG AACGAAAGTC AGAGGTTCGA
AGACGATCAG ATACCGTCGT AGTTCTGACC ATAAACGATG CCGACTAGCG ATCCGCCGTA
GTTGCTTCAA TGACTCGGCG GGCAGCTTCC GGGAAACCAA AGTCTTTGGG TTCCGGGGGA
AGTATGGTTG CAAAGCTGAA ACTTAAAGGA ATTGACGGAA GGGCACCACC AGGAGTGGAG
CCTGCGGCTT AATTTGACTC AACACGGGAA AACTCACCCG GCCCGGACAC TGTAAGGATT
GACAGATTGA GAGCTCTTTC TTGATTCGGT GGGTGGTGGT GCATGGCCGT TCTTAGTTGG
TGGAGCGATT TGTCTGGTTA ATTCCGATAA CGAACGAGAC TCTAGCCTAC TAAATAGTTC
GCCGATTCGA ATAGTCGGAA CTTCTTAGAG GGACAAGTGG CTTYTAGCCA CACGAGATTG
AGCAATAACA GGTCTGTGAT GCCCTTAGAT GTTCGGGGCC GCACGCGCGC TACACTGAAG
GAATCAACGT GCATTTGCCC TTGCCCGGAA GGGTTGGGTA ACCCGTTGAA CCTCCTTCGT
GCTAGGGATT GGGGCTTGTA ATTATTCCCC ATGAACGAGG AATTCCCAGT AAGCGCGAGT
CATAAGCTCG CGTTGATTAC GTCCCTGCCC TTTGTACACA CCGCCCGTCG CTACTACCGA
TTGAGCGGTT TAGTGACATC CTCGGACTGT T-AGACGGG- ---GGCAACC TCGTTGGACG
GGAAAACGAT GGAACTTGAT CGCTTAGAGG AAGTAAAAGT CGTAACAAGG TTTCCATCAC
TAAGCGGAGG AAAAGAAACT AACAAGGATT CCCTCAGTAA CGGCGAGTGA AGCGGGAAAA

GCCCAGCACC GAATCCCGCA GCTCATG--- GCTGCAGGGA CCTGTGGTGT TTG-GATCGT
CGAGTGTCGA TGCGTTCGGG CTCCTAAGTC CTCCTGATCG GGGCCATCCA TAGCGGGTGT
CAGGCCTTTA CAGGCGCCCG ACGC-TTCGA CTCGATCTAG GAGTAGGGTT GTTTGGGAAT
GCAGCCCGAA GTGGGTGGTA AACTCCATCT AAGGCTAAAT ACAGACACGA GTCCGATAGA
GGACAAGTAC CGTGAGGGAA AGTTGAAAAG AACTTTGAAG AGAGAGTTCA AGAGTACGTG
AAACCGCCTA GAGGTAAACG GGTGGAACCG CAAA--GTCT GCCCGGGGAA TTCAACAGTG
CACTTTCTCC GCGGAGCGCC ACGACCGGTT TTGCGGCCAG AAGGTCGGGA AGGTGACTAC
T-GCTCTGTA GTGTTATAGC CCGTCGATAC ----TGGGCA CGTCGGGAGA CCGAGGAAGG
GCCGCGGCGA GGGTTCTCGG CCCTCTTCTC GCATTCGAC- -----CTGCA GTGTGAACCG
ACTGTGTGGG GGTCGGGATT CTCGGGTCAG TGGCGAATCG GTCGGTCCTC CACCCGACCC
GTCTTGAAAC ACGGACCAAG GAGTCTAACA TGTGCGCAAG TCATGGGGTC TCGAAACCTA
AAGGCACAAT GAAAGTGAAG GCCGC--TCG TTCGGCCTAG GCGGGATCC- -CCTTGGGCG
CACCGCTGGC CCGTCTCGTC CGA------- ---------- --CTCGTCGG TGAGGCGGAG
CAAGAGCGTA CACGTTGGGA CCCGAAAGAT GGTGAACTAT GCCTGAGTAG GACGAAGCCA
GAGGAAACTC TGGTGGAGGT CCGTAGCGAT TC-CGTAAGT CCACCGGTGG CAAAGCCCCA
AGAAAGCAGT TGGCTACCAA GGCCGCACGT AAAAGTGCCC CAGCCACAGG AGGTGTGAAG
AAACCACACA GATACAGGCC CGGAACCGTC GCCTTGAGAG AAATCCGTCG TTACCAGAAG
AGCACTGAAC TTCTGATCAG GAAACTTCCA TTCCAGCGAC TTGTTCGTGA GATCGCCCAG
GATTTCAAGA CCGATCTGCG ATTCCAGAGC TCAGCTGTCA TGGCTTTGCA AGAGGCTAGC
GAAGCATACC TCGTTGGTCT TTTCGAGGAT ACCAACTTGT GCGCCATCCA CGCCAAAAGA
---------- ---------- ----TTGAGC CGGTATAGTT GGAACATCTC TTAGTCTCTT
AATTCGAGCT GAACTAGGAC AGCCTGGAGC CCTTTTAGGG GACGACCAAC TTTATAACGT
AATCGTGACA GCACATGCAT TTATTATGAT TTTCTTCCTA GTAATACCAA TAATAATAGG
AGGGTTTGGA AATTGATTTG TTCCTATAAT ACTAGGGGCT CCAGACATAG CATTCCCACG
AATAAATAAT ATGAGATTCT GACTTTTACC TCCTGCCTTA ACTCTTCTAT TGGGGTCAGC
TGCTGTAGAA AGGGGGGCTG GAACGGGGTG AACAGTATAC CCACCACTTT CTGGAAATCT
AGCTCACGCA GGTGCCTCAG TAGATCTAAC AATTTTCTCA CTACATTTAG CGGGTGCCTC
ATCAATTATA GCATCAATTA ATTTTATTAC AACAGCAATT AACATACGAT CCCGAGGAAT
ACGATTCGAA CGAATACCTT TATTTGTTTG ATCTATTAAA ATTACAGCTG TTTTACTTCT
TCTTTCACTT CCAGTTCTAG CCGGAGCAAT TACTATACTA TTAACAGACC GAAATTTTAA
CACATCATTC TTCGATCC-- ---------- ---------- ---------- ---------

>Nuculana_pella
---TCATATG CTTGTCTCAA AGACTAAGCC ATGCATGTCT AAGTACAGAC --TCTCAACG
GTGAAACCGC GAAAGGCTCA TTAGATCGGT CAATGTTTAC TGGATCGTAC AATCCTACTT
GGATAACTGT GGCAATTCTA GAGCTAATAC ATGCAACTAA GCTCCGACCT TCGGGGAAGA
GCGCTTTTAT TAGCAAGACC AATCGGTTCG GCTGGTGACT CTGGATAACT TTGGGCTGAT
CGCACGGCCT TGAGCTGGCG ACGTATCCAT CAAATGTCCG CCCTATCAAC TTTCGACGGT
ACGTGATATG CCTACCGTGG TTTTTACGGG TAACGGGGAA TCAGGGTTCG ATTCCGGAGA
GGGAGCATGA GAAACGGCTA CCACATCCAA GGAAGGCAGC AGGCGCGCAA ATTACCCACT
CCTGGCTCAG GGAGGTAGTG ACGAAAAATA ACAATACGGG ACTCTTTCGA GGCCCCGTAA
TTGGAATGAG TACACTTTAA ACCCTTTAAC GAGGATCTAT TGGAGGGCAA GTCTGGTGCC
AGCAGCCGCG GTAATTCCAG CTCCAATAGC GTATATTAAA GTTGTTGCAG TTAAAAAGCT
CGTAGTTGGA TCTCGGGTCC AGGCTTGCGG TCCGTCTCGC GATGGCTGCT CGTCCTGA--
-CCTACTCCC GGT-TCCCTT GGTGCTCTTG ACTGAGTGTC GGTGGCCGGA ACGTTTACTT
TGAAAAAATT AGAGTGTTCA AAGCA--GGC AGTTGCCTGA ATAATGGTGC ATGGAATAAT
GGAATAGGAC CTCGGTTCTA TTTTGTTGGC CTTCGGAGAG GTAATGATTA AGAGGGACGG
ACGGGGGCAT CCGTATTACG GTGTTAGAGG TGAAATTCTT GGATCGCCGT AAGACGAACA
ACTGCGAAAG CATTTGCCAA GAATGTTTTC ATTAATCAAG AACGAAAGTC AGAGGTTCGA
AGACGATCAG ATACCGTCGT AGTTCTGACC ATAAACGATG CCAACTAGCA ATCGGCCGGA

GTTGCTTCAA TGACTCGGCC GGCAGCTTCC GGGAAACCAA AGTTTTTGGG TTCCGGGGGA
AGTATGGTTG CAAAGCTGAA ACTTAAAGGA ATTGACGGAA GGGCACCACC AGGAGTGGAG
CCTGCGGCTT AATTTGACTC AACACGGGAA AACTCACCCG GCCCTGACAC TGTAAGGATT
GACAGATTGA GAGCTCTTTC TTGATTCGGT GGGTGGTGGT GCATGGCCGT TCTTAGTTGG
TGGAGCGATT TGTCTGGTTA ATTCCGATAA CGAACGAGAC TCTAGCCTAC TAAATAGTTC
GCTGATCCGT TGCGTCAGAA CTTCTTAGAG GGACAAGTGG CGTTTAGCCA CACGAGATTG
AGCAATAACA GGTCTGTGAT GCCCTTAGAT GTTCGGGGCC GCACGCGCGC TACACTGAAG
GAATCAGCGT GTTCTTGTCC TTGCCCGAAA GGGTTGGGTA ACCCGTTGAA CCTCCTTCGT
GCTAGGGATT GGGGCTTGTA ATTTTTCCCC ATGAACGAGG AATTCCCAGT AAGCGCGAGT
CATAAGCTCG CGTTGATTAC GTCCCTGCCC TTTGTACACA CCGCCCGTCG CTACTACCGA
TTGAGTGGTT AAGTGAGACC CTGGGACTTG CTAGGCAACT GCCGG----- ---TGCGCAG
GGAACTCGGT CAAACTCCAT TACTTAGAGG AAGTAAAAGT CGTAACAAGG TTTCCATTAC
TAAGCGGAGG GAAAGAAACT AACAAGGATT CCCCCAGTAA CGGCGAGTGA AGCGGGAAGA
GCCCAGCACC GAATCCCGCA GC--CTTGC- GCTGCCGGGA ACTGTGGTGT TTG-GGACGT
CAGCTGTCGC CTCGTTCGGG CGCCCAAGTC CTCCTGATCG GGGCTTCCCA GAGTGGGTGT
CAGGCCTTTA CCGGCGCCTG ACGAGGTGGC --CGTCCTCG GAGTCGGGTT GTTTGGGAAT
GCAGCCCAAA GTGGGTGGTA AACTCCATCT AAGGCTAAAT ACCGACACGA GTCCGATAGC
GGACAAGTAC CGTGAGGGAA AGTTGAAAAG AACTTTGAAG AGAGAGTTCA AGAGTACGTG
AAACCGCTCA GAGGCAAACG GGTGGATCCG CAAA--GTCG ACCCGGGGGA TTCAACGGTG
CACTTTCCCC GTGGAGCGCC ACGACCGGTT GTGCGGTCAT AAGCCCGAGA AGGTAGC---
-CGTCCT--G GTGTTATAGC TCGGCAGTGT ------GGCT CGCCGGACGA CCGAAGGAGT
GCCGCACTCA CTGACTCCGG CCTTCCGCGA CCGTTCGACT G-----TGCA GTGTTCTCCG
ACCGCGGTTG GGACCGGTA- CTAGGGTCAG TGGCGAATCG GTCGGCCCTC CACCCGACCC
GTCTTGAAAC ACGGACCAAG GAGTCTAACA TGTGCGCGAG TCATGGGGTT CCGAAACCTA
AAGGCGCAAT GAAAGTGAAG GCC-GCTCCG GCTGGCCTAG GTAGGATCCC GTC---GGCG
CACTACCGGC CCGTTCCGTC AGC------- ---------- --ACCGTCTG TGGAGCGGAG
CAAGAGCGTA CACGTTGGGA CCCGAAAGAT GGTGAACTAT GCCTGAGTAG GATGAAGCCA
GAGGAAACTC TGGTGGAGGT CCGTAGCGAT TC-AGAAAAT CTACCGGTGG CAAGGCCCCG
AGAAAGCAGC TGGCCACCAA GGCCGCTCGT AAGAGCGCCC CGGCAACCGG YGGTGTGAAG
AAACCTCACA GATACAGGCC AGGAACCGTC GCTCTCCGTG AGATCCGTCG TTACCAGAAG
AGCACCGAAC TTCTCATCCG AAAGCTCCCA TTCCAGCGCC TSGTCCGTGA AATCGCTCAG
GACTTCAAGA CCGATCTGCG TTTCCAGAGC TCCGCCGTCA TGGCCCTCCA GGAGGCTAGC
GAGGCTTACT TGGTTGGTCT GTTTGAGGAC ACCAACTTGT GYGCCATCCA CGCCAAGCGT
TACTCTANNC TTTATTATTG GTATTTGAGC GGGTTTAATA GGAGCTAGGC TTAGTATAGT
TATTCGTATA AATTTACGAG TACCAGGGAG ATGTTTATTG AGGGAGCATT TATATAATGT
GGTTATTACT GCTCATGGGT TATTAATAAT TTTTTTTATG GTTATACCAA TTATGATTGG
GGGCTTTGGT AATTGATTAG TTCCCTTAAT ATTAGGTAGG CCTGACATAT TATATCCTCG
TTTAAATAAT TTAAGGTTTT GGTTGGTACC TTTTGCGTTA GCGTGTTTGC TGATTTCTAT
GTGGGTGGAA GATGGGGCAG GAACGGGGTG AACTATTTAT CCACCTTTAT CTGGTTATAT
AGGCCACAAC GGTCCGTCTG TAGATTTTGC GTGTTTTGCA TTGCATTTAT CAGGGCTATC
TTCTTTATTA GCTTCTATAA ACTTTTTAGC AACTATTGTT ATGATGCGTC CGAGAGATAT
AAAAATGGAA AAGATTCCAT TGTTTCCTTG GTCTTTGTTA GTTACTAGAT TTTTGTTATT
AATTTCTTTA CCAGTGTTAG CAGGGGGTGT GACAATATTA ATTGCGGATC GTAATTTTAA
TACGACGTTT TTTATTCCTG GGGGGGGTGG AGATCCTGTA CTTTTCCAGC ATCTTTTTT

>Neotrigonia_margaritacea
---TCATATG CTTGTCTCAA AGATTAAGCC ATGCATGTCT AAGTACATAC TTTCA--ATA
GTGAAACTGC GAATGGCTCA TTAAATCAGT TATGGTTCCT TAGATCGTAC AATCCTACTT
GGATAACTGT GGTAATTCTA GAGCTAATAC ATGGAACACA GCTCCGACCT CACGGGAAGA
GCGCATTTAT TAGCAAAACC AATCCGGCCG GTTGGTGAAT CTGAATAACT TTGTGCTGAT

CGCATGGCCT CGAGCCGGCG ACGTATCTTT CAAATGTCTG CCCTATCAAC TTTCGATGGT
ACGTGATATG CCTACCATGG TTGTAACGGG TAACGGGGAA TCAGGGTTCG ATTCCGGAGA
GGGAGCATGA GAAACGGCTA CCACATCCAA GGAAGGCAGC AGGCGCGCAA ATTACCCACT
CCCGACACGG GGAGGTAGTG ACGAAAAATA ACAATACGGG ACTCTTTCGA GGCCCCGTAA
TTGGAATGAG TACACTTTAA ACCCTTTAAC GAGGATCCAT TGGAGGGCAA GTCTGGTGCC
AGCAGCCGCG GTAATTCCAG CTCCAATAGC GTATATTAAA GTTGTTGCAG TTAAAAAGCT
CGTAGTTGGA TCTCGGGTCC AGGCTAGCGG TCCACCTCGC GGTGGCTGCC TGTCCTGA--
-CCTACCTGC TGGTTCTCCT GATGCTCTTG ACTGAGTGTC AGTGGCCAGA ACGTTTACTT
TGAAAAAATT AGAGTGTTTA AAGCA--GGC GTGTGCCCGC ATAATGGTGC ATGGAATAAT
GGAATAGGAC CCCGGTTCTA TTTTGTTGGT TTTCGGAGAG GTAATGATTA AGAGGGACTG
ACGGGGGCAT TCGTATTACG GTGTTAGAGG TGAAATTCTT GGATCGCCGT AAGACGAACT
ACTGCGAAAG CATTTGCCAA GAATGTTTTC ATTAATCAAG AACGAAAGTC AGAGGTTCGA
AGACGATCAG ATACCGTCGT AGTTCTGACC ATAAACGATG CCAACTAGCG ATCCGCCGGA
GTTGCTTCAA TGACTCGGCG GGCAGCTTCC GGGAAACCAA AGTTTTTGGG TTCCGGGGGG
AGTATGGTTG CAAAGCTGAA ACTTAAAGGA ATTGACGGAA GGGCACCACC AGGAGTGGAG
CCTGCGGCTT AATTTGACTC AACACGGGAA AACTCACCCG GCCCGGACAC TGTAAGGATT
GACAGATTGA GAGCTCTTTC TTGATTCGGT GGGTGGTGGT GCATGGCCGT TCTTAGTTGG
TGGAGCGATT TGTCTGGTTA ATTCCGATAA CGAACGAGAC TCTAGCCTAT TAACTAGTTC
GCCGATCCGT TGCGTCGGAA CTTCTTAGAG GGACAAGTGG CTTTTAGCCA CACGAGATTG
AGCAATAACA GGTCTGTGAT GCCCTTAGAT GTTCGGGGCC GCACGCGCGC TACACTGAAG
GAATCAACGT GCATTTGCCC TTGTCCGGCA GGATTGGGTA ACCCGTTGAA CCTCCTTCGT
GCTAGGGATT GGGGCTTGTA ATTTTTCCCC ATGAACGAGG AATTCCCAGT AAGCGCGAGT
CATAAGCTCG CGTTGATTAC GTCCCTGCCC TTTGTACACA CCGCCCGTCG CTACTACCGA
TTGAATGGTT TAGTGATATC CTCGGATTGG TTGGGCAACT GTCGAGT--- ---TGCGCCG
AGAAGACGAT AGAACTTGAT CATTTAGAGG AAGTAAAAGT CGTAACAAGG TTTCC-----
---------- ---------- ---------- --CCCAGTAA CGGCGAGTGA AGCGGGAAAA
GCCCAGCACC GAATCCCACA GC--CTGGC- GCTGTCGGGA ACTGTGGTGT -ATAGGCAGC
CTAGTGTCGA CGTGTTCGGG TCCCCAAGTC CTCCTGATCG TGGCCTCCCA TAGCGGGTGT
CAGGCCCGTA CAGGCACCCG ACGGCGTCGG CTCTACCTTG GAGTCGGGTT GTTTGGGAAT
GCAGCCCAAA GCGGGTGGTA AACTCCATCT AAGGCTAAAT ACCGACACGA GTCCGATAGC
GGACAAGTAC CGTGAGGGAA AGTTGAAAAG AACTTTGAAG AGAGAGTTCA AGAGTACGTG
AAACCGCTTA GAGGCAAACG GGTGGACCCG CAAA--GTCG ACCCGGGGGA TTCAACGGTG
CACTTTCTCC GCCGAGCGCC ACGACCGGCT CGACGGCCAG AAGCACGGGA AGGTGACTTG
CCGCTTCAAG AGTTTATAGC CCGCCAGCGC ------GGCT CGTCGGCGGG CCGAGGAGGG
GCCGTGTCTC CGCTTTCGGG CCATCCGTTC ACGTCCGACT C-----CGCA GTGCTCTCGG
ACTGCGTGTG GGTTCGTCTA CACGGGCCAG TGGCGAATCG GTCGGTCCTC CACCCGACCC
GTCTTGAAAC ACGGACCAAG GAGTCTAACA TGTGCGCGAG TCATGGGGTC GCGAAACCTA
AAGGCGCAAT GAAAGTGAAG GCCGCCTCGG GTGTGCCTAG GTACGATCCT CCCGCAGGCG
CAGTACCGGC CCGTCTCGTC CG-------- ---------- --CTCGACGG GGAGGCGGAG
CAAGAGCGTA CACGTTGGGA CCCGAAAGAT GGTGAACTAT GCTTGAGTAG GACGAAGCCA
GAGGAAACTC TGGTGGAGGT CCGTAGCGAT TC-CGCAAGT CCACCGGTGG GAAGGCTCCC
AGGAAACAGC TGGCCACAAA GGCCGCTCGT AAGAGCGCCC CAGCTACTGG TGGTGTGAAG
AAACCACACA GATACAGGCC CGGAACCGTT GCCCTGAGAG AAATCAGACG TTACCAGAAG
AGCACGGAGC TTCTCATCAG GAAACTGCCC TTCCAGCGTC TCGTCCGCGA AATCGCTCAG
GACTTCAAGA CTGACCTGCG TTTCCAGAGC TCCGCCGTGA TGGCCCTTCA AGAGGCCAGC
GAGGCTTACC TGGTGGGTCT GTTTGAGGAC ACGAACTTGT GCGCTATCCA CGCCAAGAGA
---------- ---------- ----ATGGTC TGGACTTATT GGTTTGGCTT TAAGATTATT
GATTCGTGCT GAGTTGGGAC AACCAGGAAG TTTATTGGGT GATGATCAAT TGTATAATGT
AATTGTAACT GCCCATGCTT TTATAATGAT TTTTTTCTTG GTAATACCTA TGATGATTGG
GGGGTTTGGA AATTGATTAC TACCTTTAAT GTTAGGAGCT CCGGATATGG CTTTCCCCCG

```
TCTAAATAAC ATAAGATTTT GATTATTAGT GCCTGCATTG TTTTTGTTAT TAAGATCTTC
TTTAGTTGAA AGAGGTGTTG GAACTGGATG GACAGTTTAT CCCCCTTTAT CAGGAAATAT
TGGCCATTCT GGTCCTTCCG TGGATTTAGC TATTTTTTCT TTACATTTGG CGGGGGCATC
TTCTATTTTA GGGTCTATTA ATTTTATCAG GACTATTAGA AATATACGAC CTTTTGGTTT
GAGGGCAGAG CGATTGCCTT TATTTTTATG GGCTGTATGT GTGACTGCTA TTTTATTAGT
GATTGCATTA CCTGTGTTGG CAGGGGCAAT TACTATGCTT TTGACAGATC GTAATTTGAA
CACATCTTTT TTTGACCCGA CAGGTGGAGG AGACCCA--- ---------- ---------
```
