# Supplementary material for: Molecular phylogeny of the bivalve superfamily Galeommatoidea (Heterodonta, Veneroida) reveals dynamic evolution of symbiotic lifestyle and interphylum host switching
Source: BMC Evol Biol. 2012 Sep 6;12:172. doi: 10.1186/1471-2148-12-172 (PMC3532221; doi:10.1186/1471-2148-12-172)
Supplement: Additional file 3 — Morphological and ecological accounts for each clade within Galeommatoidea. [file 1471-2148-12-172-S3.doc]

**Additional file 3. Morphological and ecological accounts for each clade within Galeommatoidea.** Our study revealed that Galeommatoidea comprises at least six major clades. Below, we give accounts on the morphology, host association, and ecological adaptation to symbiotic life of the members constituting each of the major clades.

**Clade 1**

Clade 1 comprises mainly free-living species, most of which attach to the undersurfaces of rocks in the intertidal zone [1] (Figure 1A, C). According to the classification of Bieler *et al*. (2010), most members of Clade 1 belong to Galeommatidae [2]; however, the free-living species of genera belonging to Lasaeidae (i.e., *Kellia*, *Lasaea*, *Melliteryx*, *Parabornia*, and *Pythina*) are also included in this major clade. Some galeommatid species gape their valves widely and are limpet-like (Figure 1A, B). Our phylogenetic analysis suggested that these limpet-like species (*Ephippodonta gigas, Galeomma* sp., *Pseudogaleomma* sp. and *Scintilla rosea*) comprise a monophyletic group (Figure 2). *Arthritica japonica* (Figure 1D), *Divariscintilla toyohiwakensis*, and *E. gigas* (Figure 1B) were exceptional in being symbiotic with crustaceans; the first species lives on the body surface of intertidal crabs [3] (Table 1) and the other two in the burrows of thalassinidean shrimps and mantis shrimps, respectively [1,4] (Table 1). As previously reported [3,4], the host species of *A. japonica* and *D. toyohiwakensis* are *Xenophthalmus pinnotheroides* and *Acanthosquilla acanthocarpus*, respectively. The host of *E. gigas* was reported to be a thalassinidean shrimp in Phuket [1], and, in this study, the host was found to be *Callianidea typa* (Table 1).

**Clade 2**

Clade 2 includes both free-living and symbiotic species. *Mysella* aff. *bidentata* (Figure 1T) and *Nipponomysella oblongata* are free-living species that inhabit sand (Goto, Ishikawa & Hamamura, unpublished data), *Nipponomontacuta actinariophila* (Figure 1U) and *Montacutona* sp. live on the body surface of sea anemones [5], and *Curvemysella paula* (Figure 1S) lives inside the shell carried by hermit crabs [6]. The close relatedness of *Nipponomontacuta* and *Montacutona* suggests an identical origin of their association with sea anemones. The host of *N. actinariophila* was recorded as *Halcampella maxima* [5]. However, we found *N. actinariophila* associated with *Telmatactis* sp.

The shell shape of *C. paula* is markedly curved (Figure 1S) and differs from that of *Mysella* (Figure 1T) or *Nipponomysella*, genera considered to be closely related to *Curvemysella* [6]. Thus, the unique shell of *Curvemysella* has been considered as an adaptation to symbiotic life in the shells carried by hermit crabs [6]. In this study, we confirmed the close relationship between *Curvemysella*, *Nipponomysella*, and *Mysella*, which supports the morphology-based assumption described above.

**Clade 3**

Clade 3 includes only bivalves belonging to the genus *Basterotia*, all species of which live within echiuran burrows (Figure 1V) [7,8]. *Basterotia* bivalves differ morphologically from other galeommatoids in having posterior inhalant and external siphons [7]. On the basis of these characteristics, *Basterotia* has often been included in Cyamioidea [9,10]. However, a recent study of the molecular phylogeny of heterodont bivalves suggested that *Basterotia* belongs to Galeommatoidea [11]. Also, a recent morphological and ecological assessment of this bivalve genus supported the inclusion of the genus in Galeommatoidea [7]. Our molecular phylogenetic analysis found that *Basterotia* comprises a monophyletic clade within Galeommatoidea (Figures 2). The unique morphological characteristics of *Basterotia* bivalves can be considered adaptations to symbiotic life within echiuran burrows [7]. However, we did not determine whether such unique characteristics are a basal or derived condition within Galeommatoidea because the relationships among the major clades were not well resolved (Figure 2).

**Clade 4**

Clade 4 comprises only symbiotic bivalves that are associated with various invertebrate hosts, such as crustaceans, holothurians, sipunculans, and echiurans (Figure 1E–K). Of the seven species, three live in the host burrow (holothurian and echiuran), whereas five directly attach to the host body (mantis shrimp, upogebid shrimp, intertidal crab, and sipunculan) (Figure 3). This range of hosts suggests that interphylum host switches occurred frequently in this major clade, in addition to frequent host-use switches. Because of low branch support, it is not clear how many times symbiotic associations with each animal group and each host-use pattern originated in this clade (Figure 3). The host of each bivalve species matched those previously recorded (see Additional file 4).

*Peregrinamor*, bivalves that attach to the abdomen of upogebid shrimps (Figure 1H), has been placed in Mytiloidea because of their oblong shell that differs from that of other galeommatoideans (Figure 1H) [9]. However, it was suggested that *Peregrinamor* belongs to Galeommatoidea based on their anatomy and ecology [12]. Our results also show that *Peregrinamor* is grouped within Galeommatoidea (Figure 3), which supports their hypothesis.

**Clade 5**

*Neaeromya rugifera* attaches to the abdomen of the upogebid shrimp *Upogebia pugettensis* [13,14] (Figure 1R).

**Clade 6**

Most members of Clade 6 are commensal bivalves that attach directly to the host body (Figure 1L–Q), although *Entovalva* has the intriguing habit of living inside the host esophagus. The hosts of this major clade are sipunculans, holothurians, and echinoids (Figure 3), suggesting that host switching between distantly related taxa has occurred frequently in this major clade.

Three bivalves species associated with the sipunculans have different modes of host utilization [15-17]. *Salpocola philippinensis* attaches to the skin near the anus via strong byssus threads [15], whereas *Litigiella pacifica* either attaches to the skin or lives inside the host burrow [16], and *Nipponomysella subtruncata* attaches to the skin near the mid-body of the host [17] (Figure 1M–O). These three species have differing shell shapes and sizes [15-17]. Therefore, it is possible that different commensal lifestyles cause different morphological and ecological adaptations to the sipunculan host. The former two species are associated with *Sipunculus nudus*, and the latter with *Siphonosoma cumanense* (Table 1).

*Devonia*, *Anisodevonia*, and *Entovalva* are associated with the holothurians (Table 1; Figure 1P, Q) [18-20]. The former two species attach to the host body surface (Figure 1P, Q), whereas the latter lives inside the host esophagus. It was suggested that the endosymbiotic species (*Entovalva*) evolved from ectosymbiotic species based on their morphological and ecological characteristics [20]. Our molecular phylogenetic analyses supported this hypothesis.

*Scintilla stigmatica* lives among the spines on the ventral surfaces of the heart urchin *Brissus lateracarinatus* [21,22] (Figure 1L)*.*

**References**

1. Lützen J, Nielsen C: **Galeommatid bivalves from Phuket, Thailand.** *Zool J Linn* 2005, **144:**261-306.

2. Bieler R, Carter GJ, Coan EV: **Nomenclator of bivalve families with a classification of bivalve families Part2. Classification of bivalve families.** *Malacologia* 2010, **52:**113-184.

3. Lützen J, Takahashi T: ***Arthritica japonica*, sp. nov. (Bivalvia: Galeommatoidea: Leptonidae), a commensal with the pinnotherid crab *Xenophthalmus pinnotheroides* White, 1846.** *Yuriyagai* 2003, **9:**11-19.

4. Yamashita H, Haga T, Lützen J: **The bivalve *Divariscintilla toyohiwakensis* n. sp. (Heterodonta: Galeommatidae) from Japan, a commensal with a mantis shrimp.** *Venus* 2011, **69:**123-133.

5. Yamamoto T, Habe T: ***Nipponomontacuta actinariophila* gen. et sp. nov, a new commensal bivalve of sea anemone.** *Publ Seto Mar Biol Lab* 1961, **9:**265-266.

6. Goto R, Hamamura Y, Kato M: **Obligate commensalism of *Curvemysella paula* (Bivalvia: Galeommatidae) with hermit crabs.** *Mar Biol* 2007, **151:**1615-1622.

7. Goto R., Hamamura Y, Kato M: **Morphological and ecological adaptation of Basterotia bivalves (Galeommatoidea: Sportellidae) to symbiotic association with burrowing echiuran worms.** *Zool Sci* 2011, **28:**225-234.

8. Goto R, Kato M: **Geographic mosaic of mutually exclusive dominance of obligate commensals in symbiotic communities associated with a burrowing echiuran worm.** *Mar Biol*, **159:**319-330.

9. Vaught KC: *A classification of the living Mollusca*. Melborne: American Malacologists Incorporation; 1989.

10. Coan EV: **The eastern Pacific Sportellidae (Bivalvia).** *Veliger* 1999, **42:**132-151.

11. Taylor JD, Williams ST, Glover EA, Dyal P: **A molecular phylogeny of heterodont bivalves (Mollusca: Bivalvia: Heterodonta): new analyses of 18S and 28S rRNA genes.** *Zool Scrip* 2007, **36:** 587-606.

12. Kato M, Itani G: **Commensalism of a bivalve, *Pereginamor ohshimai*, with a thalassinidean burrowing shrimp, *Upogebia major.*** *J Mar Biol Assoc UK* 1995, **75:**941-947.

13. Narchi W: **On *Pseudopythina rugifera* (Carpenter, 1864) (Bivalvia).** *Veliger* 1969, **12:**43-52.

14. Ó Foighil D: **Form, function, and origin of temporary dwarf males in *Pseudopythina rugifera* (Carpenter, 1864) (Bivalvia: Galeommataceae).** *Veliger* 1985, **27:**245-252.

15. Habe T, Kanazawa T: **A new commensal bivalve from the Philippines (Montacutidae).** *Venus* 1981, **40:**123-124.

16. Lützen J, Kosuge T: **Description of the bivalve *Litigiella pacifica* n. sp. (Heterodonta: Galeommatoidea: Lasaeidae), commensal with the sipuculan *Sipunculus nudus* from the Ryukyu Islands, Japan.** *Venus* 2006, **65:**193-202.

17. Lützen J, Takahashi T, Yamaguchi T: **Morphology and reproduction of *Nipponomysella* *subtruncata* (Yokoyama), a galeommatoidean bivalve commensal with the sipunculan *Siphonosoma cumanense* (Keferstein) in Japan.** *J Zool* 2001, **254:**429-440.

18. Ohshima H: **On *Entovalva semperi* Ohshima, an aberrant commensal bivalve.** *Venus* 1931, **2:**161-177.

19. Kawahara T: **On *Devonia oshimai* sp. nov., a commensal bivalve attached to the Synaptid *Leptosynapta ooplax*.** *Venus* 1942, **11:**153-164.

20. Kato M: **Morphological and ecological adaptations in montacutid bivalves endo- and ecto-symbiotic with holothurians.** *Can J Zool* 1998, **76:**1403-1410.

21. Pilsbry HA: **Marine Mollusks of Hawaii, VIII–XIII.** *Proc Acad Natl Sci Philad* 1920, **72:**296­­­-328.

22. Yamamoto T, Habe T: ***Scintillona stigmatica* (Pirsbry) new to Japan.** *Venus* 1974, **33:** 116.
