# Supplementary material for: Molecular phylogeny of the bivalve superfamily Galeommatoidea (Heterodonta, Veneroida) reveals dynamic evolution of symbiotic lifestyle and interphylum host switching
Source: BMC Evol Biol. 2012 Sep 6;12:172. doi: 10.1186/1471-2148-12-172 (PMC3532221; doi:10.1186/1471-2148-12-172)
Supplement: Additional file 4 — Host information of each galeommatoidean species sampled. [file 1471-2148-12-172-S4.doc]

**Additional file 4. The host information of each galeommatoidean species we sampled.**

**References**

1. Yamashita H, Haga T, Lützen J: **The bivalve *Divariscintilla toyohiwakensis* n. sp. (Heterodonta: Galeommatidae) from Japan, a commensal with a mantis shrimp.** *Venus* 2011, **69:**123–133.

2. Lützen J, Nielsen C: **Galeommatid bivalves from Phuket, Thailand.** *Zool J Linn* 2005, **144:**261–306.

3. Pilsbry HA: **Marine Mollusks of Hawaii, VIII–XIII.** *Proc Acad Natl Sci Philad* 1920, **72:**296­­­–328.

4. Yamamoto T, Habe T: ***Scintillona stigmatica* (Pirsbry) new to Japan.** *Venus* 1974, **33:** 116.

5. Kawahara T: **On *Devonia oshimai* sp. nov., a commensal bivalve attached to the Synaptid *Leptosynapta ooplax*.** *Venus* 1942, **11:**153-164.

6. Kato M: **Morphological and ecological adaptations in montacutid bivalves endo- and ecto-symbiotic with holothurians.** *Can J Zool* 1998, **76:**1403-1410.

7. Lützen J, Takahashi T: ***Arthritica japonica*, sp. nov. (Bivalvia: Galeommatoidea: Leptonidae), a commensal with the pinnotherid crab *Xenophthalmus pinnotheroides* White, 1846.** *Yuriyagai* 2003, **9:**11–19.

8. Goto R, Kato M: **Geographic mosaic of mutually exclusive dominance of obligate commensals associated with a burrowing echiuran worm.** *Mar Biol*, in press.

9. Goto R, Hamamura Y, Kato M: **Obligate commensalism of *Curvemysella paula* (Bivalvia: Galeommatidae) with hermit crabs.** *Mar Biol* 2007, **151:**1615–1622.

10. Ohshima H: **On *Entovalva semperi* Ohshima, an aberrant commensal bivalve.** *Venus* 1931, **2:**161–177.

11. Lützen J, Kosuge T: **Description of the bivalve *Litigiella pacifica* n. sp. (Heterodonta: Galeommatoidea: Lasaeidae), commensal with the sipuculan *Sipunculus nudus* from the Ryukyu Islands, Japan.** *Venus* 2006, **65:**193–­202.

12. Narchi W: **On *Pseudopythina rugifera* (Carpenter, 1864) (Bivalvia).** *Veliger* 1969, **12:**43-52.

13. Ó Foighil D: **Form, function, and origin of temporary dwarf males in *Pseudopythina rugifera* (Carpenter, 1864) (Bivalvia: Galeommataceae).** *Veliger* 1985, **27:**245-252.

14. Yamamoto T, Habe T: ***Nipponomontacuta actinariophila* gen. et sp. nov, a new commensal bivalve of sea anemone.** *Publ Seto Mar Biol Lab* 1961, **9:**265-266.

15. Lützen J, Takahashi T, Yamaguchi T: **Morphology and reproduction of *Nipponomysella* *subtruncata* (Yokoyama), a galeommatoidean bivalve commensal with the sipunculan *Siphonosoma cumanense* (Keferstein) in Japan.** *J Zool* 2001, **254:**429-440.

16. Kato M, Itani G: ***Peregrinamor gastrochaenans* (Bivalvia: Mollusca), a new species symbiotic with the thalassinidean shrimp *Upogebia carinicauda* (Decapauda: Crustacea).** *Species Diversity* 2000, **5:**11–30.

17. Kato M, Itani G: **Commensalism of a bivalve, *Peregrinamor ohshimai*, with a thalassinidean burrowing shrimp, *Upogebia major*.** *J Mar Biol Assoc UK* 1995, **75:**941–947.

18. Morton B, Scott PH**. The Hong Kong Galeommatacea (Mollusca: Bivalvia) and their hosts, with descriptions of new species.** *Asian Mar Biol* 1989, **6:**129–160.

19. Morton B: **Some aspects of the functional morphology and biology of *Pseudopythina subsinuata* (Bivalvia: Jeptonacea) commensal on stomatopod crustaceans.** *J Zool* 1972, **166:**79-96.

20. Habe T, Kanazawa T: **A new commensal bivalve from the Philippines (Montacutidae).** *Venus* 1981, **40:**123–124.

21. Goto R, Hamamura Y, Kato M: **Morphological and ecological adaptation of Basterotia bivalves (Galeommatoidea: Sportellidae) to symbiotic association with burrowing echiuran worms.** *Zool Sci* 2011, **28:**225–234.
