# Supplementary material for: Molecular phylogeny of the bivalve superfamily Galeommatoidea (Heterodonta, Veneroida) reveals dynamic evolution of symbiotic lifestyle and interphylum host switching
Source: BMC Evol Biol. 2012 Sep 6;12:172. doi: 10.1186/1471-2148-12-172 (PMC3532221; doi:10.1186/1471-2148-12-172)
Supplement: Additional file 6 — Information on primers and PCR conditions used in this study. [file 1471-2148-12-172-S6.doc]

**Additional file 6. Information on primers and PCR conditions used in this study.**

**References**

1. Saunders GW, Kraft GT: **Small-subunit rRNA gene sequences from representatives of selected families of the Gigartinales and Rhodymeniales (Rhodophyta).1. Evidence for the Plocamiales ord. nov.** *Can J Bot* 1994, **72:**1250-1263.
2. Wollscheid E, Wägele H: **Initial results on the molecular phylogeny of the Nudibranchia (Gastropoda, Opisthobranchia) based on 18S rDNA data.** *Mol Phylogenet Evol* 1999, **13:**215-226.
3. Hillis DM, Dixon MT: **Ribosomal DNA: Molecular evolution and phylogentic inference.** *Q Rev Biol* 1991, **66:**411-453.
4. Hoso M, Kameda Y, Wu, SP, Asami T, Kato M, Hori M: **A speciation gene for left-right reversal in snails results in anti-predator adaptation.** *Nat Comm* 2010, **1:**133.
5. Colgan DJ, Ponder WF, Beacham E, Macaranas JM: **Gastropod phylogeny based on six segments from four genes representing coding or non-coding and mitochondrial or nuclear DNA.** *Molluscan Res* 2003, **23:**123-148.
6. Vonnemann V, Schrödl M, Klussmann-Kolb A, Wägele H: **Reconstruction of the Phylogeny of the Opisthobranchia (Mollusca: Gastropoda) by means of 18S and 28S rRNA Gene Sequences.** *J Molluscan Stud* 2005, **71:**113-125.
7. Dayrat B, Tillier A, Lecointre G, Tillier S: **New Clades of Euthyneuran Gastropods (Mollusca) from 28S rRNA Sequences.** *Mol Phylogenet Evol* 2001, **19:**225-235.
8. Colgan DJ, McLauchlan A, Wilson GDF, Livingston S, Macaranas J, Edgecombe GD, Cassis G, Gray MR: **Molecular phylogenetics of the Arthropoda: relationships based on histone H3 and U2 snRNA DNA sequences.** *Aust J Zool* 1998, **46:**419-437.
9. Folmer O, Black M, Hoeh W, Lutz RA, Vrijenhoek R: **DNA primers for amplification of mitochondrial cytochrome c oxidase**

**subunit I from diverse metazoan invertebrates.** *Mol Mar Biol Biotechnol* 1994, 3:294-299.
