# Supplementary material for: Molecular phylogeny of the bivalve superfamily Galeommatoidea (Heterodonta, Veneroida) reveals dynamic evolution of symbiotic lifestyle and interphylum host switching
Source: BMC Evol Biol. 2012 Sep 6;12:172. doi: 10.1186/1471-2148-12-172 (PMC3532221; doi:10.1186/1471-2148-12-172)
Supplement: Additional file 7 — Information on sequence alignment and models of sequence evolution for the maximum likelihood analysis. [file 1471-2148-12-172-S7.doc]

**Additional file 7. Information on sequence alignments and models of sequence evolution for maximum likelihood analysis.**

­
