# Supplementary material for: Molecular phylogeny of the bivalve superfamily Galeommatoidea (Heterodonta, Veneroida) reveals dynamic evolution of symbiotic lifestyle and interphylum host switching
Source: BMC Evol Biol. 2012 Sep 6;12:172. doi: 10.1186/1471-2148-12-172 (PMC3532221; doi:10.1186/1471-2148-12-172)
Supplement: Additional file 8 — Maximum likelihood tree of Galeommatoidea based on each partition. Numbers above branches indicate maximum-likelihood bootstrap support values followed by Bayesian posterior probabilities. Especially long branches are broken down to fit the page. [file 1471-2148-12-172-S8.pdf]

Additional file 8. Maximum likelihood tree of Galeommatoidea based on each partition. Numbers above branches indicate maximum-likelihood bootstrap support values followed by Bayesian posterior probabilities. Especially long branches are broken down to fit the page.

(a) 18S

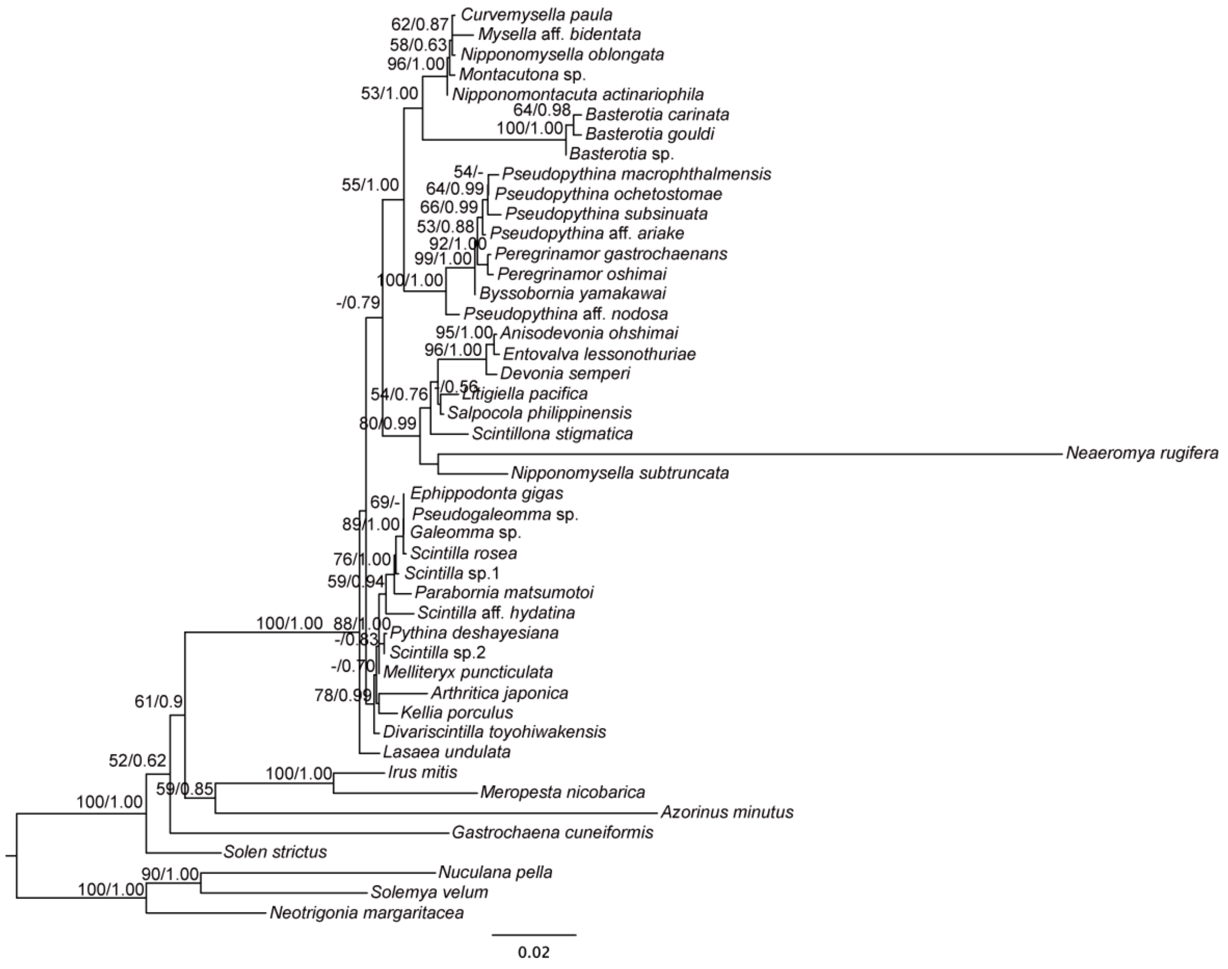

(b) 28S

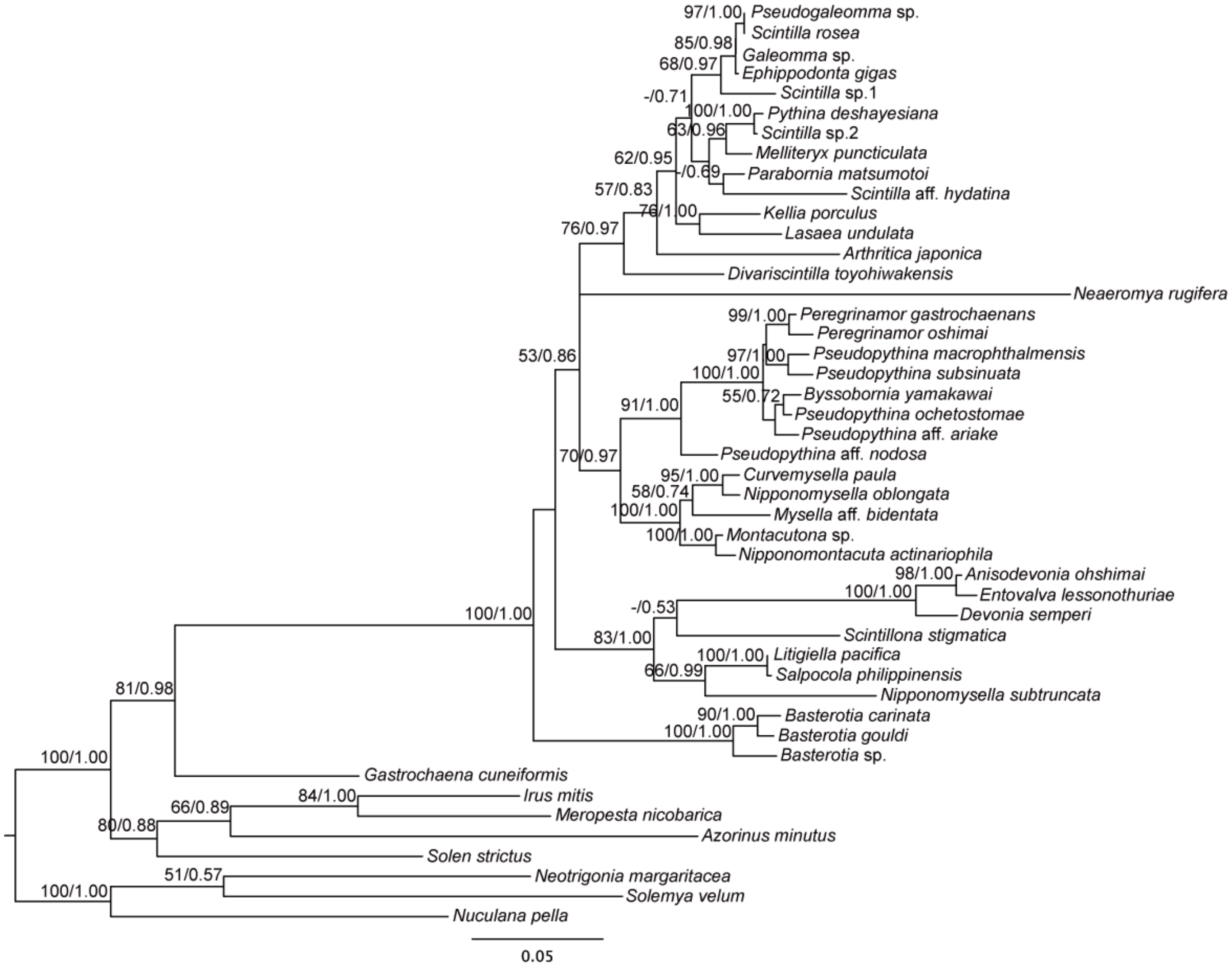
97/1.00
Pseudogaleomma sp.
Scintilla rosea
85/0.98
Galeomma sp.
68/0.97
Ephippodonta gigas
Scintilla sp.1
-/0.71
100/1.00
Pythina deshayesiana
63/0.96
Scintilla sp.2
Melliteryx puncticulata
62/0.95
-/0.69
Parabornia matsumotoi
Scintilla aff. hydatina
57/0.83
76/1.00
Kellia porculus
Lasaea undulata
76/0.97
Arthritica japonica
Divariscintilla toyohiwakensis
Neaeromya rugifera
99/1.00
Peregrinamor gastrochaenans
Peregrinamor oshimai
53/0.86
97/1.00
Pseudopythina macrophthalmensis
100/1.00
Pseudopythina subsinuata
55/0.72
Byssobornia yamakawai
Pseudopythina ochetostomae
91/1.00
Pseudopythina aff. ariake
70/0.97
Pseudopythina aff. nodosa
95/1.00
Curvemysella paula
58/0.74
Nipponomysella oblongata
100/1.00
Mysella aff. bidentata
100/1.00
Montacutona sp.
Nipponomontacuta actinariophila
98/1.00
Anisodevonia ohshimai
-/0.53
100/1.00
Entovalva lessonothuriae
Devonia semperi
100/1.00
Scintillona stigmatica
83/1.00
100/1.00
Litigiella pacifica
66/0.99
Salpocola philippinensis
Nipponomysella subtruncata
81/0.98
90/1.00
Basterotia carinata
100/1.00
Basterotia gouldi
Basterotia sp.
100/1.00
Gastrochaena cuneiformis
66/0.89
84/1.00
Irus mitis
Meropesta nicobarica
80/0.88
Azorinus minutus
Solen strictus
51/0.57
Neotrigonia margaritacea
100/1.00
Solemya velum
Nuculana pella
0.05

(c) H3

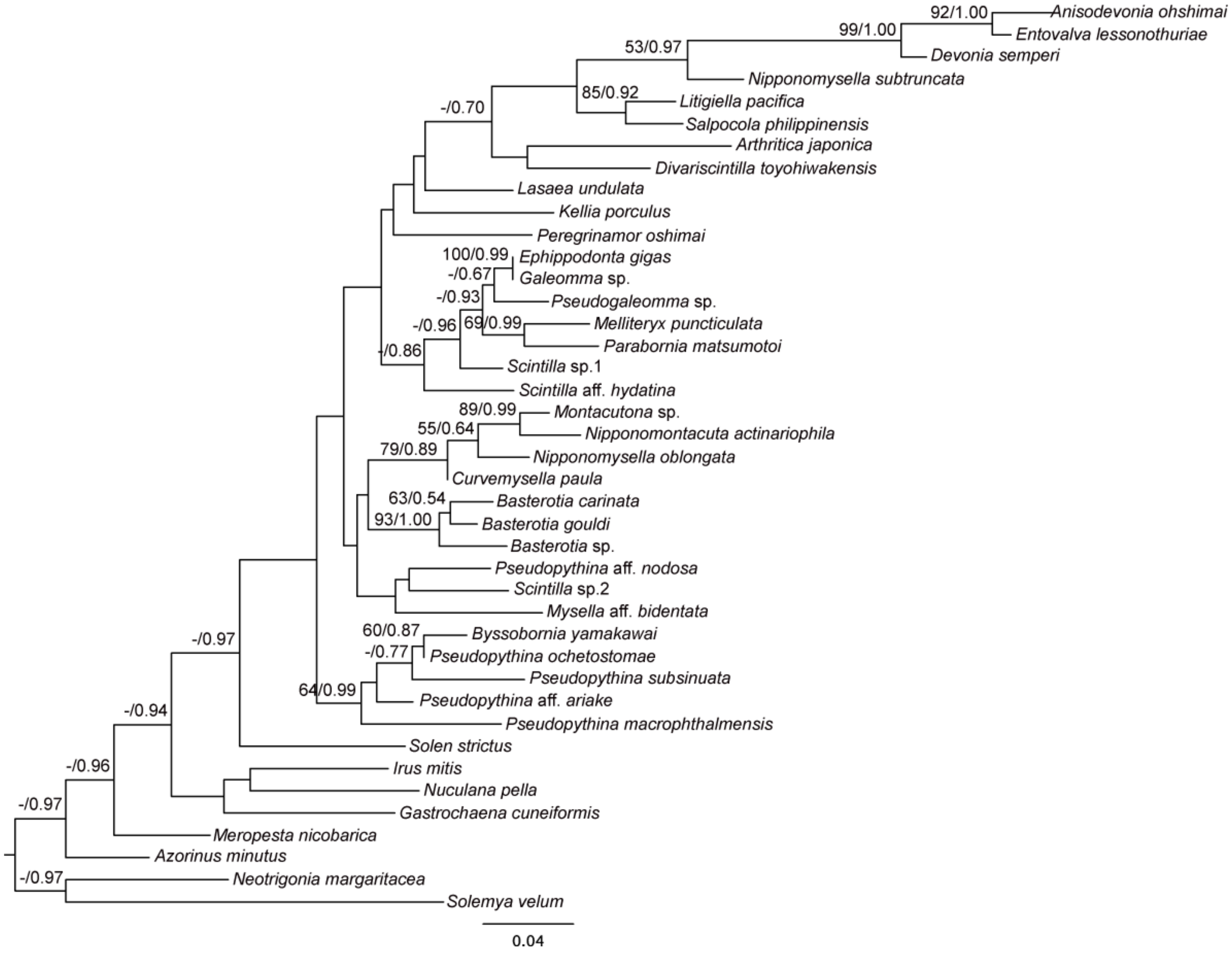

92/1.00
Anisodevonia ohshimai
99/1.00
Entovalva lessonothuriae
53/0.97
Devonia semperi
Nipponomysella subtruncata
85/0.92
Litigiella pacifica
-/0.70
Salpocola philippinensis
Arthritica japonica
Divariscintilla toyohiwakensis
Lasaea undulata
Kellia porculus
Peregrinamor oshimai
100/0.99
Ephippodonta gigas
-/0.67
Galeomma sp.
-/0.93
Pseudogaleomma sp.
-/0.96
69/0.99
Melliteryx puncticulata
Parabornia matsumotoi
-/0.86
Scintilla sp.1
Scintilla aff. hydatina
89/0.99
Montacutona sp.
55/0.64
Nipponomontacuta actinariophila
79/0.89
Nipponomysella oblongata
Curvemysella paula
63/0.54
Basterotia carinata
93/1.00
Basterotia gouldi
Basterotia sp.
Pseudopythina aff. nodosa
Scintilla sp.2
Mysella aff. bidentata
60/0.87
Byssobornia yamakawai
-/0.97
-/0.77
Pseudopythina ochetostomae
Pseudopythina subsinuata
64/0.99
Pseudopythina aff. ariake
-/0.94
Pseudopythina macrophthalmensis
Solen strictus
-/0.96
Irus mitis
Nuculana pella
-/0.97
Gastrochaena cuneiformis
Meropesta nicobarica
Azorinus minutus
-/0.97
Neotrigonia margaritacea
Solemya velum
0.04

(d) COI

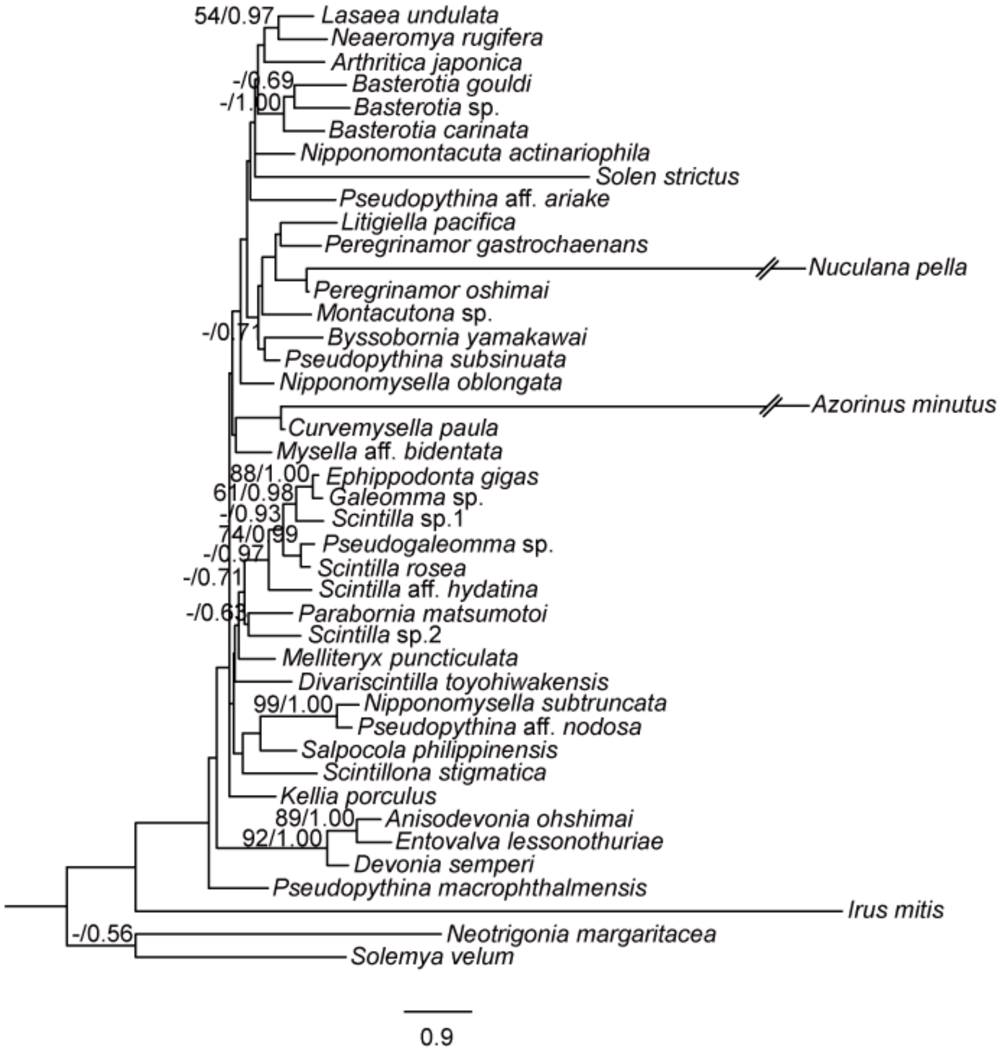

54/0.97
Lasaea undulata
Neaeromya rugifera
Arthritica japonica
-/0.69
Basterotia gouldi
-/1.00
Basterotia sp.
Basterotia carinata
Nipponomontacuta actinariophila
Solen strictus
Pseudopythina aff. ariake
Litigiella pacifica
Peregrinamor gastrochaenans
Nuculana pella
Peregrinamor oshimai
Montacutona sp.
-/0.71
Byssobornia yamakawai
Pseudopythina subsinuata
Nipponomysella oblongata
Azorinus minutus
Curvemysella paula
Mysella aff. bidentata
88/1.00
Ephippodonta gigas
61/0.98
Galeomma sp.
-/0.93
Scintilla sp.1
74/0.99
Pseudogaleomma sp.
-/0.97
Scintilla rosea
-/0.71
Scintilla aff. hydatina
-/0.63
Parabornia matsumotoi
Scintilla sp.2
Melliteryx puncticulata
Divariscintilla toyohiwakensis
99/1.00
Nipponomysella subtruncata
Pseudopythina aff. nodosa
Salpocola philippinensis
Scintillona stigmatica
Kellia porculus
89/1.00
Anisodevonia ohshimai
92/1.00
Entovalva lessonothuriae
Devonia semperi
Pseudopythina macrophthalmensis
Irus mitis
-/0.56
Neotrigonia margaritacea
Solemya velum
0.9
